# Domino Conjugate Addition-1,4-Aryl Migration for the Synthesis of α,β-Difunctionalized Amides

**DOI:** 10.1021/jacsau.4c00378

**Published:** 2024-06-17

**Authors:** Haoqi Zhang, Yi Xiao, Miran Lemmerer, Tommaso Bortolato, Nuno Maulide

**Affiliations:** † Institute of Organic Chemistry, University of Vienna, Währinger Straße 38, 1090 Vienna, Austria; ‡ Vienna Doctoral School in Chemistry, University of Vienna, Währinger Straße 42, 1090 Vienna, Austria; § Christian-Doppler Laboratory for Entropy-Oriented Drug Design, Josef-Holaubek-Platz 2, 1090 Vienna, Austria; ∥ CeMM Research Center for Molecular Medicine of the Austrian Academy of Sciences, Lazarettgasse 14, AKH BT 25.3, 1090 Vienna, Austria

**Keywords:** rearrangement, amides, Michael addition, aromatic substitution, organocatalysis

## Abstract

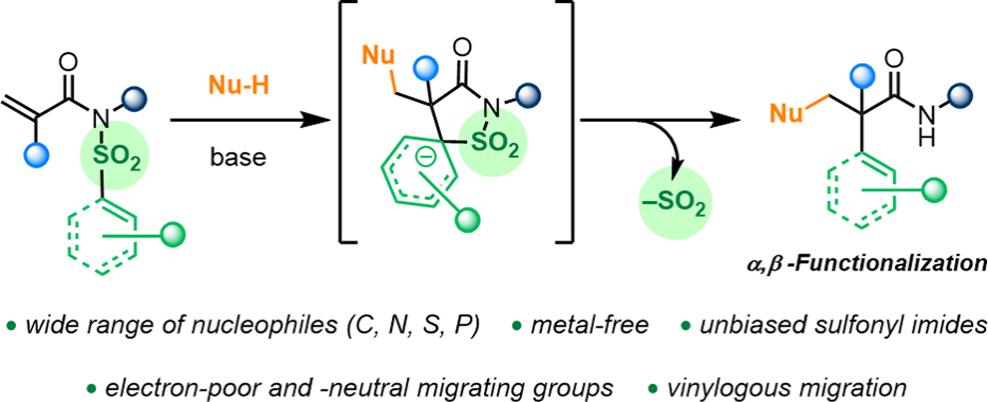

A domino difunctionalization of sulfonyl(acryl)imides to form β-substituted α-aryl amides is reported. This transformation involves a 1,4-addition followed by a polar Truce–Smiles rearrangement process, entropically driven by release of SO_2_. A wide range of carbon- and heteroatom-based nucleophiles and sulfonyl imides were employed, allowing rapid access to highly functionalized amides. In contrast to related reactions with a radical pathway, unbiased substrates could be employed. Despite the usual requirement of an electron-poor migrating moiety for the S_N_Ar event, we herein report unique and unprecedented vinylogous migrations of electron-neutral arenes. Additionally, a one-pot process toward β-amido amides starting from acrylic acids has been developed.

C–C bonds remain the quintessential connectivity of organic chemistry.^1^ A range of classical methods for C–C bond formation have been reported throughout the years, from the use of Grignard reagents to transition-metal-catalyzed cross-coupling reactions.^2−4^ An alternative, atom-economical path to C–C bond formation is offered by rearrangement reactions.^5−8^ Our group has harbored a long-standing interest in such transformations,^9−12^ primarily for the way in which they enable rapid access to complex scaffolds.

As a unique case, the classical Truce–Smiles rearrangement^13−16^ is initiated by an intramolecular attack of a polar nucleophile or radical species to the *ipso*-position of an aromatic moiety bearing a good leaving group, initially leading to the formation of a Meisenheimer intermediate (Scheme 1A).^17^ While the leaving group can remain attached to the product, complete extrusion (which can serve as an entropic driving force) is also possible. We recently reported a polar Truce–Smiles rearrangement of sulfonyl acrylimides initiated by a Lewis base (Scheme 1B).^18,19^ Therein, we employed a tertiary amine to form an enolate by reversible conjugate addition, triggering a pivotal aryl migration, generating α-aryl acrylamides. The key insight was the disconnection of a weak C–SO_2_ bond in an energetically favored pathway. Aiming to further increase the complexity, we became intrigued by domino multiple bond formation using our strategy. A transition-metal-catalyzed radical approach was reported by Nevado et al.,^20−24^ triggering a Truce–Smiles rearrangement for a second bond formation to afford β-functionalized α-aryl amides (Scheme 1C). Since their pioneering work, a range of radical precursors have been explored, each with tailored reaction conditions.^25−30^ Although constituting a powerful tool to quickly access highly complex scaffolds, the nature of the radical initiation typically requires an α-branched sulfonyl acrylimide as well as *N*-aryl substitution enabling radical stabilization, otherwise leading to overreaction.^20−22,29^

**Scheme 1:**
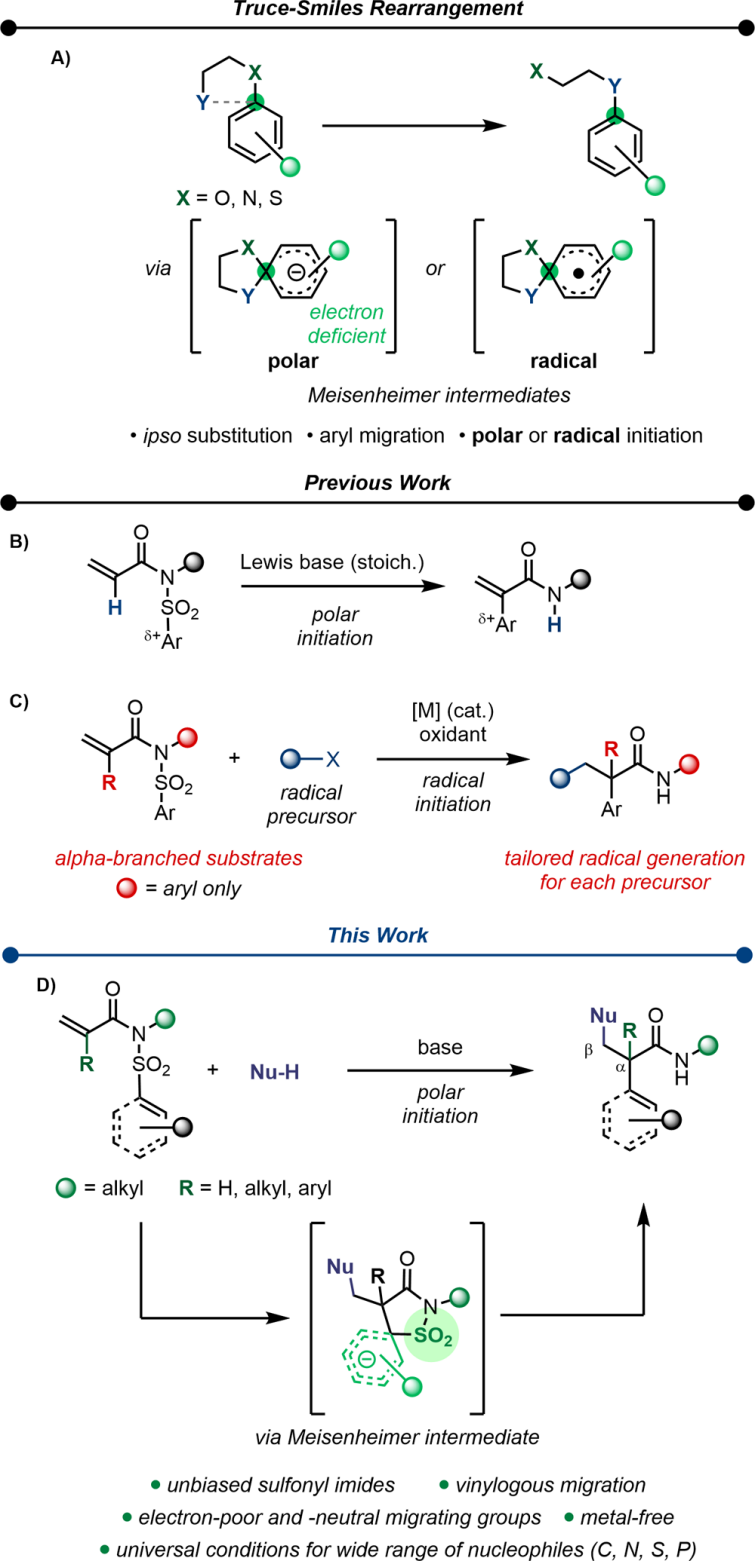
Truce–Smiles Rearrangement As a Tool for C–C Bond Formation (A) Representation of polar- and radical-initiated rearrangement. (B) Lewis base-mediated aryl migration of acryl sulfonyl imides. (C) Radical-addition-initiated domino rearrangement of methacryl sulfonyl imides. (D) This work: Brønsted base-catalyzed, conjugate-addition-triggered aryl migration for the difunctionalization of acrylamides.

To circumvent these limitations, we envisaged a universal ionic Truce–Smiles rearrangement protocol using pronucleophiles in a conjugate addition to directly generate α,β-difunctionalized amides from sulfonyl acrylimides (Scheme 1D).^31^ In contrast to the radical initiation, the ionic Truce–Smiles rearrangement with extrusion of SO_2_ is often restricted to electron-deficient aryl systems.^15,32,33^ On the other hand, ionic conjugate addition can be achieved with a wide range of nucleophiles,^34−36^ including various carbon-, sulfur-, phosphorus-, and most noteworthily, nitrogen-nucleophiles, which would enable access to a diverse range of β-amino amides. Herein, we report our key findings in this domino conjugate-addition-initiated aryl migration featuring the unprecedented ability of sulfonyl imides to transfer electron-neutral aromatic moieties by polar Truce–Smiles rearrangement.

At the outset, dimethyl 2-methylmalonate **2a** was employed as a pronucleophile in combination with sulfonyl acrylimide **1a** (Table 1). We initially chose *tert*-butyl-tetramethylguanidine **3** (BTMG) as the base, and acetonitrile as the solvent (entry 1). These conditions delivered the desired product in modest yield. Hypothesizing that ion-pair formation might play a significant role in the conjugate addition event,^37^ we investigated various polar aprotic solvents (entries 2–4; for full optimization table see the SI), revealing dimethylacetamide (DMA, entry 4) as the optimal solvent.

**Table 1:**
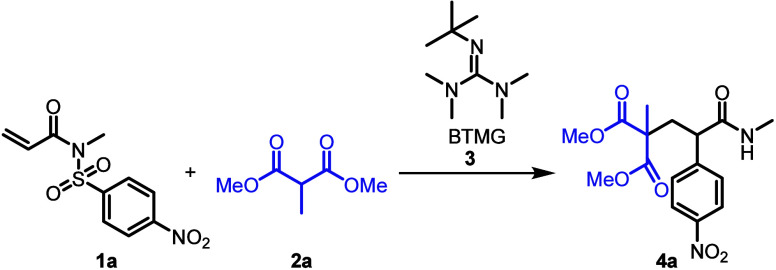
Optimization Conditions of the Conjugate-Addition-Triggered Aryl Migration

entry	eq. BTMG	solv. (0.05 M)^a^	eq. **2a**	time (h)	*T* (°C)	yield^d^
1	0.5	MeCN	1.0^b^	5	20	37%
2	0.5	acetone	1.0^b^	5	20	22%
3	0.5	DMSO	1.0^b^	5	20	31%
4	0.5	DMA	1.0^b^	5	20	63%
5	0.5	DMA	1.0^c^	5	20	66%
6	0.5	DMA	1.0^c^	16	20	73%
7	1.0	DMA	1.0^c^	16	20	80%
8	0.5	DMA	1.0^c^	67	20	83%
9	0.5	DMA	1.5^c^	67	20	89%^e^
10	0.1	DMA	1.5^c^	5	20	14%
11	0.2	DMA	1.5^c^	5	20	24%
12	0.2	DMA	1.5^c^	5	40	30%
13	0.2	DMA	1.5^c^	5	60	40%
14	0.2	DMA	1.5^c^	5	80	59%

a A 0.05 M concentration was chosen to avoid potential Rauhut–Currier-type dimerization.^18^

b Sulfonyl acrylimide **1a** was mixed with the base before addition of nucleophile **2a**.

c Nucleophile **2a** was mixed with the base, followed by addition of sulfonyl acrylimide **1a**.

d NMR yield, determined using mesitylene as an internal standard.

e Isolated yield.

Notably, under these conditions, the yield of **4a** is higher than the amount of added base. Next, we investigated the influence of reaction time (entries 6 and 8) and tested a stoichiometric amount of base (entry 7). The former revealed that prolonged reaction times are beneficial, while the latter resulted in only a modest increase in yield. Increasing the amount of nucleophile (entry 9) instead led to an improvement of the reaction outcome and allowed us to identify the optimal conditions for this transformation. Interestingly, while a further decrease in base loading (entries 10 and 11) significantly impacts the reaction outcome, elevation of the temperature partially counteracts this effect (entry 12–14). Overall, we opted to explore the substrate scope using the conditions shown in entry 9.

We first explored the scope of carbon-based nucleophiles (Scheme 2A). The reaction of diethyl 2-benzylmalonate as a more sterically demanding nucleophile for the formation of amide **4b** proceeded with only minor loss of yield. Unsubstituted malonate (c.f. **4c**) and a diketone (c.f. **4d**) are also suitable nucleophiles. Bis(phenylsulfonyl)methane smoothly reacted with **1a** to form **4e**. When the unsymmetrical nucleophile ethyl 2-(pyridin-2-yl)acetate was employed, **4f** was obtained without notable diastereoselectivity.

**Scheme 2:**
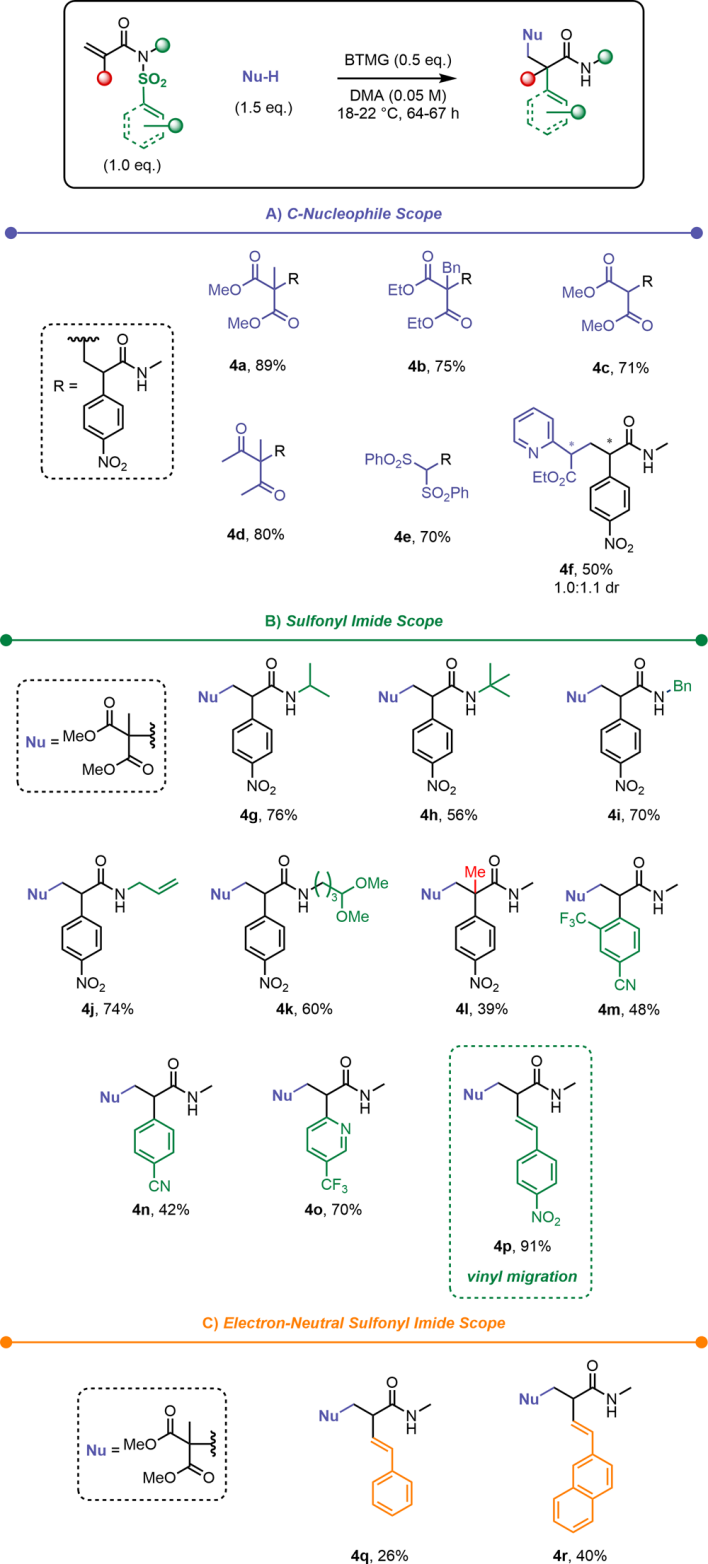
Scope of Various (A) Carbon-Based Nucleophiles, (B) Sulfonyl Acrylimides, and (C) Electron-Neutral Sulfonyl Acrylimides

As shown in Scheme 2B, an increase of steric bulk at the nitrogen atom, as showcased with *N*-isopropyl and *N*-*tert*-butyl substituents, delivered the respective amides **4g** and **4h** with reduced yields compared to the parent substrate, whereas sterically less demanding substituents (allyl and benzyl, cf. **4i** and **4j**) had no significant impact on the reaction performance. Product **4k** highlights the tolerance of this transformation for acetals, and in the case of an α-branched sulfonyl methacrylimide substrate, amide **4l** bearing an all-carbon quaternary center could be obtained. We then investigated different aryl groups, and products **4m** and **4n** were readily obtained in moderate yields. A heteroaromatic substrate bearing an electron-deficient pyridine substituent was found to give the corresponding product (**4o**) in a high yield.

Following these successful reactions, we envisaged extending this transformation to vinylogous migration,^38^ as first reported by Greaney et al.^39^ In the event, a *p-*nitrostyryl sulfonyl imide smoothly underwent the desired migration, affording **4p** in excellent yield (c.f. Scheme 2B). We hypothesized that this particularly efficient result could be the result of increased charge delocalization of the Meisenheimer intermediate, as well as reduced steric constraint by avoiding a spirocyclic structure. Intrigued by this observation, and eager to probe just how impactful these effects could be, we deployed electron-neutral sulfonyl imides (Scheme 2C). Remarkably, a simple styryl moiety led to the formation of migration product **4q** with modest yield. When the stabilization was increased within a vinylnaphthyl group (**4r**), the yield increased accordingly, further supporting our hypothesis. As an S_N_Ar reaction, the ionic Truce–Smiles rearrangement requires an electron-deficient aryl moiety, conformational enhancement or harsh conditions to work smoothly.^40−43^ Our products are the first examples of anionic Truce–Smiles rearrangements with unactivated sulfonyl imides.

Subsequently, we moved our attention to the use of heteroatom nucleophiles (Scheme 3). Diphenylphosphine oxide successfully engaged in the 1,4-addition event, resulting in the disubstituted product **5a**. The deployment of octanethiol and *para-*chloro-thiophenol resulted in thioether products **5b** and **5c**. When sodium benzenesulfinate was employed, the conjugate addition proceeded through the sulfur atom, leading to amide **5d**. In contrast to α-quaternary amide **4l**, the addition of a benzenesulfinate salt to an α-branched substrate in the absence of base resulted in product **5e** (CCDC 2313883) with an excellent 86% yield.

**Scheme 3:**
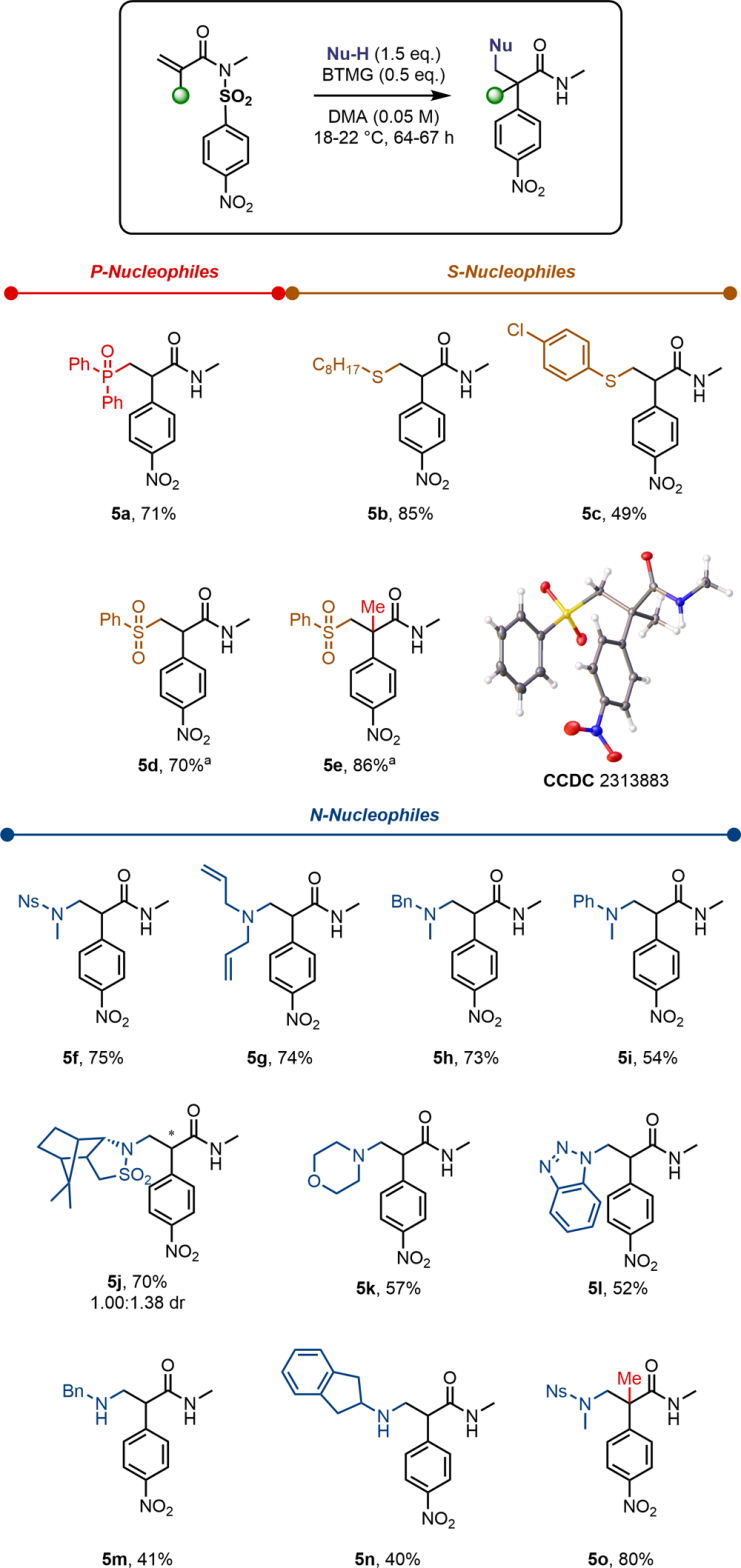
Scope of Heteroatom Nucleophiles Reaction performed using sodium benzenesulfinate without BTMG.

We were particularly interested in using nitrogen-based nucleophiles, as the products would be α-substituted β-amino amides, constituting nonproteinogenic amino acid derivatives.^44^ Despite this biological relevance, aminoarylation using free-radical processes as described in Scheme 1C has rarely been achieved and is limited in amine variety,^28^ presumably due the challenging generation and control of nitrogen-centered radicals in such a context.^45,46^ In the event of our anionic sequence, however, this was a fruitful endeavor: the use of the nucleophile *N*-methyl-nosylamide resulted in the amido amide **5f**,^47,48^ while secondary amines, specifically diallyl- (**5g**) and benzyl-methyl amine (**5h**), as well as *N-*methyl aniline (**5i**), could be employed without major loss of yield. Additionally, camphorsultam, a cyclic sulfonamide, morpholine, a cyclic secondary amine, and deprotonated benzotriazole yielded the corresponding products **5j**–**5l**. Primary amines were found to perform worse than secondary amines, in line with the expected higher p*K*_a_ (c.f. **5m** and **5n**). Comparable to our result with benzenesulfinate salt (**5e**), the reaction of *N*-methyl-nosylamide with α-methyl sulfonyl acrylimide delivered amide **5o**, bearing a quaternary carbon, in high yield.

Given the successful preparation of **5f**, we speculated that it might be possible to directly generate α,β-difunctionalized amides from the corresponding acrylic acids in a one-pot sequence. We found that the use of Mukaiyama’s reagent (**8**) in combination with BTMG and α-substituted acrylic acids **6** enables direct access to products **9** (Scheme 4). In this process, the sulfonamide notably fulfills the multivalent role of the nucleophile for amide bond formation, the nucleophile for conjugate addition, and the source of the arene moiety in the final product **5f**. Interestingly, while atropic acid afforded rearranged product **9b** in high yield, unsubstituted and α-alkyl acrylic acids favored the amide coupling products (**1a**, **1l**, and **10a**; c.f. Scheme 4).

**Scheme 4:**
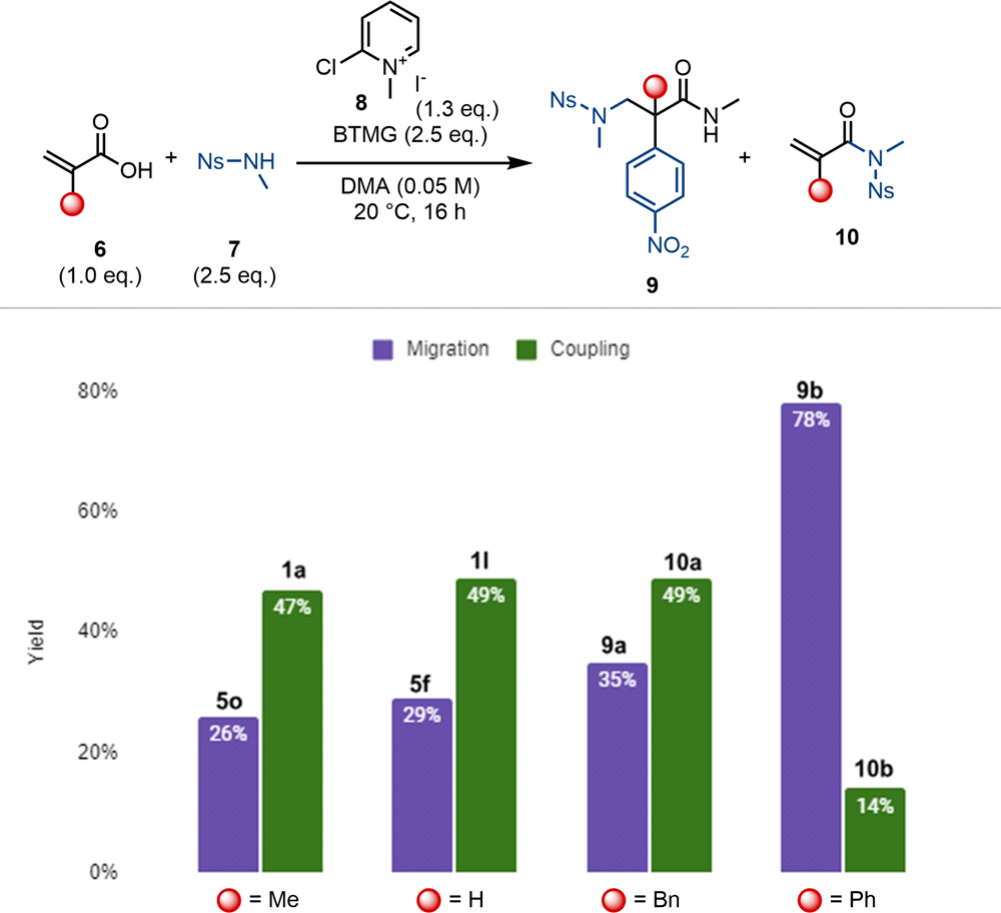
Direct Two-Step One-Pot Synthesis of the α,β-Difunctionalized Amide, Starting from the Corresponding Acrylic Acids

In summary, we have reported the synthesis of β-substituted α-aryl carboxamides by a domino conjugate addition/polar Truce–Smiles aryl migration process, mediated by a substoichiometric amount of a Brønsted base (BTMG). A wide range of carbon and heteroatom pronucleophiles can be employed, providing a high flexibility of the functionalization at the β-position. Several acryl sulfonylimides can be transformed, including underexplored vinylogous migrating groups. In contrast to classical polar Truce–Smiles rearrangements entirely reliant on electron-poor aryl moieties, electron-neutral styryl and naphthylvinyl derivatives can also be employed. Additionally, *N*-methyl-nosylamide can be directly used in a one-pot synthesis of β-amino α-aryl amides from acrylic acids.

## Supporting Information Available

Synthesis, experimental details, further characterization data of the compounds (^1^H NMR, ^13^C NMR, HRMS, IR, and X-ray), and mechanistic proposal (PDF)

## References

[ref1] Schmidt N. G.; Eger E.; Kroutil W. Building Bridges: Biocatalytic C-C-Bond Formation toward Multifunctional Products. ACS Catal. 2016, 6 (7), 4286–4311. 10.1021/acscatal.6b00758. 27398261 PMC4936090

[ref2] Luh T.-Y.; Leung M.-k.; Wong K.-T. Transition Metal-Catalyzed Activation of Aliphatic C-X Bonds in Carbon-Carbon Bond Formation. Chem. Rev. 2000, 100 (8), 3187–3204. 10.1021/cr990272o. 11749317

[ref3] Ackerman-Biegasiewicz L. K. G.; Kariofillis S. K.; Weix D. J. Multimetallic-Catalyzed C-C Bond-Forming Reactions: From Serendipity to Strategy. J. Am. Chem. Soc. 2023, 145 (12), 6596–6614. 10.1021/jacs.2c08615. 36913663 PMC10163949

[ref4] Milligan J. A.; Phelan J. P.; Badir S. O.; Molander G. A. Alkyl Carbon-Carbon Bond Formation by Nickel/Photoredox Cross-Coupling. Angew. Chem. Int. Ed. 2019, 58 (19), 6152–6163. 10.1002/anie.201809431. PMC6551614 30291664

[ref5] Majumdar K. C.; Nandi R. K. The Claisen Rearrangement in the Syntheses of Bioactive Natural Products. Tetrahedron 2013, 69 (34), 6921–6957. 10.1016/j.tet.2013.06.003.

[ref6] Ghosh A. K.; Brindisi M.; Sarkar A. The Curtius Rearrangement: Applications in Modern Drug Discovery and Medicinal Chemistry. ChemMedChem. 2018, 13 (22), 2351–2373. 10.1002/cmdc.201800518. 30187672 PMC6604631

[ref7] Chen L.; Li G.; Zu L. Natural Product Total Synthesis Using Rearrangement Reactions. Org. Chem. Front. 2022, 9 (19), 5383–5394. 10.1039/D2QO01040B.

[ref8] Liu Y.; Liu X.; Feng X. Recent Advances in Metal-Catalysed Asymmetric Sigmatropic Rearrangements. Chem. Sci. 2022, 13 (42), 12290–12308. 10.1039/D2SC03806D. 36382273 PMC9629009

[ref9] Kaiser D.; Bauer A.; Lemmerer M.; Maulide N. Amide Activation: An Emerging Tool for Chemoselective Synthesis. Chem. Soc. Rev. 2018, 47 (21), 7899–7925. 10.1039/C8CS00335A. 30152510

[ref10] Kaldre D.; Klose I.; Maulide N. Stereodivergent Synthesis of 1,4-Dicarbonyls by Traceless Charge-Accelerated Sulfonium Rearrangement. Science 2018, 361 (6403), 664–667. 10.1126/science.aat5883. 30115803

[ref11] Kaiser D.; Klose I.; Oost R.; Neuhaus J.; Maulide N. Bond-Forming and -Breaking Reactions at Sulfur(IV): Sulfoxides, Sulfonium Salts, Sulfur Ylides, and Sulfinate Salts. Chem. Rev. 2019, 119 (14), 8701–8780. 10.1021/acs.chemrev.9b00111. 31243998 PMC6661881

[ref12] Feng M.; Mosiagin I.; Kaiser D.; Maryasin B.; Maulide N. Deployment of Sulfinimines in Charge-Accelerated Sulfonium Rearrangement Enables a Surrogate Asymmetric Mannich Reaction. J. Am. Chem. Soc. 2022, 144 (29), 13044–13049. 10.1021/jacs.2c05368. 35839521 PMC9374180

[ref13] Holden C. M.; Greaney M. F. Modern Aspects of the Smiles Rearrangement. Chemistry 2017, 23 (38), 8992–9008. 10.1002/chem.201700353. 28401655

[ref14] Henderson A. R. P.; Kosowan J. R.; Wood T. E. The Truce–Smiles Rearrangement and Related Reactions: A Review. Can. J. Chem. 2017, 95 (5), 483–504. 10.1139/cjc-2016-0594.

[ref15] Whalley D. M.; Greaney M. F. Recent Advances in the Smiles Rearrangement: New Opportunities for Arylation. Synthesis 2022, 54, 1908–1918. 10.1055/a-1710-6289.

[ref16] G.-Simonian N.; Guerinot A.; Cossy J. SO_2_-Extrusive 1,4-(Het)Aryl Migration: Synthesis of α-Aryl Amides and Related Reactions. Synthesis 2023, 55 (11), 1616–1641. 10.1055/s-0040-1720035.

[ref17] Lennox A. J. J. Meisenheimer Complexes in S_N_Ar Reactions: Intermediates or Transition States?. Angew. Chem. Int. Ed. 2018, 57 (45), 14686–14688. 10.1002/anie.201809606. 30320484

[ref18] Lemmerer M.; Zhang H.; Fernandes A. J.; Fischer T.; Mießkes M.; Xiao Y.; Maulide N. Synthesis of α-Aryl Acrylamides via Lewis-Base-Mediated Aryl/Hydrogen Exchange. Angew. Chem. Int. Ed. 2022, 61 (40), e202207475 10.1002/anie.202207475. PMC9804524 35881564

[ref19] Lemmerer M.; Maulide N. Lewis Base-Assisted Arylation of Unsaturated Carbonyls. Chemistry 2023, 29 (66), e202302490 10.1002/chem.202302490. 37647146 PMC10947297

[ref20] Kong W.; Casimiro M.; Fuentes N.; Merino E.; Nevado C. Metal-Free Aryltrifluoromethylation of Activated Alkenes. Angew. Chem. Int. Ed. 2013, 52 (49), 13086–13090. 10.1002/anie.201307377. 24174281

[ref21] Kong W.; Merino E.; Nevado C. Arylphosphonylation and Arylazidation of Activated Alkenes. Angew. Chem. Int. Ed. 2014, 53 (20), 5078–5082. 10.1002/anie.201311241. 24692217

[ref22] Fuentes N.; Kong W.; Fernández-Sánchez L.; Merino E.; Nevado C. Cyclization Cascades via N-Amidyl Radicals toward Highly Functionalized Heterocyclic Scaffolds. J. Am. Chem. Soc. 2015, 137 (2), 964–973. 10.1021/ja5115858. 25561161

[ref23] Kong W.; Fuentes N.; García-Domínguez A.; Merino E.; Nevado C. Stereoselective Synthesis of Highly Functionalized Indanes and Dibenzocycloheptadienes through Complex Radical Cascade Reactions. Angew. Chem. Int. Ed. 2015, 54 (8), 2487–2491. 10.1002/anie.201409659. 25597296

[ref24] Hervieu C.; Kirillova M. S.; Suárez T.; Müller M.; Merino E.; Nevado C. Asymmetric, Visible Light-Mediated Radical Sulfinyl-Smiles Rearrangement to Access All-Carbon Quaternary Stereocentres. Nat. Chem. 2021, 13 (4), 327–334. 10.1038/s41557-021-00668-4. 33833448

[ref25] Fan J.-H.; Yang J.; Song R.-J.; Li J.-H. Palladium-Catalyzed Oxidative Heck-Type Alkylation/Aryl Migration/Desulfonylation between Alkenes with α-Carbonyl Alkyl Bromides. Org. Lett. 2015, 17 (4), 836–839. 10.1021/ol503660a. 25654662

[ref26] Ni Z.; Huang X.; Pan Y. Metal-Free Mediated Meerwein-Type Reaction: A Radical Cascade Arylation/Aryl Migration/Desulfonylation of Conjugated Alkenes. Org. Lett. 2016, 18 (11), 2612–2615. 10.1021/acs.orglett.6b01041. 27219900

[ref27] Liu K.; Sui L.-C.; Jin Q.; Li D.-Y.; Liu P.-N. CuBr-Mediated Radical Cascade Difluoroacetamidation of Acrylamides Using α,α-Difluoro-α-(TMS)-Acetamides. Org. Chem. Front. 2017, 4 (8), 1606–1610. 10.1039/C7QO00209B.

[ref28] Wang J.-L.; Liu M.-L.; Zou J.-Y.; Sun W.-H.; Liu X.-Y. Copper-Catalyzed Aminoarylation of Alkenes via Aminyl Radical Addition and Aryl Migration. Org. Lett. 2022, 24 (1), 309–313. 10.1021/acs.orglett.1c03973. 34931822

[ref29] Zhao Z.-W.; Ran Y.-S.; Hou Y.-J.; Chen X.; Ding X.-L.; Zhang C.; Li Y.-M. Free Radical Cascade Carbochloromethylations of Activated Alkenes. J. Org. Chem. 2022, 87 (6), 4183–4194. 10.1021/acs.joc.1c03024. 35234480

[ref30] Wu X.; Zhang X.; Ji X.; Deng G.-J.; Huang H. Visible-Light-Induced Cascade Arylazidation of Activated Alkenes with Trimethylsilyl Azide. Org. Lett. 2023, 25 (27), 5162–5167. 10.1021/acs.orglett.3c01933. 37382596

[ref31] Guoren Y.; Zheng Z. The Reaction of 1-Aryl-2-(4-Nitrophenylsulfonyl)Ethanones with α,β-Unsaturated Esters. Synth. Commun. 1997, 27 (8), 1455–1463. 10.1080/00397919708006077.

[ref32] Swaby C.; Taylor A.; Greaney M. F. An NHC-Catalyzed Desulfonylative Smiles Rearrangement of Pyrrole and Indole Carboxaldehydes. J. Org. Chem. 2023, 88 (17), 12821–12825. 10.1021/acs.joc.3c01089. 37589318 PMC10476196

[ref33] Sephton T.; Large J. M.; Butterworth S.; Greaney M. F. Synthesis of Functionalized Pyrrolidinone Scaffolds via Smiles-Truce Cascade. Org. Lett. 2023, 25 (36), 6736–6740. 10.1021/acs.orglett.3c02559. 37668613 PMC10510726

[ref34] Hayama N.; Kuramoto R.; Földes T.; Nishibayashi K.; Kobayashi Y.; Pápai I.; Takemoto Y. Mechanistic Insight into Asymmetric Hetero-Michael Addition of α,β-Unsaturated Carboxylic Acids Catalyzed by Multifunctional Thioureas. J. Am. Chem. Soc. 2018, 140 (38), 12216–12225. 10.1021/jacs.8b07511. 30215516

[ref35] Das T.; Mohapatra S.; Mishra N. P.; Nayak S.; Raiguru B. P. Recent Advances in Organocatalytic Asymmetric Michael Addition Reactions to α,β-unsaturated Nitroolefins. ChemistrySelect 2021, 6 (15), 3745–3781. 10.1002/slct.202100679.

[ref36] Das T.; Mohapatra S.; Pradhan A. K.; Nayak S. Recent Advances of Michael/Hetero-Michael Addition Reaction in the Synthesis of 3-Nitro-2*H*-chromene Derivatives. ChemistrySelect 2023, 8 (14), e202300477 10.1002/slct.202300477.

[ref37] Fedotova A.; Kondrashov E.; Legros J.; Maddaluno J.; Rulev A. Y. Solvent Effects in the Aza-Michael Addition of Anilines. C. R. Chim. 2018, 21 (7), 639–643. 10.1016/j.crci.2018.03.006.

[ref38] Mas-Roselló J.; Hachisu S.; Clayden J. Geometry-Retentive C-Alkenylation of Lithiated α-Aminonitriles: Quaternary α-Alkenyl Amino Acids and Hydantoins. Angew. Chem. Int. Ed. 2017, 56 (36), 10750–10754. 10.1002/anie.201704908. 28649795

[ref39] Johnson S.; Kovács E.; Greaney M. F. Arylation and Alkenylation of Activated Alkyl Halides Using Sulfonamides. Chem. Commun. 2020, 56 (21), 3222–3224. 10.1039/D0CC00220H. 32073052

[ref40] Truce W. E.; Ray W. J. Jr; Norman O. L.; Eickemeyer D. B. Rearrangements of Aryl Sulfones. I. the Metalation and Rearrangement of Mesityl Phenyl Sulfone^1^. J. Am. Chem. Soc. 1958, 80 (14), 3625–3629. 10.1021/ja01547a038.

[ref41] Clayden J.; Dufour J.; Grainger D. M.; Helliwell M. Substituted Diarylmethylamines by Stereospecific Intramolecular Electrophilic Arylation of Lithiated Ureas. J. Am. Chem. Soc. 2007, 129 (24), 7488–7489. 10.1021/ja071523a. 17521189

[ref42] Costil R.; Dale H. J. A.; Fey N.; Whitcombe G.; Matlock J. V.; Clayden J. Heavily Substituted Atropisomeric Diarylamines by Unactivated Smiles Rearrangement of N-Aryl Anthranilamides. Angew. Chem. Int. Ed. 2017, 56 (41), 12533–12537. 10.1002/anie.201706341. 28817222

[ref43] Abrams R.; Jesani M. H.; Browning A.; Clayden J. Triarylmethanes and Their Medium-Ring Analogues by Unactivated Truce-Smiles Rearrangement of Benzanilides. Angew. Chem. Int. Ed. 2021, 60 (20), 11272–11277. 10.1002/anie.202102192. PMC8252078 33830592

[ref44] Paulsen M. H.; Engqvist M.; Ausbacher D.; Strøm M. B.; Bayer A. Efficient and Scalable Synthesis of α,α-Disubstituted β-Amino Amides. Org. Biomol. Chem. 2016, 14 (31), 7570–7578. 10.1039/C6OB01219A. 27439743

[ref45] Pratley C.; Fenner S.; Murphy J. A. Nitrogen-Centered Radicals in Functionalization of sp^2^ Systems: Generation, Reactivity, and Applications in Synthesis. Chem. Rev. 2022, 122 (9), 8181–8260. 10.1021/acs.chemrev.1c00831. 35285636

[ref46] Kwon K.; Simons R. T.; Nandakumar M.; Roizen J. L. Strategies to Generate Nitrogen-Centered Radicals That May Rely on Photoredox Catalysis: Development in Reaction Methodology and Applications in Organic Synthesis. Chem. Rev. 2022, 122 (2), 2353–2428. 10.1021/acs.chemrev.1c00444. 34623809 PMC8792374

[ref47] Coulibali S.; Godou T.; Canesi S. Use of the Nosyl Group as a Functional Protecting Group in Applications of a Michael/Smiles Tandem Process. Org. Lett. 2016, 18 (17), 4348–4351. 10.1021/acs.orglett.6b02105. 27513700

[ref48] Liu J.; Ba D.; Lv W.; Chen Y.; Zhao Z.; Cheng G. Base-promoted Michael Addition/Smiles Rearrangement/N-Arylation Cascade: One-step Synthesis of 1,2,3-trisubstituted 4-quinolones from Ynones and Sulfonamides. Adv. Synth. Catal. 2020, 362 (1), 213–223. 10.1002/adsc.201900960.

