# Supplementary material for: Domino Conjugate Addition-1,4-Aryl Migration for the Synthesis of α,β-Difunctionalized Amides
Source: JACS Au. 2024 Jun 17;4(7):2456–61. doi: 10.1021/jacsau.4c00378 (PMC11267538; doi:10.1021/jacsau.4c00378)
Supplement: Supplementary file 1 — au4c00378_si_001.pdf [file au4c00378_si_001.pdf]

Supporting Information

Domino Conjugate Addition-1,4-Aryl Migration for the Synthesis of α,β-Difunctionalised Amides

Haoqi Zhang,[a,b,c] Yi Xiao,[a,b,d] Miran Lemmerer,[a,b] Tommaso Bortolato,[a] and Nuno Maulide*[a,c,d]

[a]Institute of Organic Chemistry, University of Vienna, Währinger Straße 38, 1090 Vienna, Austria, E-mail: nuno.maulide@univie.ac.at

[b]Vienna Doctoral School in Chemistry, University of Vienna, Währinger Straße 42, 1090 Vienna, Austria.

[c]Christian-Doppler Laboratory for Entropy-Oriented Drug Design, Josef-Holaubek-Platz 2, 1090 Vienna, Austria

[d]CeMM Research Center for Molecular Medicine of the Austrian Academy of Sciences, Lazarettgasse 14, AKH BT 25.3, 1090 Vienna, Austria

Table of Contents

1. General Information 6

2. Optimisation 7

3. Starting Material Synthesis 8

Previously reported sulfonyl acrylimides 8

Synthesis of sulfonamides 9

1oa: *N*-methyl-5-(trifluoromethyl)pyridine-2-sulfonamide 9

1pa: (*E*)-*N*-methyl-2-(4-nitrophenyl)ethene-1-sulfonamide 10

1qa: (*E*)-*N*-methyl-2-phenylethene-1-sulfonamide 11

1ra: (E)-*N*-methyl-2-(naphthalen-2-yl)ethene-1-sulfonamide 12

Synthesis of sulfonyl acrylimides 13

1l: *N*-methyl-*N*-((4-nitrophenyl)sulfonyl)methacrylamide 13

1o: *N*-methyl-*N*-((5-(trifluoromethyl)pyridin-2-yl)sulfonyl)acrylamide 14

1p: (*E*)-*N*-methyl-*N*-((4-nitrostyryl)sulfonyl)acrylamide 14

1q: (*E*)-*N*-methyl-*N*-(styrylsulfonyl)acrylamide 15

1r: (*E*)-*N*-methyl-*N*-((2-(naphthalen-2-yl)vinyl)sulfonyl)acrylamide 16

4. Synthesis of α,β-substituted amides 17

Unsuccessful substrates 18

S1: *N*-methyl-*N*-phenylacrylamide 19

S2: *N*-methyl-3-((*N*-methyl-4-nitrophenyl)sulfonamido)-*N*-phenylpropanamide 19

4a: Dimethyl 2-methyl-2-(3-(methylamino)-2-(4-nitrophenyl)-3-oxopropyl)malonate 20

4b: Diethyl 2-benzyl-2-(3-(methylamino)-2-(4-nitrophenyl)-3-oxopropyl)malonate 21

4c: Dimethyl 2-(3-(methylamino)-2-(4-nitrophenyl)-3-oxopropyl)malonate 21

4d: 4-acetyl-*N*,4-dimethyl-2-(4-nitrophenyl)-5-oxohexanamide 22

4e: N-methyl-2-(4-nitrophenyl)-4,4-bis(phenylsulfonyl)butanamide 22

4f: Ethyl 5-(methylamino)-4-(4-nitrophenyl)-5-oxo-2-(pyridin-2-yl)pentanoate 23

4g: Dimethyl 2-(3-(isopropylamino)-2-(4-nitrophenyl)-3-oxopropyl)-2-methylmalonate 24

4h: Dimethyl 2-(3-(*tert*-butylamino)-2-(4-nitrophenyl)-3-oxopropyl)-2-methylmalonate 24

4i: Dimethyl 2-(3-(benzylamino)-2-(4-nitrophenyl)-3-oxopropyl)-2-methylmalonate 25

4j: Dimethyl 2-(3-(allylamino)-2-(4-nitrophenyl)-3-oxopropyl)-2-methylmalonate 25

4k: Dimethyl 2-(3-((4,4-dimethoxybutyl)amino)-2-(4-nitrophenyl)-3-oxopropyl)-2-methylmalonate 26

4l: Dimethyl 2-methyl-2-(2-methyl-3-(methylamino)-2-(4-nitrophenyl)-3-oxopropyl)malonate 26

4m: Dimethyl 2-(2-(4-cyano-2-(trifluoromethyl)phenyl)-3-(methylamino)-3-oxopropyl)-2-methylmalonate 27

4n: Dimethyl 2-(2-(4-cyanophenyl)-3-(methylamino)-3-oxopropyl)-2-methylmalonate 27

4o: Dimethyl 2-methyl-2-(3-(methylamino)-3-oxo-2-(5-(trifluoromethyl)pyridin-2-yl)propyl)malonate 28

4p: Dimethyl (*E*)-2-methyl-2-(2-(methylcarbamoyl)-4-(4-nitrophenyl)but-3-en-1-yl)malonate (XX). 29

4q: Dimethyl (*E*)-2-methyl-2-(2-(methylcarbamoyl)-4-phenylbut-3-en-1-yl)malonate 29

4r: Dimethyl (*E*)-2-methyl-2-(2-(methylcarbamoyl)-4-(naphthalen-2-yl)but-3-en-1-yl)malonate 30

5a: 3-(diphenylphosphoryl)-*N*-methyl-2-(4-nitrophenyl)propanamide 30

5b: *N*-methyl-2-(4-nitrophenyl)-3-(octylthio)propanamide 31

5c: 3-((4-chlorophenyl)thio)-*N*-methyl-2-(4-nitrophenyl)propanamide 31

5d: *N*-methyl-2-(4-nitrophenyl)-3-(phenylsulfonyl)propanamide 32

5e: *N*,2-dimethyl-2-(4-nitrophenyl)-3-(phenylsulfonyl)propanamide 32

5f: *N*-methyl-3-((*N*-methyl-4-nitrophenyl)sulfonamido)-2-(4-nitrophenyl)propanamide 33

5g: 3-(diallylamino)-*N*-methyl-2-(4-nitrophenyl)propanamide 34

5h: 3-(benzyl(methyl)amino)-*N*-methyl-2-(4-nitrophenyl)propanamide 34

5i: *N*-methyl-3-(methyl(phenyl)amino)-2-(4-nitrophenyl)propanamide 35

5j: 3-((3a*R*,6*S*,7a*S*)-8,8-dimethyl-2,2-dioxidotetrahydro-3*H*-3a,6-methanobenzo[*c*]isothiazol-1(4*H*)-yl)-*N*-methyl-2-(4-nitrophenyl)propanamide 35

5k: *N*-methyl-3-morpholino-2-(4-nitrophenyl)propenamide 36

5l: 3-(1*H*-benzo[*d*][1,2,3]triazol-1-yl)-*N*-methyl-2-(4-nitrophenyl)propanamide 37

5m: 3-(benzylamino)-*N*-methyl-2-(4-nitrophenyl)propanamide 37

5n: 3-((2,3-dihydro-1*H*-inden-2-yl)amino)-*N*-methyl-2-(4-nitrophenyl)propenamide 38

5o: *N*,2-dimethyl-3-((*N*-methyl-4-nitrophenyl)sulfonamido)-2-(4-nitrophenyl)propanamide 38

5. One-pot amide coupling/Smiles rearrangement 39

Optimisation 39

9b: *N*-methyl-3-((N-methyl-4-nitrophenyl)sulfonamido)-2-(4-nitrophenyl)-2-phenylpropanamide . 40

9b: *N*-methyl-N-((4-nitrophenyl)sulfonyl)-2-phenylacrylamide 40

9a: 2-benzyl-*N*-methyl-3-((N-methyl-4-nitrophenyl)sulfonamido)-2-(4-nitrophenyl)propanamide .. 41

10a: 2-benzyl-*N*-methyl-*N*-((4-nitrophenyl)sulfonyl)acrylamide 41

6. Mechanistical Proposal 42

7. X-ray Analysis 43

5e: *N*,2-dimethyl-2-(4-nitrophenyl)-3-(phenylsulfonyl)propanamide 43

8. NMR Spectra 50

1oa: *N*-methyl-5-(trifluoromethyl)pyridine-2-sulfonamide 50

1pa: (*E*)-*N*-methyl-2-(4-nitrophenyl)ethene-1-sulfonamide 52

1qa: (*E*)-*N*-methyl-2-phenylethene-1-sulfonamide 53

1ra: (*E*)-*N*-methyl-2-(naphthalen-2-yl)ethene-1-sulfonamide 54

1l: *N*-methyl-*N*-((4-nitrophenyl)sulfonyl)methacrylamide 55

1o: *N*-methyl-*N*-((5-(trifluoromethyl)pyridin-2-yl)sulfonyl)acrylamide 56

1p: (*E*)-*N*-methyl-*N*-((4-nitrostyryl)sulfonyl)acrylamide 58

1q: (*E*)-*N*-methyl-*N*-(styrylsulfonyl)acrylamide 59

1r: (*E*)-*N*-methyl-*N*-((2-(naphthalen-2-yl)vinyl)sulfonyl)acrylamide 60

S1: N-methyl-N-phenylacrylamide 61

S2: *N*-methyl-3-((*N*-methyl-4-nitrophenyl)sulfonamido)-*N*-phenylpropanamide 62

4a: Dimethyl 2-methyl-2-(3-(methylamino)-2-(4-nitrophenyl)-3-oxopropyl)malonate 63

4b: Diethyl 2-benzyl-2-(3-(methylamino)-2-(4-nitrophenyl)-3-oxopropyl)malonate 64

4c: Dimethyl 2-(3-(methylamino)-2-(4-nitrophenyl)-3-oxopropyl)malonate 65

4d: 4-acetyl-*N*,4-dimethyl-2-(4-nitrophenyl)-5-oxohexanamide 66

4e: *N*-methyl-2-(4-nitrophenyl)-4,4-bis(phenylsulfonyl)butanamide 67

4f: Ethyl 5-(methylamino)-4-(4-nitrophenyl)-5-oxo-2-(pyridin-2-yl)pentanoate 68

4g: Dimethyl 2-(3-(isopropylamino)-2-(4-nitrophenyl)-3-oxopropyl)-2-methylmalonate 69

4h: Dimethyl 2-(3-(*tert*-butylamino)-2-(4-nitrophenyl)-3-oxopropyl)-2-methylmalonate 70

4i: Dimethyl 2-(3-(benzylamino)-2-(4-nitrophenyl)-3-oxopropyl)-2-methylmalonate 71

4j: Dimethyl 2-(3-(allylamino)-2-(4-nitrophenyl)-3-oxopropyl)-2-methylmalonate 72

4k: Dimethyl 2-(3-((4,4-dimethoxybutyl)amino)-2-(4-nitrophenyl)-3-oxopropyl)-2-methylmalonate 73

4l: Dimethyl 2-methyl-2-(2-methyl-3-(methylamino)-2-(4-nitrophenyl)-3-oxopropyl)malonate 74

4m: Dimethyl 2-(2-(4-cyano-2-(trifluoromethyl)phenyl)-3-(methylamino)-3-oxopropyl)-2-methylmalonate 75

4n: Dimethyl 2-(2-(4-cyanophenyl)-3-(methylamino)-3-oxopropyl)-2-methylmalonate 77

4o: Dimethyl 2-methyl-2-(3-(methylamino)-3-oxo-2-(5-(trifluoromethyl)pyridin-2-yl)propyl)malonate 78

4p: Dimethyl (*E*)-2-methyl-2-(2-(methylcarbamoyl)-4-(4-nitrophenyl)but-3-en-1-yl)malonate 80

4q: Dimethyl (*E*)-2-methyl-2-(2-(methylcarbamoyl)-4-phenylbut-3-en-1-yl)malonate 81

4r: Dimethyl (*E*)-2-methyl-2-(2-(methylcarbamoyl)-4-(naphthalen-2-yl)but-3-en-1-yl)malonate 82

5a: 3-(diphenylphosphoryl)-*N*-methyl-2-(4-nitrophenyl)propanamide 83

5b: *N*-methyl-2-(4-nitrophenyl)-3-(octylthio)propanamide 85

5c: 3-((4-chlorophenyl)thio)-*N*-methyl-2-(4-nitrophenyl)propanamide 86

5d: *N*-methyl-2-(4-nitrophenyl)-3-(phenylsulfonyl)propanamide 87

5e: *N*,2-dimethyl-2-(4-nitrophenyl)-3-(phenylsulfonyl)propanamide 88

5f: *N*-methyl-3-((*N*-methyl-4-nitrophenyl)sulfonamido)-2-(4-nitrophenyl)propanamide 89

5g: 3-(diallylamino)-*N*-methyl-2-(4-nitrophenyl)propanamide 90

5h: 3-(benzyl(methyl)amino)-*N*-methyl-2-(4-nitrophenyl)propanamide 91

5i: *N*-methyl-3-(methyl(phenyl)amino)-2-(4-nitrophenyl)propanamide 92

5j-A: 3-((3aR,6S,7aS)-8,8-dimethyl-2,2-dioxidotetrahydro-3H-3a,6-methanobenzo[c]isothiazol-1(4H)-yl)-*N*-methyl-2-(4-nitrophenyl)propanamide 93

5j-B: 3-((3aR,6S,7aS)-8,8-dimethyl-2,2-dioxidotetrahydro-3H-3a,6-methanobenzo[c]isothiazol-1(4H)-yl)-*N*-methyl-2-(4-nitrophenyl)propanamide 94

5k: *N*-methyl-3-morpholino-2-(4-nitrophenyl)propenamide 95

5l: 3-(1H-benzo[d][1,2,3]triazol-1-yl)-*N*-methyl-2-(4-nitrophenyl)propanamide 96

5m: 3-(benzylamino)-*N*-methyl-2-(4-nitrophenyl)propenamide 97

5n: 3-((2,3-dihydro-1H-inden-2-yl)amino)-*N*-methyl-2-(4-nitrophenyl)propenamide 98

5o: *N*,2-dimethyl-3-((*N*-methyl-4-nitrophenyl)sulfonamido)-2-(4-nitrophenyl)propanamide 99

9b: *N*-methyl-3-((*N*-methyl-4-nitrophenyl)sulfonamido)-2-(4-nitrophenyl)-2-phenylpropanamide 100

10b: *N*-methyl-N-((4-nitrophenyl)sulfonyl)-2-phenylacrylamide 101

9a: 2-benzyl-*N*-methyl-3-((*N*-methyl-4-nitrophenyl)sulfonamido)-2-(4-nitrophenyl)propanamide 102

10a: 2-benzyl-*N*-methyl-*N*-((4-nitrophenyl)sulfonyl)acrylamide 103

8. References 104

1. General Information

Unless otherwise stated, all glassware was flame-dried before use and all reactions were performed under an atmosphere of argon with anhydrous solvents. All reagents were used as received from commercial suppliers, unless otherwise stated. Reaction progress was monitored by thin layer chromatography (TLC) performed on aluminium plates coated with silica gel F254 with 0.2 mm thickness. Chromatograms were visualised by fluorescence quenching with UV light at 254 nm or by staining using potassium permanganate. Flash column chromatography was performed using silica gel 60 (230-400 mesh, Merck and co.). DMA mixture for column chromatography was made of DCM, MeOH, concentrated ammonia (90:10:1). Neat infra-red spectra were recorded using a Perkin-Elmer Spectrum 100 FT-IR spectrometer. Wavenumbers (ν_{max}) are reported in cm^{-1}. Mass spectra were obtained using a Finnigan MAT 8200 or (70 eV) or an Agilent 5973 (70 eV) spectrometer, using electrospray ionization (ESI). All ^{1}H NMR and ^{13}C NMR spectra were recorded using a Bruker AV-400, AV-600 or AV-700 spectrometer at 300 K. Chemical shifts are given in parts per million (ppm, δ) and coupling constants (*J*) are quoted in Hz, referenced to the solvent peak of $CDCl_3$, defined at δ = 7.26 ppm (^{1}H NMR) and δ = 77.16 (^{13}C NMR). Hz^{1}H NMR splitting patterns are designated as singlet (s), doublet (d), triplet (t), quartet (q), and pentet (p). Splitting patterns that could not be interpreted or easily visualized are designated as multiplet (m) or broad (br), additionally, peaks differing in appearance from their expected splitting patterns are designated as apparent (app). Selected ^{13}C NMR spectra were partially recorded using the attached proton test (APT) to facilitate confirmation and assignment of the structure. Single-crystal X-ray diffraction data were collected with a Stadivari Diffractometer (STOE & Cie GmbH, Germany) equipped with an EIGER2 R500 detector (Dectris Ltd, Switzerland). Data were processed and scaled with the STOE software suite X-Area (STOE & Cie GmbH). Structures were solved with SHELXT,[1] and refined with SHELXL[2] or Olex2.[3] Model building was done with Olex2 or ShelXle. The structure was validated with CHECKCIF (https://checkcif.iucr.org/). See the respective CIF file for exact versions and more details.

2. Optimisation

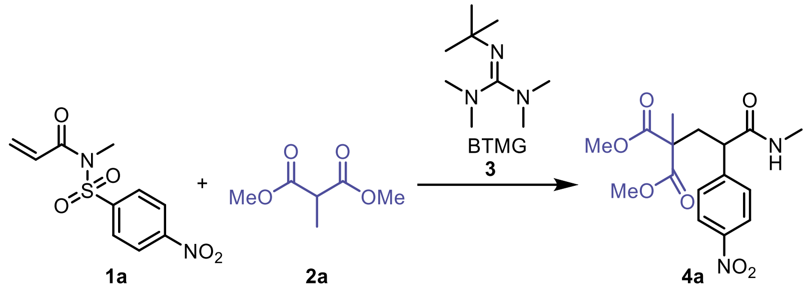

*Table 1 Detailed optimisation table for product **4a***

Entry	Base	eq. Base	Solvent	c [M]	Eq. **2a**	t [h]	T [°C]	Additive	Yield[c]
1	BTMG	0.5	MeCN	0.05	1.0[a]	5	20	-	37%
2	BTMG	0.5	**acetone**	0.05	1.0[a]	5	20	-	22%
3	BTMG	0.5	**DMF**	0.05	1.0[a]	5	20	-	20%
4	BTMG	0.5	**DMSO**	0.05	1.0[a]	5	20	-	31%
5	BTMG	0.5	**DMA**	0.05	1.0[a]	5	20	-	63%
6	BTMG	0.5	DMA	0.05	**1.0[b]**	5	20	-	66%
7	BTMG	0.5	DMA	0.05	1.0[b]	**16**	20	-	73%
8	BTMG	0.5	DMA	**0.2**	1.0[b]	16	20	-	67%
9	BTMG	0.5	**DMC**	0.05	1.0[b]	16	20	-	73%
10	BTMG	**1**	DMA	0.05	1.0[b]	16	20	-	80%
11	BTMG	0.5	DMA	0.05	1.0[b]	**67**	20	-	83%
12	BTMG	0.5	DMA	0.05	**1.5[b]**	67	20	-	89%[d]
13	BTMG	**0.4**	DMA	0.05	1.5[b]	16	20	-	71%
14	BTMG	**0.3**	DMA	0.05	1.5[b]	16	20	-	49%
15	BTMG	**0.2**	DMA	0.05	1.5[b]	16	20	-	24%
16	BTMG	**0.1**	DMA	0.05	1.5[b]	16	20	-	14%
17	BTMG	**0.2**	DMA	0.05	1.5[b]	16	20	-	24%
18	BTMG	0.2	DMA	0.05	1.5[b]	**67**	20	-	24%
19	BTMG	0.2	DMA	0.05	1.5[b]	67	20	**1.0 eq. HFIP**	21%
20	BTMG	0.2	DMA	0.05	1.5[b]	67	20	**1.0 eq. H_2O**	24%
21	**TMG**	0.2	DMA	0.05	1.5[b]	67	20	-	18%
22	**PS**	0.2	DMA	0.05	1.5[b]	67	20	-	0%
23	**DBN**	0.2	DMA	0.05	1.5[b]	67	20	-	7%
24	**MTBD**	0.2	DMA	0.05	1.5[b]	67	20	-	12%
25	BTMG	0.2	DMA	0.05	1.5[b]	16	**40**	-	30%
26	BTMG	0.2	DMA	0.05	1.5[b]	16	**60**	-	40%
27	BTMG	0.2	DMA	0.05	1.5[b]	16	**80**	-	59%

[a]Sulfonyl acrylamide **1a** was mixed with base before addition of nucleophile **2a**. [b] Nucleophile **2a** was mixed with the base, followed by addition of sulfonyl acrylamide **1a**. [c] NMR-yield, determined using mesitylene as an internal standard. [d] Isolated yield. Eq.: Equivalence; c: reaction concentration; t: reaction time; T: reaction temperature.

3. Starting Material Synthesis

Previously reported sulfonyl acrylimides

The following sulfonyl acrylimides were synthesised and characterised according to the procedures reported in our previous work.[4]

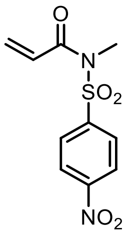

1a

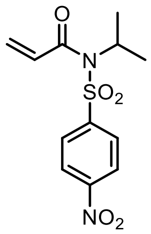

1g

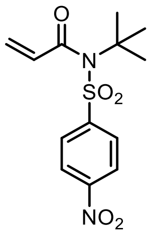

1h

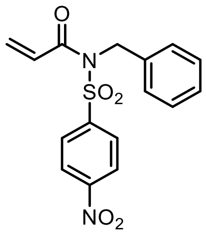

1i

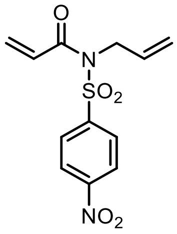

1j

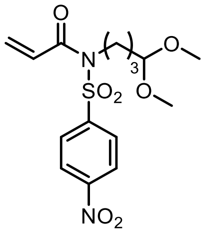

1k

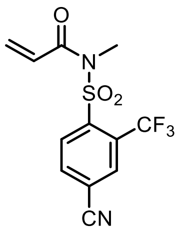

1m

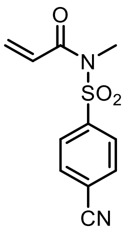

1n

Synthesis of sulfonamides

1oa: *N*-methyl-5-(trifluoromethyl)pyridine-2-sulfonamide

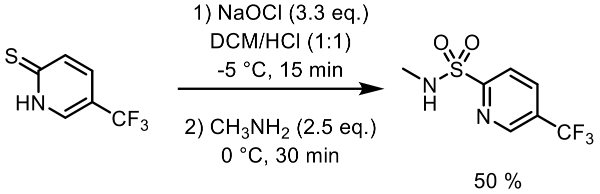

Following a procedure adapted from Gonçalves *et al.*,[5] (trifluormethyl)-2(1*H*)-pyridinethione (896 mg, 5.00 mmol, 1.00 eq.) was added to an Erlenmeyer flask. DCM (25 mL) and aqueous HCl (1 M, 25 mL) were then added and the mixture was cooled to -10 °C. Sodium hypochlorite (13% in H_2O, 7.50 mL, 16.6 mmol, 3.30 eq.) was added slowly with vigorous stirring, ensuring that the temperature did not exceed -5 °C. The mixture was stirred at -10 to -5 °C for 15 min, after which it was transferred to a separatory funnel (precooled). The aqueous phase was collected in an Erlenmeyer flask and cooled to -78 °C. Methylamine (aq. 40%, 1.40 mL, 12.5 mmol, 2.50 eq.) was added slowly and the mixture was allowed to warm to 20 °C and stirred at that temperature for 14 h °C. The mixture was washed with aqueous HCl (1 M), aqueous saturated $NaHCO_3$ solution and dried over $MgSO_4$. The solvent was removed *in vacuo* to afford the title compound as yellow crystals (50%, 606 mg, 2.52 mmol). See NMR.

^{1}H NMR (400 MHz, $CDCl_3$) δ 8.96 (s, 1H), 8.27 – 8.10 (m, 2H), 5.16 (s, 1H), 2.80 (s, 3H).

^{13}C NMR (101 MHz, $CDCl_3$) δ 160.2, 147.2 (q, J = 4.0 Hz), 135.8 (q, J = 3.4 Hz), 129.5 (q, J = 33.9 Hz), 122.7 (q, J = 273.1 Hz), 122.4, 30.0.

^{19}F NMR (376 MHz, $CDCl_3$) δ -62.63.

HRMS (ESI$^+$) *m/z* calculated for $[C_7H_7F_3N_2NaO_2S]^+$ ($[M+Na]^+$) 263.0073, found 263.0056.

ATR-FTIR (cm^{-1}) 3299, 3057, 2924, 1322, 1164, 1141, 1105, 1071, 1012, 847, 720.

1pa: (*E*)-*N*-methyl-2-(4-nitrophenyl)ethene-1-sulfonamide

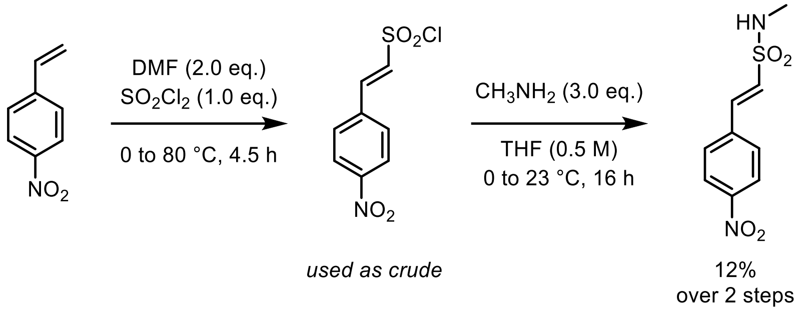

Following a procedure adapted from Zhang *et al.*,[6] sulfuryl chloride (777 µL, 9.58 mmol, 2.00 eq.) was added dropwise to anhydrous dimethylformamide (741 µL, 9.58 mmol, 2.00 eq.) at 0 °C under argon atmosphere. After completion of the addition, the reaction mixture was allowed to warm to 23 °C and stirred for 30 min. 4-Nitrostyrene (614 µL, 4.79 mmol, 1.00 eq.) was added and the reaction mixture was gradually heated to 80 °C in an oil bath under stirring for 16 h. The resulting mixture was cooled to 0 °C, quenched by the addition of crushed ice (30 mL) and extracted with EtOAc (3 x 20 mL). The combined organic phases were washed with H_2O and brine, dried over Na_2SO_4, filtered and concentrated *in vacuo*. The resulting residue (640 mg), consisting of 60% desired product and 40% unreacted alkene, was dissolved in THF (2 mL) under stirring at 23 °C, after which the resulting solution was cooled to 0 °C and methylamine (40% in H_2O, 400 µL, 4.65 mmol, 3.00 eq.) was added dropwise. The reaction mixture was stirred at 23 °C for 16 h, after which it was acidified with aqueous HCl (1 M) to pH = 2, extracted with EtOAc (2 x 20 mL), washed with brine, dried over Na_2SO_4 and concentrated *in vacuo*. Purification by flash column chromatography on silica gel (EtOAc/heptane) afforded the title compound as an orange solid (12% over 2 steps, 140 mg, 0.578 mmol). See NMR.

^{1}H NMR (600 MHz, CDCl$_3$) δ 8.28 (d, *J* = 8.8 Hz, 2H), 7.66 (d, *J* = 8.7 Hz, 2H), 7.54 (d, *J* = 15.5 Hz, 1H), 6.88 (d, *J* = 15.5 Hz, 1H), 4.31 (q, *J* = 5.1 Hz, 1H), 2.81 (d, *J* = 5.3 Hz, 3H).

^{13}C NMR (151 MHz, CDCl$_3$) δ 149.0, 139.3, 138.8, 129.0 (2C), 128.6, 124.5 (2C), 29.3.

HRMS (ESI$^+$) m/z calculated for $[C_9H_{10}N_2O_4SNa]^+$ ($[M+Na]^+$) 265.0253, found 265.0256.

ATR-FTIR (cm^{-1}) 3297, 3058, 1599, 1519, 1347, 1324, 1310, 1148, 1128, 849, 741, 664.

1qa: (*E*)-*N*-methyl-2-phenylethene-1-sulfonamide

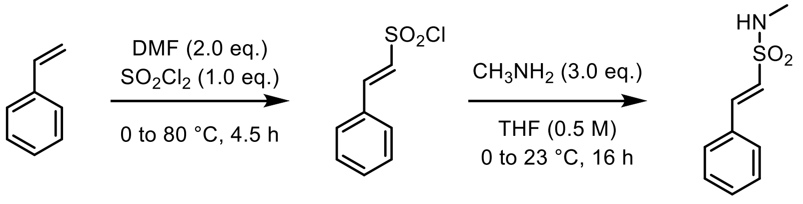

Following a procedure adapted from Zhang *et al.,*[6] sulfuryl chloride (1.62 mL, 20.0 mmol, 2.00 eq.) was added dropwise to anhydrous dimethylformamide (1.55 mL, 20.0 mmol, 2.00 eq.), mL mmol eq.at 0 °C under argon atmosphere. After completion of the addition, the reaction mixture was allowed to warm to 23 °C and stirred for 30 min. Styrene (1.15 mL, 10.0 mmol, 1.00 eq.) was added and the reaction mixture was gradually heated to 80 °C in an oil bath under stirring for 4 h. The resulting mixture was cooled to 0 °C, quenched by the addition of crushed ice (30 mL) and extracted with EtOAc (3 x 20 mL). The combined organic phases were washed with H_2O and brine, dried over Na_2SO_4, filtered and concentrated *in vacuo*. The resulting residue (1.33 g) was dissolved in THF (6.5 mL) under stirring. Methylamine (40% in H_2O, 1.70 mL, 19.7 mmol, 3.00 eq.) was added dropwise at 0 °C. The reaction mixture was stirred at room temperature for 16 h, after which it was acidified with aqueous HCl (1 M) to pH = 2, extracted with EtOAc (2 x 40 mL), washed with brine, dried over Na_2SO_4 and concentrated *in vacuo*. Purification by flash column chromatography on silica gel (EtOAc/heptane) afforded the title compound as a yellow solid (37% over 2 steps, 727 mg, 3.69 mmol). See NMR.

^{1}H NMR (600 MHz, $CDCl_3$) δ 7.52 – 7.47 (m, 3H), 7.42 – 7.40 (m, 3H), 6.74 (d, *J* = 15.5 Hz, 1H), 4.59 (q, *J* = 4.9 Hz, 1H), 2.76 (d, *J* = 5.3 Hz, 3H).

^{13}C NMR (151 MHz, $CDCl_3$) δ 142.5, 132.6, 131.0, 129.2, 128.4, 124.0, 29.2.

HRMS (ESI$^+$) *m/z* calculated for $[C_9H_{11}NO_2SNa]^+$ ($[M+Na]^+$) 220.0403, found 220.0404.

ATR-FTIR (cm^{-1}) 3288, 3057, 2941, 1618, 1315, 1196, 1180, 862, 840, 742, 532.

1ra: (*E*)-*N*-methyl-2-(naphthalen-2-yl)ethene-1-sulfonamide

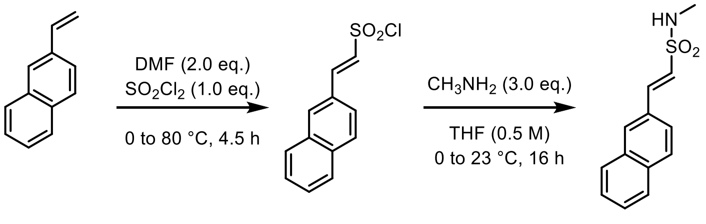

The procedure was inspired by Zhang *et al.*.[6] To anhydrous dimethylformamide (0.57 mL, 7.4 mmol, 2.0 eq.), sulfuryl chloride (0.6 mL, 7.4 mmol, 2.0 eq.) was added dropwise at 0 °C under argon atmosphere. After the addition was finished, the reaction mixture was allowed to warm to 23 °C and stirred for 30 min. 2-Vinylnaphthalene (0.51 mL, 3.7 mmol, 1.0 eq.) was added and the reaction mixture was gradually heated to 80 °C in an oil bath under stirring for 4 h. The resulting mixture was cooled to 0 °C, quenched by the addition of crushed ice and extracted with EtOAc (3 x 20 mL). The organic phase was washed with H_2O and brine, dried over Na_2SO_4, filtered and concentrated *in vacuo*. The resulting residue (935 mg) was dissolved in THF (7.4 mL) under stirring. Methylamine (40% in H_2O, 1.23 mL, 11.1 mmol, 3.0 eq.) was added dropwise at 0 °C. The reaction mixture was stirred at 23 °C for 16 h, after which it was acidified with aqueous HCl (1 M) to pH = 2, extracted with EtOAc (2 x 30 mL), washed with brine, dried over Na_2SO_4 and concentrated *in vacuo*. Purification by flash column chromatography on silica gel (EtOAc/heptane) afforded the title compound as a pale yellow solid (22% over 2 steps, 203 mg, 813 µmol). See NMR.

^{1}H NMR (700 MHz, $CDCl_3$) δ 7.94 (s, 1H), 7.91 – 7.80 (m, 3H), 7.67 (d, *J* = 15.4 Hz, 1H), 7.60 (dd, *J* = 8.6, 1.7 Hz, 1H), 7.57 – 7.47 (m, 2H), 6.83 (d, *J* = 15.4 Hz, 1H), 4.23 (app d, *J* = 5.2 Hz, 1H), 2.80 (d, *J* = 5.4 Hz, 3H).

^{13}C NMR (176 MHz, $CDCl_3$) δ 142.7, 134.6, 133.3, 130.6, 130.1, 129.2, 128.8, 128.0, 127.8, 127.2, 124.1, 123.4, 29.3.

HRMS (ESI$^+$) *m/z* calculated for $[C_{13}H_{13}NO_2S]^+$ ($[M+H]^+$) 270.0559, found 270.0552.

ATR-FTIR (cm^{-1}) 3300, 3052, 2921, 2852, 1614, 1319, 1274, 1149, 1129, 1070, 857, 806, 744, 477.

Synthesis of sulfonyl acrylimides

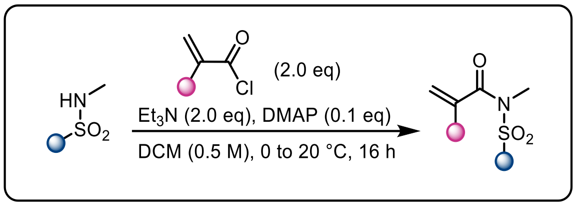

General Procedure A:

To a flame-dried flask were added sulfonamide (1.0 eq.), triethylamine (2.0 eq.), 4-dimethylaminopyridine (DMAP, 0.1 eq.) and DCM (0.5 M). The solution was cooled to 0 °C and the corresponding acryloyl chloride (2.0 eq.) was added dropwise. The reaction mixture was allowed to warm to 20 °C and stirred for 16 h. Afterwards, the reaction was quenched with aqueous saturated $NaHCO_3$ solution, transferred to a separatory funnel and extracted with DCM. The combined organic layers were dried over Na_2SO_4, filtered and concentrated *in vacuo*. Purification was performed by flash column chromatography on silica gel (EtOAc/heptane).

1l: *N*-methyl-*N*-((4-nitrophenyl)sulfonyl)methacrylamide

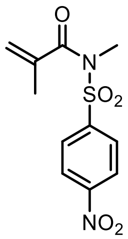

The product was synthesised according to **General Procedure B** using *N*-methyl-4-nitrobenzenesulfonamide (1.08 g, 5.00 mmol, 1.00 eq.) and methacryloyl chloride (1.05 g, 10.0 mmol, 2.00 eq.), and obtained as a white solid (70%, 989 mg, 3.48 mmol). See NMR.

^{1}H NMR (400 MHz, $CDCl_3$) δ 8.40 (d, *J* = 9.0 Hz, 2H), 8.20 (d, *J* = 9.0 Hz, 2H), 5.47 (d, *J* = 1.5 Hz, 1H), 5.30 (s, 1H), 3.34 (s, 3H), 1.95 (s, 3H).

^{13}C NMR (151 MHz, $CDCl_3$) δ 172.1, 150.8, 144.4, 129.8 (2C), 124.2 (2C), 121.3, 35.1, 19.2.

HRMS (ESI$^+$) *m/z* calculated for $[C_{11}H_{12}N_2NaO_5S]^+$ ($[M+Na]^+$) 307.0360, found 307.0354.

ATR-FTIR (cm^{-1}) 3112, 2957, 2930, 2158, 1695, 1533, 1351, 1177, 1027, 936, 856, 610.

1o: *N*-methyl-*N*-((5-(trifluoromethyl)pyridin-2-yl)sulfonyl)acrylamide

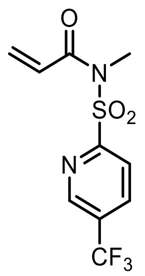

The product was synthesized according to **General Procedure A** using *N*-methyl-5-(trifluoromethyl)pyridine-2-sulfonamide (480 mg, 2.00 mmol, 1.00 eq.) and acryloyl chloride (434 mg, 4.80 mmol, 2.40 eq.), and obtained as colourless crystals (30%, 177 mg, 0.602 mmol). See NMR.

^{1}H NMR (600 MHz, $CDCl_3$) δ 8.93 (s, 1H), 8.27 – 8.19 (m, 2H), 6.99 (dd, *J* = 16.7, 10.5 Hz, 1H), 6.41 (dd, *J* = 16.7, 1.5 Hz, 1H), 5.83 (dd, *J* = 10.5, 1.5 Hz, 1H), 3.43 (s, 3H).

^{13}C NMR (151 MHz, $CDCl_3$) δ 166.5, 159.7, 147.5 (q, *J* = 3.9 Hz), 136.0 (q, *J* = 3.5 Hz), 132.0, 130.2 (q, *J* = 33.9 Hz), 128.4, 122.9, 122.5 (q, *J* = 273.4 Hz), 34.1.

^{19}F NMR (376 MHz, $CDCl_3$) δ -62.68.

HRMS (ESI$^+$) *m/z* calculated for $[C_{10}H_9F_3N_2NaO_3S]^+$ ($[M+Na]^+$) 317.0178, found 317.0175.

ATR-FTIR (cm^{-1}) 1693, 1366, 1327, 1173, 1138, 1104, 1073, 1013, 730, 621.

1p: (*E*)-*N*-methyl-*N*-((4-nitrostyryl)sulfonyl)acrylamide

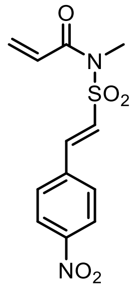

The product was synthesised according to **General Procedure A** using (*E*)-*N*-methyl-2-(4-nitrophenyl)ethene-1-sulfonamide (140 mg, 0.578 mmol, 1.00 eq.) and acryloyl chloride (105 mg, 1.16 mmol, 2.00 eq.), and obtained as a yellow solid (57%, 98.0 mg, 0.331 mmol). See NMR.

^{1}H NMR (700 MHz, $CDCl_3$) δ 8.28 (d, *J* = 8.8 Hz, 2H), 7.69 (d, *J* = 8.7 Hz, 2H), 7.66 (d, *J* = 15.5 Hz, 1H), 7.12 (d, *J* = 15.4 Hz, 1H), 6.91 (dd, *J* = 16.7, 10.4 Hz, 1H), 6.49 (dd, *J* = 16.7, 1.5 Hz, 1H), 5.90 (dd, *J* = 10.5, 1.5 Hz, 1H), 3.35 (s, 3H).

^{13}C NMR (176 MHz, $CDCl_3$) δ 166.3, 149.4, 141.0, 137.9, 132.4, 129.5 (2C), 128.3, 128.1, 124.5 (2C), 32.9.

HRMS (ESI$^+$) *m/z* calculated for $[C_{12}H_{12}N_2O_5SNa]^+$ ($[M+Na]^+$) 319.0359, found 319.0351.

ATR-FTIR (cm^{-1}) 3113, 3076, 2958, 1687, 1493, 1346, 1147, 847, 742.

1q: (*E*)-*N*-methyl-*N*-(styrylsulfonyl)acrylamide

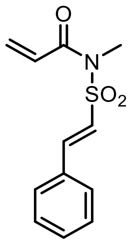

The product was synthesized according to **General Procedure A** using (*E*)-*N*-methyl-2-phenylethene-1-sulfonamide (3.29 g, 16.7 mmol, 1.00 eq.) and acryloyl chloride (3.02 g, 33.4 mmol, 2.00 eq.), and obtained as a colourless viscous oil (28%, 1.16 g, 4.62 mmol). See NMR.

^{1}H NMR (600 MHz, $CDCl_3$) δ 7.61 (d, *J* = 15.4 Hz, 1H), 7.54 – 7.48 (m, 2H), 7.48-7.40 (m, 3H), 7.00 (dd, *J* = 16.7, 10.5 Hz, 1H), 6.92 (d, *J* = 15.4 Hz, 1H), 6.46 (dd, *J* = 16.7, 1.6 Hz, 1H), 5.85 (dd, *J* = 10.4, 1.6 Hz, 1H), 3.32 (s, 3H).

^{13}C NMR (151 MHz, $CDCl_3$) δ 166.4, 144.2, 131.9, 131.8, 131.6, 129.4, 128.8 (2C), 128.6 (2C), 123.8, 32.8.

HRMS (ESI) m/z calculated for $[C_{12}H_{13}NNaO_3S]^+$($[M+Na]^+$) 274.0508, found 274.0507.

ATR-FTIR (cm^{-1}): 3060, 2954, 1681, 1613, 1352, 1146, 1014, 908, 817, 743.

1r: (*E*)-*N*-methyl-*N*-((2-(naphthalen-2-yl)vinyl)sulfonyl)acrylamide

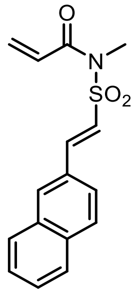

The product was synthesized according to **General Procedure A** using (*E*)-*N*-methyl-2-(naphthalen-2-yl)ethene-1-sulfonamide (200 mg, 0.808 mmol, 1.00 eq.) and acryloyl chloride (146 mg, 1.62 mmol, 2.00 eq.), and was obtained as an off-white solid (25%, 62.0 mg, 0.206 mmol). See NMR.

^{1}H NMR (700 MHz, $CDCl_3$) δ 7.97 (s, 1H), 7.90 – 7.85 (m, 3H), 7.78 (d, *J* = 15.3 Hz, 1H), 7.62-7.52 (m, 3H), 7.03 (dd, *J* = 16.1, 10.2 Hz, 2H), 6.47 (dd, *J* = 16.7, 1.6 Hz, 1H), 5.86 (dd, *J* = 10.4, 1.6 Hz, 1H), 3.35 (s, 3H).

^{13}C NMR (176 MHz, $CDCl_3$) δ 166.4, 144.3, 134.9, 133.3, 131.6, 131.5, 129.3, 129.3, 128.9, 128.6, 128.3, 128.1, 127.3, 123.8, 123.4, 32.8.

HRMS (ESI) m/z calculated for $[C_{16}H_{15}NNaO_3S]^+$($[M+Na]^+$) 324.6057, found 324.6066.

ATR-FTIR (cm^{-1}): 3058, 2954, 2922, 1684, 1353, 1149, 910, 745, 733.

4. Synthesis of α,β-substituted amides

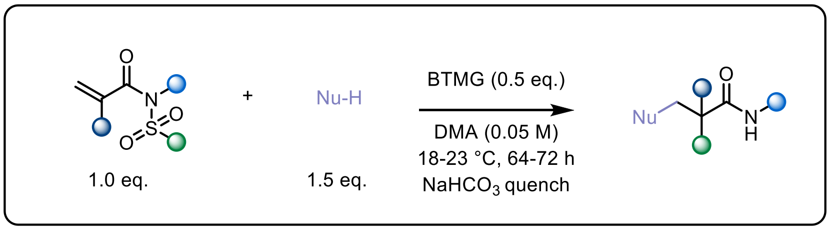

General Procedure B:

An oven-dried flask or vial was successively charged with a stirring bar, the corresponding nucleophile (1.5 eq.), DMA (0.05 M) and BTMG (0.5 eq.). The mixture was stirred for 10 min at ambient temperature, before addition of the sulfonyl acrylimide. The resulting solution was stirred for an additional 64-67 h at ambient temperature. Afterwards, the mixture was transferred to a separatory funnel with DCM and quenched by addition of aqueous saturated $NaHCO_3$ solution. After separation of phases, the aqueous phase was extracted with DCM (2 x 10 mL). The combined organic layers were washed with brine, dried over Na_2SO_4, filtered and concentrated *in vacuo*. Two equivalents of mesitylene were added as NMR standard, before an NMR spectrum of the crude mixture was taken. Purification was performed by flash column chromatography on silica gel (EtOAc/heptane).

Unsuccessful substrates

The expected products, corresponding to the following starting materials, could only be obtained in low yields (<20%) or were not detected:

Sulfonyl imides

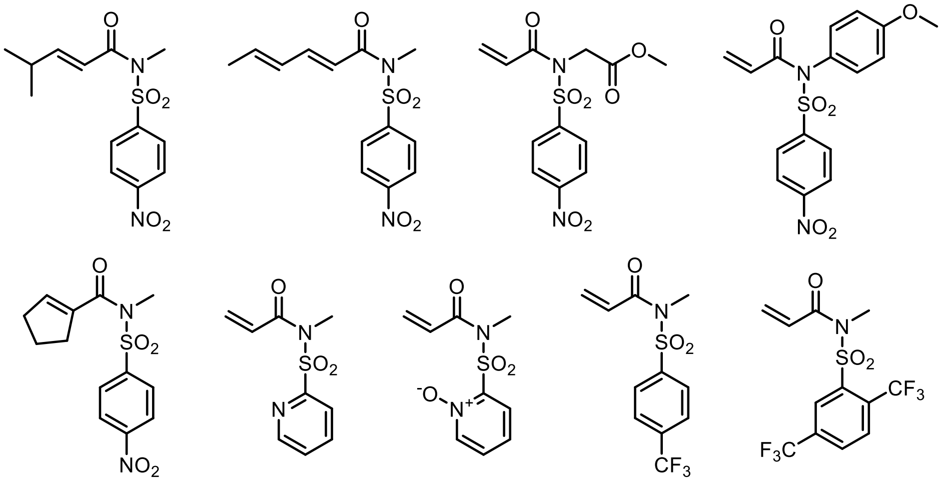

Pronucleophiles

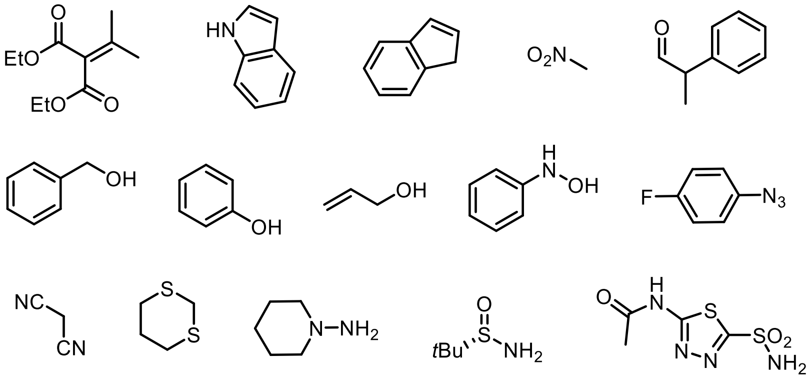

Rerouted Michael-Smiles

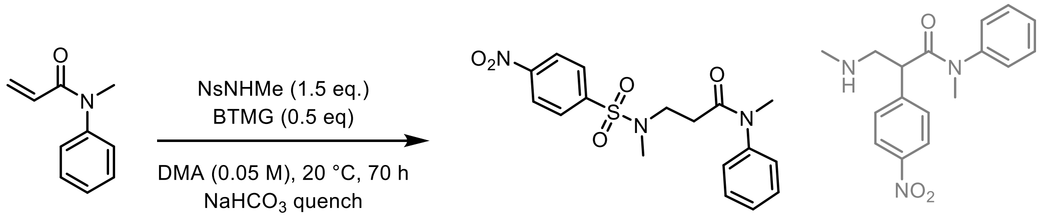

S1 **S2** *Not detected*

S1: *N*-methyl-*N*-phenylacrylamide

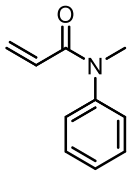

To a solution of aniline (536 mg, 5.0 mmol, 1.00 eq) and triethylamine (557 mg, 5.5 mmol, 1.10 eq) in DCM (0.25 M) at 0 °C was added dropwise acrylyl chloride (0.447 mL, 5.5 mmol, 1.10 eq) over 15 minutes. The reaction was allowed to warm to 20 °C and stirred for 16 hours. Water was added to quench the reaction and the reaction was diluted with DCM (30 mL). The organic layer was washed sequentially with aqueous HCl (1 M, 30 mL) and brine (30 mL). The organic layer was dried with $MgSO_4$, filtered and concentrated *in vacuo*. Purification by flash column chromatography (eluent heptane/EtOAc 3:7) afforded the product as a pale blue crystal (75%, 604 mg, 3.75 mmol). See NMR.

^{1}H NMR (600 MHz, CDCl$_3$) δ 7.40 (dt, *J* = 10.0, 2.0 Hz, 2H), 7.32 (t, *J* = 7.4 Hz, 1H), 7.21 – 7.08 (m, 2H), 6.35 (dd, *J* = 16.5, 1.8 Hz, 1H), 6.06 (dd, *J* = 16.5, 10.4 Hz, 1H), 5.50 (dd, *J* = 10.4, 1.8 Hz, 1H), 3.35 (s, 3H).

^{13}C NMR (151 MHz, CDCl$_3$) δ 165.8, 143.6, 129.7 (2C), 128.6, 127.7, 127.5, 127.4 (2C), 37.5.

HRMS (ESI$^+$) *m/z* calculated for [$C_{10}H_{12}NO^+$] ([M+H]$^+$) 162.0913, found 162.0915.

ATR-FTIR (cm^{-1}): 3065, 2359, 1657, 1615, 1595, 1496, 1423, 1305, 1123, 793, 700.

S2: *N*-methyl-3-((*N*-methyl-4-nitrophenyl)sulfonamido)-*N*-phenylpropanamide

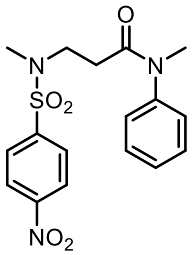

The product was synthesised according to **General procedure B**, using **SI1** (16.1 mg, 0.10 mmol, 1.00 eq.) and *N*-methyl-4-nitrobenzenesulfonamide (32.4 mg, 0.15 mmol, 1.50 eq.), and obtained as a yellow solid (65%, 24.4 mg, 0.065 mmol). See NMR.

^{1}H NMR (600 MHz, CDCl$_3$) δ 8.32 (d, *J* = 8.7 Hz, 2H), 7.90 (d, *J* = 8.7 Hz, 2H), 7.46 – 7.40 (m, 2H), 7.38 (t, *J* = 7.4 Hz, 1H), 7.17 (d, *J* = 7.4 Hz, 2H), 3.37 (t, *J* = 7.2 Hz, 2H), 3.25 (s, 3H), 2.77 (s, 3H), 2.35 (t, *J* = 7.2 Hz, 2H).

^{13}C NMR (151 MHz, CDCl$_3$) δ 170.2, 150.2, 143.8, 143.5, 130.2 (2C), 128.5 (2C), 128.4, 127.4 (2C), 124.5 (2C), 47.1, 37.5, 35.9, 33.6.

HRMS (ESI$^+$) *m/z* calculated for [$C_{17}H_{19}N_3O_5NaS^+$] ([M+Na]$^+$) 400.0938, found 400.0926.

ATR-FTIR (cm^{-1}): 3104, 2940, 1653, 1595, 1349, 1164, 741, 601.

4a: Dimethyl 2-methyl-2-(3-(methylamino)-2-(4-nitrophenyl)-3-oxopropyl)malonate

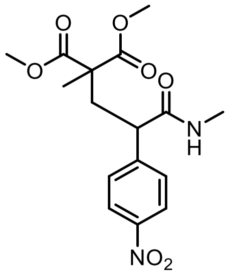

The product was synthesised according to **General Procedure B**, using *N*-methyl-*N*-((4-nitrophenyl)sulfonyl)acrylamide (27.0 mg, 0.100 mmol, 1.00 eq.) and dimethyl methylmalonate (21.9 mg, 0.150 mmol, 1.50 eq.), and obtained as a white solid (88%, 31.0 mg, 0.088 mmol). See NMR.

The scale up was performed under the same procedure, using *N*-methyl-*N*-((4-nitrophenyl)sulfonyl)acrylamide (270 mg, 1.00 mmol, 1.00 eq.) and dimethyl methylmalonate (219 mg, 1.50 mmol, 1.5 eq.), and obtained as a white solid (76%, 267 mg, 0.758 mmol).

^{1}H NMR (700 MHz, $CDCl_3$) δ 8.17 (d, *J* = 8.7 Hz, 2H), 7.50 (d, *J* = 8.7 Hz, 2H), 5.55 (d, *J* = 4.1 Hz, 1H), 3.70 (dd, *J* = 7.3, 4.5 Hz, 1H), 3.65 (s, 3H), 3.62 (s, 3H), 2.86 (dd, *J* = 14.5, 7.3 Hz, 1H), 2.76 (d, *J* = 4.8 Hz, 3H), 2.20 (dd, *J* = 14.5, 4.5 Hz, 1H), 1.43 (s, 3H).

^{13}C NMR (176 MHz, $CDCl_3$) δ 172.4, 172.2, 172.0, 148.1, 147.3, 129.0 (2C), 124.0 (2C), 53.3, 52.74, 52.73, 49.3, 39.8, 26.9, 21.4.

HRMS (ESI$^+$) *m/z* calculated for $[C_{16}H_{20}N_2NaO_7]^+$ ($[M+Na]^+$): 375.1163, found 375.1164.

ATR-FTIR (cm^{-1}) 3399, 3306, 3080, 3000, 2953, 1731, 1652, 1521, 1382, 1077, 856, 739.

4b: Diethyl 2-benzyl-2-(3-(methylamino)-2-(4-nitrophenyl)-3-oxopropyl)malonate

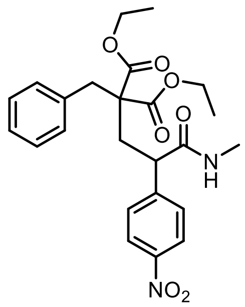

The product was synthesised according to **General Procedure B**, using *N*-methyl-*N*-((4-nitrophenyl)sulfonyl)acrylamide (27.0 mg, 0.100 mmol, 1.00 eq.) and diethyl benzylmalonate (21.9 mg, 0.150 mmol, 1.50 eq.), and obtained as a white solid (75%, 34.3 mg, 0.075 mmol). See NMR.

^{1}H NMR (600 MHz, CDCl$_3$) δ 8.13 (d, *J* = 8.6 Hz, 2H), 7.46 (d, *J* = 8.6 Hz, 2H), 7.24 – 7.17 (m, 3H), 7.07 (d, *J* = 6.5 Hz, 2H), 5.61 (d, *J* = 4.5 Hz, 1H), 4.17 – 4.08 (m, 1H), 4.05 – 3.91 (m, 3H), 3.74 (dd, *J* = 6.8, 4.7 Hz, 1H), 3.28 (app q, *J* = 14.0 Hz, 2H), 2.79 (dt, *J* = 24.6, 12.3 Hz, 1H), 2.72 (d, *J* = 4.8 Hz, 3H), 2.12 (dd, *J* = 14.5, 4.5 Hz, 1H), 1.25 (t, *J* = 7.1 Hz, 3H), 1.18 (t, *J* = 7.1 Hz, 3H).

^{13}C NMR (151 MHz, CDCl$_3$) δ 172.1, 171.0, 170.9, 148.1, 147.2, 135.5, 130.1 (2C), 129.1 (2C), 128.6 (2C), 127.4, 123.9 (2C), 61.7, 61.6, 58.2, 49.0, 40.5, 37.0, 26.8, 14.0, 13.9.

HRMS (ESI$^+$) *m/z* calculated for $[C_{24}H_{28}N_2NaO_7]^+$ ($[M+Na]^+$) 479.1789, found 479.1784.

ATR-FTIR (cm^{-1}) 3387, 3308, 2980, 1725, 1649, 1520, 736, 701.

4c: Dimethyl 2-(3-(methylamino)-2-(4-nitrophenyl)-3-oxopropyl)malonate

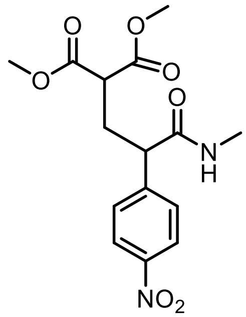

The product was synthesised according to **General Procedure B**, using *N*-methyl-*N*-((4-nitrophenyl)sulfonyl)acrylamide (27.0 mg, 0.100 mmol, 1.00 eq.) and dimethyl malonate (19.8 mg, 0.15 mmol, 1.50 eq.), and obtained as a white solid (72%, 24.2 mg, 0.072 mmol). See NMR.

^{1}H NMR (700 MHz, CDCl$_3$) δ 8.26 – 8.12 (m, 2H), 7.55 – 7.44 (m, 2H), 5.55 (t, *J* = 14.9 Hz, 1H), 3.73 (s, 3H), 3.72 (s, 3H), 3.62 – 3.54 (m, 1H), 3.38 – 3.26 (m, 1H), 2.78 (t, *J* = 6.0 Hz, 3H), 2.73 – 2.54 (m, 1H), 2.38 – 2.24 (m, 1H).

^{13}C NMR (176 MHz, CDCl$_3$) δ 171.4, 169.5, 169.3, 147.6, 146.3, 129.1 (2C), 124.2 (2C), 52.89, 52.87, 50.2, 49.4, 32.6, 26.8.

HRMS (ESI$^+$) *m/z* calculated for $[C_{15}H_{18}N_2O_7Na^+]$ ($[M+Na]^+$) 361.1006, found 361.1004.

ATR-FTIR (cm^{-1}) 3312, 3048, 2853, 1732, 1562, 1604, 1520, 1346, 1156, 856, 740, 698.

4d: 4-acetyl-*N*,4-dimethyl-2-(4-nitrophenyl)-5-oxohexanamide

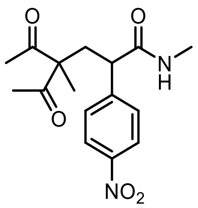

The product was synthesised according to **General Procedure B**, using *N*-methyl-*N*-((4-nitrophenyl)sulfonyl)acrylamide (27.0 mg, 0.10 mmol, 1.00 eq.) and 3-methyl-2,4-pentanedione (17.1 mg, 0.15 mmol, 1.50 eq.), and obtained as a yellow oil (80%, 25.6 mg, 0.080 mmol). See NMR.

^{1}H NMR (600 MHz, CDCl$_3$) δ 8.14 (d, *J* = 8.6 Hz, 2H), 7.50 (d, *J* = 8.6 Hz, 2H), 5.71 (d, *J* = 4.2 Hz, 1H), 3.46 (dd, *J* = 8.1, 3.3 Hz, 1H), 2.80 (dd, *J* = 14.4, 8.2 Hz, 1H), 2.71 (d, *J* = 4.8 Hz, 3H), 2.14 (s, 3H), 2.07 (dd, *J* = 14.4, 3.3 Hz, 1H), 2.04 (s, 3H), 1.37 (s, 3H).

^{13}C NMR (151 MHz, CDCl$_3$) δ 208.8, 207.5, 172.1, 148.5, 147.2, 128.8 (2C), 124.1 (2C), 65.7, 48.9, 38.4, 27.0, 26.9, 26.8, 19.9.

HRMS (ESI$^+$) m/z calculated for $[C_{16}H_{20}N_2O_5Na]^+$ ($[M+Na]^+$) 343.1264, found 343.1270.

ATR-FTIR (cm^{-1}) 3307, 2941, 1696, 1652, 1604, 1519, 1345, 856, 740, 561.

4e: N-methyl-2-(4-nitrophenyl)-4,4-bis(phenylsulfonyl)butanamide

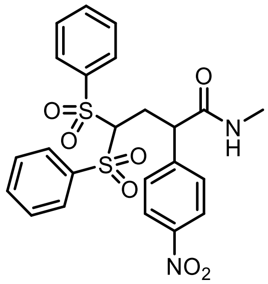

The product was synthesised according to **General Procedure B**, using *N*-methyl-*N*-((4-nitrophenyl)sulfonyl)acrylamide (27.0 mg, 0.100 mmol, 1.00 eq.) and bis(phenylsulfonyl)methane (44.9 mg, 0.150 mmol, 1.50 eq.), and obtained as a colourless oil (70%, 35.0 mg, 0.070 mmol). See NMR.

^{1}H NMR (700 MHz, CDCl$_3$) δ 8.05 (d, *J* = 8.7 Hz, 2H), 7.90 (dd, *J* = 8.4, 1.1 Hz, 2H), 7.78 (dd, *J* = 8.4, 1.1 Hz, 2H), 7.73 – 7.68 (m, 2H), 7.58 (dd, *J* = 8.3, 7.6 Hz, 2H), 7.52 (dd, *J* = 8.3, 7.6 Hz, 2H), 7.34 (d, *J* = 8.7 Hz, 2H), 5.79 (d, *J* = 4.7 Hz, 1H), 4.33 (dd, *J* = 7.4, 5.2 Hz, 1H), 4.28 – 4.22 (m, 1H), 2.87 – 2.81 (m, 1H), 2.72 (d, *J* = 4.8 Hz, 3H), 2.64 (ddd, *J* = 15.5, 8.7, 5.1 Hz, 1H).

^{13}C NMR (176 MHz, CDCl$_3$) δ 170.9, 147.6, 144.8, 137.7, 137.2, 135.04, 135.00, 129.64 (2C), 129.58 (2C), 129.44 (2C), 129.38 (2C), 129.2 (2C), 124.3 (2C), 80.5, 49.4, 29.8, 26.8.

HRMS (ESI$^+$) *m/z* calculated for $[C_{23}H_{22}N_2O_7S_2Na]^+$ ($[M+Na]^+$) 525.0761, found 525.0753.

ATR-FTIR (cm^{-1}) 3399, 3306, 3080, 3000, 2953, 1731, 1652, 1521, 1382, 1077, 856, 739.

4f: Ethyl 5-(methylamino)-4-(4-nitrophenyl)-5-oxo-2-(pyridin-2-yl)pentanoate

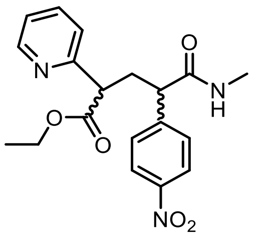

1.1 : 1 mixture of diastereoisomers

The product was synthesised according to **General Procedure B**, using *N*-methyl-*N*-((4-nitrophenyl)sulfonyl)acrylamide (27.0 mg, 0.100 mmol, 1.00 eq.) and ethyl 2-(pyridin-2-yl)acetate (22.9 µl, 0.150 mmol, 1.50 eq.), and obtained as a yellow oil (50%, 18.5 mg, 0.050 mmol). The product consists of a 1.1:1 mixture of diastereoisomers (*di* **A** and **B**). See NMR.

^{1}H NMR (600 MHz, $CDCl_3$) δ 8.55 (q, *J* = 4.6 Hz, 1H, *di* **A** + **B**), 8.14 (dd, *J* = 10.0, 8.9 Hz, 2H, *di* **A** + **B**), 7.65 (td, *J* = 7.7, 1.5 Hz, 1H, *di* **A** + **B**), 7.46 (t, *J* = 8.6 Hz, 2H, *di* **A** + **B**), 7.21 (dt, *J* = 12.0, 6.7 Hz, 2H, *di* **A** + **B**), 5.92 (q, *J* = 4.1 Hz, 1H, *di* **B**), 5.64 (d, *J* = 3.9 Hz, 1H, *di* **A**), 4.18 – 4.08 (m, 2H, *di* **A** + **B**), 3.73 (dt, *J* = 15.0, 7.6 Hz, 1H, *di* **A** + **B**), 3.50 (t, *J* = 7.5 Hz, 1H, *di* **A**), 3.44 – 3.39 (m, 1H, *di* **B**), 2.92 – 2.84 (m, 1H, *di* **B**), 2.78 (d, *J* = 4.8 Hz, 3H, *di* **B**), 2.77 – 2.71 (m, 1H, *di* **A**), 2.75 (d, *J* = 4.8 Hz, 3H, *di* **A**), 2.61 – 2.54 (m, 1H, *di* **A**), 2.41 – 2.34 (m, 1H, *di* **B**), 1.17 (dt, *J* = 14.5, 7.1 Hz, 3H, *di* **A** + **B**).

^{13}C NMR (151 MHz, $CDCl_3$) δ 172.4, 172.3, 172.0, 172.0, 158.1, 157.8, 149.7, 149.6, 147.4, 147.3, 147.0 (2C), 137.2, 137.0, 129.2 (2C), 129.1 (2C), 124.01, 123.95, 123.4, 122.9, 122.7, 122.6, 61.4, 61.3, 51.4, 51.3, 50.6, 50.1, 36.1, 35.8, 26.7, 26.7, 14.2, 14.2 ppm.

HRMS (ESI$^+$) *m/z* calculated for $[C_{19}H_{21}N_3O_5Na]^+$ ($[M+Na]^+$) 394.1373, found 394.1378.

ATR-FTIR (cm^{-1}) 3302, 3079, 2939, 1730, 1651, 1590, 1346, 1027, 856, 747, 540.

4g: Dimethyl 2-(3-(isopropylamino)-2-(4-nitrophenyl)-3-oxopropyl)-2-methylmalonate

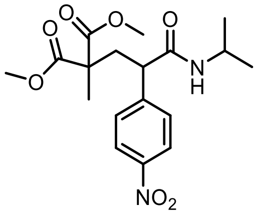

The product was synthesised according to **General Procedure B**, using *N*-isopropyl-*N*-((4-nitrophenyl)sulfonyl)acrylamide (29.8 mg, 0.100 mmol, 1.00 eq.) and dimethyl methylmalonate (21.9 mg, 0.150 mmol, 1.50 eq.), and obtained as a white solid (28.8 mg, 76%, 0.076 mmol). See NMR.

^{1}H NMR (400 MHz, $CDCl_3$) δ 8.22 – 8.08 (m, 2H), 7.55 – 7.44 (m, 2H), 5.39 (d, *J* = 7.6 Hz, 1H), 4.04 – 3.91 (m, 1H), 3.72 – 3.57 (m, 7H), 2.83 (dd, *J* = 14.5, 7.4 Hz, 1H), 2.18 (dd, *J* = 14.5, 4.4 Hz, 1H), 1.42 (s, 3H), 1.15 (d, *J* = 6.6 Hz, 3H), 1.00 (d, *J* = 6.5 Hz, 3H).

^{13}C NMR (101 MHz, $CDCl_3$) δ 172.4, 172.2, 170.5, 148.4, 147.2, 128.9 (2C), 124.0 (2C), 53.4, 52.7, 49.4, 42.0, 39.6, 22.6, 22.5, 21.2, 14.2.

HRMS (ESI$^+$) *m/z* calculated for $[C_{18}H_{24}N_2O_7Na]^+$ ($[M+Na]^+$) 403.1476, found 403,1479.

ATR-FTIR (cm^{-1}) 3384, 3313, 2973, 1731, 1650, 1605, 1521, 1492, 1347, 1110, 856, 740, 699.

4h: Dimethyl 2-(3-(*tert*-butylamino)-2-(4-nitrophenyl)-3-oxopropyl)-2-methylmalonate

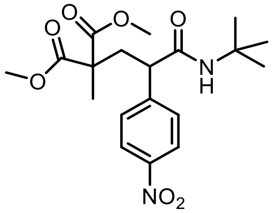

The product was synthesised according to **General Procedure B**, using *N*-(*tert*-butyl-*N*-((4-nitrophenyl)sulfonyl)acrylamide (31.2 mg, 0.100 mmol, 1.00 eq.) and dimethyl methylmalonate (21.9 mg, 0.150 mmol, 1.50 eq.), and obtained as a white solid (22.0 mg, 56%, 0.056 mmol). See NMR.

^{1}H NMR (400 MHz, $CDCl_3$) δ 8.22 – 8.13 (m, 2H), 7.52 – 7.40 (m, 2H), 5.38 (s, 1H), 3.68 (s, 3H), 3.64 – 3.55 (m, 4H), 2.81 (dd, *J* = 14.5, 7.4 Hz, 1H), 2.14 (dd, *J* = 14.5, 4.2 Hz, 1H), 1.43 (s,3H), 1.27 (s, 9H).

^{13}C NMR (101 MHz, $CDCl_3$) δ 172.5, 172.3, 170.5, 148.7, 147.2, 128.8 (2C), 124.0 (2C), 53.5, 52.8, 52.7, 51.7, 49.9, 39.6, 28.7 (3C), 21.2.

HRMS (ESI$^+$) *m/z* calculated for $[C_{19}H_{26}N_2O_7Na]^+$ ($[M+Na]^+$) 417.1632, found 417.1635.

ATR-FTIR (cm^{-1}) 3392, 2957, 1728, 1679, 1604, 1518, 1454, 1345, 982, 858, 699.

4i: Dimethyl 2-(3-(benzylamino)-2-(4-nitrophenyl)-3-oxopropyl)-2-methylmalonate

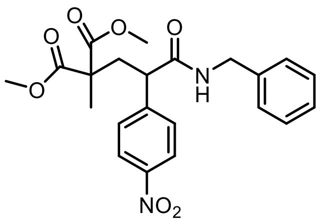

The product was synthesised according to **General Procedure C**, using *N*-benzyl-*N*-((4-nitrophenyl)sulfonyl)acrylamide (34.6 mg, 0.100 mmol, 1.00 eq.) and dimethyl methylmalonate (21.9 mg, 0.15 mmol, 1.50 eq.), and obtained as a white solid (30.0 mg, 70%, 0.070 mmol). See NMR.

^{1}H NMR (600 MHz, CDCl$_3$) δ 8.15 (d, *J* = 8.6 Hz, 2H), 7.50 (d, *J* = 8.6 Hz, 2H), 7.29 – 7.22 (m, 3H), 7.16 (d, *J* = 7.1 Hz, 2H), 5.92 (t, *J* = 5.3 Hz, 1H), 4.37 (d, *J* = 5.3 Hz, 2H), 3.76 (dd, *J* = 7.3, 4.3 Hz, 1H), 3.62 (s, 3H), 3.61 (s, 3H), 2.89 (dd, *J* = 14.5, 7.3 Hz, 1H), 2.22 (dd, *J* = 14.5, 4.3 Hz, 1H), 1.43 (s, 3H).

^{13}C NMR (151 MHz, CDCl$_3$) δ 172.4, 172.2, 171.3, 148.1, 147.3, 137.8, 128.9 (2C), 128.8 (2C), 127.9 (2C), 127.8, 124.0 (2C), 53.3, 52.74, 52.72, 49.3, 44.2, 39.6, 21.3.

HRMS (ESI$^+$) *m/z* calculated for $[C_{22}H_{24}N_2O_7Na]^+$ ($[M+Na]^+$) 451.1476, found 451.1477.

ATR-FTIR (cm^{-1}) 3302, 2952, 1729, 1650, 1519, 1345, 1108, 855, 738, 699.

4j: Dimethyl 2-(3-(allylamino)-2-(4-nitrophenyl)-3-oxopropyl)-2-methylmalonate

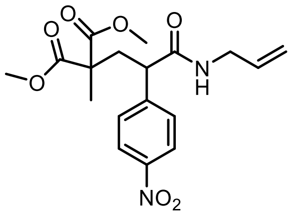

The product was synthesised according to **General Procedure B**, using *N*-allyl-*N*-((4-nitrophenyl)sulfonyl)acrylamide (29.6 mg, 0.100 mmol, 1.00 eq.) and dimethyl methylmalonate (21.9 mg, 0.150 mmol, 1.50 eq.), and obtained as a white solid (28.1 mg, 74%, 0.074 mmol). See NMR.

^{1}H NMR (600 MHz, CDCl$_3$) δ 8.16 (d, *J* = 8.5 Hz, 2H), 7.50 (d, *J* = 8.5 Hz, 2H), 5.74 (ddd, *J* = 24.1, 11.9, 6.4 Hz, 2H), 5.12 – 5.00 (m, 2H), 3.85 – 3.78 (m, 2H), 3.74 (dd, *J* = 7.1, 4.6 Hz, 1H),3.65 (s, 3H), 3.61 (s, 3H), 2.86 (dd, *J* = 14.5, 7.1 Hz, 1H), 2.21 (dd, *J* = 14.5, 4.6 Hz, 1H), 1.43 (s, 3H).

^{13}C NMR (151 MHz, CDCl$_3$) δ 172.4, 172.2, 171.3, 148.1, 147.3, 133.8, 129.0 (2C), 124.0 (2C), 116.9, 53.3 (2C), 52.7, 49.3, 42.4, 39.7, 21.4.

HRMS (ESI$^+$) *m/z* calculated for $[C_{18}H_{22}N_2O_7Na]^+$ ($[M+Na]^+$) 401.1319, found 401.1319.

ATR-FTIR (cm^{-1}) 3303, 2953, 1730, 1654, 1520, 1346, 1162, 855.

4k: Dimethyl 2-(3-((4,4-dimethoxybutyl)amino)-2-(4-nitrophenyl)-3-oxopropyl)-2-methylmalonate

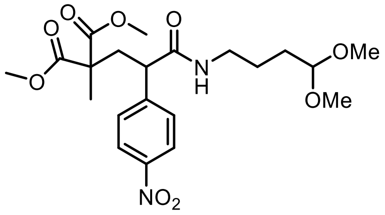

The product was synthesised according to **General Procedure B**, using *N*-(4,4-dimethoxybutyl)-*N*-((4-nitrophenyl)sulfonyl)acrylamide (37.2 mg, 0.100 mmol, 1.00 eq.) and dimethyl methylmalonate (21.9 mg, 0.150 mmol, 1.50 eq.), and obtained as a white solid (27.5 mg, 60%, 0.060 mmol). See NMR.

^{1}H NMR (400 MHz, CDCl$_3$) δ 8.23 – 8.09 (m, 2H), 7.49 (d, *J* = 8.8 Hz, 2H), 5.84 (t, *J* = 5.2 Hz, 1H), 4.28 (t, *J* = 5.2 Hz, 1H), 3.67 (dd, *J* = 7.2, 4.5 Hz, 1H), 3.63 (s, 3H), 3.61 (s, 3H), 3.27 (s, 3H), 3.25 (s, 3H), 3.22 – 3.15 (m, 2H), 2.89 – 2.80 (m, 1H), 2.20 (dd, *J* = 14.5, 4.5 Hz, 1H), 1.55 – 1.46 (m, 4H), 1.42 (s, 3H).

^{13}C NMR (101 MHz, CDCl$_3$) δ 172.4, 172.2, 171.4, 148.3, 147.2, 128.9 (2C), 124.0 (2C), 104.3, 53.3, 53.2, 53.1, 52.70, 52.67, 49.3, 39.7, 39.5, 29.9, 24.3, 21.2.

HRMS (ESI$^+$) *m/z* calculated for $[C_{21}H_{30}N_2O_9Na]^+$ ($[M+Na]^+$) 477.1844, found 477.1857.

ATR-FTIR (cm^{-1}) 3314, 2925, 2854, 1732, 1653, 1522, 1346, 1111, 856, 740.

4l: Dimethyl 2-methyl-2-(2-methyl-3-(methylamino)-2-(4-nitrophenyl)-3-oxopropyl)malonate

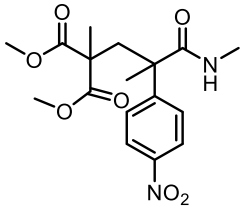

The product was synthesised according to **General Procedure B**, using *N*-methyl-*N*-((4-nitrophenyl)sulfonyl)methacrylamide (28.4 mg, 0.100 mmol, 1.00 eq.) and dimethyl methylmalonate (21.9 mg, 0.150 mmol, 1.50 eq.), and obtained as a yellow solid (14.3 mg, 39%, 0.039 mmol). See NMR.

^{1}H NMR (600 MHz, CDCl$_3$) δ 8.17 (d, *J* = 8.9 Hz, 2H), 7.59 (d, *J* = 8.9 Hz, 2H), 5.48 (d, *J* = 4.2 Hz, 1H), 3.68 (s, 3H), 3.66 (s, 3H), 3.06 (d, *J* = 14.9 Hz, 1H), 2.76 (d, *J* = 4.7 Hz, 3H), 2.65 (d, *J* = 14.9 Hz, 1H), 1.61 (s, 3H), 1.32 (s, 3H).

^{13}C NMR (151 MHz, CDCl$_3$) δ 174.7, 173.0, 172.5, 152.2, 147.0, 127.6 (2C), 123.8 (2C), 53.2, 52.9, 52.8, 49.8, 43.4, 27.1, 21.8, 21.6.

HRMS (ESI$^+$) *m/z* calculated for $[C_{17}H_{22}N_2NaO_7]^+$ ($[M+Na]^+$) 389.1320, found 389.1319.

ATR-FTIR (cm^{-1}): 3433, 3361, 2998, 2953, 1731, 1668, 1520, 1348, 1267, 1086, 856.

4m: Dimethyl 2-(2-(4-cyano-2-(trifluoromethyl)phenyl)-3-(methylamino)-3-oxopropyl)-2-methylmalonate

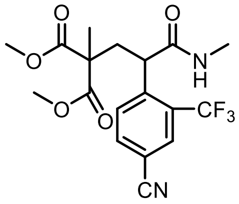

The product was synthesised according to **General procedure B**, using *N*-((4-cyano-2-(trifluoromethyl)phenyl)sulfonyl)-*N*-methylacrylamide (31.8 mg, 0.100 mmol, 1.00 eq.) and dimethyl methylmalonate (21.9 mg, 0.150 mmol, 1.50 eq.), and obtained as a white solid (48%, 19.2 mg, 0.048 mmol). See NMR.

^{1}H NMR (600 MHz, $CDCl_3$) δ 8.00 (d, *J* = 8.3 Hz, 1H), 7.90 (s, 1H), 7.82 (d, *J* = 8.2 Hz, 1H), 5.65 (d, *J* = 4.2 Hz, 1H), 3.86 (dd, *J* = 9.3, 2.3 Hz, 1H), 3.70 (s, 3H), 3.68 (s, 3H), 3.00 (dd, *J* = 14.4, 9.5 Hz, 1H), 2.74 (d, *J* = 4.8 Hz, 3H), 2.12 (dd, *J* = 14.4, 3.0 Hz, 1H), 1.41 (s, 3H).

^{13}C NMR (151 MHz, CDCl3) δ 172.1, 172.0, 170.9, 144.4, 135.7, 131.4, 129.7-129.5 (m), 128.5 (q, *J* = 30.6 Hz), 123.5 (q, *J* = 274.4 Hz), 122.6, 120.7, 53.3, 52.9, 52.8, 43.8, 39.4, 27.0, 20.4.

^{19}F NMR (565 MHz, $CDCl_3$) δ -58.6.

HRMS (ESI$^+$) *m/z* calculated for $[C_{18}H_{19}F_3N_2NaO_5]^+$ ($[M+Na]^+$) 423.1138, found 423.1138.

ATR-FTIR (cm^{-1}) 3403, 3001, 2955, 2235, 1730, 1674, 1529, 1317, 1057, 666.

4n: Dimethyl 2-(2-(4-cyanophenyl)-3-(methylamino)-3-oxopropyl)-2-methylmalonate

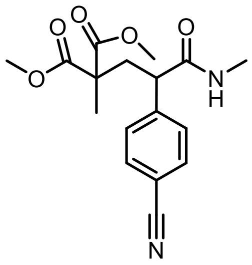

The product was synthesised according to **General Procedure C**, using *N*-((4-cyanophenyl)sulfonyl)-*N*-methylacrylamide (31.8 mg, 0.100 mmol, 1.00 eq.) and dimethyl methylmalonate (21.9 mg, 0.150 mmol, 1.50 eq.), and obtained as a white solid (19.2 mg, 48%, 0.048 mmol). See NMR.

^{1}H NMR (600 MHz, $CDCl_3$) δ 7.60 (d, *J* = 8.3 Hz, 2H), 7.44 (d, *J* = 8.3 Hz, 2H), 5.55 (d, *J* = 4.5 Hz, 1H), 3.65-3.62 (m, 4H), 3.61 (s, 3H), 2.83 (dd, *J* = 14.5, 7.2 Hz, 1H), 2.75 (d, *J* = 4.5 Hz, 3H), 2.19 (dd, *J* = 14.5, 4.7 Hz, 1H), 1.42 (s, 3H).

^{13}C NMR (151 MHz, $CDCl_3$) δ 172.4, 172.2, 172.2, 146.0, 132.6 (2C), 128.9 (2C), 118.8, 111.3, 53.3, 52.7, 52.7, 49.5, 39.6, 26.8, 21.4.

HRMS (ESI$^+$) *m/z* calculated for $[C_{17}H_{20}N_2NaO_5]^+$ ($[M+Na]^+$) 355.1264, found 355.1264.

ATR-FTIR (cm^{-1}) 3318, 2923, 2852, 2228, 1732, 1652, 1606, 1537, 1503, 1458, 1413, 1379, 1267, 1111, 1020, 738.

4o: Dimethyl 2-methyl-2-(3-(methylamino)-3-oxo-2-(5-(trifluoromethyl)pyridin-2-yl)propyl)malonate

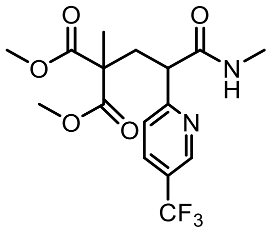

The product was synthesised according to **General Procedure B**, using *N*-methyl-*N*-((5-(trifluoromethyl)pyridin-2-yl)sulfonyl)acrylamide (29.4 mg, 0.100 mmol, 1.00 eq.) and dimethyl methylmalonate (21.9 mg, 0.150 mmol, 1.50 eq.), and obtained as a colourless solid (26.5 mg, 70%, 0.070 mmol). See NMR.

^{1}H NMR (600 MHz, CDCl$_3$) δ 8.79 (d, *J* = 0.5 Hz, 1H), 7.88 (dd, *J* = 8.2, 2.2 Hz, 1H), 7.46 (d, *J* = 8.2 Hz, 1H), 6.39 (d, *J* = 4.2 Hz, 1H), 3.87 (t, *J* = 6.2 Hz, 1H), 3.63 (s, 3H), 3.57 (s, 3H), 2.79 (dd, *J* = 14.4, 6.4 Hz, 1H), 2.75 (d, *J* = 4.9 Hz, 3H), 2.57 (dd, *J* = 14.4, 6.4 Hz, 1H), 1.41 (s, 3H).

^{13}C NMR (151 MHz, CDCl$_3$) δ 172.3, 172.1, 171.6, 163.4, 146.2 (q, *J* = 4.0 Hz), 134.2 (q, *J* = 3.4 Hz), 125.5 (q, *J* = 33.2 Hz), 123.6 (q, *J* = 272.3 Hz) 123.1, 53.3, 52.8, 52.6, 52.0, 38.4, 26.7, 20.7.

^{19}F NMR (376 MHz, CDCl$_3$) δ -62.39.

HRMS (ESI$^+$) *m/z* calculated for $[C_{16}H_{19}F_3N_2NaO_5]^+$ ($[M+Na]^+$) 399.1138, found 399.1142.

ATR-FTIR (cm^{-1}) 3301, 2954, 1732, 1654, 1328, 1163, 1122, 1081.

4p: Dimethyl (*E*)-2-methyl-2-(2-(methylcarbamoyl)-4-(4-nitrophenyl)but-3-en-1-yl)malonate (XX)

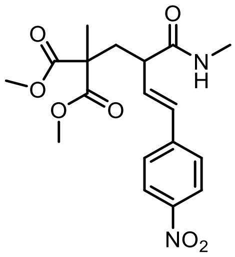

The product was synthesised according to **General Procedure B**, using (*E*)-*N*-methyl-*N*-((4-nitrostyryl)sulfonyl)acrylamide (29.6 mg, 0.100 mmol, 1.00 eq.) and dimethyl methylmalonate (21.9 mg, 0.150 mmol, 1.50 eq.), and obtained as a yellow oil (34.5 mg, 91%, 0.091 mmol). See NMR.

^{1}H NMR (600 MHz, CDCl$_3$) δ 8.14 (d, *J* = 8.8 Hz, 2H), 7.46 (d, *J* = 8.8 Hz, 2H), 6.49 (d, *J* = 15.9 Hz, 1H), 6.37 (dd, *J* = 15.9, 9.0 Hz, 1H), 5.83 (q, *J* = 4.5 Hz, 1H), 3.64 (s, 3H), 3.63 (s, 3H), 3.21 (dt, *J* = 9.0, 6.2 Hz, 1H), 2.80 (d, *J* = 4.5 Hz, 3H), 2.58 (dd, *J* = 14.5, 6.2 Hz, 1H), 2.20 (dd, *J* = 14.5, 6.2 Hz, 1H), 1.45 (s, 3H).

^{13}C NMR (151 MHz, CDCl$_3$) δ 172.7, 172.5, 172.4, 147.1, 143.1, 133.9, 130.3, 127.0 (2C), 124.2 (2C), 53.0, 52.8, 52.7, 47.4, 38.0, 26.7, 21.1.

HRMS (ESI$^+$) *m/z* calculated for $[C_{18}H_{22}N_2O_7Na]^+$ ($[M+Na]^+$) 401.1319, found 401.1319.

ATR-FTIR (cm^{-1}) 3403, 3302, 3084, 2997, 2952, 1731, 1645, 1517, 1343, 1265, 1111, 976, 863, 746.

4q: Dimethyl (*E*)-2-methyl-2-(2-(methylcarbamoyl)-4-phenylbut-3-en-1-yl)malonate

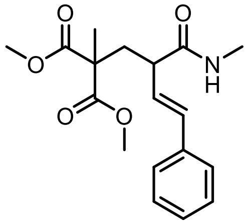

The product was synthesised according to **General Procedure B**, using (*E*)-*N*-methyl-2-phenylethene-1-sulfonamide (25.1 mg, 0.10 mmol, 1.00 eq.) and dimethyl methylmalonate (21.9 mg, 0.150 mmol, 1.50 eq.), and obtained as a yellow oil (8.8 mg, 26%, 0.026 mmol). See NMR.

^{1}H NMR (700 MHz, CDCl$_3$) δ 7.37 – 7.33 (m, 2H), 7.30 (dd, *J* = 10.5, 4.9 Hz, 2H), 7.25 – 7.21 (m, 1H), 6.43 (d, *J* = 15.9 Hz, 1H), 6.13 (dd, *J* = 15.9, 9.4 Hz, 1H), 5.69 (app s, 1H), 3.66 (s, 3H), 3.60 (s, 3H), 3.14 (ddd, *J* = 9.2, 7.4, 5.3 Hz, 1H), 2.79 (d, *J* = 4.9 Hz, 3H), 2.64 (dd, *J* = 14.5, 5.2 Hz, 1H), 2.21 (dd, *J* = 14.5, 7.4 Hz, 1H), 1.47 (s, 3H).

^{13}C NMR (176 MHz, CDCl$_3$) δ 173.2, 172.7, 172.4, 136.5, 133.0, 128.8 (2C), 128.5, 128.0, 126.5 (2C), 53.0, 52.8, 52.7, 47.3, 37.7, 26.7, 20.9.

HRMS (ESI) m/z calculated for $[M+Na]^+$ $[C_{18}H_{23}NNaO_5]^+$($[M+Na]^+$) 356.1467, found 356.1461.

ATR-FTIR (cm^{-1}) 3391, 3060, 3027, 2999, 2952, 1729, 1642, 1262, 1200, 1160, 1112, 1028, 736, 694.

4r: Dimethyl (*E*)-2-methyl-2-(2-(methylcarbamoyl)-4-(naphthalen-2-yl)but-3-en-1-yl)malonate

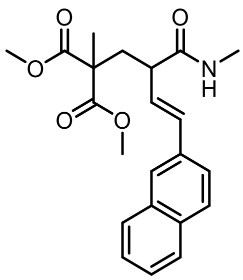

The product was synthesised according to **General Procedure B**, using (*E*)-*N*-methyl-*N*-((2-(naphthalen-2-yl)vinyl)sulfonyl)acrylamide (30.1 mg, 0.100 mmol, 1.00 eq.) and dimethyl methylmalonate (21.9 mg, 0.150 mmol, 1.50 eq.), and obtained as a yellow oil (15.2 mg, 40%, 0.040 mmol). See NMR.

^{1}H NMR (700 MHz, CDCl$_3$) δ 7.81 – 7.75 (m, *J* = 10.0, 9.3 Hz, 3H), 7.70 (s, 1H), 7.56 (dd, *J* = 8.5, 1.7 Hz, 1H), 7.49 – 7.39 (m, *J* = 7.7, 5.4, 1.2 Hz, 2H), 6.59 (d, *J* = 15.8 Hz, 1H), 6.26 (dd, *J* = 15.8, 9.3 Hz, 1H), 5.76 (d, *J* = 4.0 Hz, 1H), 3.66 (s, 3H), 3.59 (s, 3H), 3.20 (ddd, *J* = 9.0, 7.7, 5.1 Hz, 1H), 2.81 (d, *J* = 4.9 Hz, 3H), 2.66 (dd, *J* = 14.5, 5.1 Hz, 1H), 2.26 (dd, *J* = 14.5, 7.5 Hz, 1H), 1.49 (s, 3H).

^{13}C NMR (176 MHz, CDCl$_3$) δ 173.2, 172.7, 172.4, 134.0, 133.7, 133.2, 133.1, 128.9, 128.5, 128.1, 127.8, 126.6, 126.5, 126.2, 123.4, 53.0, 52.8, 52.7, 47.4, 37.8, 26.7, 21.0.

HRMS (ESI) m/z calculated for [M+Na]$^+$ [$C_{22}H_{25}NNaO_5$]$^+$ ([M+Na]$^+$) 406.1625, found 406.1617.

ATR-FTIR (cm^{-1}) 3297, 3056, 2998, 2952, 1731, 1645, 1557, 1264, 1201, 1115, 743

5a: 3-(diphenylphosphoryl)-*N*-methyl-2-(4-nitrophenyl)propanamide

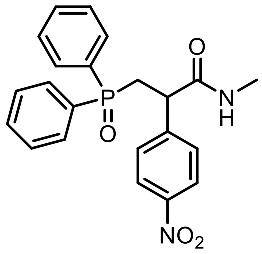

The product was synthesised according to **General Procedure B**, using *N*-methyl-*N*-((4-nitrophenyl)sulfonyl)acrylamide (27.0 mg, 0.100 mmol, 1.00 eq.) and diphenylphosphine oxide (30.3 mg, 0.150 mmol, 1.50 eq.), and obtained as a white solid (71%, 29.1 mg, 0.071 mmol). See NMR.

^{1}H NMR (600 MHz, CDCl$_3$) δ 7.97 (d, *J* = 8.7 Hz, 2H), 7.79 – 7.72 (m, 2H), 7.56 – 7.51 (m, 3H) , 7.51 (d, *J* = 8.7 Hz, 2H), 7.50 – 7.46 (m, 2H), 7.42 (td, *J* = 7.5, 1.1 Hz, 1H), 7.32 (td, *J* = 7.7, 2.8 Hz, 2H), 7.15 (q, *J* = 4.2 Hz, 1H), 4.39 (dt, *J* = 11.6, 6.8 Hz, 1H), 3.32 (ddd, *J* = 15.4, 10.4, 7.3 Hz, 1H), 2.77 – 2.71 (m, 1H), 2.52 (d, *J* = 4.8 Hz, 3H).

^{13}C NMR (151 MHz, CDCl$_3$) δ 171.4 (d, *J* = 7.5 Hz), 147.2, 147.0 (d, *J* = 8.2 Hz), 132.4 (d, *J* = 99.9 Hz), 132.4 (d, *J* = 100.2 Hz), 132.3 (d, *J* = 2.5 Hz), 132.0 (d, *J* = 3.0 Hz), 130.8 (d, *J* = 9.8 Hz), 130.6 (d, *J* = 9.7 Hz), 129.4, 128.9 (d, *J* = 12.1 Hz), 128.7 (d, *J* = 11.6 Hz), 123.7, 45.4 (d, *J* = 1.6 Hz), 33.8 (d, *J* = 70.0 Hz), 26.5.

^{31}P NMR (243 MHz, CDCl$_3$) δ 30.43.

HRMS (ESI$^+$) *m/z* calculated for [$C_{22}H_{21}N_2O_4NaP$]$^+$ ([M+Na]$^+$) 431.1131, found 431.1127.

ATR-FTIR (cm^{-1}) 3427, 3267, 3078, 1666, 1519, 1346, 1176, 695.

5b: *N*-methyl-2-(4-nitrophenyl)-3-(octylthio)propanamide

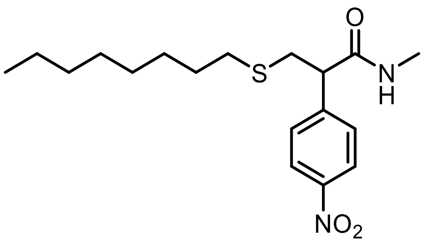

The product was synthesised according to **General Procedure B**, using *N*-methyl-*N*-((4-nitrophenyl)sulfonyl)acrylamide (27.0 mg, 0.100 mmol, 1.00 eq.) and 1-octanethiol (21.9 mg, 0.150 mmol, 1.5 eq.), and obtained as a white solid (85%, 30.1 mg, 0.085 mmol). See NMR.

^{1}H NMR (700 MHz, CDCl$_3$) δ 8.18 (d, *J* = 8.8 Hz, 2H), 7.54 (d, *J* = 8.7 Hz, 2H), 5.68 (br s, 1H), 3.59 (t, *J* = 7.4 Hz, 1H), 3.29 (dd, *J* = 13.1, 8.1 Hz, 1H), 2.87 (dd, *J* = 13.1, 6.8 Hz, 1H), 2.81 (d, *J* = 4.9 Hz, 3H), 2.52 – 2.44 (m, 2H), 1.56 – 1.45 (m, 2H), 1.36 – 1.19 (m, 10H), 0.87 (t, *J* = 7.1 Hz, 3H).

^{13}C NMR (176 MHz, CDCl$_3$) δ 171.4, 147.5, 146.3, 129.0 (2C), 124.1 (2C), 53.9, 35.8, 33.3, 31.9, 29.7, 29.3, 29.3, 28.9, 26.8, 22.8, 14.2.

HRMS (ESI$^+$) *m/z* calculated for $[C_{18}H_{28}N_2NaO_3S]^+$ ($[M+Na]^+$) 375.1713, found 375.1714.

ATR-FTIR (cm^{-1}) 3407, 3295, 3081, 2953, 2924, 2854, 1649, 1520, 1345, 855, 695.

5c: 3-((4-chlorophenyl)thio)-*N*-methyl-2-(4-nitrophenyl)propanamide

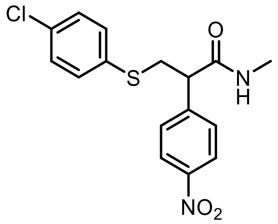

The product was synthesised according to **General Procedure B**, using *N*-methyl-*N*-((4-nitrophenyl)sulfonyl)acrylamide (27.0 mg, 0.100 mmol, 1.00 eq.) and 4-chlorobenzenethiol (21.7 mg, 0.150 mmol, 1.50 eq.), and obtained as a white solid (49%, 17.0 mg, 0.049 mmol). See NMR.

^{1}H NMR (600 MHz, CDCl$_3$) δ 8.17 (d, *J* = 8.6 Hz, 2H), 7.48 (d, *J* = 8.6 Hz, 2H), 7.28 – 7.23 (m, 4H), 5.53 (q, *J* = 3.3 Hz, 1H), 3.70 (dd, *J* = 13.5, 7.8 Hz, 1H), 3.57 (t, *J* = 7.4 Hz, 1H), 3.23 (dd, *J* = 13.5, 6.9 Hz, 1H), 2.78 (d, *J* = 4.8 Hz, 3H).

^{13}C NMR (151 MHz, CDCl$_3$) δ 170.8, 147.7, 145.6, 133.7, 133.0, 131.4 (2C), 129.4 (2C), 129.0 (2C), 124.2 (2C), 52.6, 37.5, 26.8.

HRMS (ESI$^+$) *m/z* calculated for $[C_{16}H_{15}N_2NaO_3SCl]^+$ ($[M+Na]^+$) 373.0385, found 373.0381.

ATR-FTIR (cm^{-1}) 3403, 3307, 1651, 1519, 1347, 1110, 1012, 1084, 856.

5d: *N*-methyl-2-(4-nitrophenyl)-3-(phenylsulfonyl)propanamide

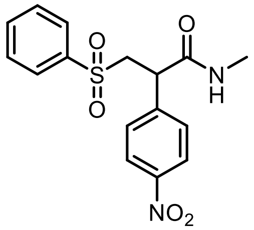

The product was synthesised according to **General Procedure B** without BTMG, using *N*-methyl-*N*-((4-nitrophenyl)sulfonyl)acrylamide (27.0 mg, 0.100 mmol, 1.00 eq.) and sodium benzenesulfinate (24.6 mg, 0.150 mmol, 1.50 eq.), and obtained as a pale yellow solid (70%, 24.2 mg, 0.070 mmol). See NMR.

^{1}H NMR (600 MHz, CDCl$_3$) δ 8.09 – 8.02 (m, 2H), 7.81 – 7.74 (m, 2H), 7.61 – 7.54 (m, 1H), 7.45 (ddt, *J* = 16.7, 9.3, 2.0 Hz, 4H), 5.79 (d, *J* = 4.6 Hz, 1H), 4.23 – 4.12 (m, 2H), 3.35 (dt, *J* = 10.7, 8.1 Hz, 1H), 2.66 (d, *J* = 4.6 Hz, 3H).

^{13}C NMR (151 MHz, CDCl$_3$) δ 169.6, 147.8, 144.5, 139.2, 134.3, 129.5 (2C), 129.1 (2C), 128.1 (2C), 124.3 (2C), 58.8, 46.6, 27.1.

HRMS (ESI$^+$) *m/z* calculated for $[C_{16}H_{16}N_2NaO_5S]^+$ ($[M+Na]^+$) 371.0672, found 371.0667.

ATR-FTIR (cm^{-1}) 3318, 3095, 2956, 1684, 1521, 1348, 736.

5e: *N*,2-dimethyl-2-(4-nitrophenyl)-3-(phenylsulfonyl)propanamide

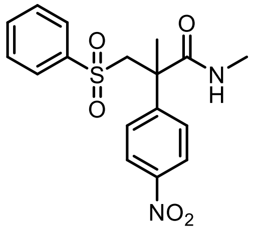

The product was synthesised according to **General Procedure B** without BTMG, using *N*-methyl-*N*-(4-nitrophenyl)methacrylamide (28.4 mg, 0.100 mmol, 1.00 eq.) and sodium benzenesulfinate (24.6 mg, 0.150 mmol, 1.50 eq.), and obtained as a colourless solid (86%, 31.3 mg, 0.086 mmol). See NMR. See X-ray.

^{1}H NMR (700 MHz, CDCl$_3$) 8.15 – 8.06 (m, 2H), 7.75 – 7.67 (m, 2H), 7.58 (t, *J* = 7.5 Hz, 1H), 7.51 – 7.48 (m, 2H), 7.46 – 7.42 (m, 2H), 5.39 (d, *J* = 3.4 Hz, 1H), 4.09 (d, *J* = 14.7 Hz, 1H), 3.78 (d, *J* = 14.7 Hz, 1H), 2.78 (d, *J* = 4.8 Hz, 3H), 2.08 (s, 3H).

^{13}C NMR (176 MHz, CDCl$_3$) δ 173.5, 147.7, 141.0, 133.7, 129.3 (2C), 128.1 (2C), 127.7 (2C), 123.9 (2C), 64.0, 49.7, 27.3, 22.5.

HRMS (ESI$^+$) *m/z* calculated for $[C_{17}H_{18}N_2NaO_5S]^+$ ($[M+Na]^+$) 385.0829, found 385.0826.

ATR-FTIR (cm^{-1}) 3366, 2921, 1660, 1529, 1316, 1135, 844, 549.

5f: *N*-methyl-3-((*N*-methyl-4-nitrophenyl)sulfonamido)-2-(4-nitrophenyl)propanamide

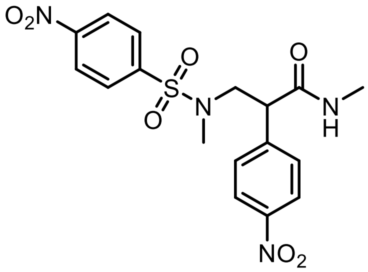

The product was synthesised according to **General Procedure B**, using *N*-methyl-*N*-((4-nitrophenyl)sulfonyl)acrylamide (27.0 mg, 0.100 mmol, 1.00 eq.) and *N*-methyl-4-nitrobenzenesulfonamide (32.4 mg, 0.150 mmol, 1.50 eq.), and obtained as a white solid (75%, 31.8 mg, 0.075 mmol). See NMR.

^{1}H NMR (600 MHz, $CDCl_3$) δ 8.37 (d, *J* = 8.7 Hz, 2H), 8.22 (d, *J* = 8.7 Hz, 2H), 7.95 (d, *J* = 8.7 Hz, 2H), 7.59 (d, *J* = 8.7 Hz, 2H), 5.67 (d, *J* = 4.6 Hz, 1H), 3.99 (dd, *J* = 8.4, 6.4 Hz, 1H), 3.58 (dd, *J* = 14.3, 8.4 Hz, 1H), 3.40 (dd, *J* = 14.3, 6.4 Hz, 1H), 2.84 (d, *J* = 4.6 Hz, 3H), 2.77 (s, 3H).

^{13}C NMR (151 MHZ, $CDCl_3$) δ 170.6, 150.4, 147.9, 144.0, 142.8, 129.3 (2C), 128.7 (2C), 124.7 (2C), 124.4 (2C), 54.2, 54.0, 37.8, 26.9.

HRMS (ESI$^+$) *m/z* calculated for $[C_{17}H_{18}N_4NaO_7S]^+$ ($[M+Na]^+$) 445.0788, found 445.0785.

ATR-FTIR (cm^{-1}) 3274, 3109, 2923, 2859, 1645, 1516, 1167, 856, 742, 600.

5g: 3-(diallylamino)-*N*-methyl-2-(4-nitrophenyl)propanamide

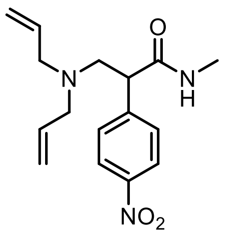

The product was synthesised according to **General Procedure B**, using *N*-methyl-*N*-((4-nitrophenyl)sulfonyl)acrylamide (27.0 mg, 0.100 mmol, 1.00 eq.) and diallylamine (14.9 mg, 0.150 mmol, 1.50 eq.), and obtained as a yellow oil (74%, 22.5 mg, 0.074 mmol). See NMR.

^{1}H NMR (600 MHz, $CDCl_3$) δ 8.15 (d, *J* = 8.6 Hz, 2H), 7.40 (d, *J* = 8.6 Hz, 2H), 7.37 (br s, 1H), 5.83 – 5.74 (m, 2H), 5.25 – 5.14 (m, 4H), 3.71 (dd, *J* = 10.2, 5.0 Hz, 1H), 3.24 (dd, *J* = 14.1, 6.1 Hz, 2H), 3.13 (dd, *J* = 13.1, 10.2 Hz, 1H), 3.07 (dd, *J* = 14.1, 7.0 Hz, 2H), 2.81 (d, *J* = 4.8 Hz, 3H), 2.64 (dd, *J* = 13.1, 5.0 Hz, 1H).

^{13}C NMR (151 MHz, $CDCl_3$) δ 172.3, 147.2, 146.7, 134.6 (2C), 129.7 (2C), 123.8 (2C), 118.7 (2C), 57.2 (2C), 56.9, 50.3, 26.3.

HRMS (ESI$^+$) *m/z* calculated for $[C_{16}H_{22}N_3O_3]^+$ ($[M+H]^+$) 304.1656, found 304.1657.

ATR-FTIR (cm^{-1}) 3304, 3078, 2926, 2809, 1646, 1519, 1345, 996, 855, 741, 533.

5h: 3-(benzyl(methyl)amino)-*N*-methyl-2-(4-nitrophenyl)propanamide

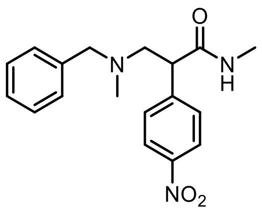

The product was synthesised according to **General Procedure B**, using *N*-methyl-*N*-((4-nitrophenyl)sulfonyl)acrylamide (27.0 mg, 0.100 mmol, 1.00 eq.) and *N*-benzylmethylamine (18.2 mg, 0.150 mmol, 1.50 eq.), and obtained as a pale yellow solid (73%, 24.0 mg, 0.073 mmol). See NMR.

^{1}H NMR (600 MHz, $CDCl_3$) δ 8.20 – 8.13 (m, 2H), 7.56 (s, 1H), 7.39 – 7.30 (m, 5H), 7.26 – 7.21 (m, 2H), 3.72 (dd, *J* = 10.1, 5.1 Hz, 1H), 3.65 (d, *J* = 13.0 Hz, 1H), 3.53 (d, *J* = 13.0 Hz, 1H), 3.13 (dd, *J* = 12.8, 10.1 Hz, 1H), 2.82 (d, *J* = 4.8 Hz, 3H), 2.62 (dd, *J* = 12.8, 5.2 Hz, 1H), 2.33 (s, 3H).

^{13}C NMR (151 MHz, $CDCl_3$) δ 172.2, 147.2, 146.6, 138.0, 129.7 (2C), 129.2 (2C), 128.6 (2C), 127.7, 123.8 (2C), 62.7, 60.5, 49.9, 42.4, 26.3.

HRMS (ESI$^+$) *m/z* calculated for $[C_{18}H_{21}N_3NaO_3]^+$ ($[M+Na]^+$) 350.1475, found 350.1473.

ATR-FTIR (cm^{-1}) 3335, 2975, 1729, 1651, 1522, 1347, 1050, 881, 739.

5i: *N*-methyl-3-(methyl(phenyl)amino)-2-(4-nitrophenyl)propanamide

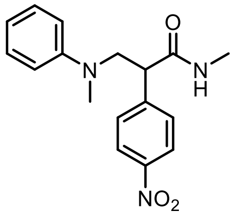

The product was synthesised according to **General Procedure B**, using *N*-methyl-*N*-((4-nitrophenyl)sulfonyl)acrylamide (27.0 mg, 0.100 mmol, 1.00 eq.) and *N*-methylaniline (16.1 mg, 0.150 mmol, 1.50 eq.), and obtained as an orange solid (54%, 17.2 mg, 0.054 mmol). See NMR.

^{1}H NMR (600 MHz, CDCl$_3$) δ 8.18 (d, *J* = 8.6 Hz, 2H), 7.57 (d, *J* = 8.6 Hz, 2H), 7.33 – 7.19 (m, 2H), 6.75 (t, *J* = 7.2 Hz, 1H), 6.68 (d, *J* = 8.3 Hz, 2H), 5.63 (d, *J* = 3.0 Hz, 1H), 4.12 (dd, *J* = 14.8, 8.4 Hz, 1H), 3.82 (dd, *J* = 8.4, 5.4 Hz, 1H), 3.67 (dd, *J* = 14.8, 5.4 Hz, 1H), 2.86 (s, 3H), 2.77 (d, *J* = 4.8 Hz, 3H).

^{13}C NMR (151 MHz, CDCl$_3$) δ 171.5, 148.1, 147.5, 145.5, 129.6 (2C), 129.1 (2C), 124.0 (2C), 117.1, 112.2 (2C), 56.9, 50.9, 39.7, 26.8.

HRMS (ESI$^+$) *m/z* calculated for $[C_{17}H_{19}N_3NaO_3]^+$ ($[M+Na]^+$) 336.1319, found 336.1313.

ATR-FTIR (cm^{-1}) 3394, 3301, 2939, 1649, 1598, 1518, 1506, 1345, 956, 748, 693.

5j: 3-((3a*R*,6*S*,7a*S*)-8,8-dimethyl-2,2-dioxidotetrahydro-3*H*-3a,6-methanobenzo[*c*]isothiazol-1(4*H*)-yl)-*N*-methyl-2-(4-nitrophenyl)propanamide

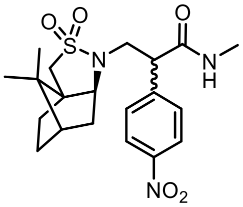

The product was synthesised according to **General Procedure B**, using *N*-methyl-*N*-((4-nitrophenyl)sulfonyl)acrylamide (27.0 mg, 0.100 mmol, 1.00 eq.) and (+)-10,2-camphorsultam (32.3 mg, 0.150 mmol, 1.50 eq.), and obtained as two separable diasteromers.

The minor diasteromer (**5j-A**) was obtained as a white solid (29%, 12.3 mg, 0.029 mmol). See NMR.

^{1}H NMR (600 MHz, CDCl$_3$) δ 8.16 (d, *J* = 8.8 Hz, 2H), 7.62 (d, *J* = 8.7 Hz, 2H), 6.04 (q, *J* = 4.6 Hz, 1H), 4.10 (dd, *J* = 12.6, 3.8 Hz, 1H), 3.42 (t, *J* = 12.7 Hz, 1H), 3.22 (dd, *J* = 12.7, 3.9 Hz, 1H), 3.23 – 3.17 (m, 2H), 3.01 (dd, *J* = 7.9, 4.4 Hz, 1H), 2.74 (d, *J* = 4.9 Hz, 3H), 2.16 (ddd, *J* = 12.4, 6.6, 3.8 Hz, 1H), 1.94 (t, *J* = 3.8 Hz, 1H), 1.90 – 1.81 (m, 2H), 1.66 (dd, *J* = 12.6, 7.8 Hz, 1H), 1.42 – 1.35 (m, 1H), 1.29 – 1.22 (m, 1H), 1.11 (s, 3H), 0.92 (s, 3H).

^{13}C NMR (151 MHz, CDCl$_3$) δ 171.4, 147.6, 144.0, 129.2 (2C), 123.9 (2C), 68.4, 50.9, 50.8, 50.3, 47.7, 47.1, 44.6, 35.3, 32.2, 27.1, 26.7, 20.1, 20.1.

HRMS (ESI$^+$) *m/z* calculated for $[C_{20}H_{27}N_3NaO_5]^+$ ($[M+Na]^+$) 444.1564, found 444.1567.

ATR-FTIR (cm^{-1}) 3386, 2956, 2882, 1661, 1521, 1347, 1301, 1055, 737.

The major diasteromer (**5j-B**) was obtained as a white solid (40%, 17.0 mg, 0.040 mmol). See NMR.

^{1}H NMR (600 MHz, CDCl$_3$) δ 8.15 (d, *J* = 8.7 Hz, 1H), 7.66 (d, *J* = 8.7 Hz, 2H), 5.81 (q, *J* = 4.1 Hz, 1H), 4.11 (dd, *J* = 11.1, 5.0 Hz, 1H), 3.65 (dd, *J* = 14.0, 5.0 Hz, 1H), 3.28 (dd, *J* = 14.0, 11.2 Hz, 2H), 3.06 (dd, *J* = 47.4, 13.8 Hz, 2H), 2.99 (dd, *J* = 7.8, 4.6 Hz, 1H), 2.77 (d, *J* = 4.8 Hz, 3H), 1.82 – 1.74 (m, 1H), 1.72 (t, *J* = 3.7 Hz, 1H), 1.56 (dd, *J* = 12.6, 7.9 Hz, 1H), 1.42 – 1.36 (m, 1H), 1.34 (dd, *J* = 11.4, 6.8 Hz, 1H), 1.19 (dd, *J* = 9.7, 7.8 Hz, 1H), 0.77 (s, 3H), 0.39 (s, 3H).

^{13}C NMR (151 MHz, CDCl$_3$) δ 170.7, 147.5, 145.8, 129.7 (2C), 123.7 (2C), 67.8, 50.3, 50.2, 50.0, 47.3, 46.8, 44.3, 35.1, 32.2, 26.9, 26.7, 19.9, 19.5.

HRMS (ESI$^+$) *m/z* calculated for $[C_{20}H_{27}N_3NaO_5]^+$ ($[M+Na]^+$) 444.1564, found 444.1568.

ATR-FTIR (cm^{-1}) 3368, 2959, 2882, 1658, 1520, 1347, 1303, 1054, 737.

5k: *N*-methyl-3-morpholino-2-(4-nitrophenyl)propenamide

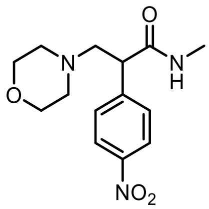

The product was synthesised according to **General procedure B**, using *N*-methyl-*N*-((4-nitrophenyl)sulfonyl)acrylamide (27.0 mg, 0.100 mmol, 1.00 eq.) and morpholine (13.1 mg, 0.150 mmol, 1.50 eq.), and obtained as a white solid (57%, 16.8 mg, 0.057 mmol). See NMR.

^{1}H NMR (700 MHz, CDCl$_3$) δ 8.17 (d, *J* = 8.7 Hz, 2H), 7.42 (d, *J* = 8.7 Hz, 2H), 3.76 – 3.64 (m, 5H), 3.09 (dd, *J* = 12.9, 10.1 Hz, 1H), 2.83 (d, *J* = 4.8 Hz, 3H), 2.65 – 2.54 (m, 3H), 2.54 – 2.40 (m, 2H).

^{13}C NMR (176 MHz, CDCl$_3$) δ 172.0, 147.3, 146.4, 129.6 (2C), 123.9 (2C), 67.1 (2C), 61.8, 53.7 (2C), 49.3, 26.5.

HRMS (ESI) m/z calculated for $[C_{14}H_{20}N_3O_4]^+$($[M+H]^+$) 294.1448, found 294.1441.

ATR-FTIR (cm^{-1}) 3304, 3079, 2953, 2854, 2810, 1648, 1516, 1344, 1112, 856, 734, 699.

5l: 3-(1*H*-benzo[*d*][1,2,3]triazol-1-yl)-*N*-methyl-2-(4-nitrophenyl)propanamide

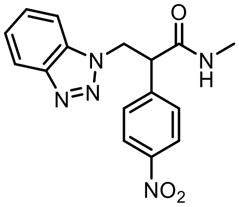

The product was synthesised according to **General Procedure B**, using *N*-methyl-*N*-((4-nitrophenyl)sulfonyl)acrylamide (27.0 mg, 0.100 mmol, 1.00 eq.) and 1*H*-benzotriazole (17.9 mg, 0.150 mmol, 1.50 eq.), and obtained as a white solid (52%, 16.9 mg, 0.052 mmol). See NMR.

^{1}H NMR (400 MHz, CDCl$_3$) δ 8.15 (d, *J* = 8.7 Hz, 2H), 7.98 (d, *J* = 8.4 Hz, 1H), 7.59 (d, *J* = 8.7 Hz, 2H), 7.52 – 7.42 (m, 2H), 7.34 (dd, *J* = 11.1, 3.9 Hz, 1H), 5.85 (s, 1H), 5.34 (dd, *J* = 13.9, 8.3 Hz, 1H), 4.89 (dd, *J* = 13.9, 6.5 Hz, 1H), 4.54 – 4.39 (m, 1H), 2.69 (d, *J* = 4.8 Hz, 3H).

^{13}C NMR (176 MHz, CDCl$_3$) δ 169.9, 148.0, 145.7, 143.4, 133.5, 129.2 (2C), 128.0, 124.4 (2C), 124.4, 119.9, 109.70, 53.04, 50.4, 26.9.

HRMS (ESI$^+$) *m/z* calculated for $[C_{16}H_{15}N_5NaO_3]^+$ ($[M+Na]^+$) 348.1067, found 348.1067.

ATR-FTIR (cm^{-1}) 3299, 3070, 2948, 1676, 1346, 745.

5m: 3-(benzylamino)-*N*-methyl-2-(4-nitrophenyl)propanamide

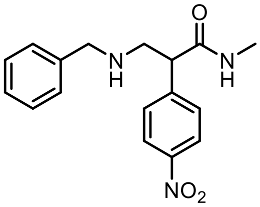

The product was synthesised according to **General Procedure B**, using *N*-methyl-*N*-((4-nitrophenyl)sulfonyl)acrylamide (27.0 mg, 0.100 mmol, 1.00 eq.) and benzylamine (16.1 mg, 0.150 mmol, 1.50 eq.), and obtained as a white solid (41%, 12.8 mg, 0.041 mmol). See NMR.

^{1}H NMR (700 MHz, CDCl$_3$) δ 8.20 – 8.13 (m, 2H), 7.46 – 7.40 (m, 2H), 7.35 – 7.30 (m, 2H), 7.29 – 7.26 (m, 2H), 7.25 (m, 1H), 6.86 (s, 1H), 3.80 (d, *J* = 2.6 Hz, 2H), 3.69 (dd, *J* = 8.2, 4.8 Hz, 1H), 3.26 (dd, *J* = 12.2, 8.2 Hz, 1H), 2.96 (dd, *J* = 12.2, 4.8 Hz, 1H), 2.81 (d, *J* = 4.8 Hz, 3H), 1.77 (s, 1H).

^{13}C NMR (176 MHz, CDCl$_3$) δ 172.1, 147.4, 146.0, 139.4, 129.3 (2C), 128.8 (2C), 128.3 (2C), 127.5, 124.1 (2C), 54.0, 52.8, 52.0, 26.5.

HRMS (ESI$^+$) *m/z* calculated for $[C_{17}H_{20}N_3O_3]^+$ ($[M+H]^+$) 314.1499, found 314.1506.

ATR-FTIR (cm^{-1}) 3408, 2981, 1653, 1518, 1347, 1051, 737, 648.

5n: 3-((2,3-dihydro-1*H*-inden-2-yl)amino)-*N*-methyl-2-(4-nitrophenyl)propenamide

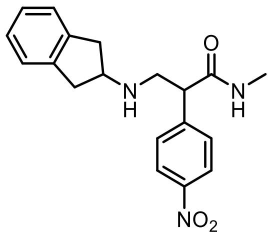

The product was synthesised according to **General procedure B**, using *N*-methyl-*N*-((4-nitrophenyl)sulfonyl)acrylamide (27.0 mg, 0.100 mmol, 1.00 eq.) and 2,3-dihydro-1*H*-inden-2-amine (21.9 mg, 0.150 mmol, 1.50 eq.), and obtained as a white solid (40%, 13.7 mg, 0.040 mmol). See NMR.

^{1}H NMR (600 MHz, CDCl$_3$) δ 8.24 – 8.16 (m, 2H), 7.51 – 7.42 (m, 2H), 7.19 (dd, *J* = 5.2, 3.5 Hz, 2H), 7.15 (dd, *J* = 5.6, 3.1 Hz, 2H), 6.93 (br.s, 1H), 3.72 (dd, *J* = 8.2, 4.5 Hz, 1H), 3.67 – 3.60 (m, 1H), 3.31 (dd, *J* = 12.0, 8.3 Hz, 1H), 3.16 (ddd, *J* = 15.7, 6.9, 2.4 Hz, 2H), 3.02 (dd, *J* = 12.0, 4.6 Hz, 1H), 2.80 – 2.70 (m, 5H).

^{13}C NMR (151 MHz, CDCl$_3$) δ 172.1, 147.4, 146.0, 141.3, 141.3, 129.3 (2C), 126.8, 126.8, 124.9, 124.9, 124.1 (2C), 59.6, 52.7, 50.9, 39.9, 39.9, 26.4.

HRMS (ESI$^+$) m/z calculated for $[M+H]^+$ $[C_{19}H_{22}N_3O_3]^+$($[M+H]^+$) 340.1656, found 340.1648.

ATR-FTIR (cm^{-1}): 3297, 3070, 2937, 2843, 1647, 1516, 1343, 735, 698.

5o: *N*,2-dimethyl-3-((*N*-methyl-4-nitrophenyl)sulfonamido)-2-(4-nitrophenyl)propanamide

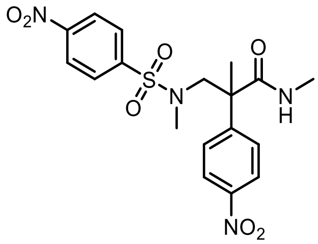

The product was synthesised according to **General Procedure C**, using *N*-methyl-*N*-(4-nitrophenyl)methacrylamide (28.4 mg, 0.100 mmol, 1.00 eq.) and *N*-methyl-4-nitrobenzenesulfonamide (32.4 mg, 0.150 mmol, 1.50 eq.), and obtained as a white solid (80%, 35.1 mg, 0.080 mmol). See NMR.

^{1}H NMR (400 MHz, CDCl$_3$) δ 8.36 (d, *J* = 8.7 Hz, 2H), 8.22 (d, *J* = 8.9 Hz, 2H), 7.92 (d, *J* = 8.7 Hz, 2H), 7.59 (d, *J* = 8.9 Hz, 2H), 5.52 (br s, 1H), 3.94 (d, *J* = 14.1 Hz, 1H), 3.44 (d, *J* = 14.1 Hz, 1H), 2.81 (d, *J* = 4.8 Hz, 3H), 2.45 (s, 3H), 1.84 (s, 3H).

^{13}C NMR (150 MHz, CDCl$_3$) δ 174.3, 150.4, 149.0, 147.5, 143.2, 128.8 (2C), 128.1 (2C), 124.6 (2C), 124.1 (2C), 58.7, 51.1, 37.4, 27.1, 21.2.

HRMS (ESI$^+$) *m/z* calculated for $[C_{18}H_{20}N_4NaO_7S]^+$ ($[M+Na]^+$) 459.0954, found 459.0948.

ATR-FTIR (cm^{-1}) 3340, 2925, 1633, 1606, 1519, 1341, 1154, 975, 849, 600.

5. One-pot amide coupling/Smiles rearrangement

Optimisation

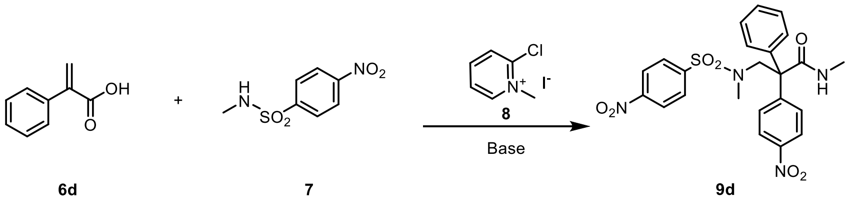

Entry	Base	Eq. Base	Eq. **8**	Solvent	c [M]	Eq. **7**	t [h]	T [°C]	Yield
1	Et_3N	3	1.3	DCM	0.2	1.1	6	20	37%
2	Et_3N	**2.5**	1.3	**DMA**	**0.05**	2	16	20	69%
3	Et_3N	2.5	1.3	DMA	0.05	2	**67**	20	25%
4	Et_3N	2.5	**1.3**	DMA	0.05	**2.5**	16	20	79%
5	**BTMG**	2.5	**1.3**	DMA	0.05	2.5	16	20	78%

Table 2 Optimisation for one-pot amide coupling/Smiles rearrangement.

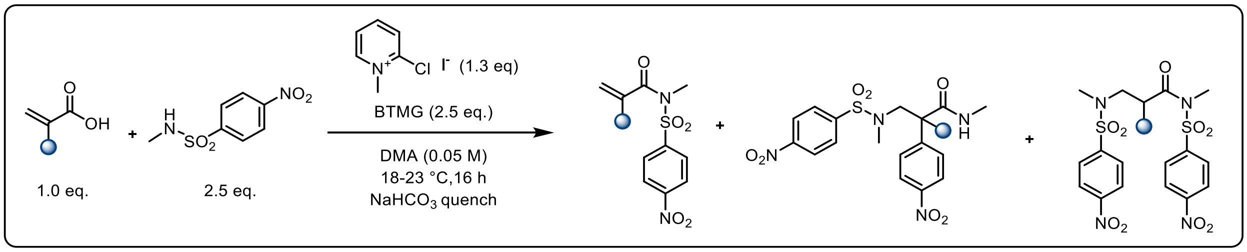

General Procedure C

To an oven-dried vial were added α,β-unsaturated carboxylic acid (1.0 eq.), *p*-nosyl methylamide (2.5 eq.) and 2-chloro-1-methylpyridinium iodide (1.3 eq.). The reactants were dissolved in DMA (0.05 M) and BTMG (2.5 eq.) was added. The solution was stirred at 20 °C for 16 h. The solution was then diluted with DCM (20 mL), washed sequentially with aqueous saturated $NaHCO_3$ solution (20 mL) and brine (2 x 20 mL). The organic phase was dried over $MgSO_4$, filtered, concentrated *in vacuo* and the product was purified by flash column chromatography on silica gel (EtOAc/heptane) to afford the products.

9b: *N*-methyl-3-((N-methyl-4-nitrophenyl)sulfonamido)-2-(4-nitrophenyl)-2-phenylpropanamide

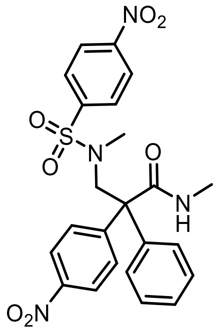

The product was synthesised according to **General Procedure C** using atropic acid (14.8 mg, 0.100 mmol, 1.00 eq.), and obtained as a white solid (78%, 39.0 mg, 0.078 mmol). See NMR.

^{1}H NMR (600 MHz, $CDCl_3$) δ 8.39 – 8.32 (m, 2H), 8.27 – 8.21 (m, 2H), 7.91 – 7.83 (m, 2H), 7.61 – 7.54 (m, 2H), 7.46 – 7.36 (m, 3H), 7.31 – 7.26 (m, 2H), 5.62 (d, J = 4.8 Hz, 1H), 4.31 (d, J = 13.9 Hz, 1H), 4.16 (d, J = 13.9 Hz, 1H), 2.81 (d, J = 4.8 Hz, 3H), 2.58 (s, 3H).

^{13}C NMR (151 MHz, $CDCl_3$) δ 172.6, 150.3, 147.39, 147.36, 143.5, 139.9, 130.9 (2C), 129.3 (2C), 128.9 (2C), 128.7 (2C), 128.6, 124.6 (2C), 123.5 (2C), 61.7, 57.0, 37.0, 27.4.

HRMS (ESI$^+$) *m/z* calculated for $[C_{23}H_{22}N_4NaO_7S]^+$ ($[M+Na]^+$) 521.1101, found 521.1092.

ATR-FTIR (cm^{-1}) 3413, 2926, 1663, 1519, 1348, 1165, 741, 600.

9b: *N*-methyl-N-((4-nitrophenyl)sulfonyl)-2-phenylacrylamide

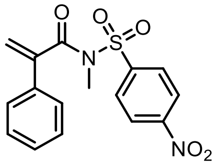

The product was synthesized according to **General Procedure C** using atropic acid (14.8 mg, 0.100 mmol, 1.00 eq.), and obtained as a white solid (14%, 4.8 mg, 0.014 mmol). See NMR.

^{1}H NMR (600 MHz, $CDCl_3$) δ 8.37 (d, J = 8.8 Hz, 2H), 8.16 (d, J = 8.8 Hz, 2H), 7.39 – 7.32 (m, 3H), 7.30 – 7.22 (m, 2H), 5.81 (s, 1H), 5.51 (s, 1H), 3.22 (s, 3H).

^{13}C NMR (151 MHz, $CDCl_3$) δ 170.4, 150.8, 144.33, 144.28, 134.3, 130.1 (2C), 129.5, 129.3 (2C), 126.0 (2C), 124.2 (2C), 119.0, 34.8.

HRMS (ESI$^+$) *m/z* calculated for $[C_{16}H_{14}N_2NaO_5S]^+$) ($[M+Na]^+$) 369.0516, found 369.0512.

ATR-FTIR (cm^{-1}) 3106, 1690, 1529, 1348, 1173, 854, 738, 682, 637, 578.

9a: 2-benzyl-*N*-methyl-3-((N-methyl-4-nitrophenyl)sulfonamido)-2-(4-nitrophenyl)propanamide

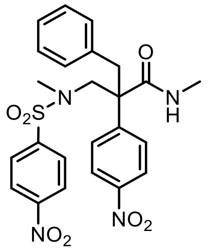

The product was synthesised according to **General Procedure C** using 2-benzylacrylic acid (17.9 mg, 0.100 mmol, 1.00 eq.), and obtained as a pale yellow viscous oil (35%, 17.9 mg, 0.035 mmol). See NMR.

^{1}H NMR (700 MHz, $CDCl_3$) δ 8.40 – 8.35 (m, 2H), 8.27 – 8.21 (m, 2H), 7.91 – 7.86 (m, 2H), 7.65 – 7.59 (m, 2H), 7.25 (dd, *J* = 5.8, 3.5 Hz, 3H), 7.06 (dd, *J* = 6.6, 2.8 Hz, 2H), 5.64 (d, *J* = 3.8 Hz, 1H), 3.70 – 3.63 (m, 2H), 3.62 (d, *J* = 14.2 Hz, 1H), 3.39 (d, *J* = 14.2 Hz, 1H), 2.72 (d, *J* = 4.7 Hz, 3H), 2.43 (s, 3H).

^{13}C NMR (176 MHz, $CDCl_3$) δ 172.6, 150.5, 147.5, 147.4, 142.5, 135.7, 130.3 (2C), 130.1, 128.9, 128.6 (2C), 127.7, 124.6 (2C), 123.7 (2C), 58.3, 57.6, 42.4, 37.8, 26.8.

HRMS (ESI$^+$) m/z calculated for $[C_{24}H_{24}N_4NaO_7S]^+$ ($[M+Na]^+$) 535.1258, found 535.1249.

ATR-FTIR (cm^{-1}) 3411,2930, 1658, 1604, 1527, 1348, 1166, 855, 742.

10a: 2-benzyl-*N*-methyl-*N*-((4-nitrophenyl)sulfonyl)acrylamide

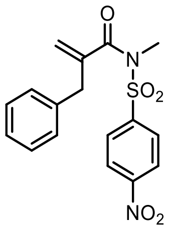

The product was synthesized according to **General Procedure C** using 2-benzylacrylic acid (16.2 mg, 0.100 mmol, 1.00 eq), and obtained as a white solid (49%, 17.5 mg, 0.049 mmol). See NMR.

^{1}H NMR (700 MHz, $CDCl_3$) δ 8.33 – 8.28 (m, 2H), 7.97 – 7.91 (m, 2H), 7.25 – 7.21 (m, 3H), 7.11 – 7.05 (m, 2H), 5.46 (app.t, *J* = 1.4 Hz, 1H), 5.41 (s, 1H), 3.61 (s, 2H), 3.13 (s, 3H).

^{13}C NMR (176 MHz, $CDCl_3$) δ 171.4, 150.7, 143.9, 143.5, 136.9, 129.8 (2C), 129.3 (2C), 128.9 (2C), 127.1, 124.2 (2C), 120.9, 39.9, 35.1.

HRMS (ESI$^+$) *m/z* calculated for $[C_{17}H_{16}N_2NaO_5S]^+$ ($[M+Na]^+$) requires 383.0672, found 383.0669.

ATR-FTIR (cm^{-1}) 2922, 2852, 1690, 1531, 1351, 1176, 1012, 742.

6. Mechanistical Proposal

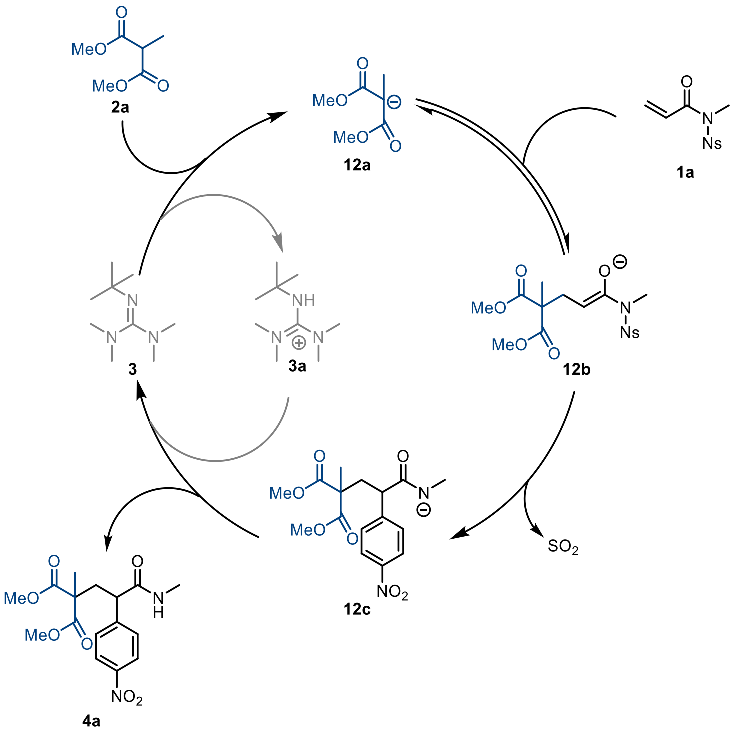

We surmised that the reaction is initiated by the deprotonation of **2a**, forming anion **12b**. This intermediate is captured by sulfonyl imide **1a**, leading to formation of **12b,** which undergoes subsequent rearrangement and extrusion of SO_2. By reprotonation with **3a**, the product **4a** is released with regeneration of the base **3**.

7. X-ray Analysis

5e: *N*,2-dimethyl-2-(4-nitrophenyl)-3-(phenylsulfonyl)propanamide

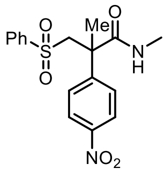

5e

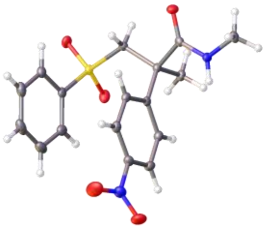

CSD-ID: 2313883

Table 4. Crystal data and structure refinement for **5e**.

Identification code	**5e**	
Empirical formula	$C_{17}H_{18}N_2O_5S$	
Formula weight	362.40	
Temperature	100 K	
Wavelength	1.54186 Å	
Crystal system	Monoclinic	
Space group	$P2_1/c$	
Unit cell dimensions	a = 17.9792(4) Å	α= 90°.
	b = 6.0373(2) Å	β= 98.835(2)°.
	c = 15.3625(3) Å	γ = 90°.
Volume	1647.74(7) $Å^3$	
Z	4	
Density (calculated)	1.461 mg/m^3	
Absorption coefficient	2.033 mm^{-1}	
F(000)	760	
Crystal size	0.123 x 0.068 x 0.040 mm^3	
Theta range for data collection	4.979 to 71.167°.	
Index ranges	-21<=h<=5, -7<=k<=6, -17<=l<=18	
Reflections collected	18699	
Independent reflections	3158 [R(int) = 0.0295]	
Completeness to theta = 67.686°	99.1 %	
Absorption correction	Semi-empirical from equivalents	
Max. and min. transmission	0.9670 and 0.5844	
Refinement method	Full-matrix least-squares on F^2	

Data / restraints / parameters	3158 / 0 / 228
Goodness-of-fit on F^2	1.008
Final R indices [I>2sigma(I)]	R1 = 0.0324, wR2 = 0.0834
R indices (all data)	R1 = 0.0402, wR2 = 0.0877
Extinction coefficient	n/a
Largest diff. peak and hole	0.240 and -0.394 e.$Å^{-3}$

Table 4. Atomic coordinates (x 10^4) and equivalent isotropic displacement parameters ($Å^2$x 10^3) for **27**. U(eq) is defined as one third of the trace of the orthogonalized U^{ij} tensor.

	x	y	z	U(eq)
S(1)	1810(1)	2532(1)	8925(1)	13(1)
O(1)	1948(1)	362(2)	8596(1)	21(1)
O(2)	1438(1)	2668(2)	9694(1)	19(1)
O(3)	4716(1)	4472(2)	6152(1)	31(1)
O(4)	406(1)	6758(2)	6929(1)	19(1)
O(5)	4792(1)	7013(3)	7148(1)	40(1)
N(1)	1004(1)	5955(2)	5775(1)	16(1)
N(2)	4449(1)	5542(2)	6706(1)	23(1)
C(1)	2680(1)	3912(3)	9191(1)	15(1)
C(2)	2692(1)	5970(3)	9601(1)	18(1)
C(3)	3367(1)	7100(3)	9786(1)	24(1)
C(4)	4020(1)	6164(3)	9573(1)	28(1)
C(5)	4008(1)	4098(3)	9182(1)	27(1)
C(6)	3332(1)	2949(3)	8984(1)	21(1)
C(7)	1244(1)	4174(2)	8108(1)	13(1)
C(8)	1373(1)	3900(2)	7141(1)	12(1)
C(9)	2195(1)	4265(2)	7036(1)	13(1)
C(10)	2609(1)	2674(2)	6654(1)	15(1)
C(11)	3351(1)	3077(3)	6545(1)	18(1)
C(12)	3674(1)	5071(3)	6837(1)	17(1)
C(13)	3286(1)	6688(3)	7228(1)	17(1)
C(14)	2544(1)	6271(2)	7314(1)	14(1)
C(15)	1044(1)	1665(2)	6787(1)	16(1)
C(16)	879(1)	5715(2)	6609(1)	13(1)
C(17)	531(1)	7328(3)	5132(1)	17(1)

Table 5. Bond lengths [Å] and angles [°] for **27**.

S(1)-O(1)	1.4392(11)
S(1)-O(2)	1.4476(11)
S(1)-C(1)	1.7647(16)
S(1)-C(7)	1.7891(14)
O(3)-N(2)	1.2238(19)
O(4)-C(16)	1.2215(19)
O(5)-N(2)	1.226(2)
N(1)-C(16)	1.3417(19)
N(1)-C(17)	1.4580(19)
N(2)-C(12)	1.467(2)
C(1)-C(6)	1.389(2)
C(1)-C(2)	1.391(2)
C(2)-C(3)	1.384(2)
C(3)-C(4)	1.387(3)
C(4)-C(5)	1.383(3)
C(5)-C(6)	1.391(3)
C(7)-C(8)	1.5456(19)
C(8)-C(9)	1.528(2)
C(8)-C(15)	1.538(2)
C(8)-C(16)	1.561(2)
C(9)-C(10)	1.399(2)
C(9)-C(14)	1.400(2)
C(10)-C(11)	1.391(2)
C(11)-C(12)	1.381(2)
C(12)-C(13)	1.388(2)
C(13)-C(14)	1.384(2)
O(1)-S(1)-O(2)	117.72(7)
O(1)-S(1)-C(1)	108.43(7)
O(2)-S(1)-C(1)	106.73(7)
O(1)-S(1)-C(7)	111.62(7)
O(2)-S(1)-C(7)	104.94(7)
C(1)-S(1)-C(7)	106.80(7)
C(16)-N(1)-C(17)	122.87(13)

O(3)-N(2)-O(5)	123.40(15)
O(3)-N(2)-C(12)	118.44(14)
O(5)-N(2)-C(12)	118.14(14)
C(6)-C(1)-C(2)	121.55(15)
C(6)-C(1)-S(1)	119.93(13)
C(2)-C(1)-S(1)	118.52(12)
C(3)-C(2)-C(1)	118.99(15)
C(2)-C(3)-C(4)	119.90(17)
C(5)-C(4)-C(3)	120.83(16)
C(4)-C(5)-C(6)	119.99(16)
C(1)-C(6)-C(5)	118.72(16)
C(8)-C(7)-S(1)	117.50(10)
C(9)-C(8)-C(15)	114.49(12)
C(9)-C(8)-C(7)	112.06(11)
C(15)-C(8)-C(7)	109.14(12)
C(9)-C(8)-C(16)	108.97(11)
C(15)-C(8)-C(16)	105.89(11)
C(7)-C(8)-C(16)	105.75(11)
C(10)-C(9)-C(14)	118.64(14)
C(10)-C(9)-C(8)	122.13(13)
C(14)-C(9)-C(8)	119.21(13)
C(11)-C(10)-C(9)	120.86(14)
C(12)-C(11)-C(10)	118.40(14)
C(11)-C(12)-C(13)	122.69(14)
C(11)-C(12)-N(2)	118.97(14)
C(13)-C(12)-N(2)	118.31(14)
C(14)-C(13)-C(12)	117.97(14)
C(13)-C(14)-C(9)	121.42(14)
O(4)-C(16)-N(1)	123.92(14)
O(4)-C(16)-C(8)	121.74(13)
N(1)-C(16)-C(8)	114.30(12)

Symmetry transformations used to generate equivalent atoms:

Table 6. Anisotropic displacement parameters ($Å^2$x 10^3) for **5e**. The anisotropic displacement factor exponent takes the form: $-2\pi^2[h^2 a^{*2}U^{11} + ... + 2 h k a^* b^* U^{12}]$

	U^{11}	U^{22}	U^{33}	U^{23}	U^{13}	U^{12}
S(1)	17(1)	14(1)	9(1)	1(1)	2(1)	0(1)
O(1)	30(1)	14(1)	18(1)	0(1)	-1(1)	2(1)
O(2)	21(1)	25(1)	12(1)	2(1)	5(1)	-3(1)
O(3)	22(1)	41(1)	33(1)	-2(1)	14(1)	3(1)
O(4)	19(1)	20(1)	19(1)	2(1)	5(1)	7(1)
O(5)	24(1)	50(1)	49(1)	-17(1)	13(1)	-16(1)
N(1)	17(1)	19(1)	12(1)	2(1)	2(1)	5(1)
N(2)	17(1)	30(1)	22(1)	2(1)	6(1)	-1(1)
C(1)	17(1)	18(1)	10(1)	3(1)	0(1)	2(1)
C(2)	20(1)	20(1)	13(1)	2(1)	-1(1)	2(1)
C(3)	27(1)	25(1)	16(1)	3(1)	-4(1)	-5(1)
C(4)	19(1)	43(1)	20(1)	9(1)	-4(1)	-8(1)
C(5)	17(1)	45(1)	20(1)	6(1)	2(1)	4(1)
C(6)	21(1)	27(1)	13(1)	4(1)	2(1)	7(1)
C(7)	13(1)	15(1)	12(1)	1(1)	2(1)	1(1)
C(8)	14(1)	12(1)	11(1)	1(1)	2(1)	1(1)
C(9)	14(1)	15(1)	8(1)	2(1)	1(1)	1(1)
C(10)	18(1)	16(1)	13(1)	-1(1)	2(1)	1(1)
C(11)	19(1)	20(1)	15(1)	0(1)	5(1)	4(1)
C(12)	14(1)	23(1)	15(1)	2(1)	3(1)	-1(1)
C(13)	19(1)	17(1)	13(1)	2(1)	1(1)	-2(1)
C(14)	18(1)	14(1)	11(1)	2(1)	2(1)	2(1)
C(15)	18(1)	14(1)	13(1)	-1(1)	1(1)	-2(1)
C(16)	13(1)	12(1)	14(1)	0(1)	0(1)	-2(1)
C(17)	19(1)	18(1)	14(1)	4(1)	-2(1)	1(1)

Table 7. Hydrogen coordinates (x 10^4) and isotropic displacement parameters ($Å^2$x 10^{3}) for **5e**.

	x	y	z	U(eq)
H(1)	1387	5253	5609	19
H(2)	2242	6589	9752	21
H(3)	3383	8516	10058	28
H(4)	4481	6952	9697	34
H(5)	4461	3463	9049	33
H(6)	3317	1533	8712	25
H(7A)	710	3838	8135	16
H(7B)	1327	5751	8271	16
H(10)	2381	1301	6467	18
H(11)	3629	2006	6276	21
H(13)	3523	8039	7430	20
H(14)	2265	7370	7567	17
H(15A)	1091	1530	6162	23
H(15B)	512	1586	6855	23
H(15C)	1320	456	7118	23
H(17A)	467	6601	4555	26
H(17B)	771	8774	5092	26
H(17C)	38	7531	5317	26

8. NMR Spectra

1oa: *N*-methyl-5-(trifluoromethyl)pyridine-2-sulfonamide

^{1}H NMR (400 MHz, $CDCl_3$):

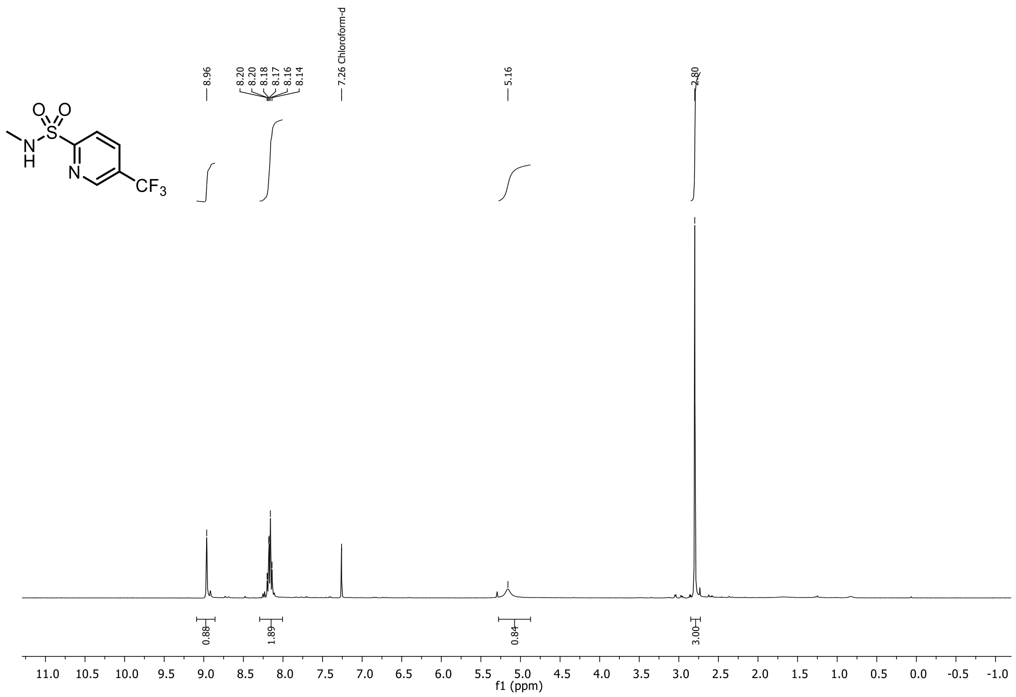

^{13}C NMR (101 MHz, $CDCl_3$):

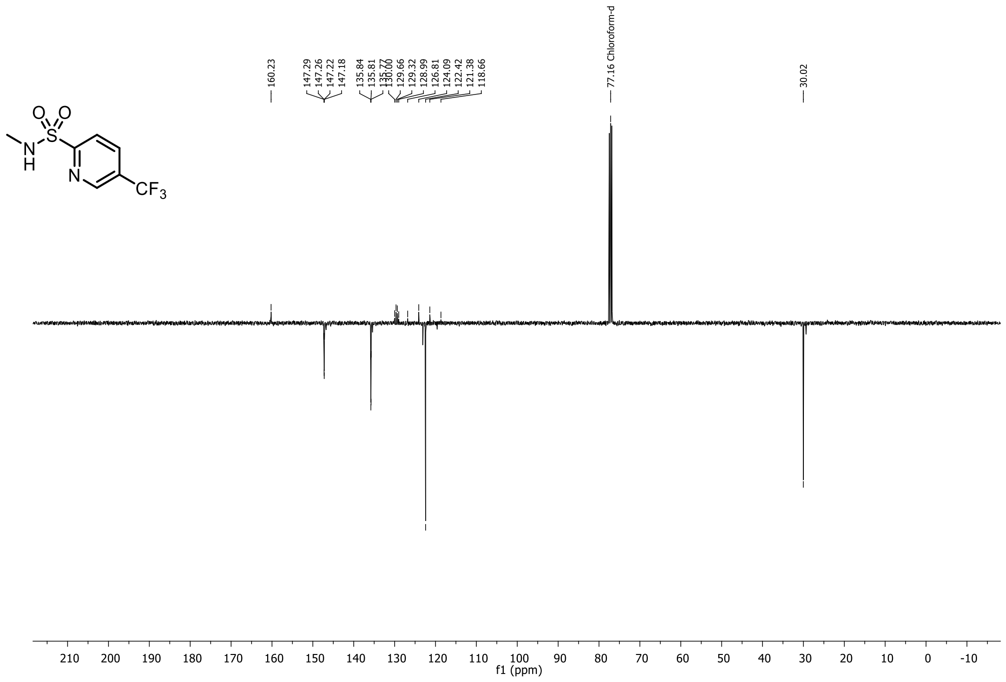

^{19}F NMR (376 MHz, $CDCl_3$):

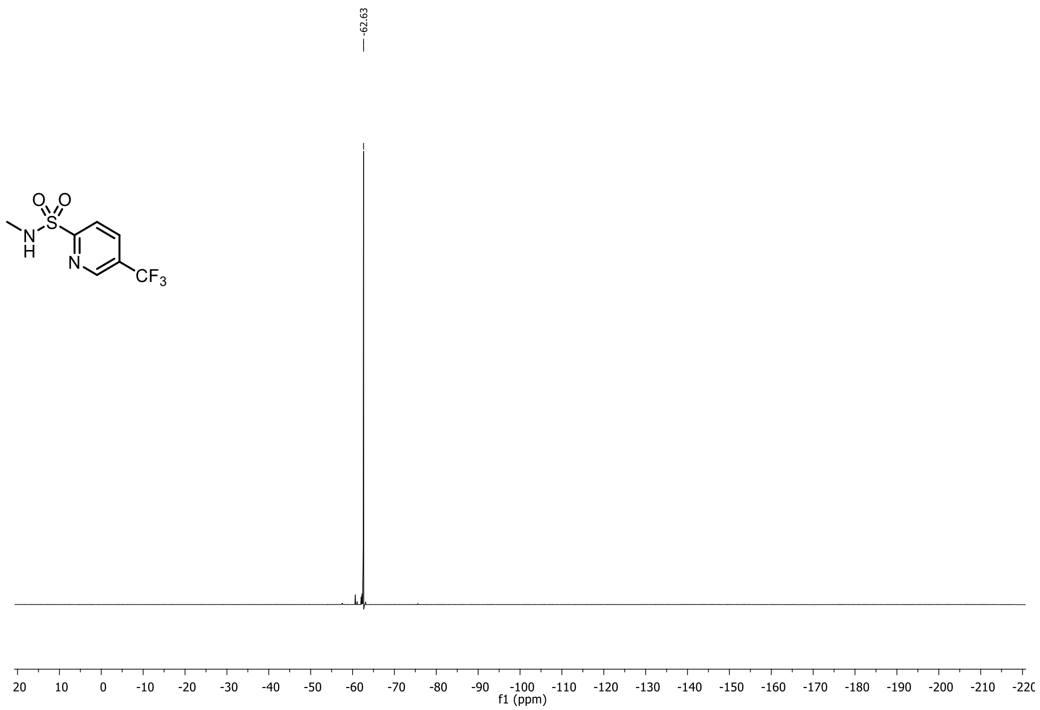

1pa: (*E*)-*N*-methyl-2-(4-nitrophenyl)ethene-1-sulfonamide

¹H NMR (600 MHz, $CDCl_3$):

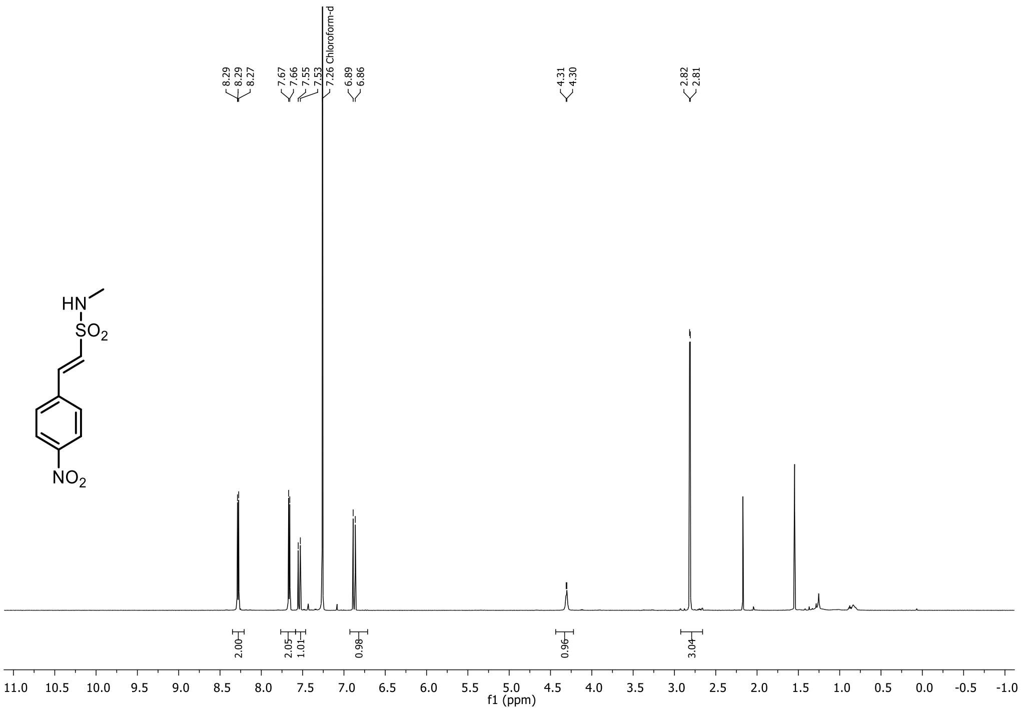

¹³C NMR (151MHz, $CDCl_3$):

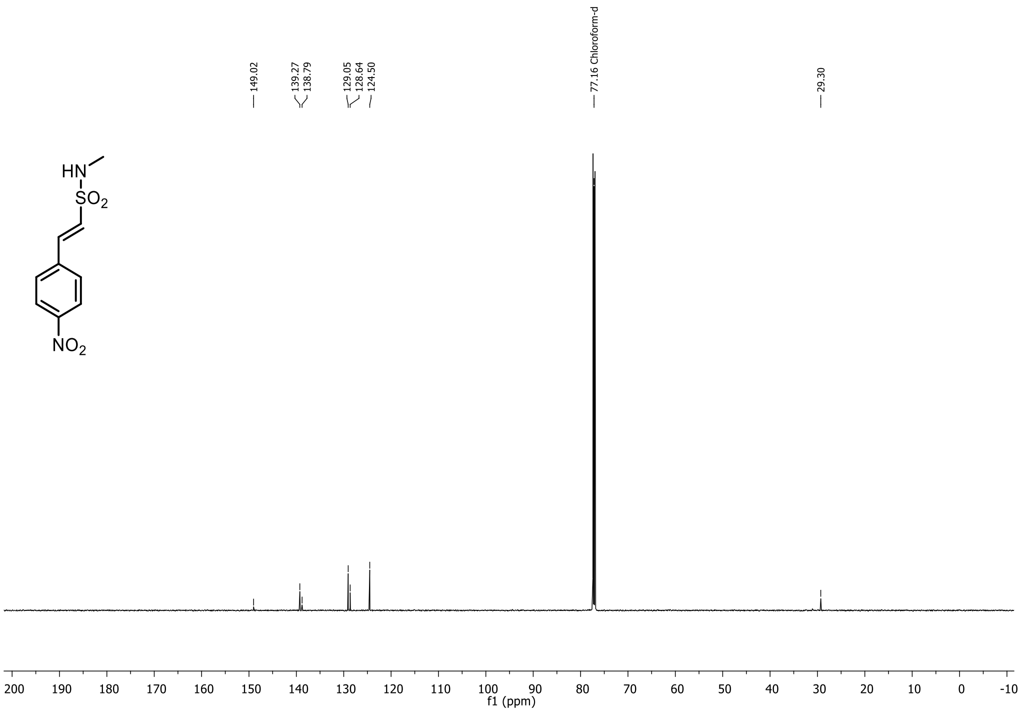

1qa: (*E*)-*N*-methyl-2-phenylethene-1-sulfonamide

^{1}H NMR (600 MHz, $CDCl_3$):

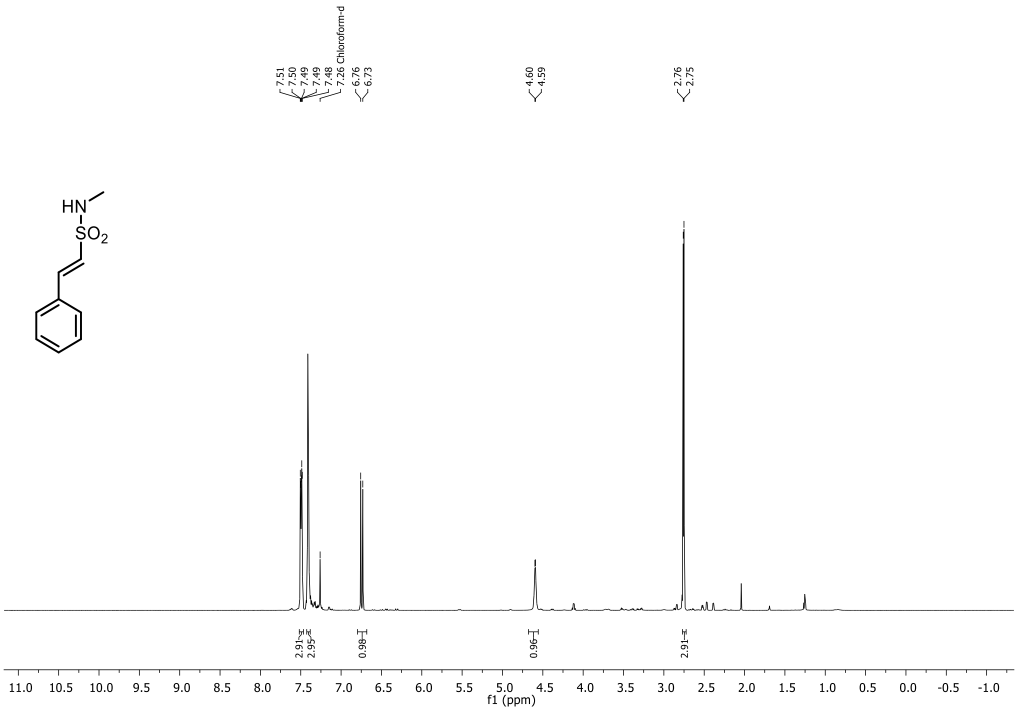

^{13}C NMR (151MHz, $CDCl_3$):

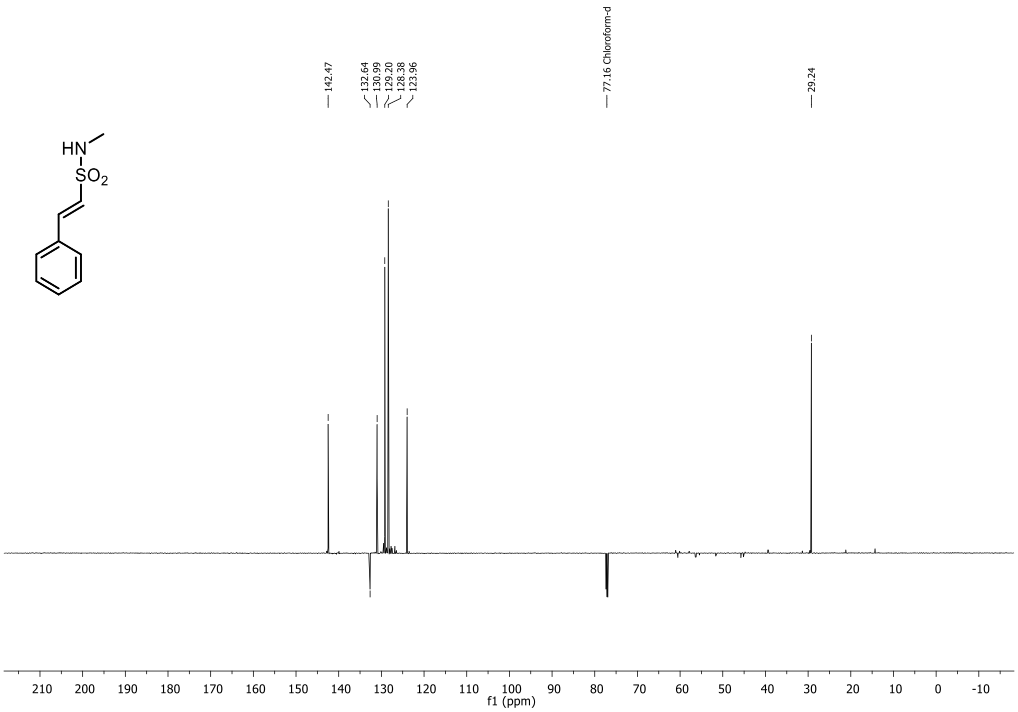

1ra: (*E*)-*N*-methyl-2-(naphthalen-2-yl)ethene-1-sulfonamide

^{1}H NMR (700 MHz, $CDCl_3$):

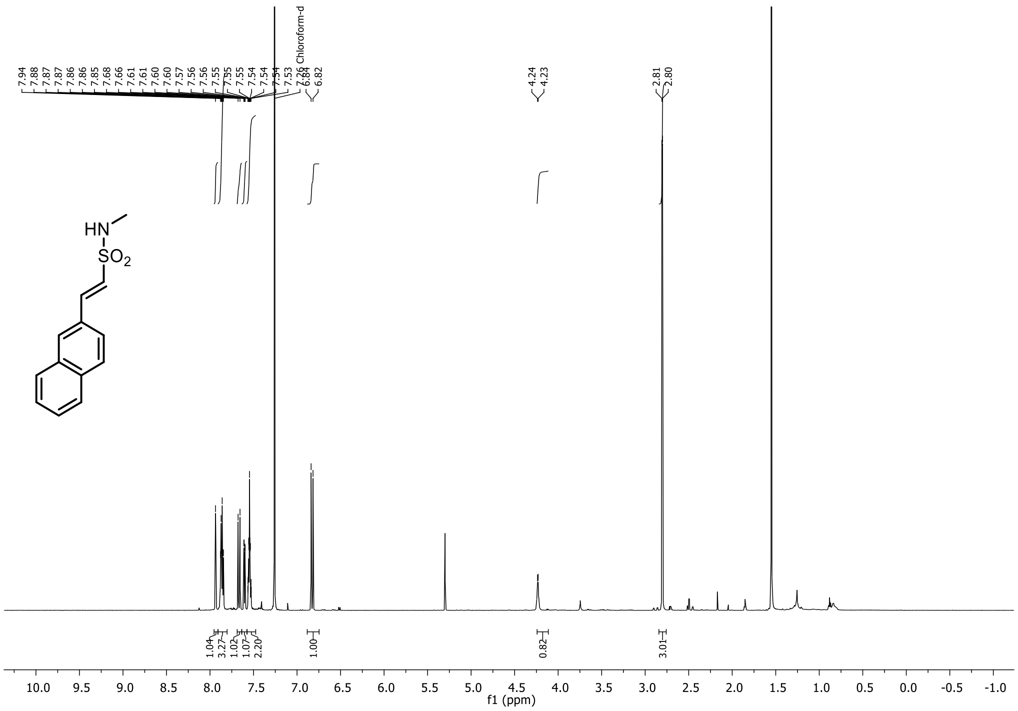

^{13}C NMR (176 MHz, $CDCl_3$):

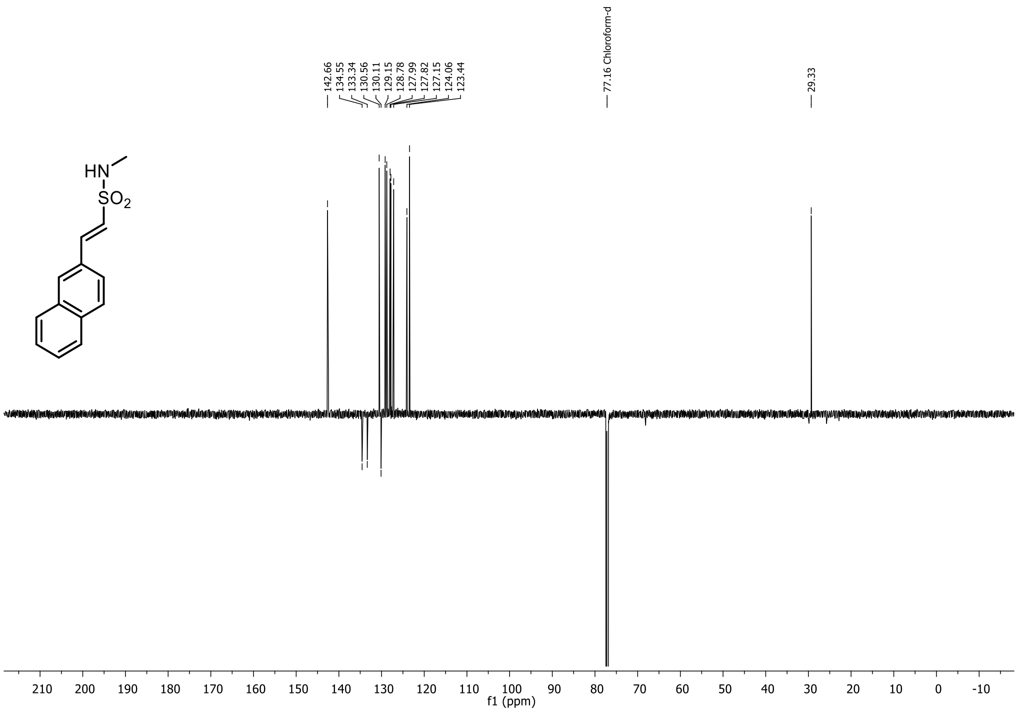

1l: *N*-methyl-*N*-((4-nitrophenyl)sulfonyl)methacrylamide

^{1}H NMR (400 MHz, $CDCl_3$):

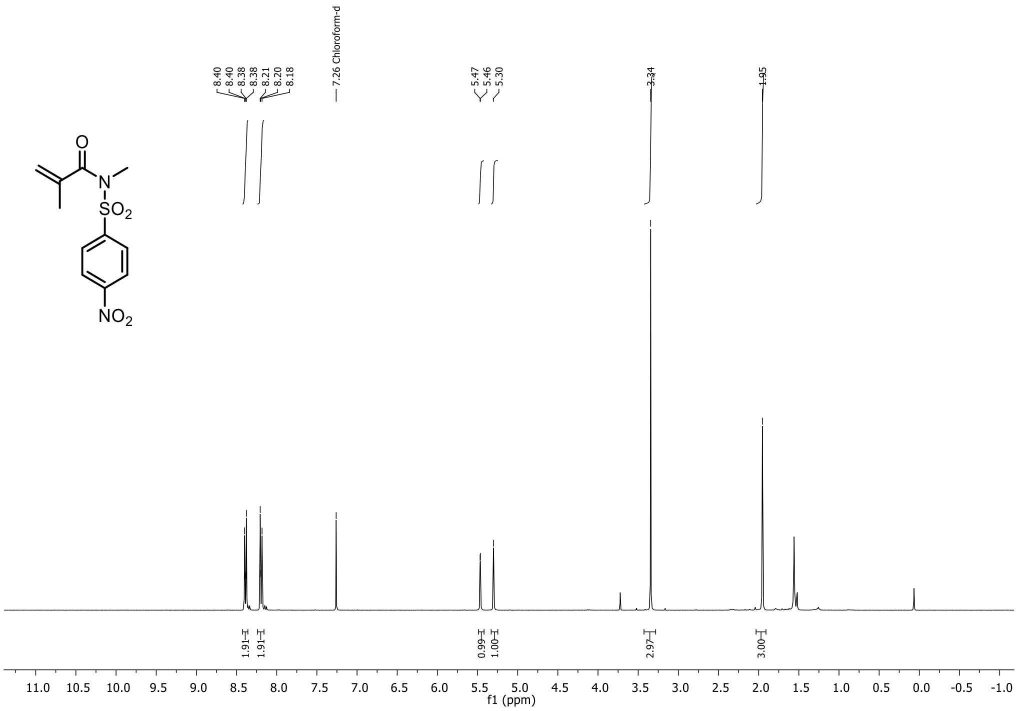

^{13}C NMR (151 MHz, $CDCl_3$):

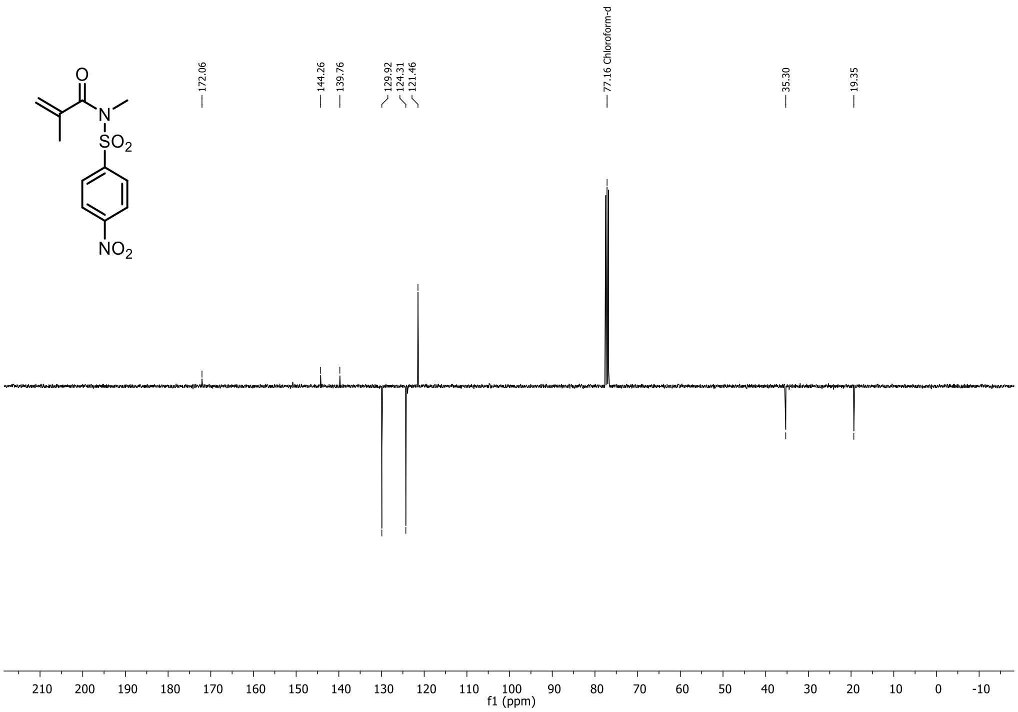

1o: *N*-methyl-*N*-((5-(trifluoromethyl)pyridin-2-yl)sulfonyl)acrylamide

^{1}H NMR (600 MHz, $CDCl_3$):

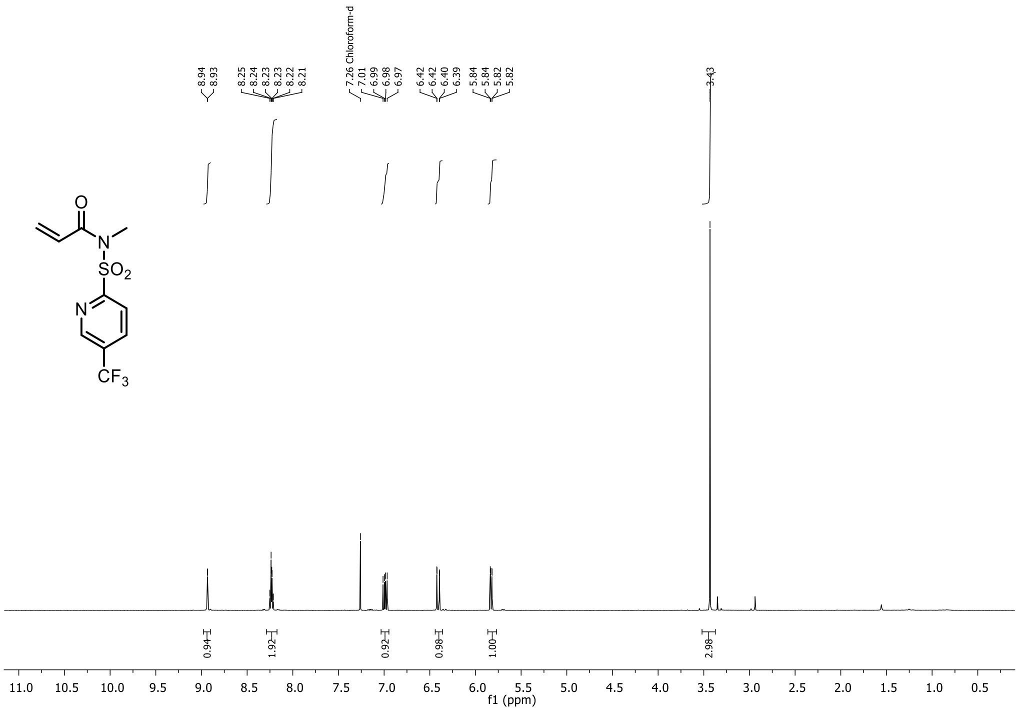

^{13}C NMR (151 MHz, $CDCl_3$):

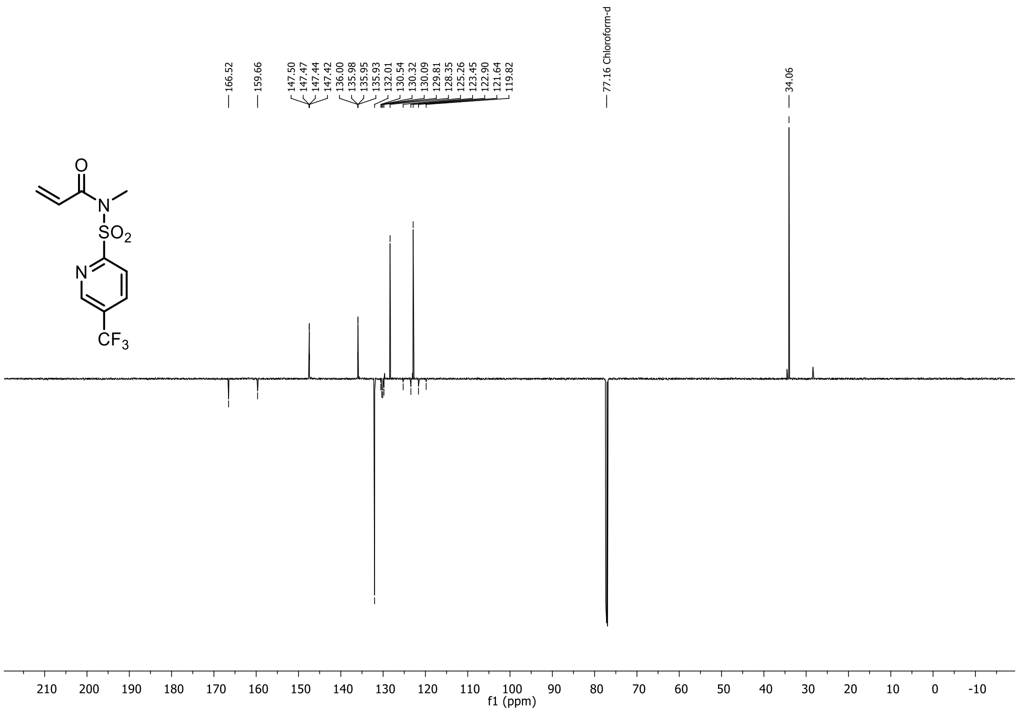

^{19}F NMR (376 MHz, $CDCl_3$):

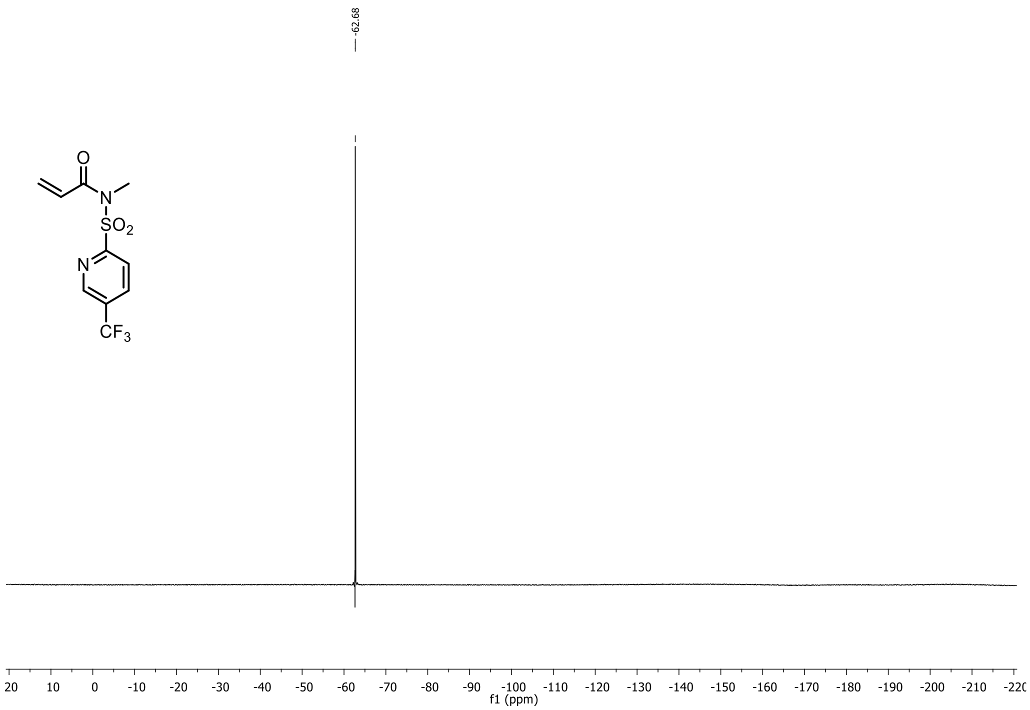

1p: (*E*)-*N*-methyl-*N*-((4-nitrostyryl)sulfonyl)acrylamide

^{1}H NMR (700 MHz, $CDCl_3$):

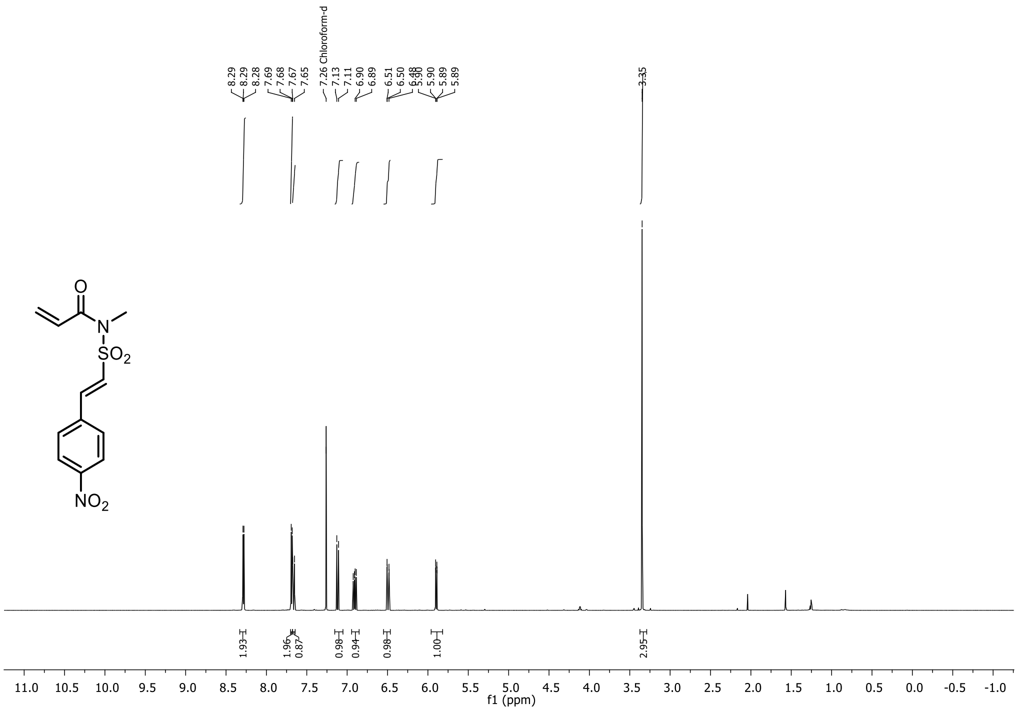

^{13}C NMR (176 MHz, $CDCl_3$):

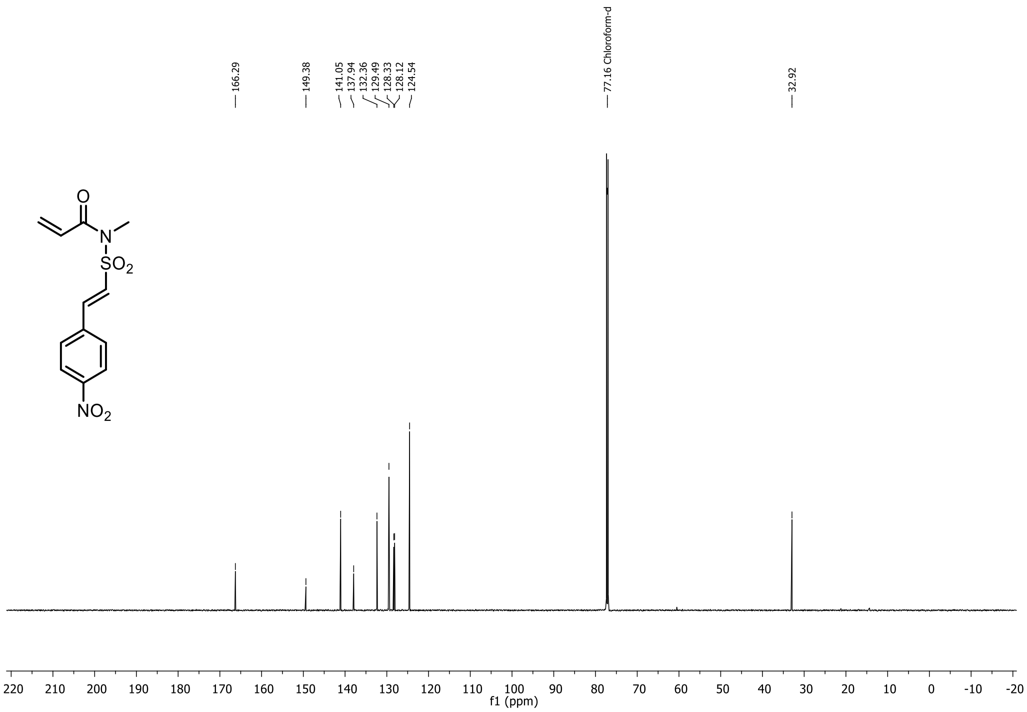

1q: (*E*)-*N*-methyl-*N*-(styrylsulfonyl)acrylamide

^{1}H NMR (600 MHz, $CDCl_3$):

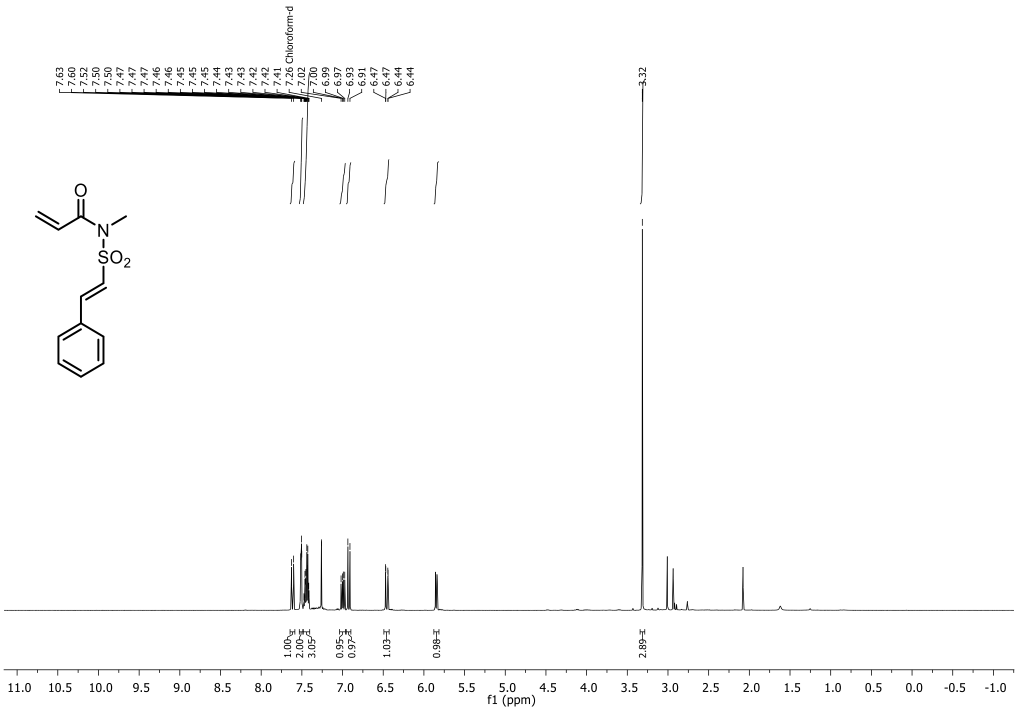

^{13}C NMR (151 MHz, $CDCl_3$):

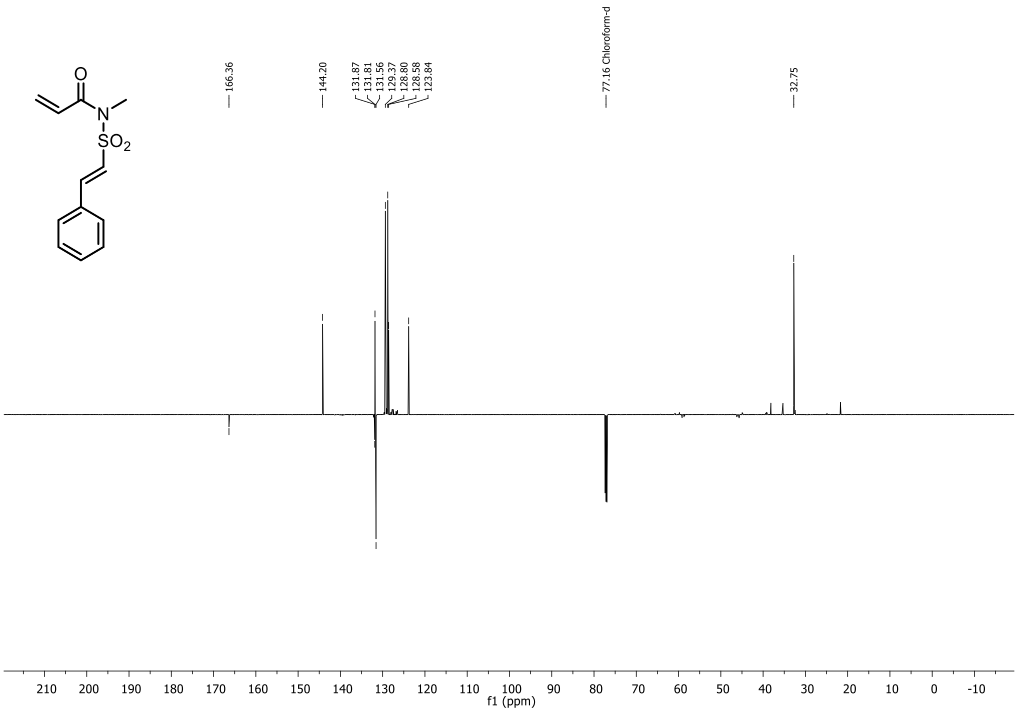

1r: (*E*)-*N*-methyl-*N*-((2-(naphthalen-2-yl)vinyl)sulfonyl)acrylamide

^{1}H NMR (700 MHz, $CDCl_3$):

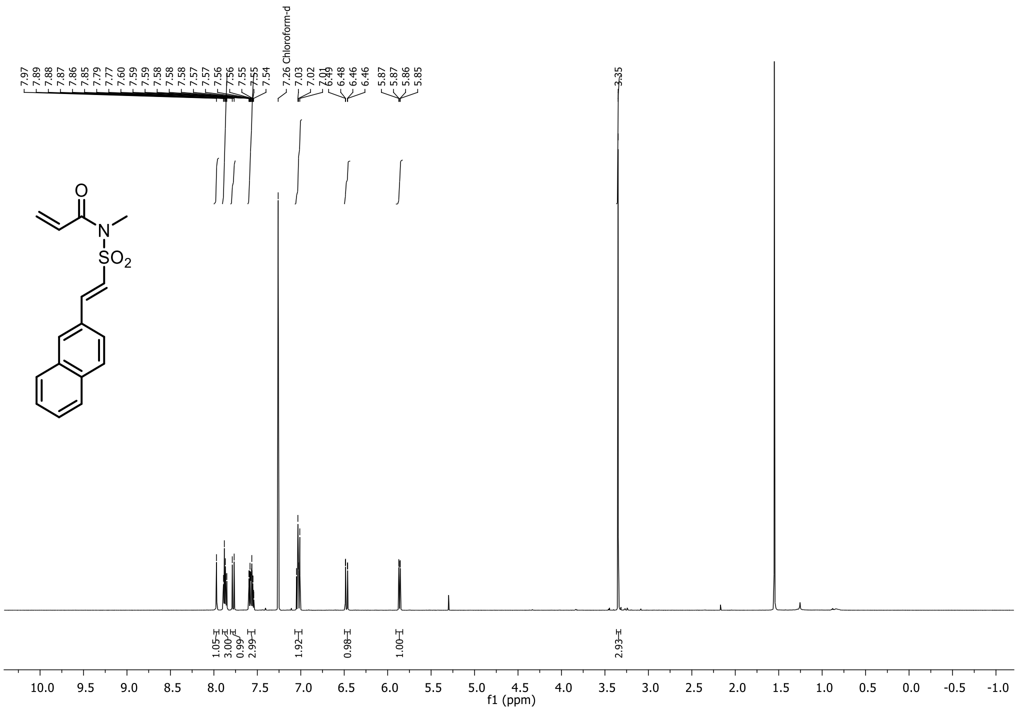

^{13}C NMR (176 MHz, $CDCl_3$):

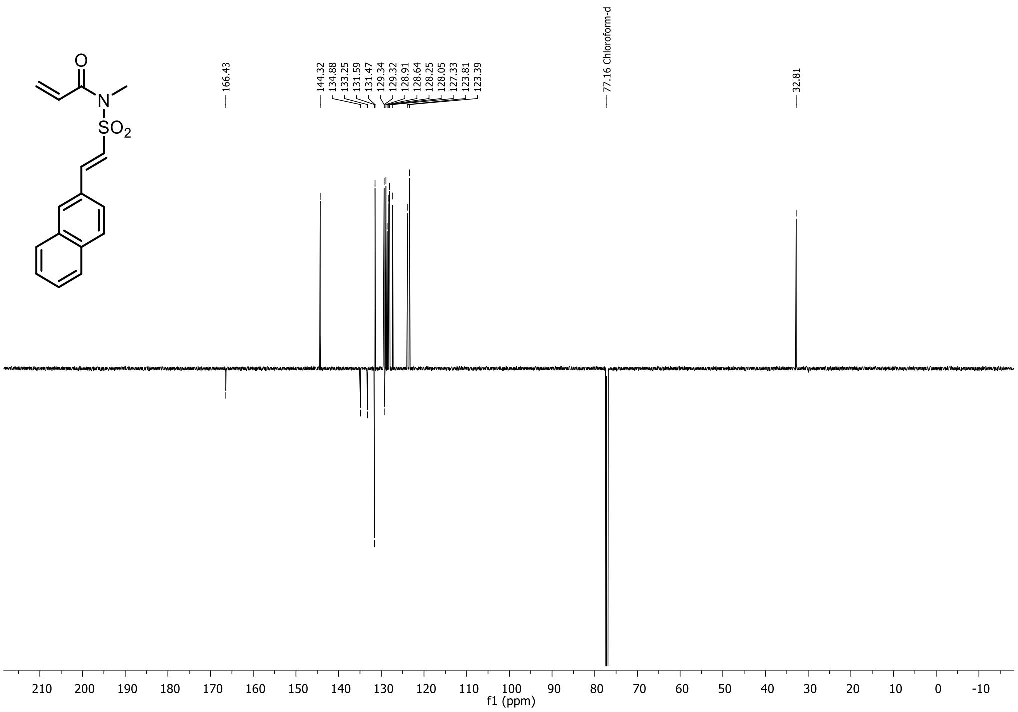

S1: N-methyl-N-phenylacrylamide

^{1}H NMR (600 MHz, $CDCl_3$)

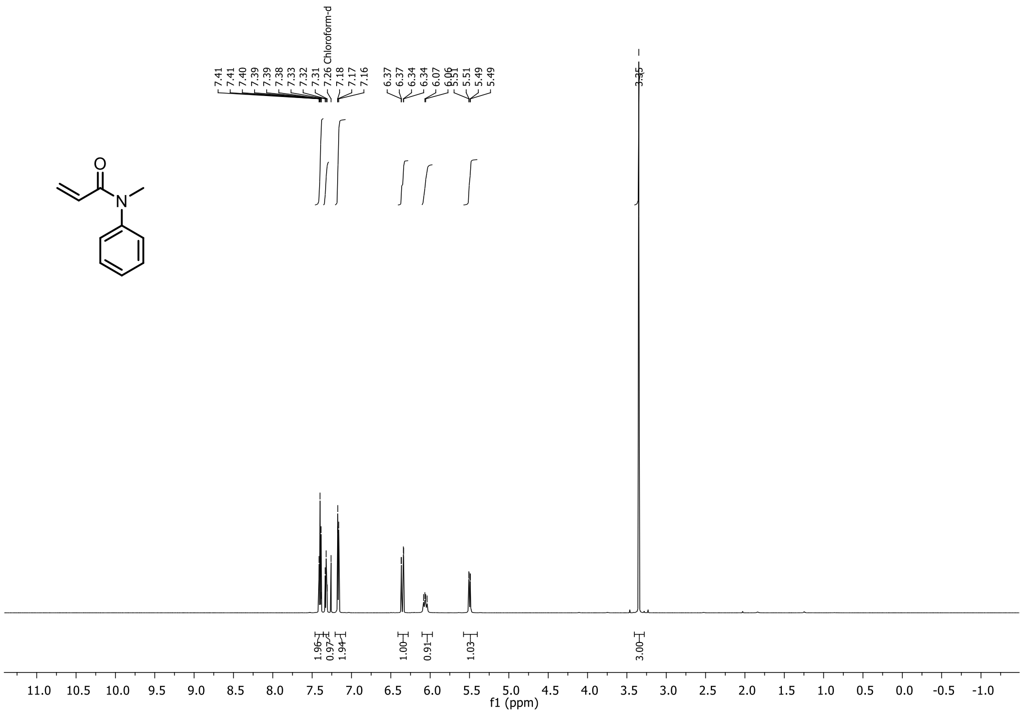

^{13}C NMR (151 MHz, $CDCl_3$)

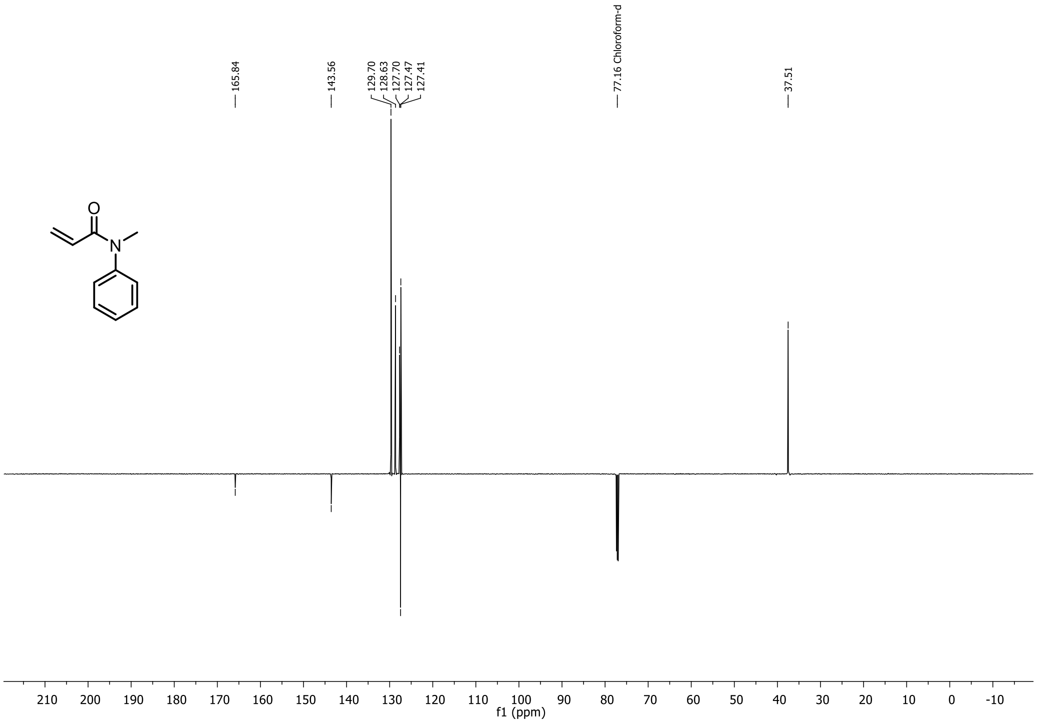

S2: *N*-methyl-3-((*N*-methyl-4-nitrophenyl)sulfonamido)-*N*-phenylpropanamide

^{1}H NMR (600 MHz, $CDCl_3$)

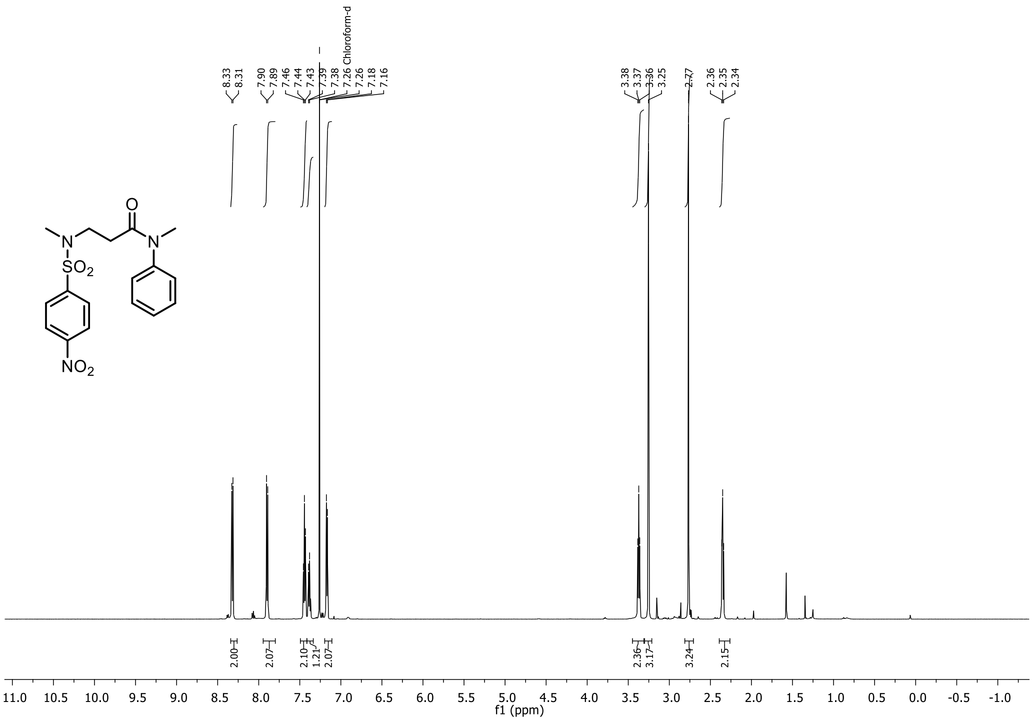

^{13}C NMR (151 MHz, $CDCl_3$)

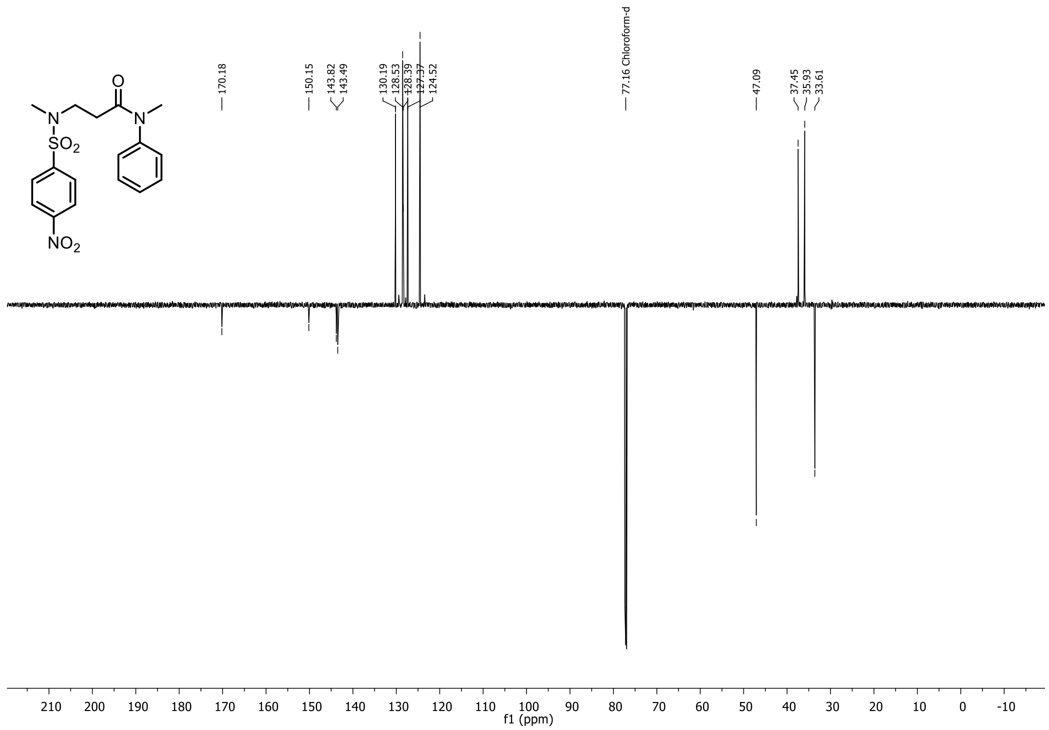

4a: Dimethyl 2-methyl-2-(3-(methylamino)-2-(4-nitrophenyl)-3-oxopropyl)malonate

^{1}H NMR (700 MHz, $CDCl_3$):

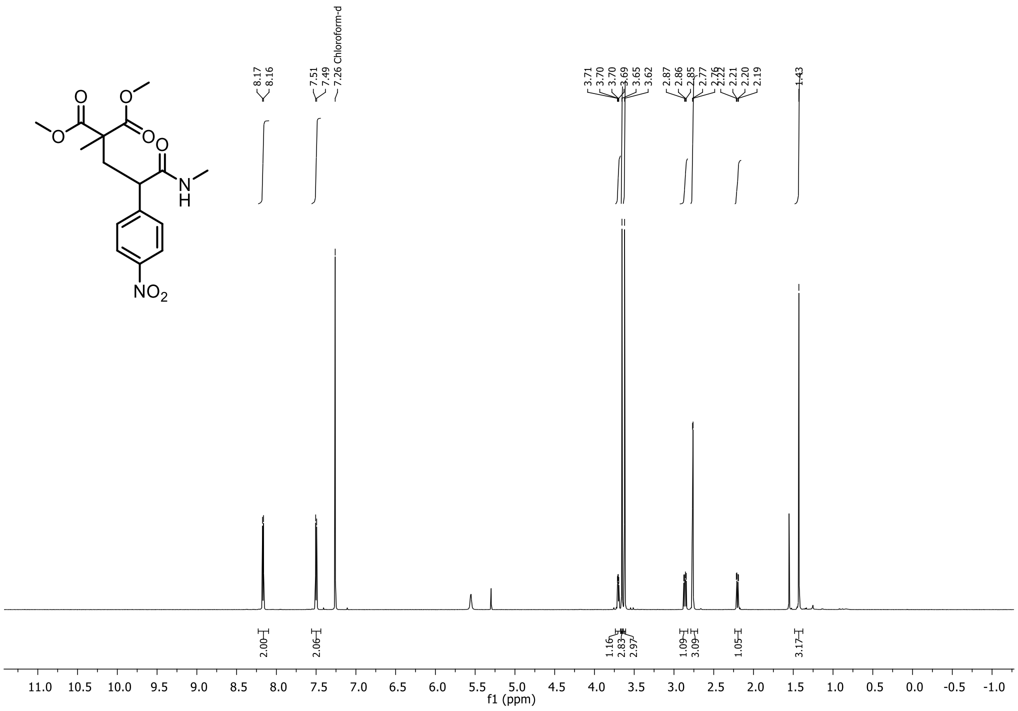

^{13}C NMR (176 MHz, $CDCl_3$):

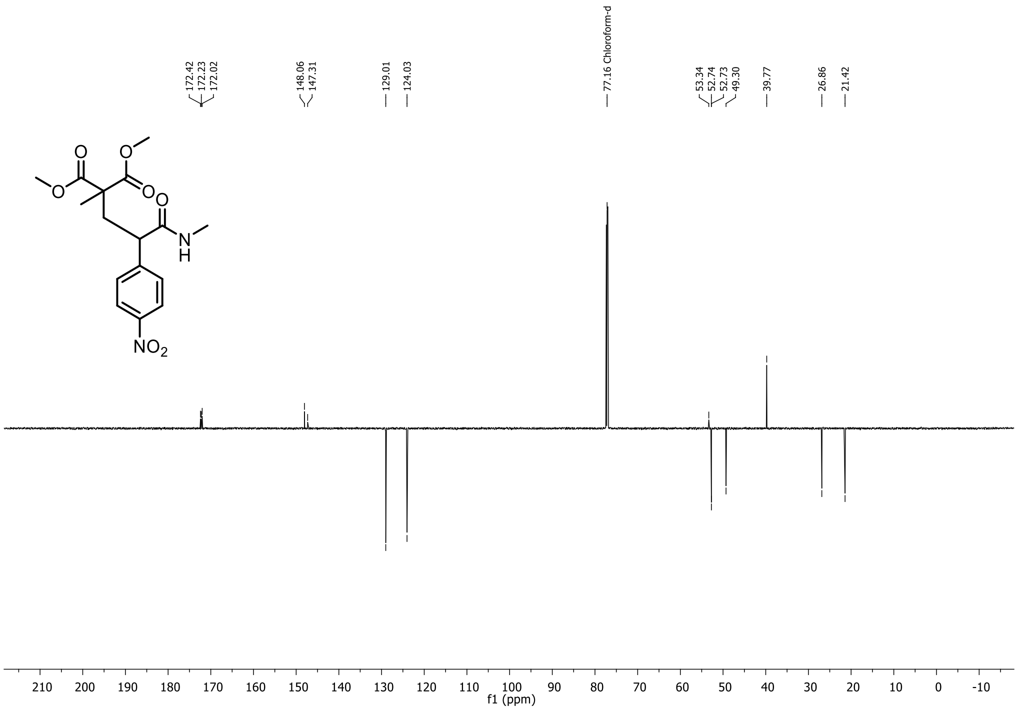

4b: Diethyl 2-benzyl-2-(3-(methylamino)-2-(4-nitrophenyl)-3-oxopropyl)malonate

^{1}H NMR (600 MHz, $CDCl_3$):

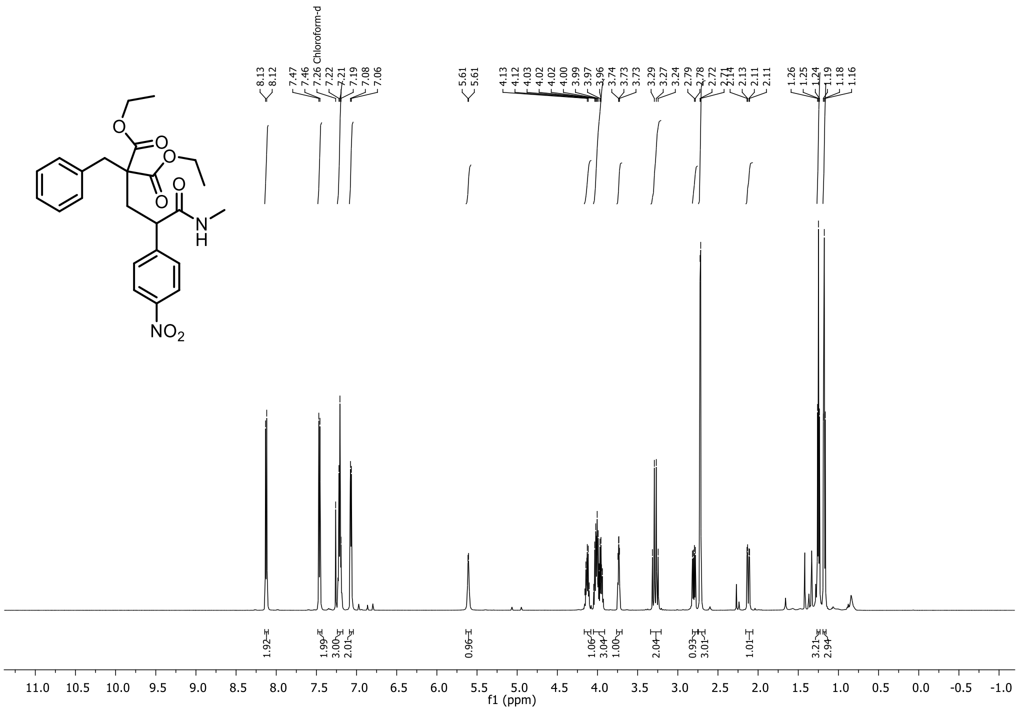

^{13}C NMR (151 MHz, $CDCl_3$):

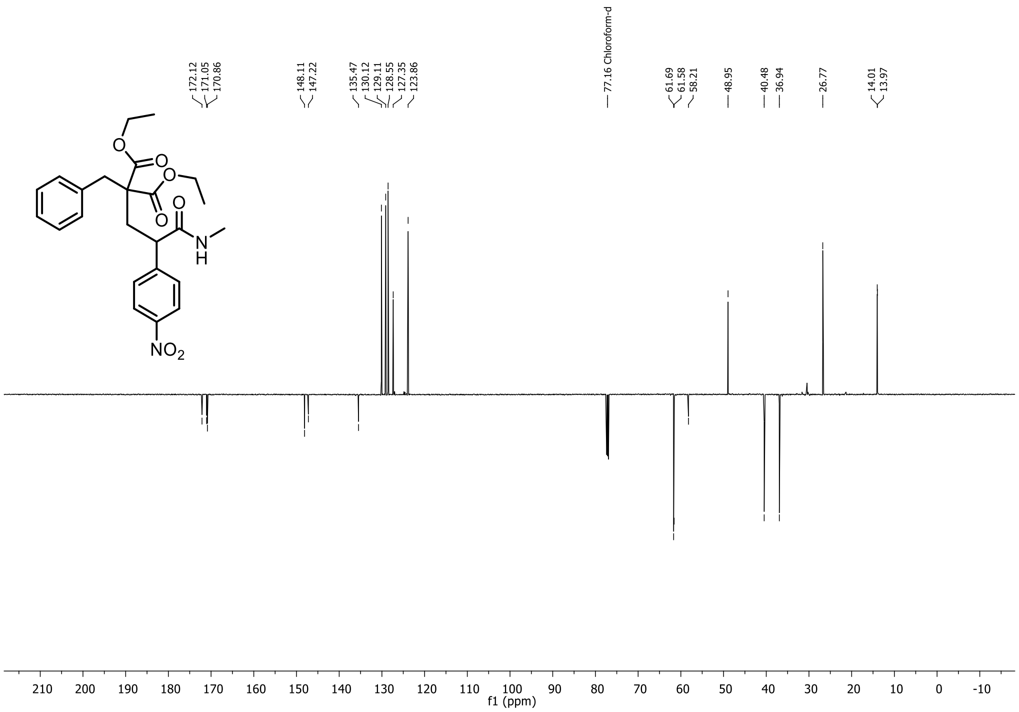

4c: Dimethyl 2-(3-(methylamino)-2-(4-nitrophenyl)-3-oxopropyl)malonate

^{1}H NMR (700 MHz, $CDCl_3$):

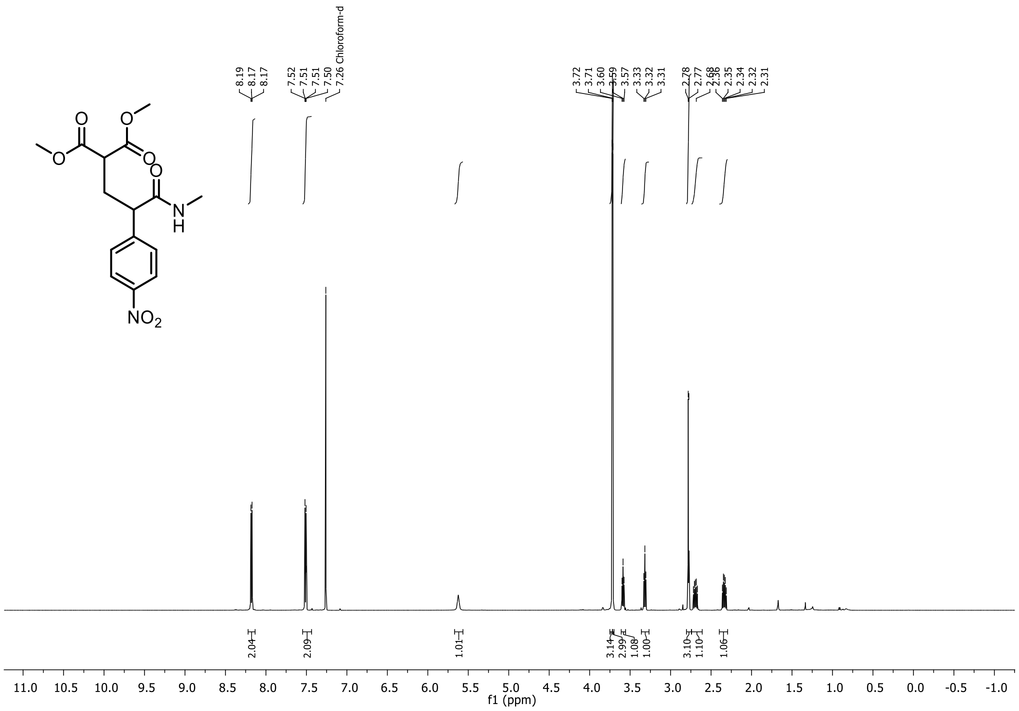

^{13}C NMR (176 MHz, $CDCl_3$):

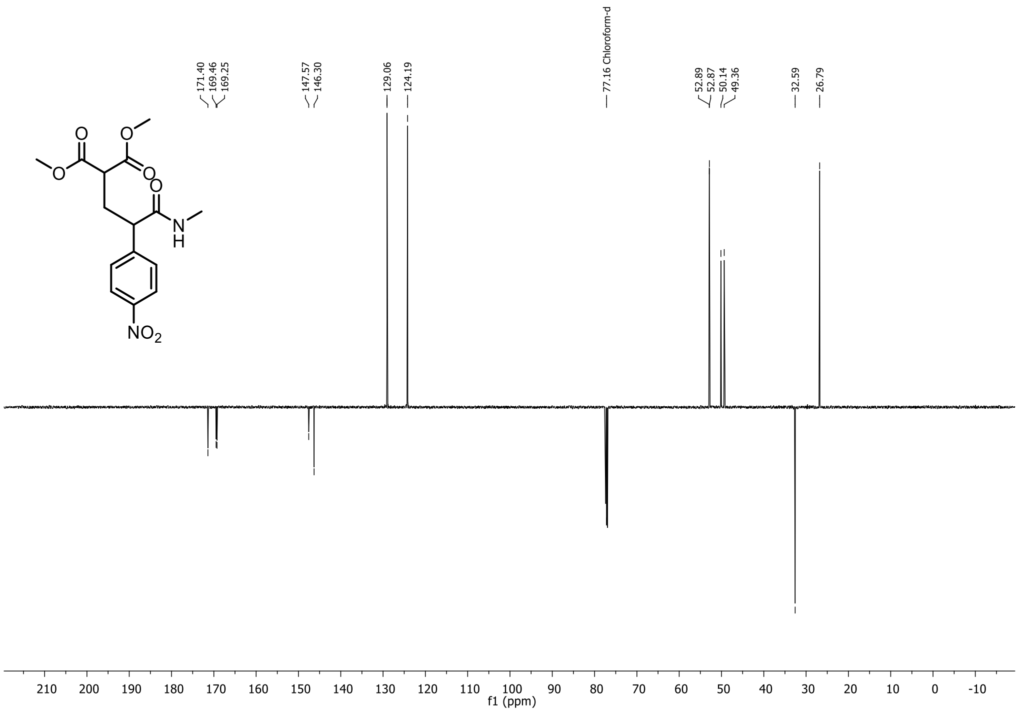

4d: 4-acetyl-*N*,4-dimethyl-2-(4-nitrophenyl)-5-oxohexanamide

^{1}H NMR (600 MHz, $CDCl_3$):

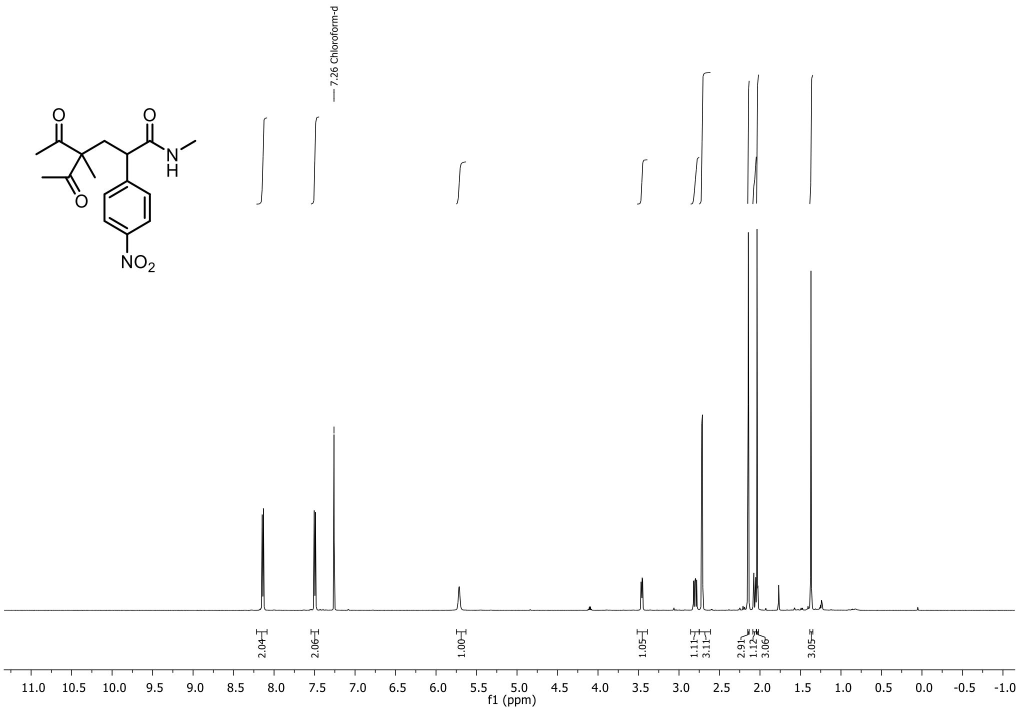

^{13}C NMR (151 MHz, $CDCl_3$):

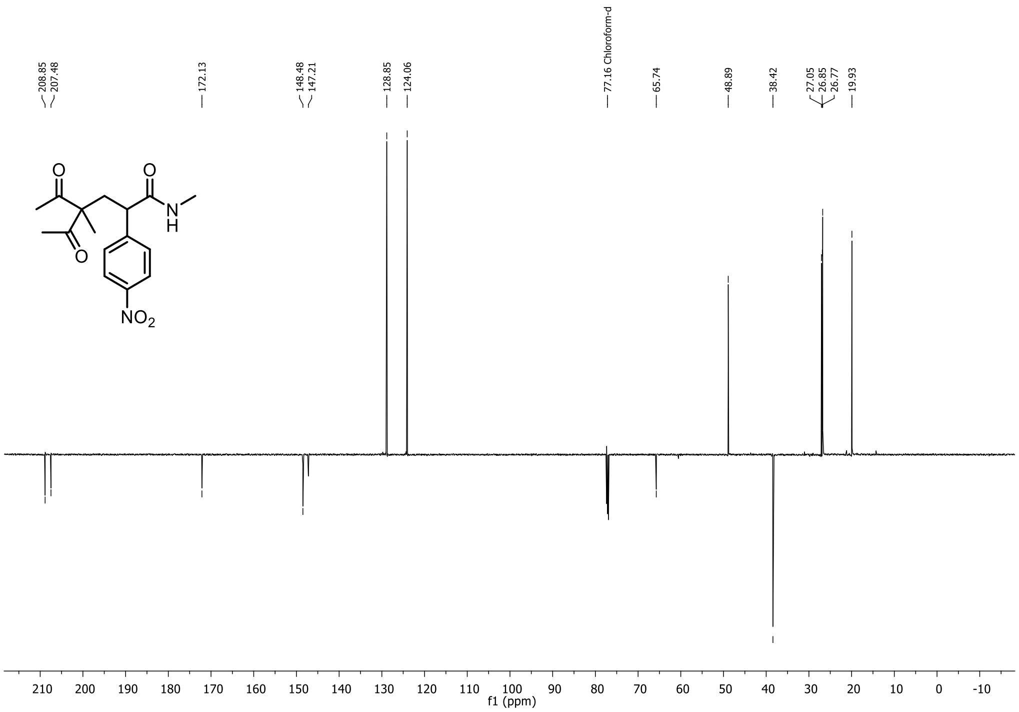

4e: *N*-methyl-2-(4-nitrophenyl)-4,4-bis(phenylsulfonyl)butanamide

^{1}H NMR (700 MHz, $CDCl_3$):

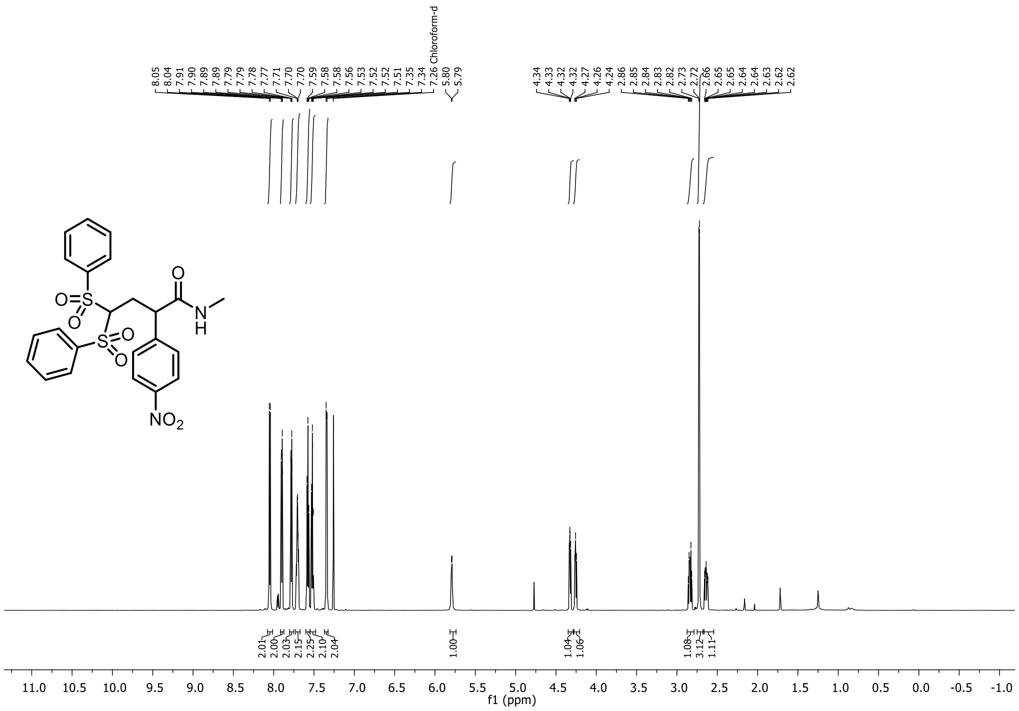

^{13}C NMR (176 MHz, $CDCl_3$):

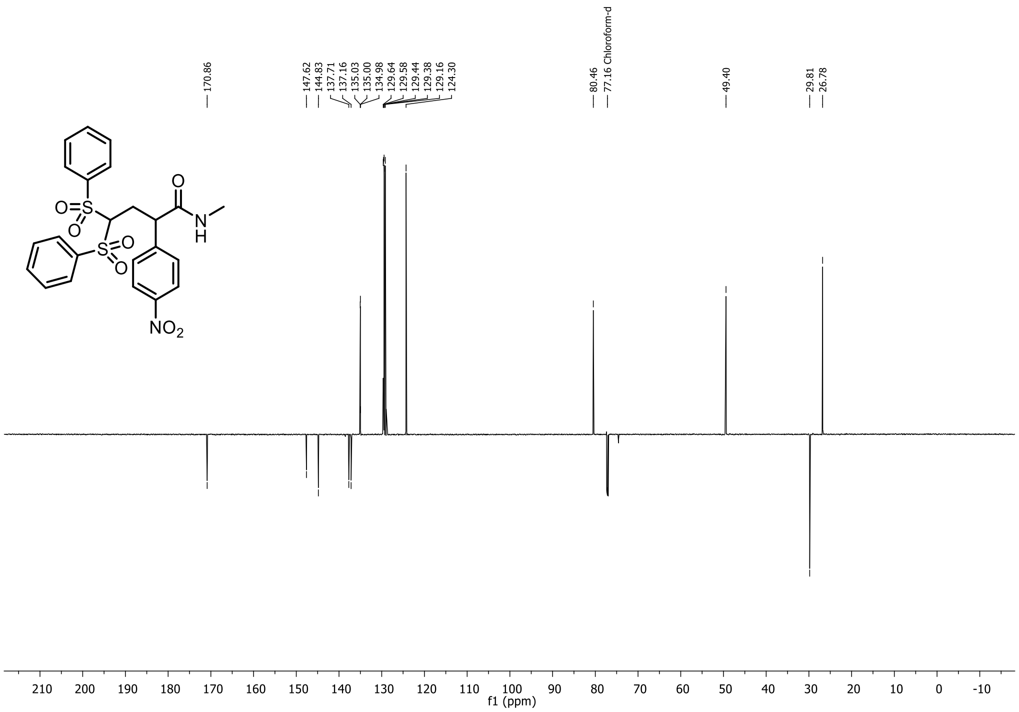

^{1}H NMR (600 MHz, $CDCl_3$):

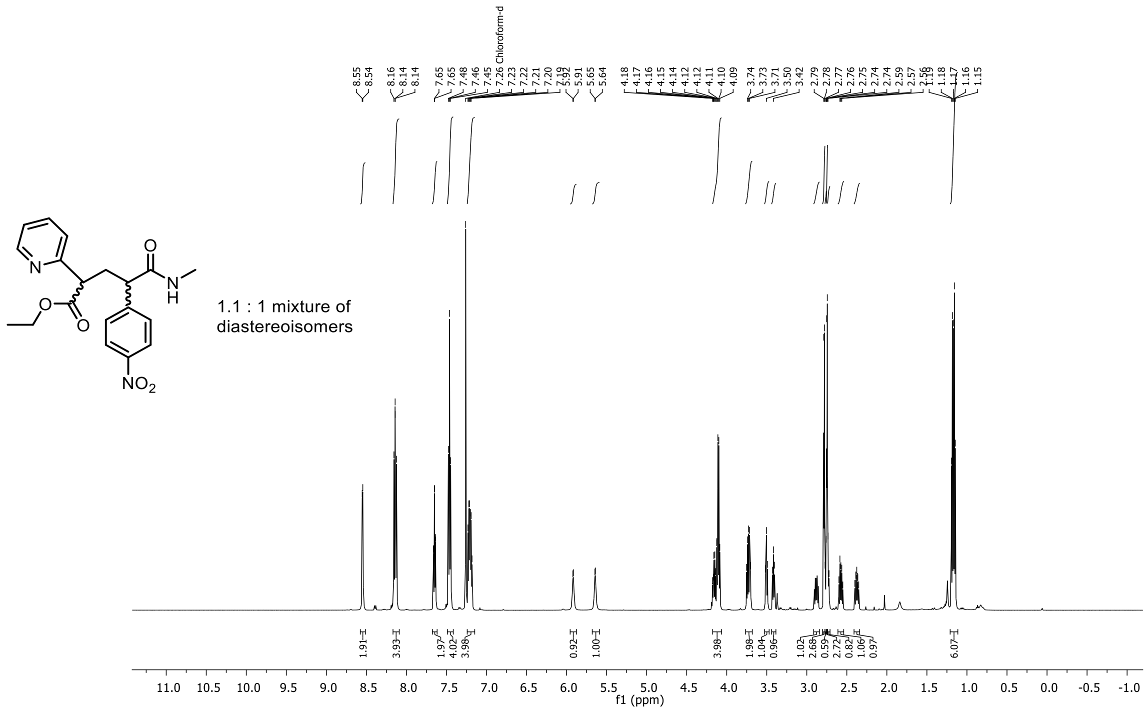

^{13}C NMR (151 MHz, $CDCl_3$):

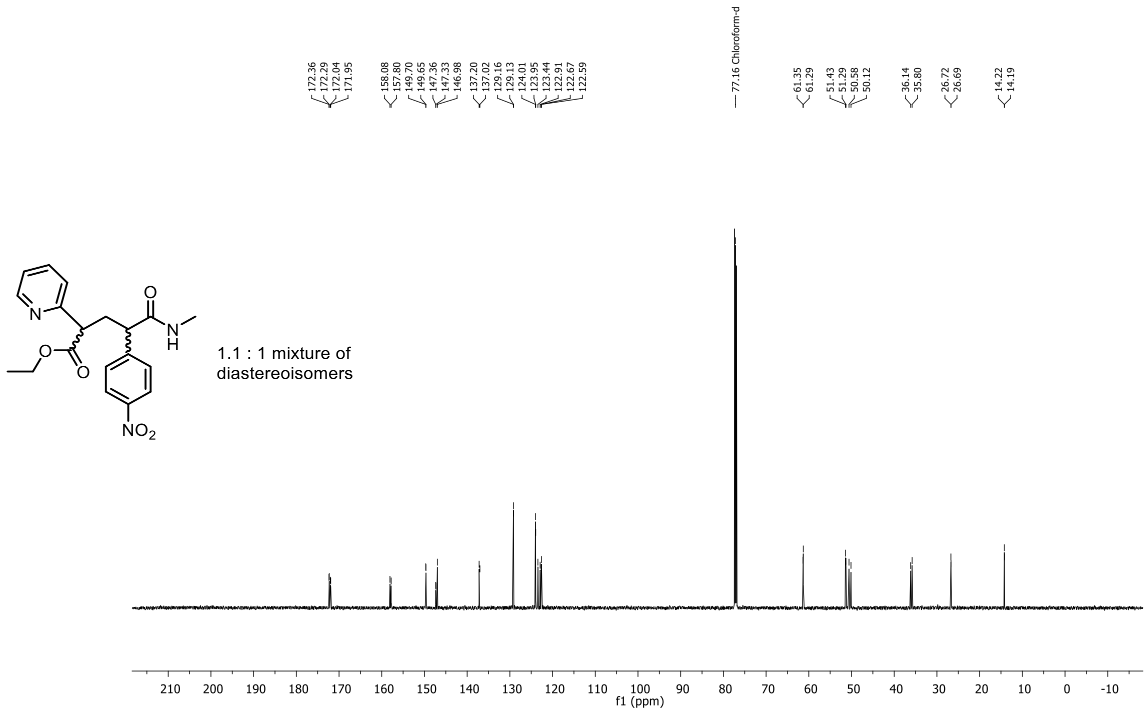

4g: Dimethyl 2-(3-(isopropylamino)-2-(4-nitrophenyl)-3-oxopropyl)-2-methylmalonate

^{1}H NMR (400 MHz, $CDCl_3$):

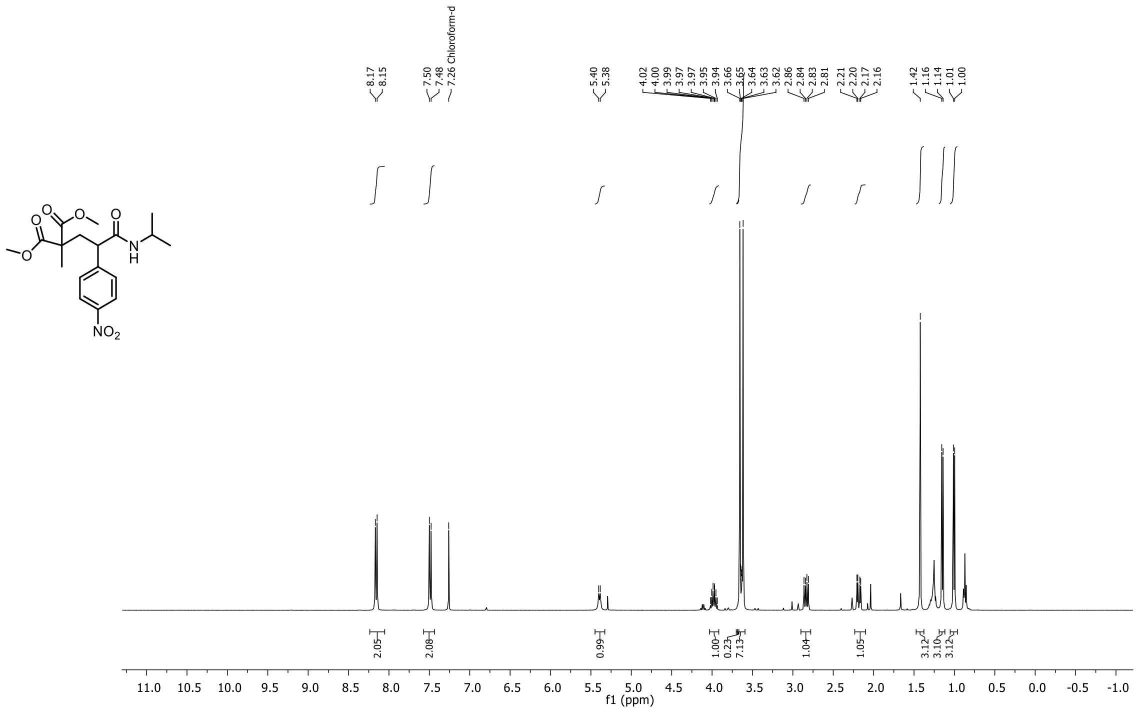

^{13}C NMR (101 MHz, $CDCl_3$):

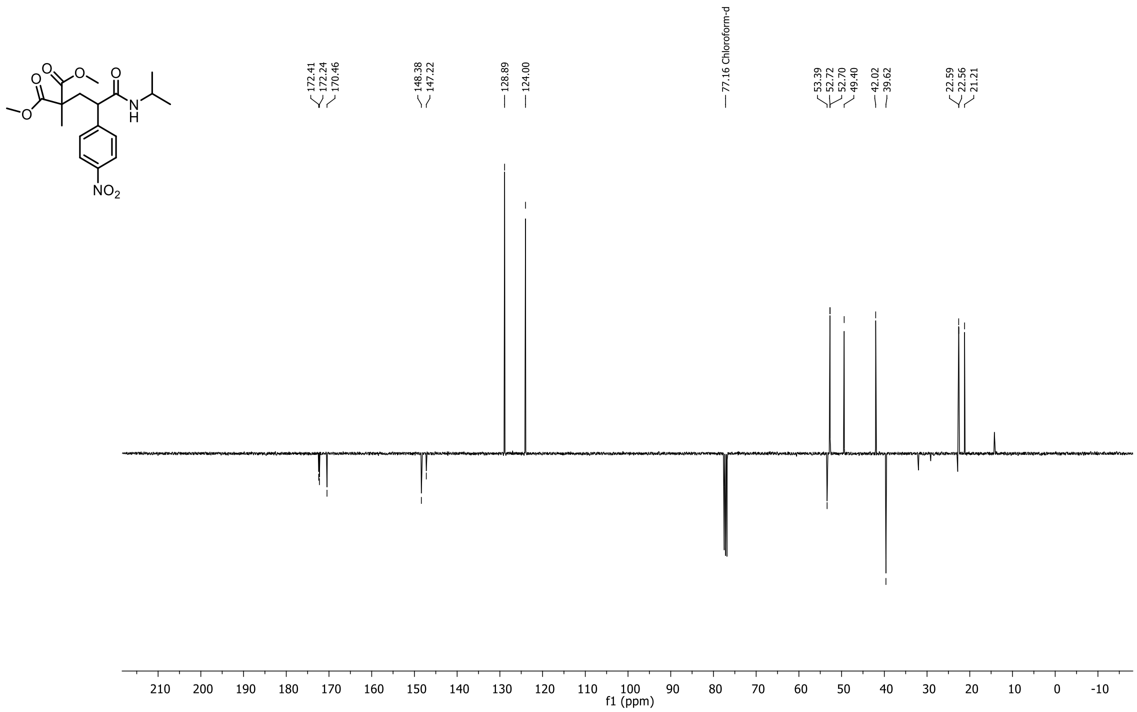

4h: Dimethyl 2-(3-(*tert*-butylamino)-2-(4-nitrophenyl)-3-oxopropyl)-2-methylmalonate

^{1}H NMR (400 MHz, $CDCl_3$):

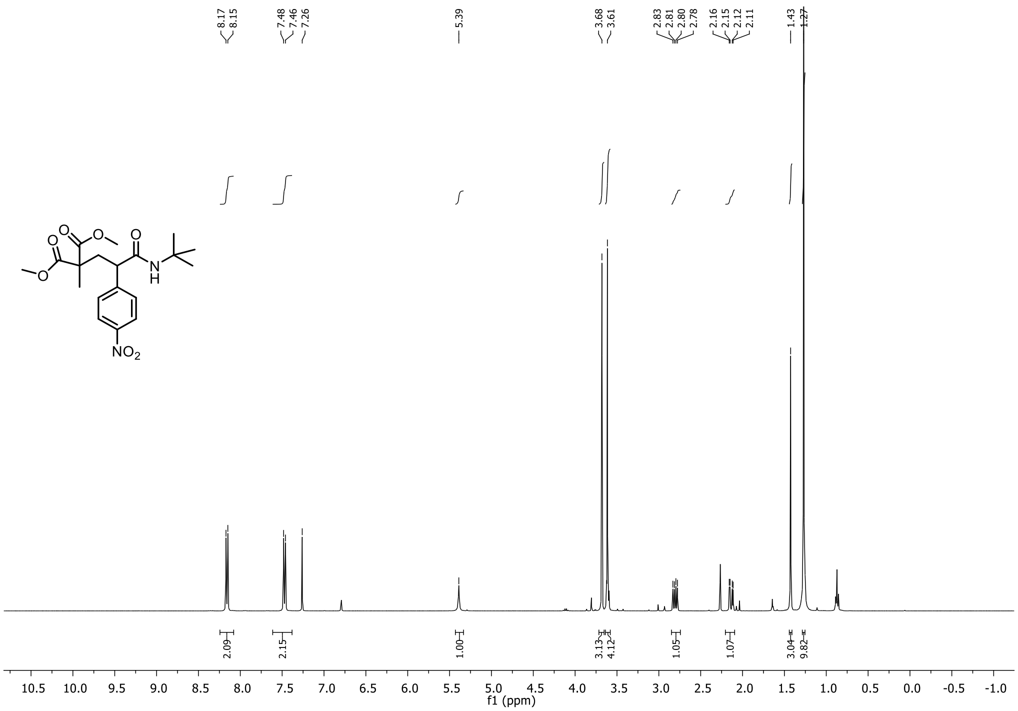

^{13}C NMR (101 MHz, $CDCl_3$):

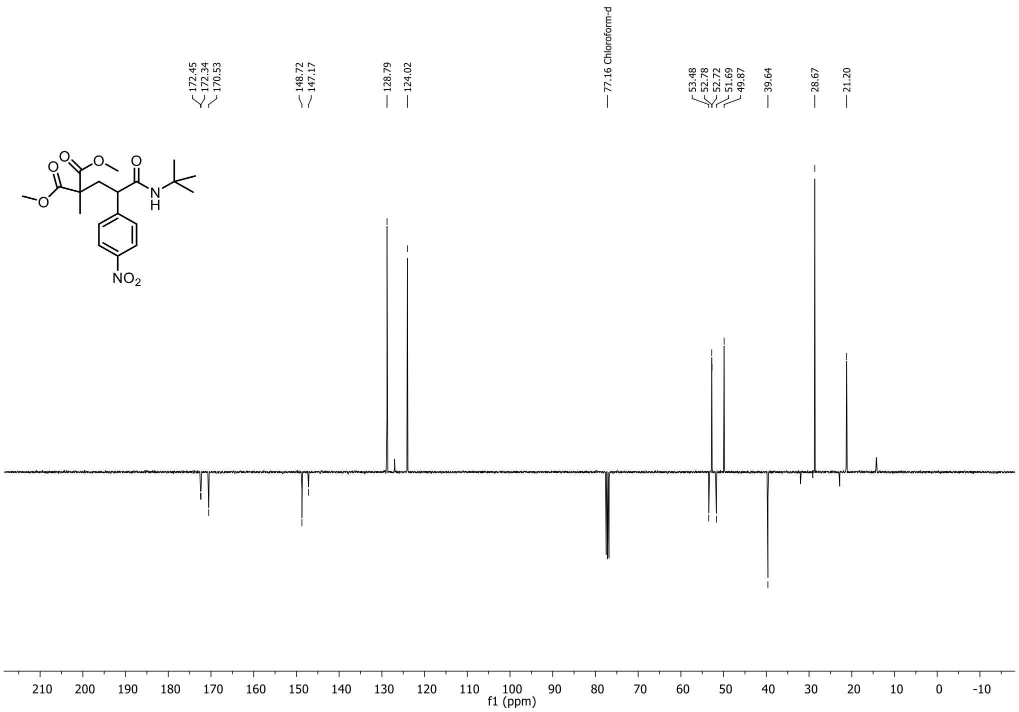

4i: Dimethyl 2-(3-(benzylamino)-2-(4-nitrophenyl)-3-oxopropyl)-2-methylmalonate

¹H NMR (400 MHz, $CDCl_3$):

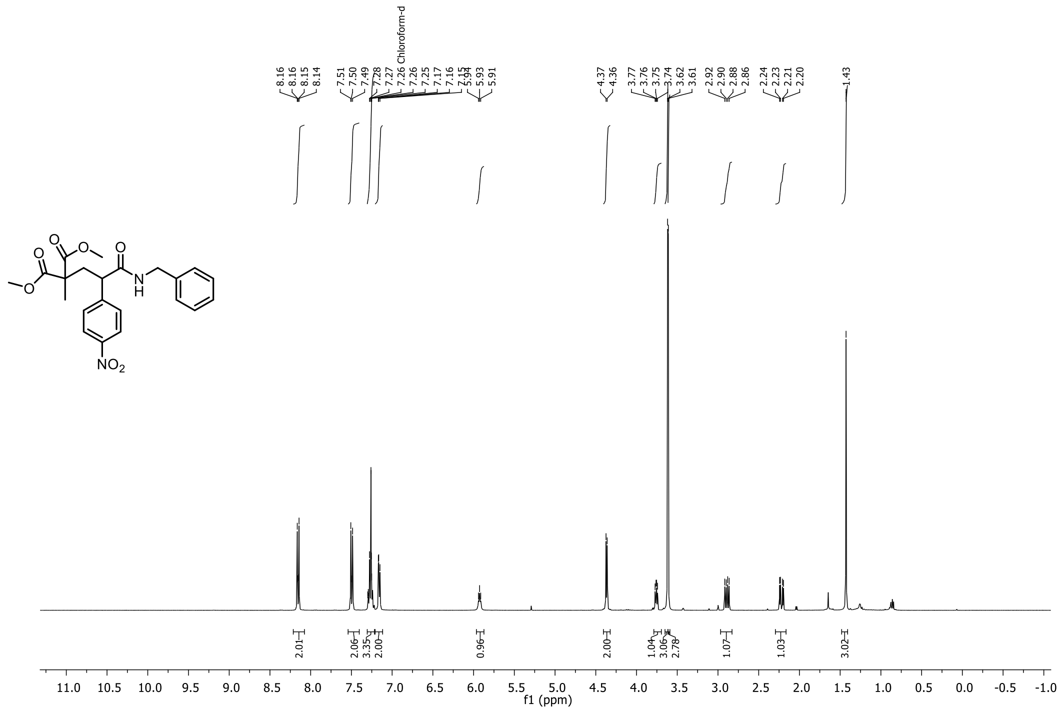

¹³C NMR (101 MHz, $CDCl_3$):

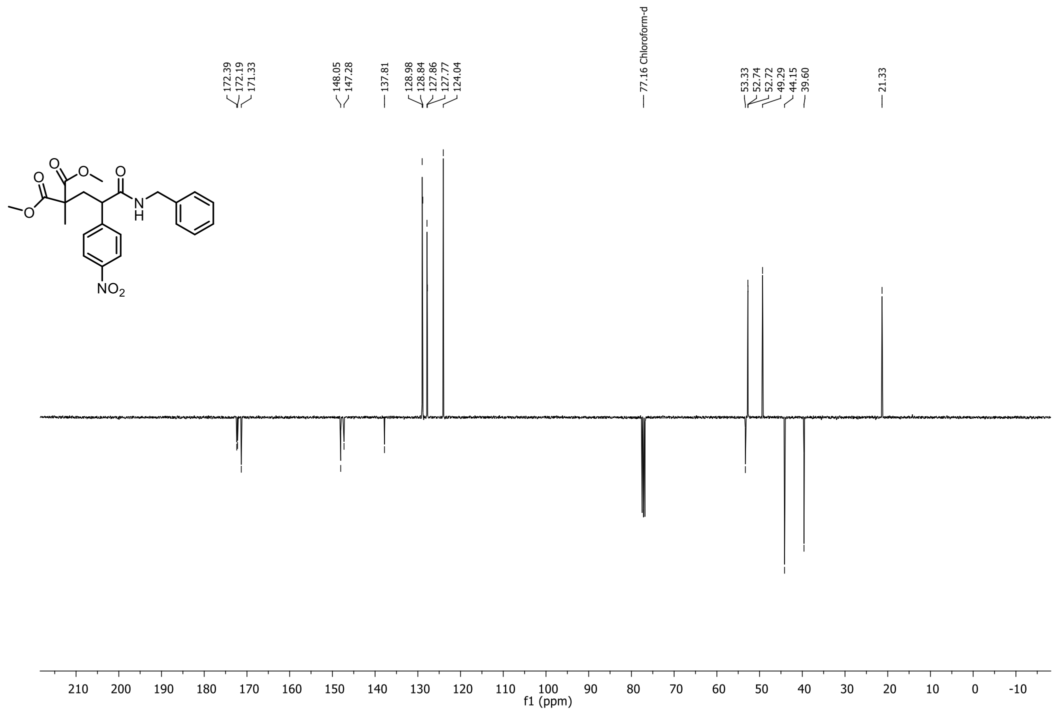

4j: Dimethyl 2-(3-(allylamino)-2-(4-nitrophenyl)-3-oxopropyl)-2-methylmalonate

^{1}H NMR (600 MHz, $CDCl_3$):

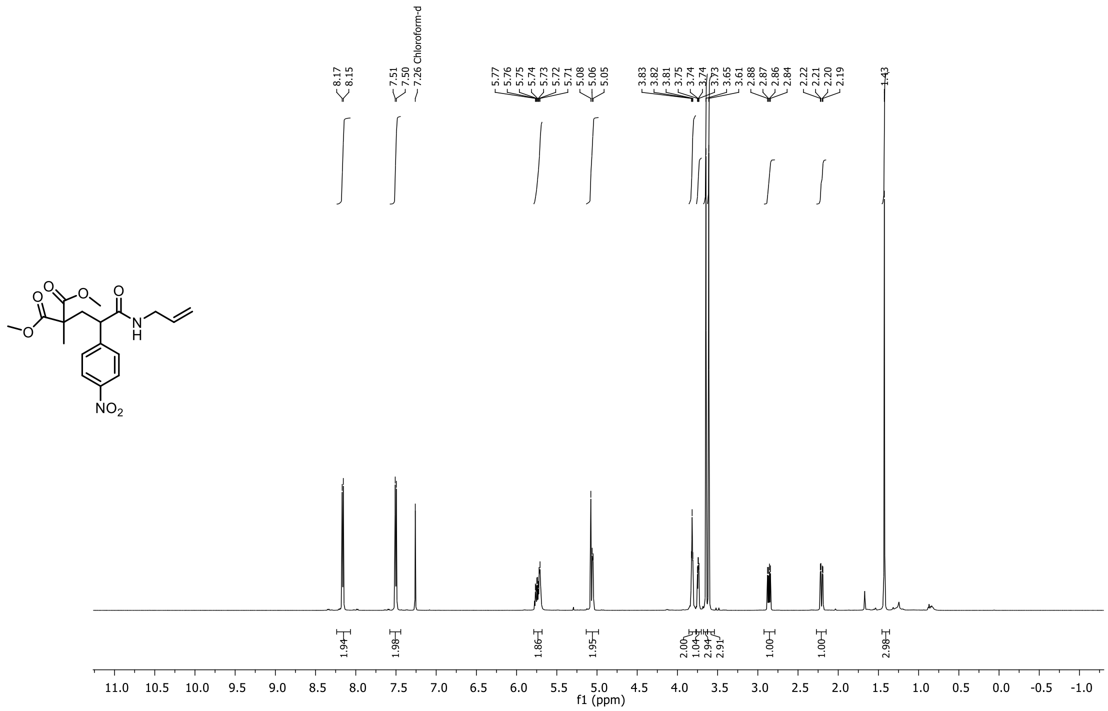

^{13}C NMR (156 MHz, $CDCl_3$):

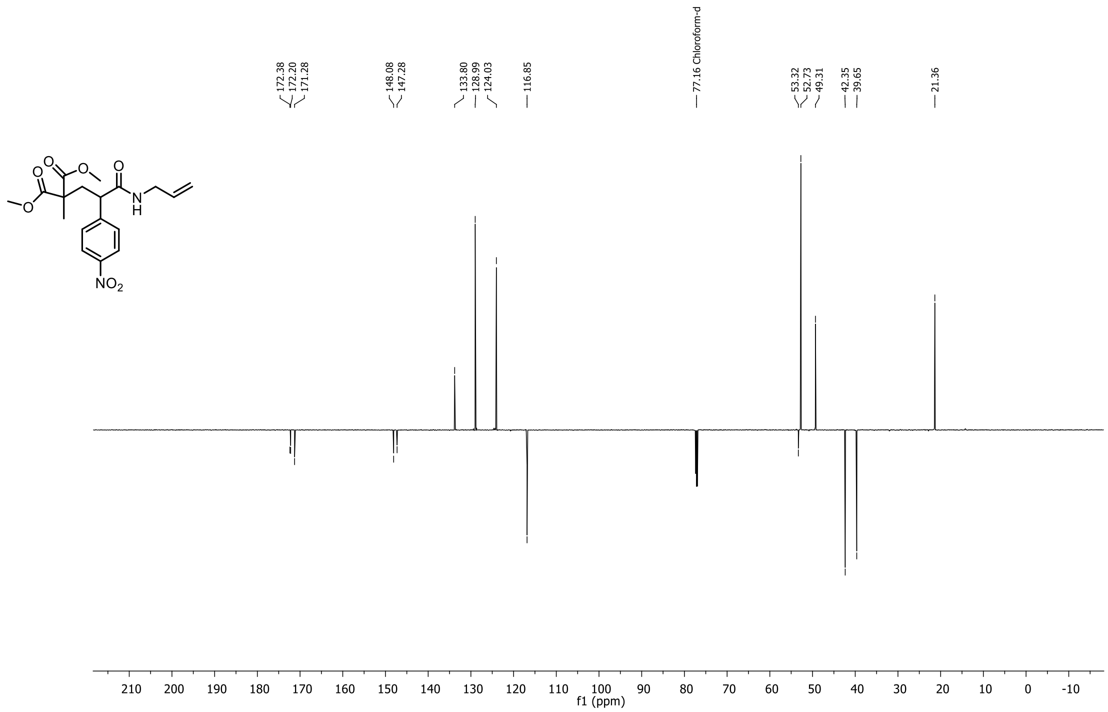

4k: Dimethyl 2-(3-((4,4-dimethoxybutyl)amino)-2-(4-nitrophenyl)-3-oxopropyl)-2-methylmalonate

^{1}H NMR (400 MHz, $CDCl_3$):

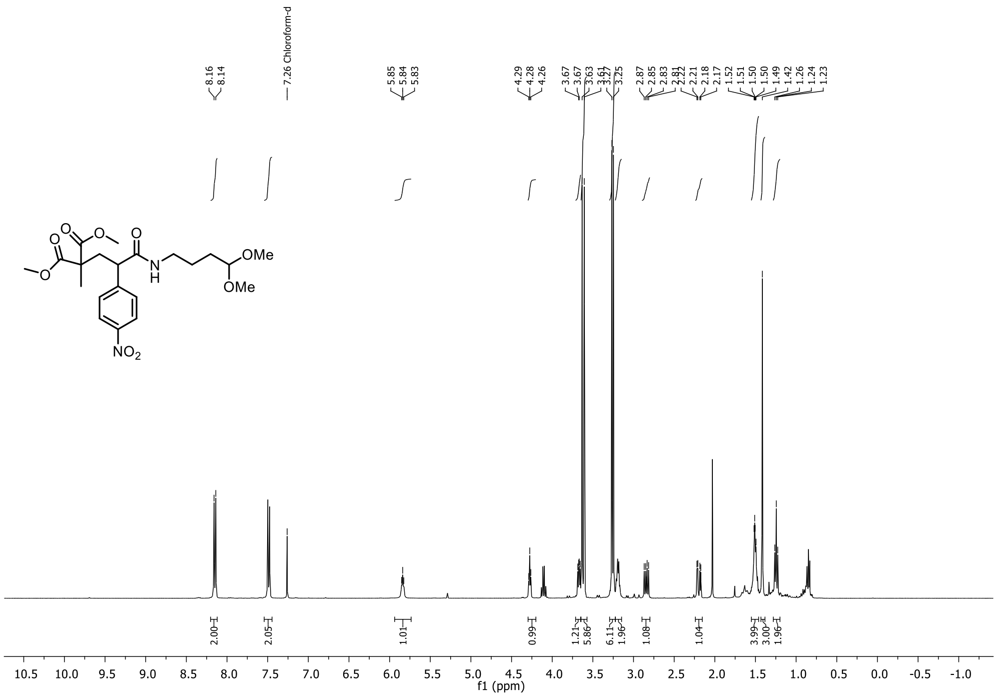

^{13}C NMR (101 MHz, $CDCl_3$):

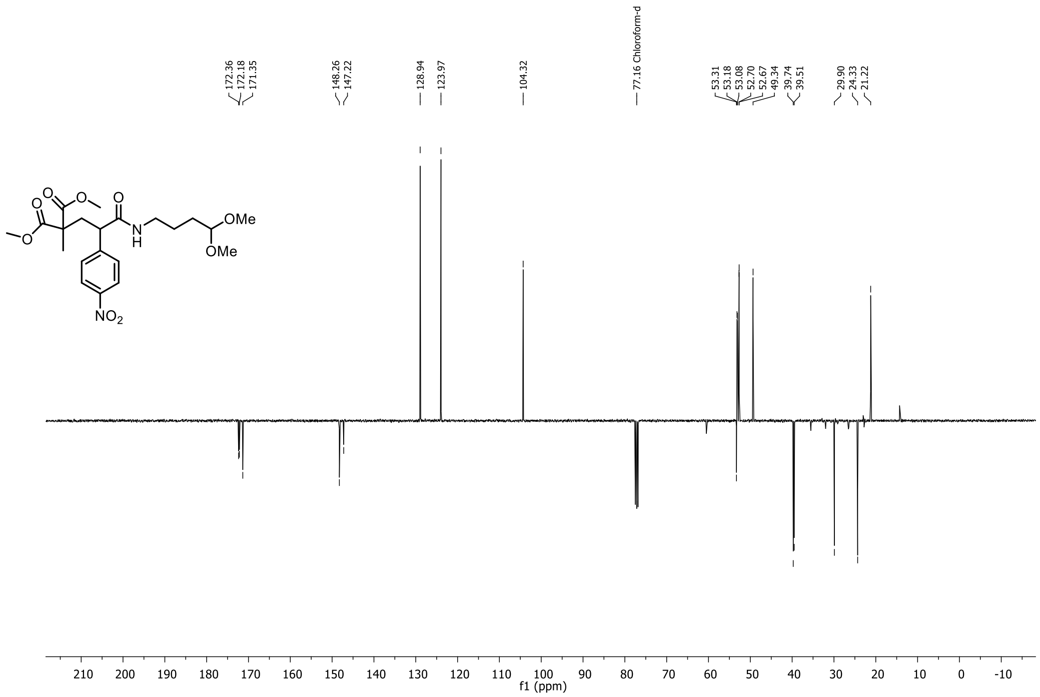

4l: Dimethyl 2-methyl-2-(2-methyl-3-(methylamino)-2-(4-nitrophenyl)-3-oxopropyl)malonate

^{1}H NMR (600 MHz, $CDCl_3$):

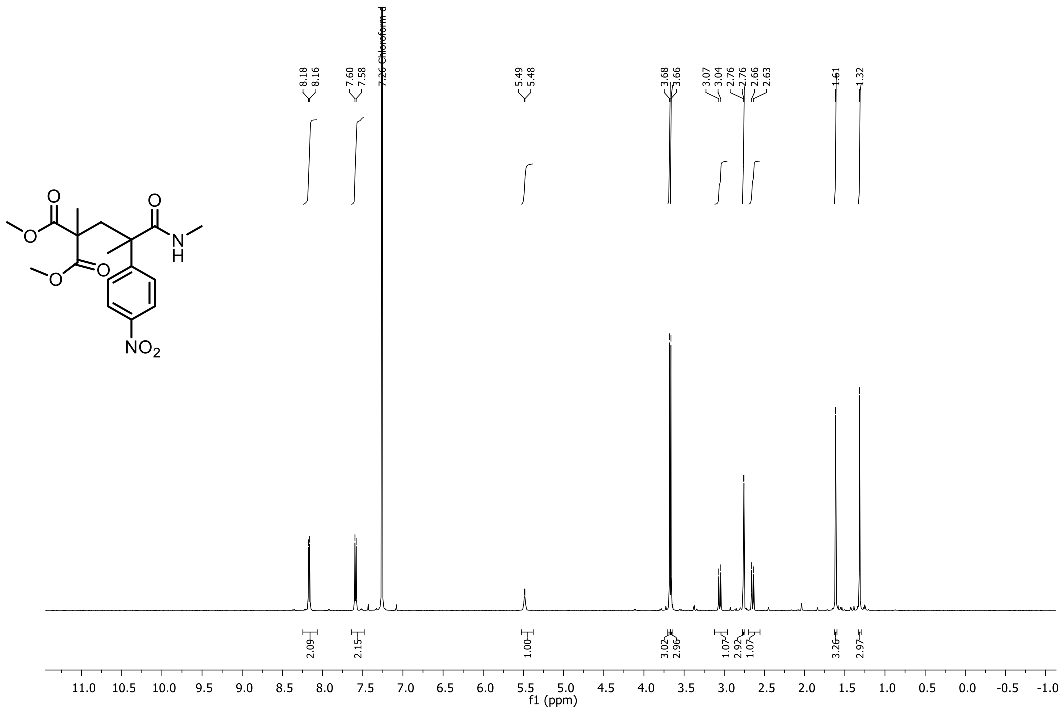

^{13}C NMR (151 MHz, $CDCl_3$):

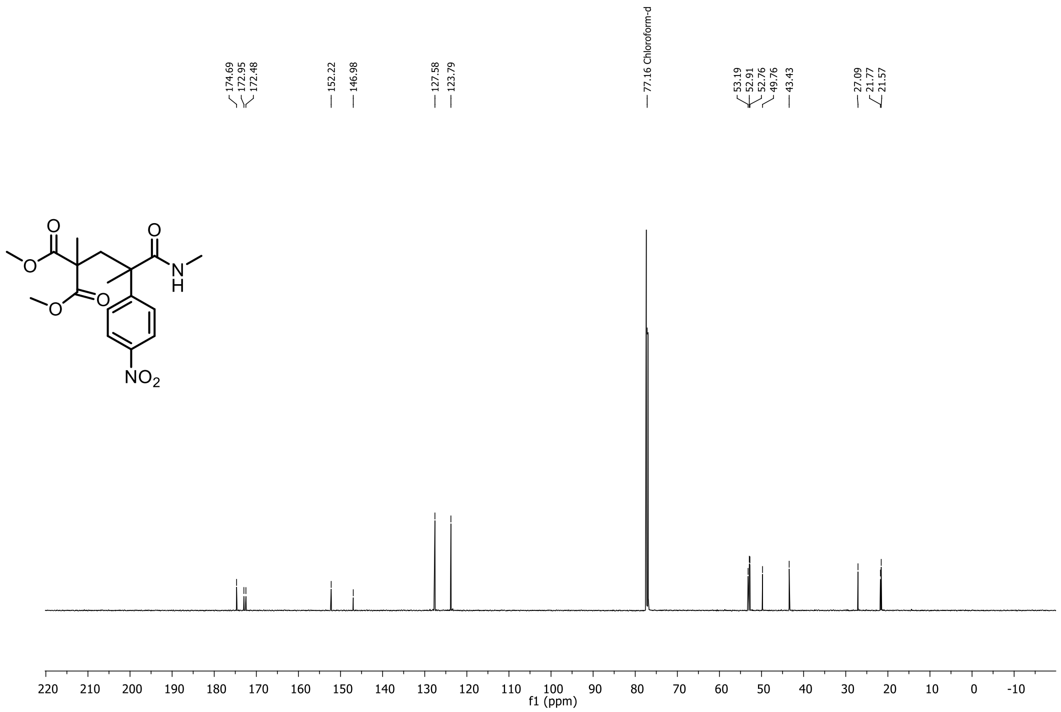

4m: Dimethyl 2-(2-(4-cyano-2-(trifluoromethyl)phenyl)-3-(methylamino)-3-oxopropyl)-2-methylmalonate

^{1}H NMR (600 MHz, $CDCl_3$):

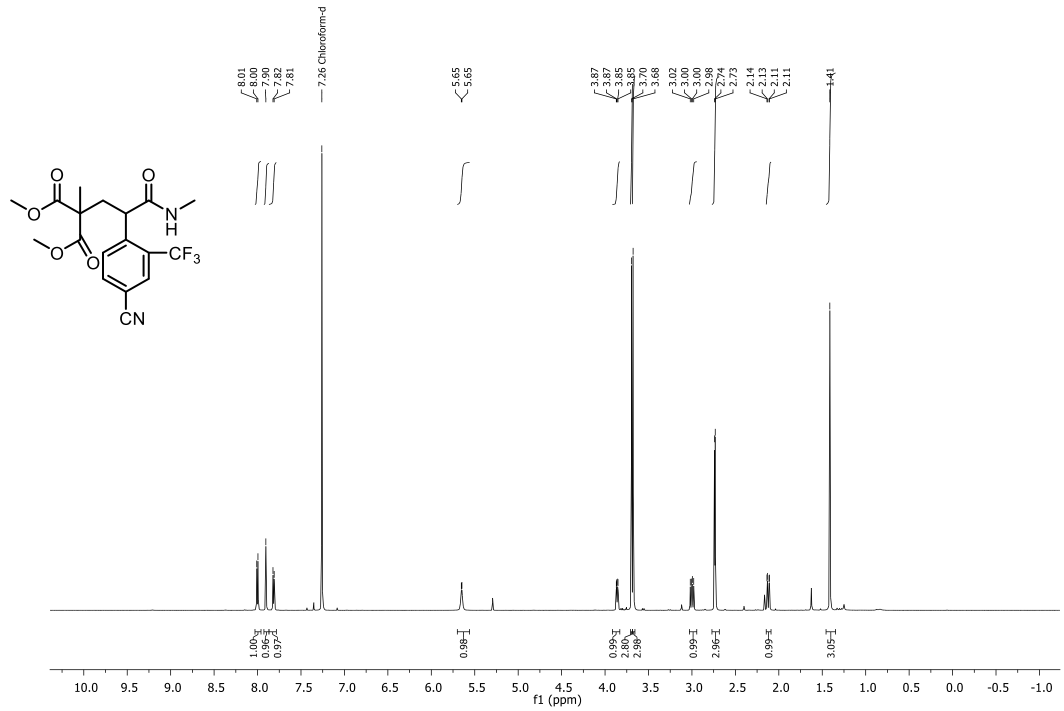

^{13}C NMR (151 MHz, $CDCl_3$):

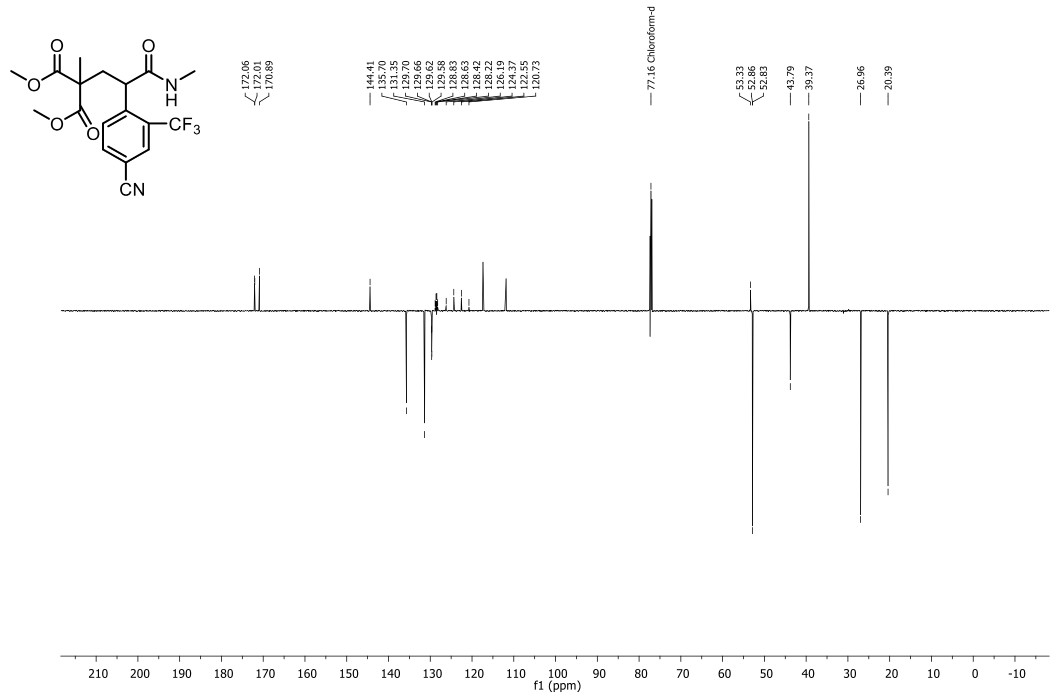

^{19}F NMR (565 MHz, $CDCl_3$):

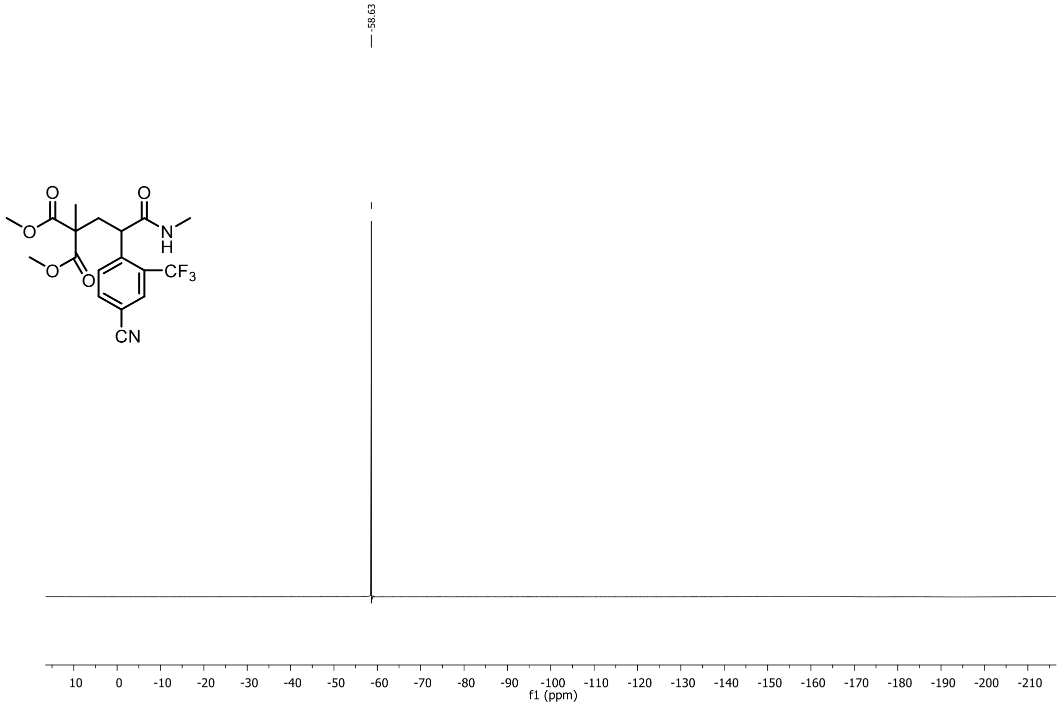

4n: Dimethyl 2-(2-(4-cyanophenyl)-3-(methylamino)-3-oxopropyl)-2-methylmalonate

^{1}H NMR (600 MHz, $CDCl_3$):

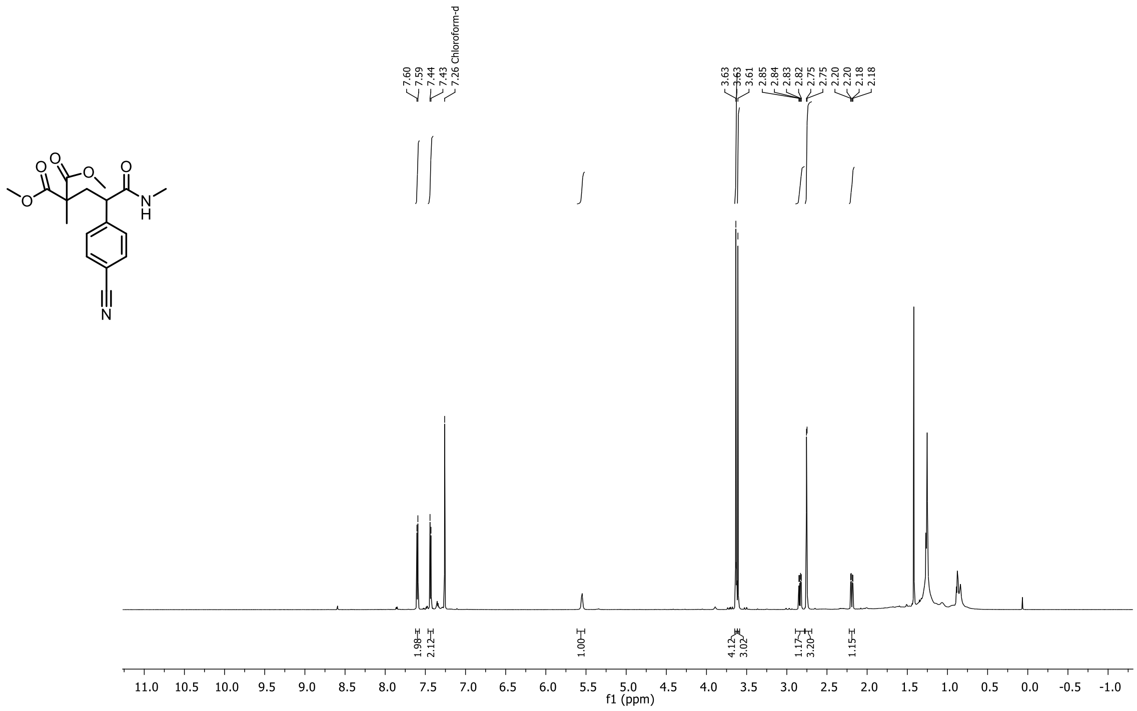

^{13}C NMR (151 MHz, $CDCl_3$):

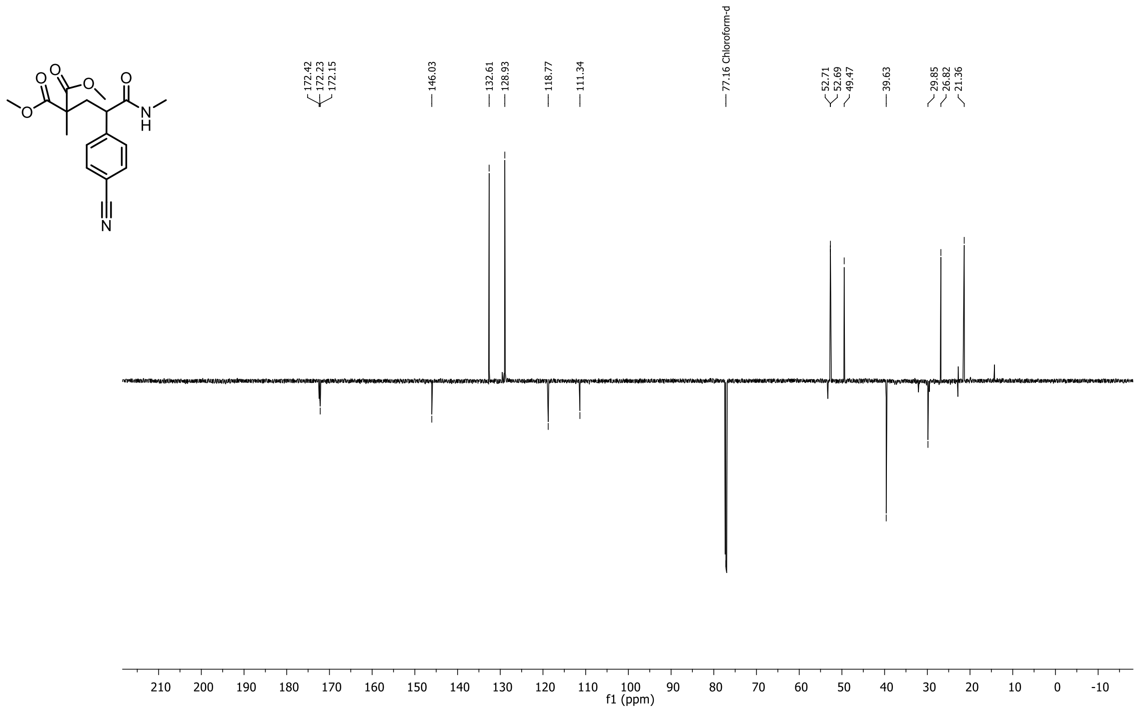

4o: Dimethyl 2-methyl-2-(3-(methylamino)-3-oxo-2-(5-(trifluoromethyl)pyridin-2-yl)propyl)malonate

^{1}H NMR (600 MHz, $CDCl_3$):

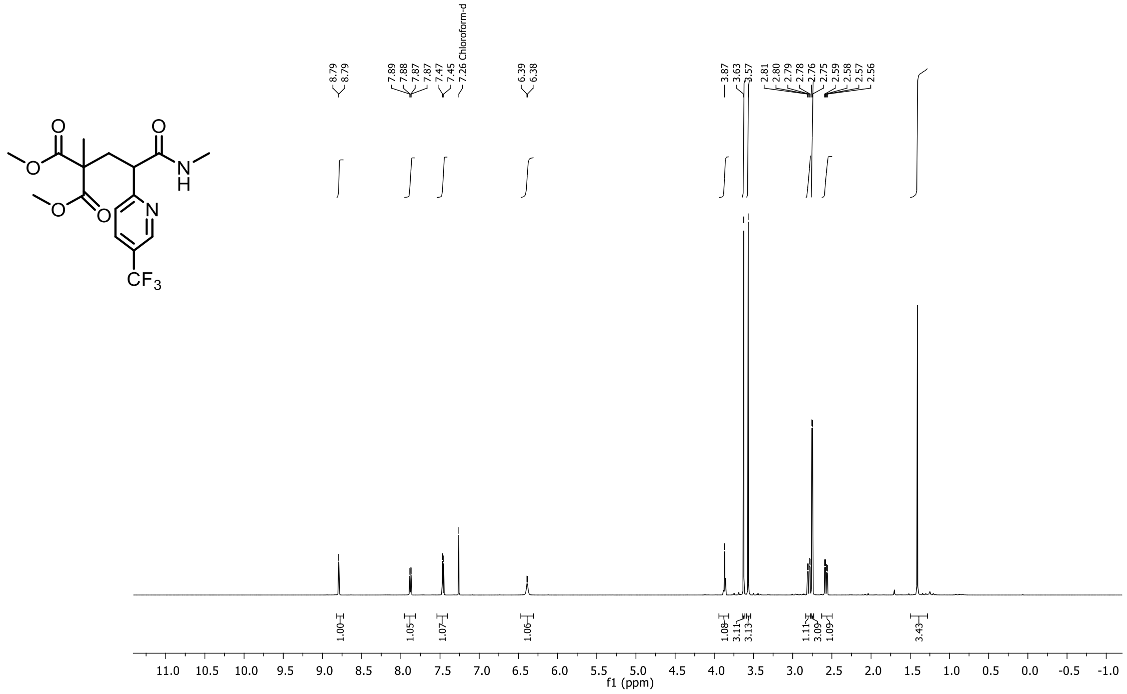

^{13}C NMR (151 MHz, $CDCl_3$):

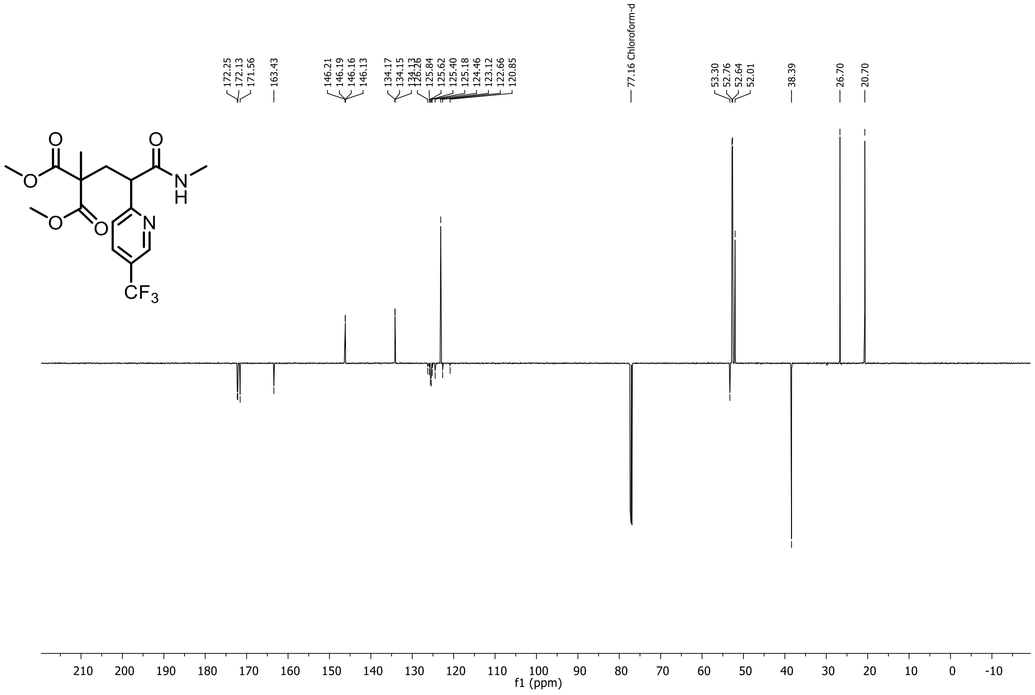

^{19}F NMR (376 MHz, $CDCl_3$):

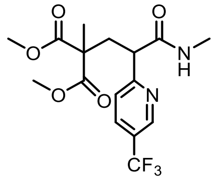

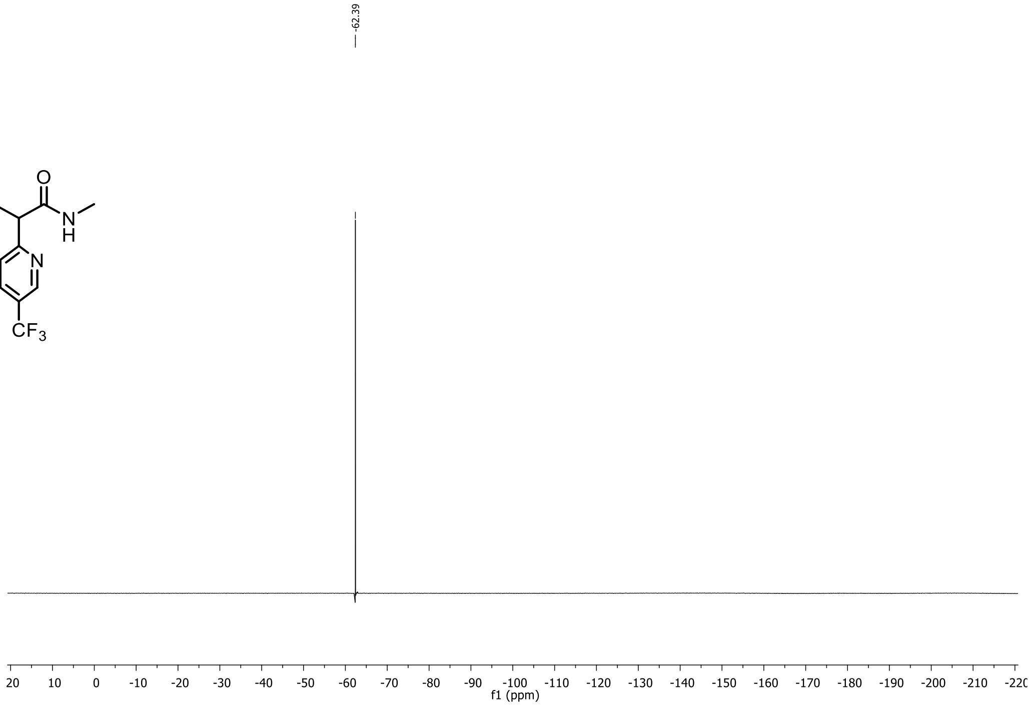

4p: Dimethyl (*E*)-2-methyl-2-(2-(methylcarbamoyl)-4-(4-nitrophenyl)but-3-en-1-yl)malonate

^{1}H NMR (600 MHz, $CDCl_3$):

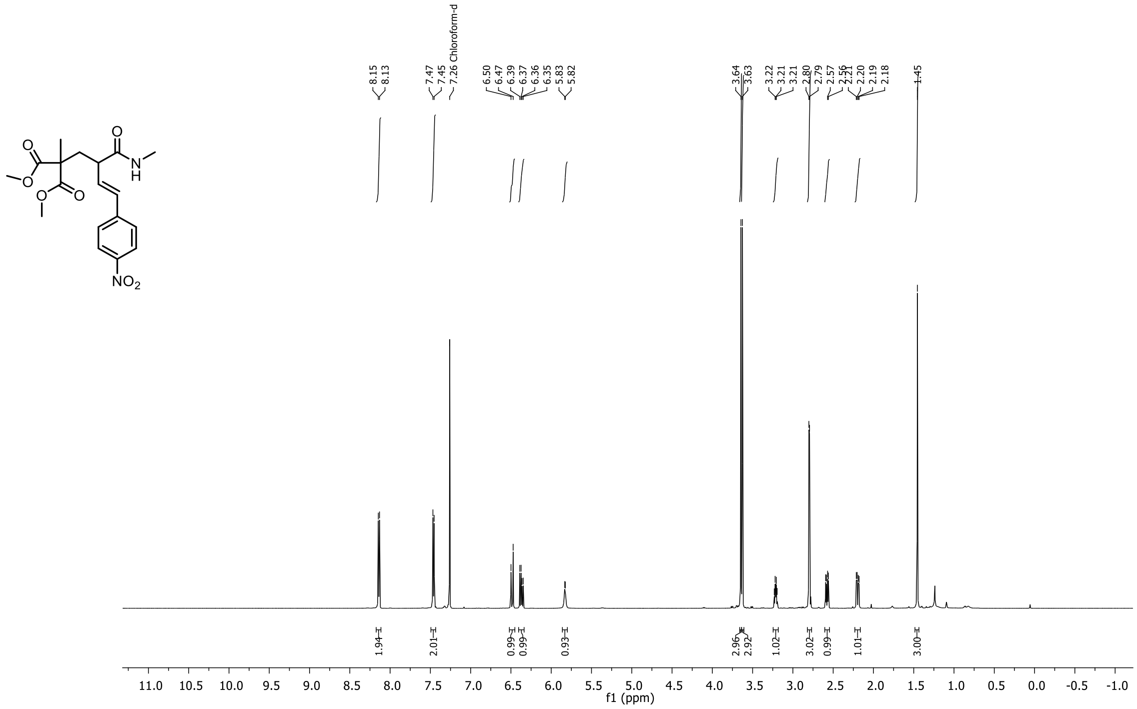

^{13}C NMR (151 MHz, $CDCl_3$):

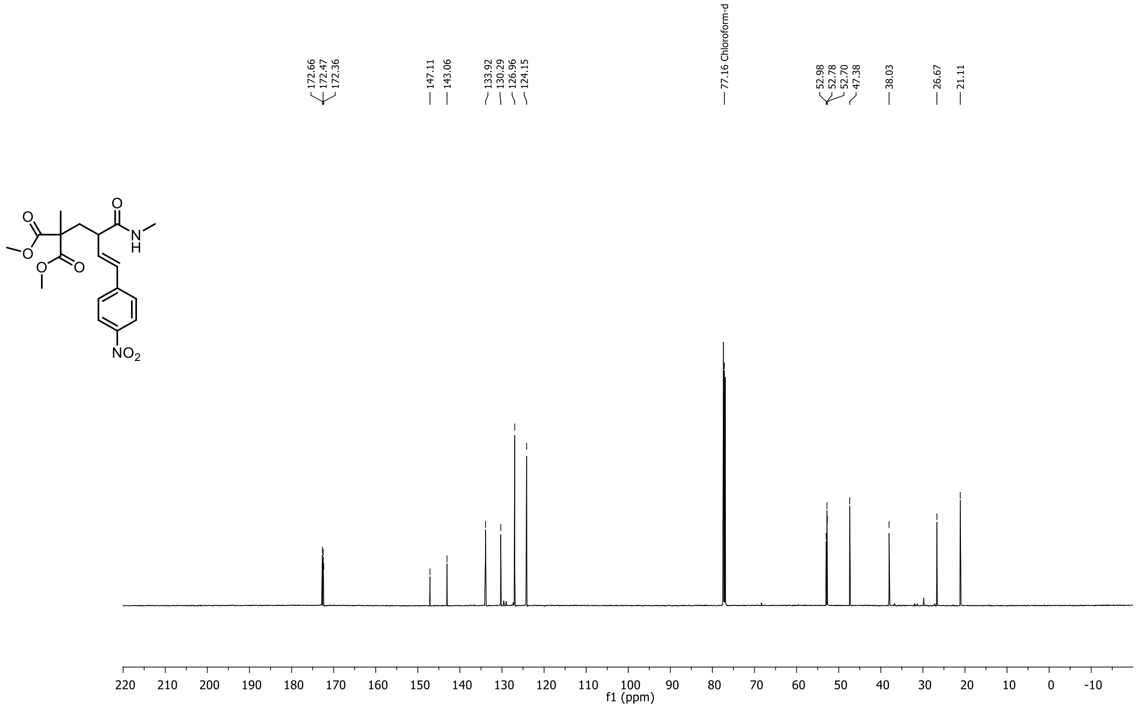

4q: Dimethyl (*E*)-2-methyl-2-(2-(methylcarbamoyl)-4-phenylbut-3-en-1-yl)malonate

^{1}H NMR (700 MHz, $CDCl_3$):

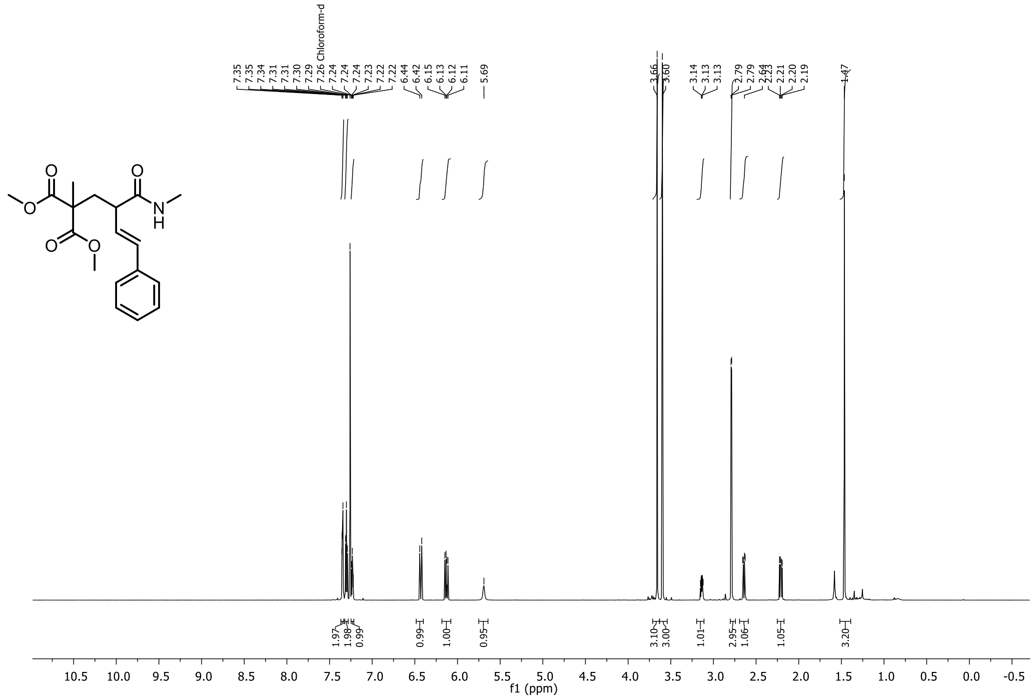

^{13}C NMR (176 MHz, $CDCl_3$):

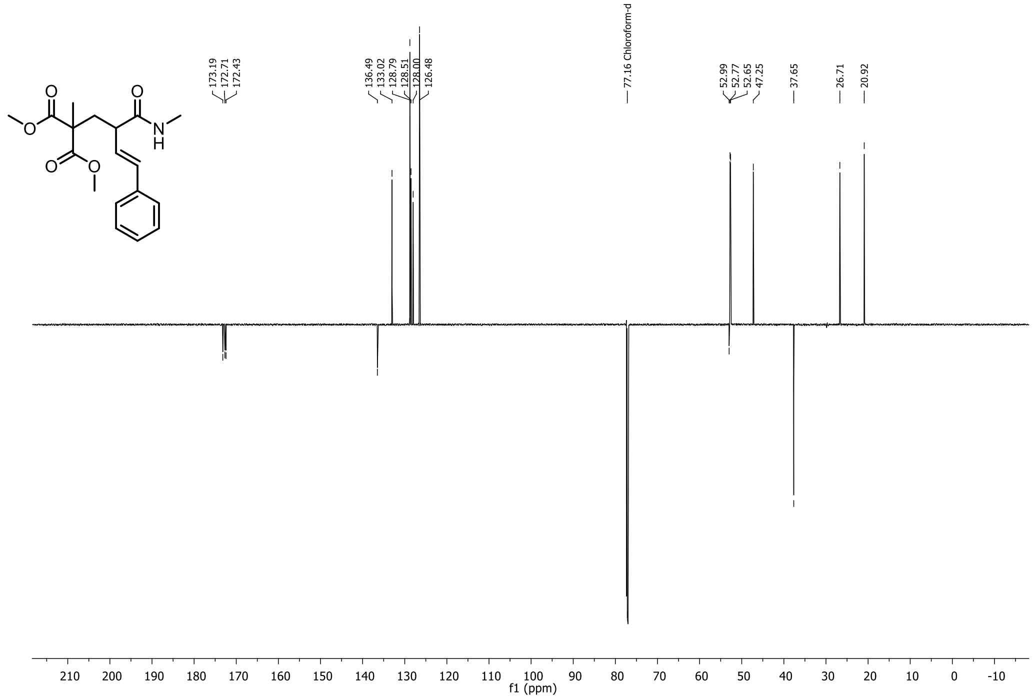

4r: Dimethyl (*E*)-2-methyl-2-(2-(methylcarbamoyl)-4-(naphthalen-2-yl)but-3-en-1-yl)malonate

^{1}H NMR (700 MHz, $CDCl_3$):

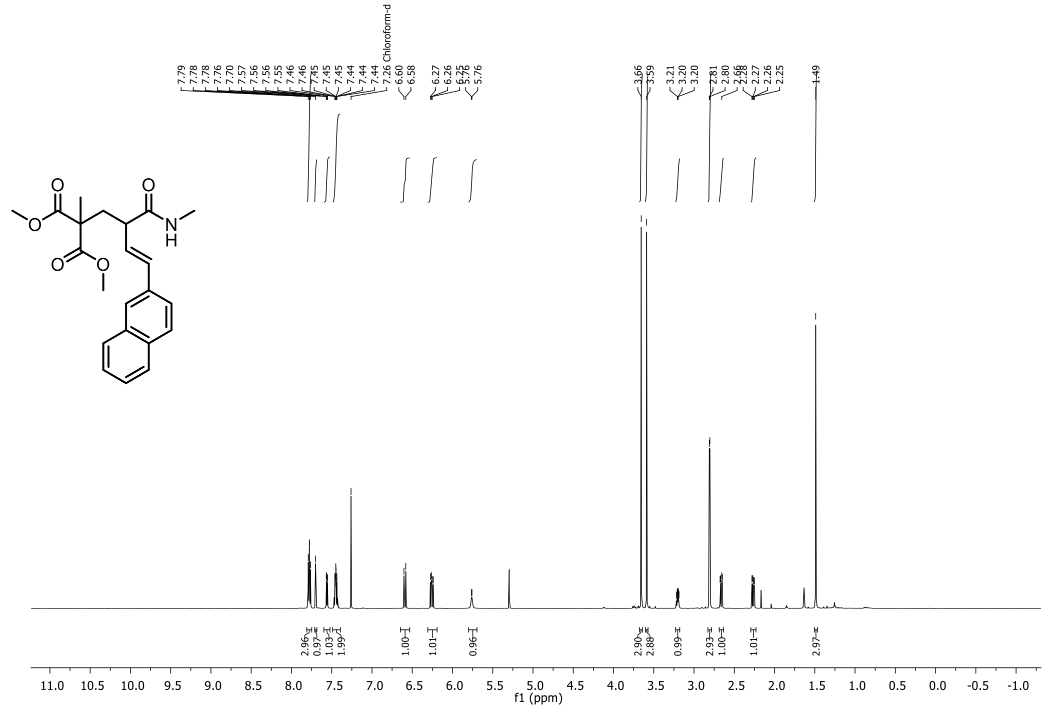

^{13}C NMR (176 MHz, $CDCl_3$):

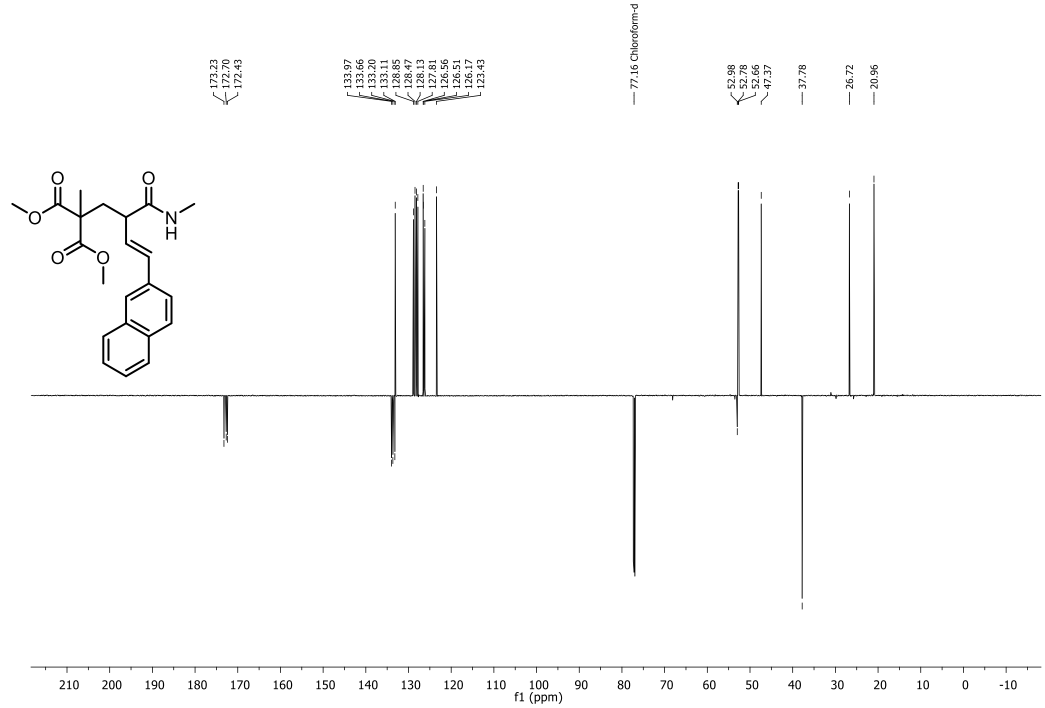

5a: 3-(diphenylphosphoryl)-*N*-methyl-2-(4-nitrophenyl)propanamide

^{1}H NMR (600 MHz, $CDCl_3$):

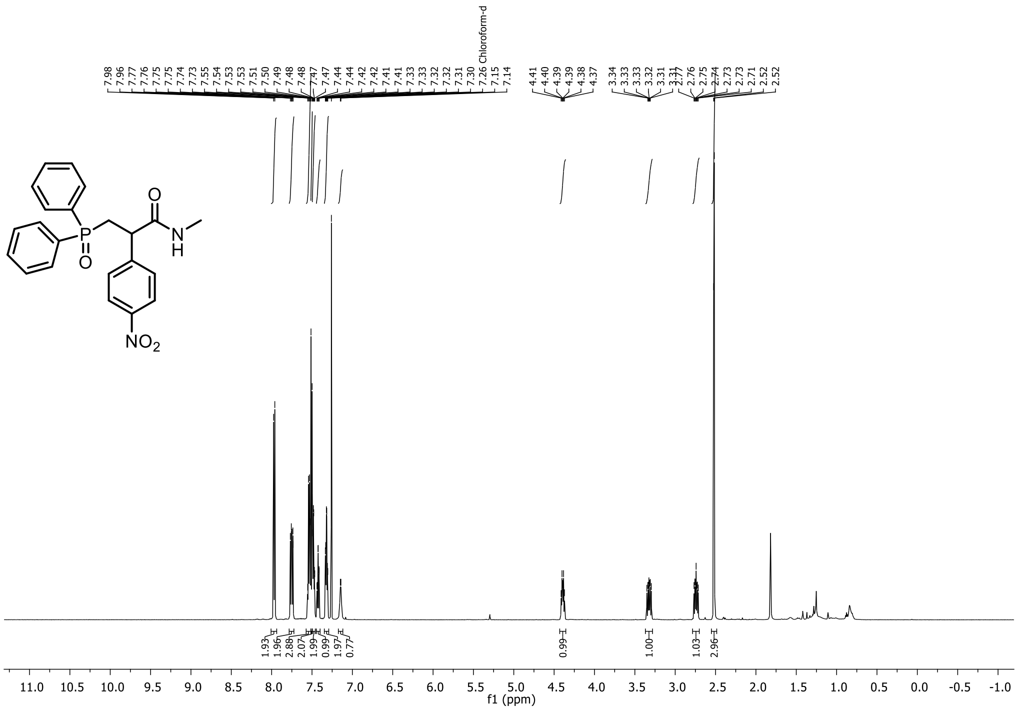

^{13}C NMR (151 MHz, $CDCl_3$):

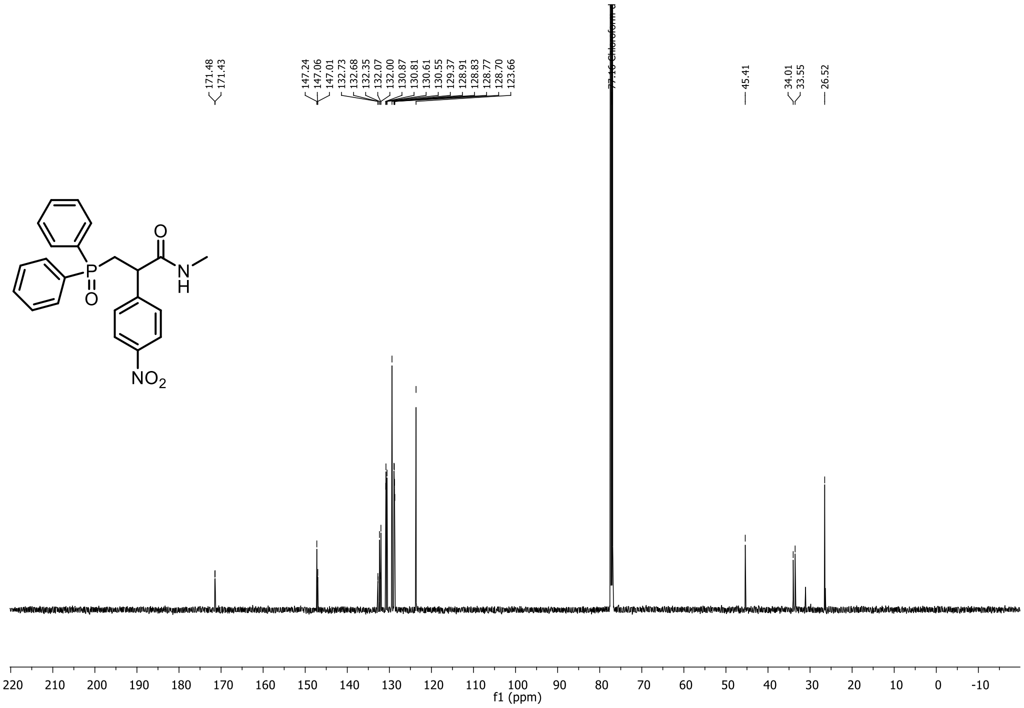

^{31}P NMR (243 MHz, $CDCl_3$):

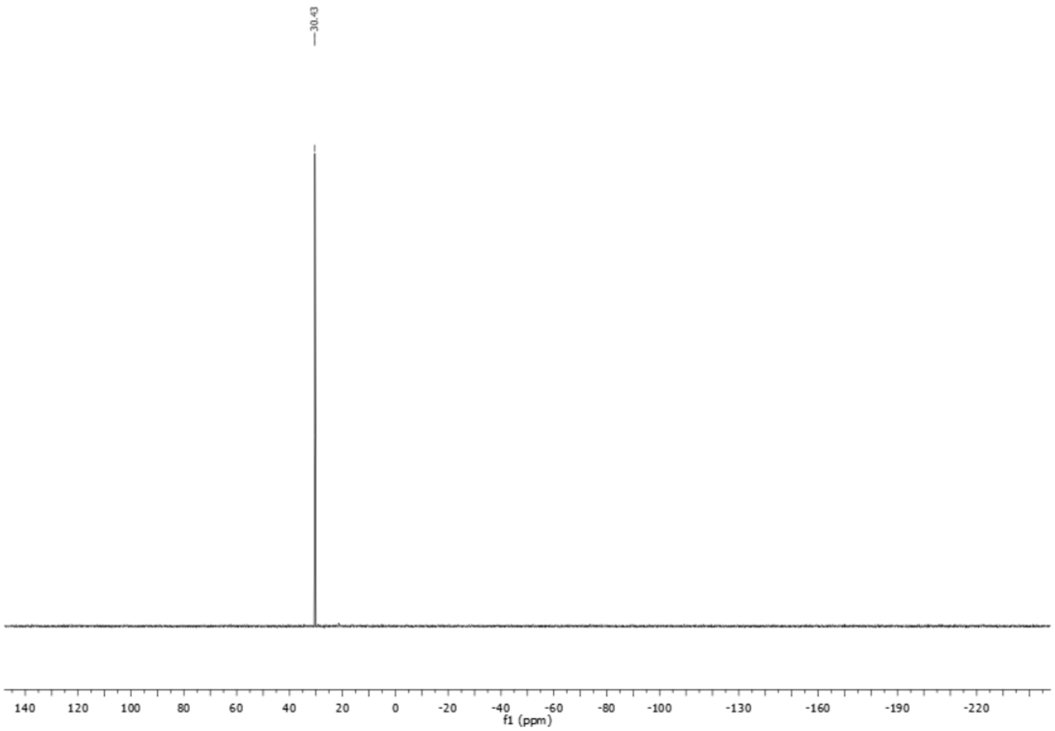

5b: *N*-methyl-2-(4-nitrophenyl)-3-(octylthio)propanamide

^{1}H NMR (700 MHz, $CDCl_3$):

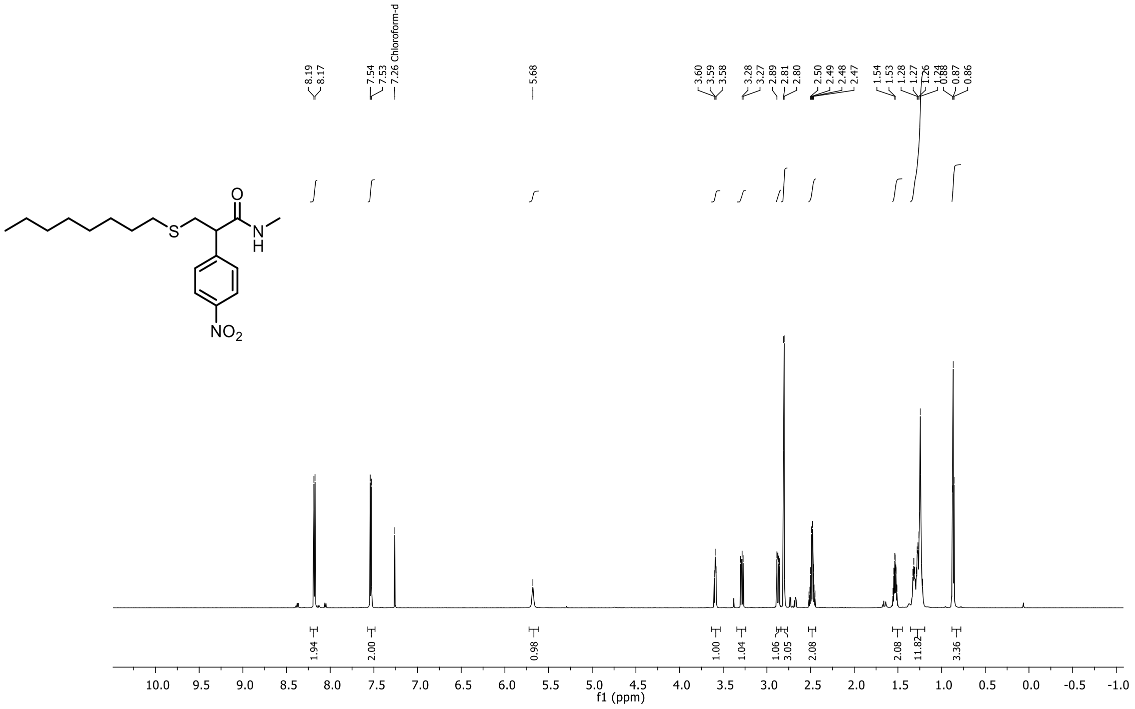

^{13}C NMR (176 MHz, $CDCl_3$):

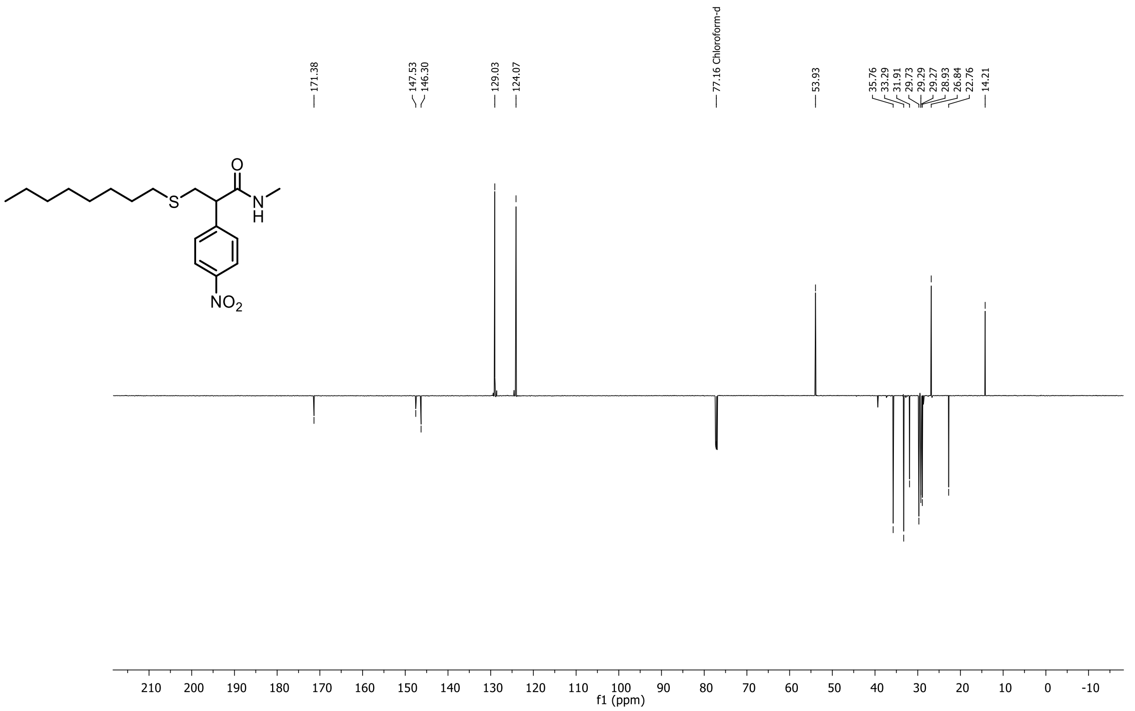

5c: 3-((4-chlorophenyl)thio)-*N*-methyl-2-(4-nitrophenyl)propanamide

^{1}H NMR (600 MHz, $CDCl_3$):

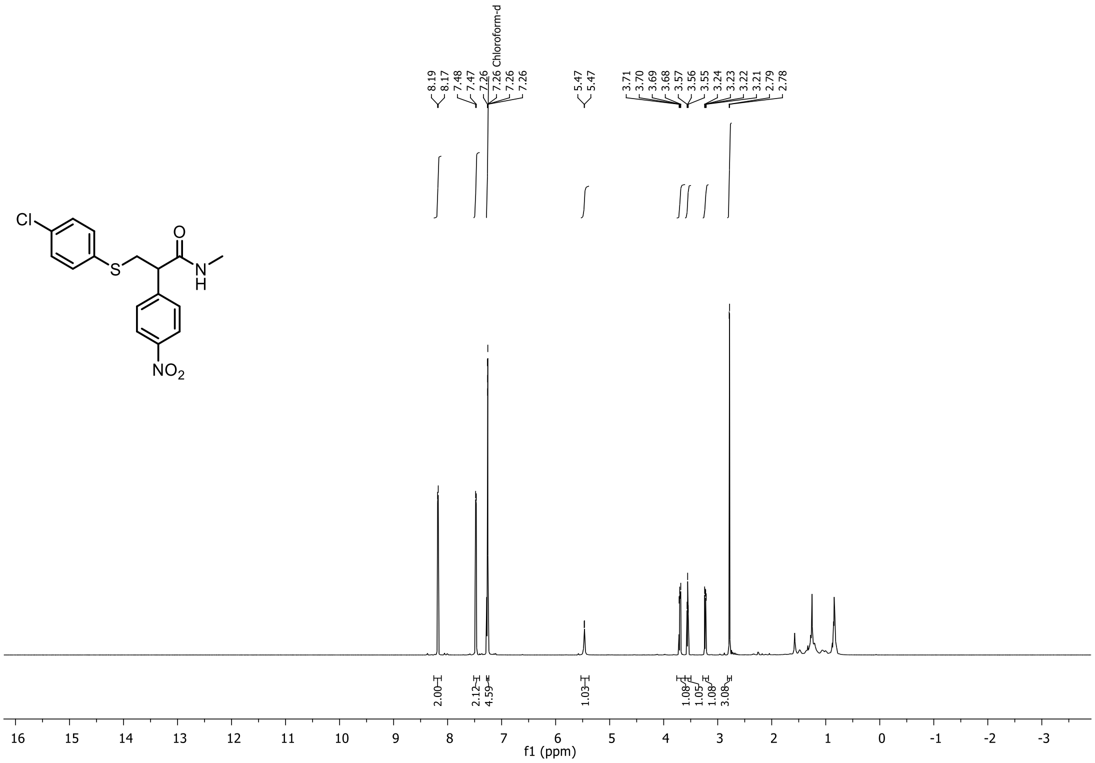

^{13}C NMR (151 MHz, $CDCl_3$):

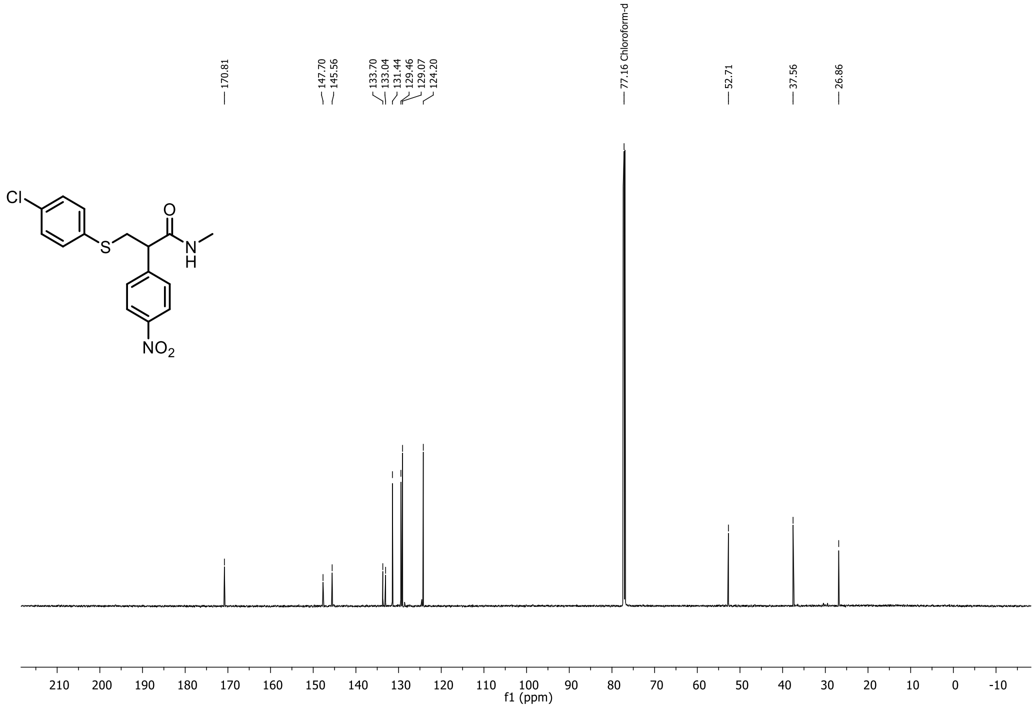

5d: *N*-methyl-2-(4-nitrophenyl)-3-(phenylsulfonyl)propanamide

^{1}H NMR (600 MHz, $CDCl_3$):

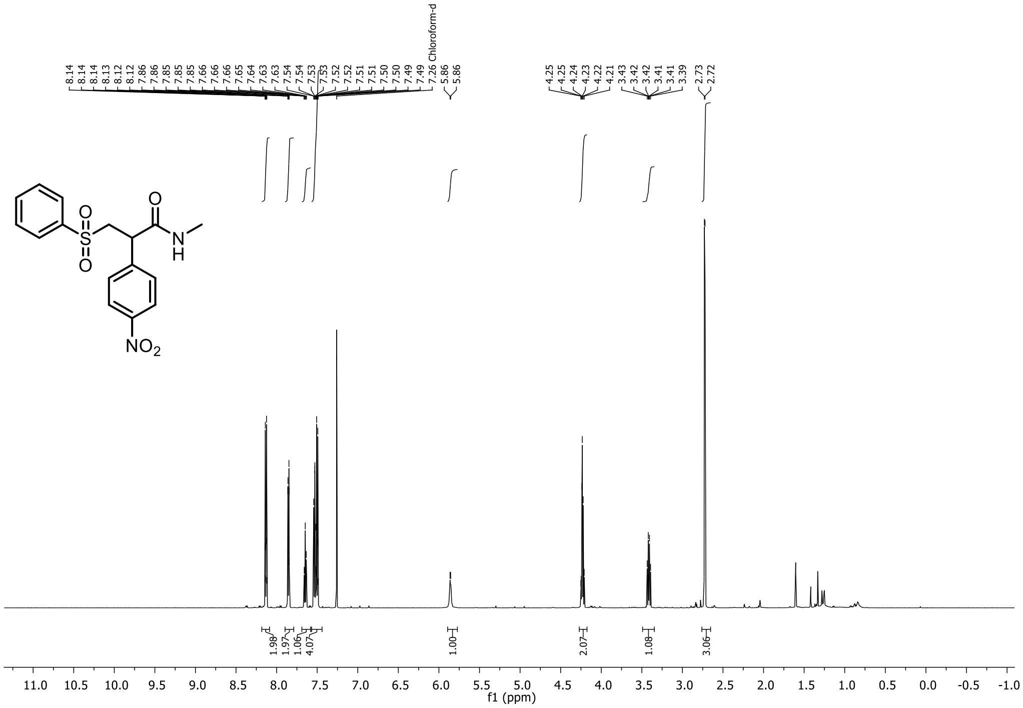

^{13}C NMR (151 MHz, $CDCl_3$):

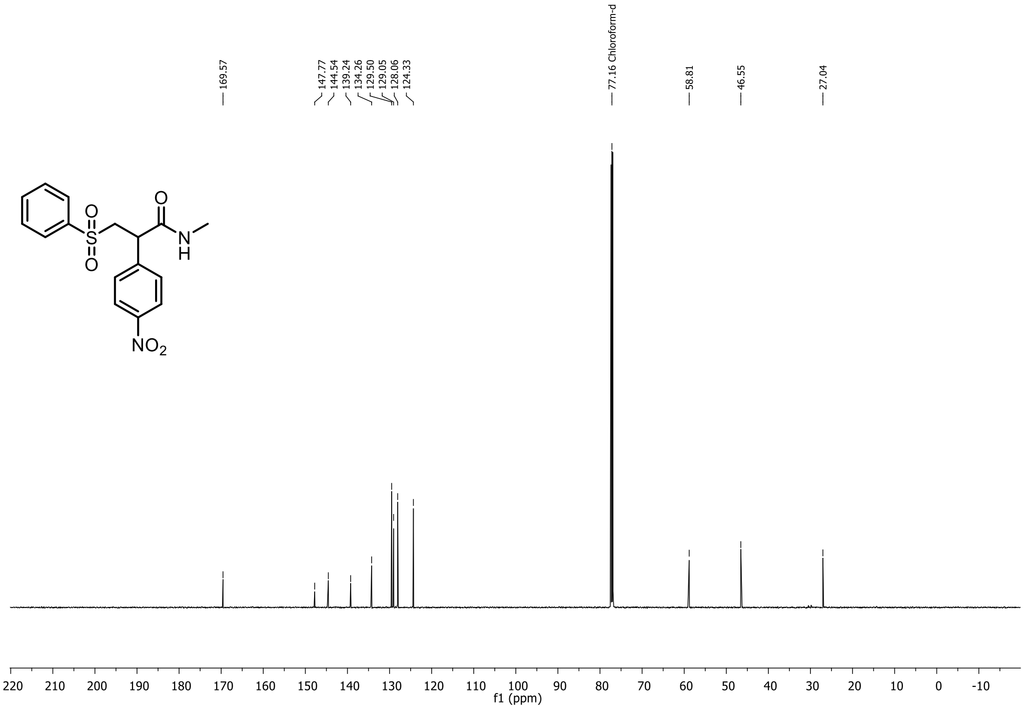

5e: *N*,2-dimethyl-2-(4-nitrophenyl)-3-(phenylsulfonyl)propanamide

^{1}H NMR (700 MHz, $CDCl_3$):

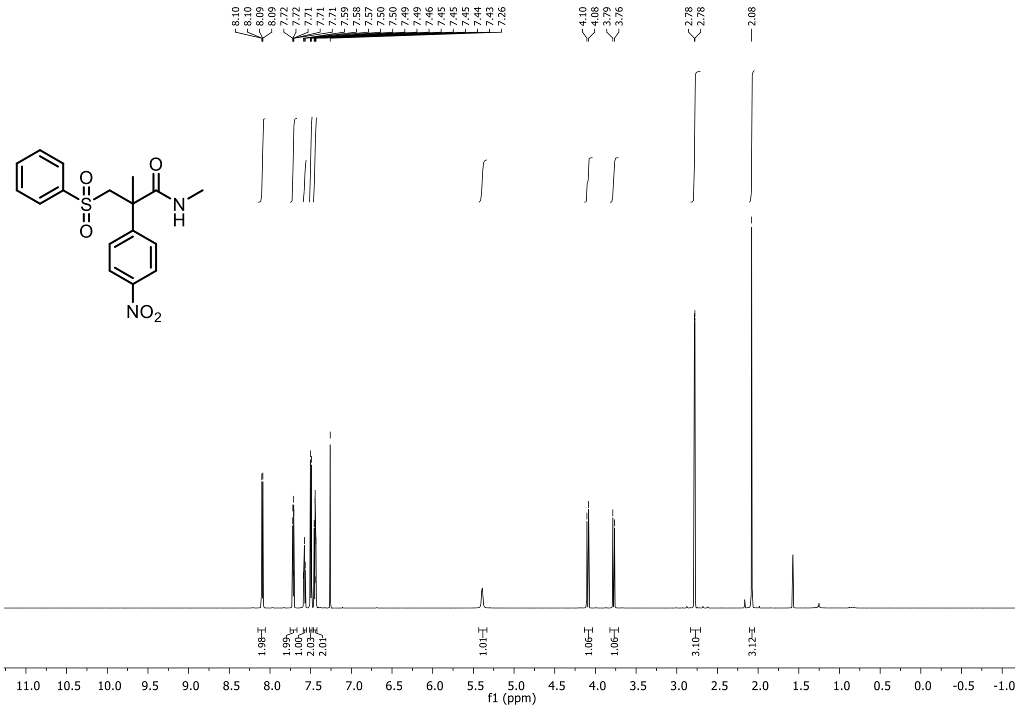

^{13}C NMR (176 MHz, $CDCl_3$):

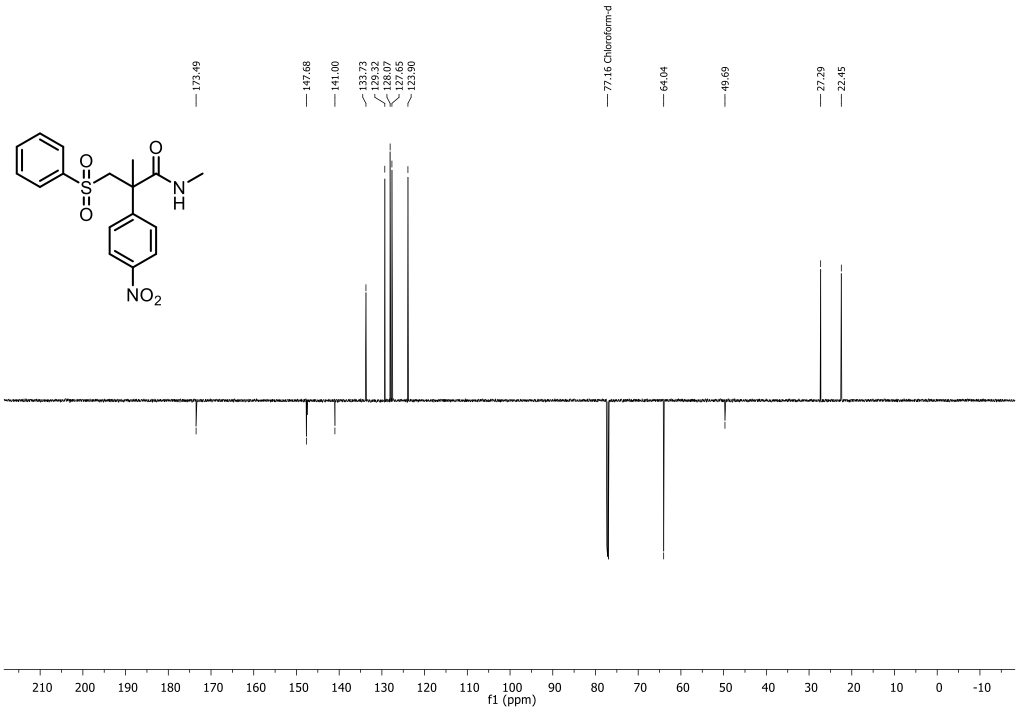

5f: *N*-methyl-3-((*N*-methyl-4-nitrophenyl)sulfonamido)-2-(4-nitrophenyl)propanamide

¹H NMR (600 MHz, $CDCl_3$):

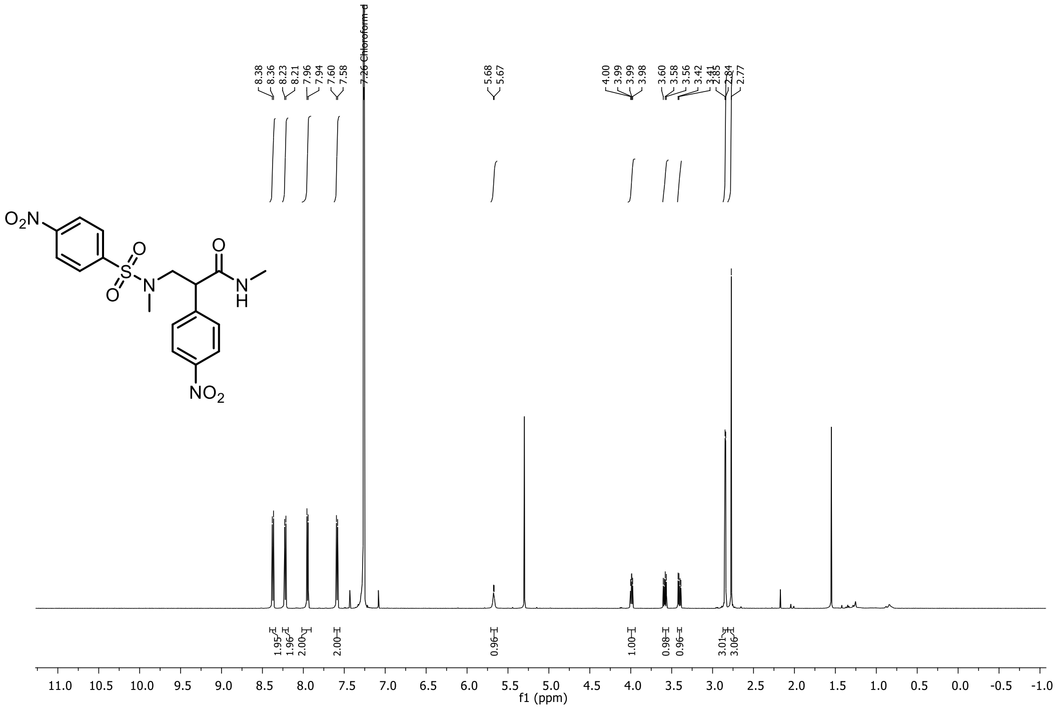

¹³C NMR (151 MHz, $CDCl_3$):

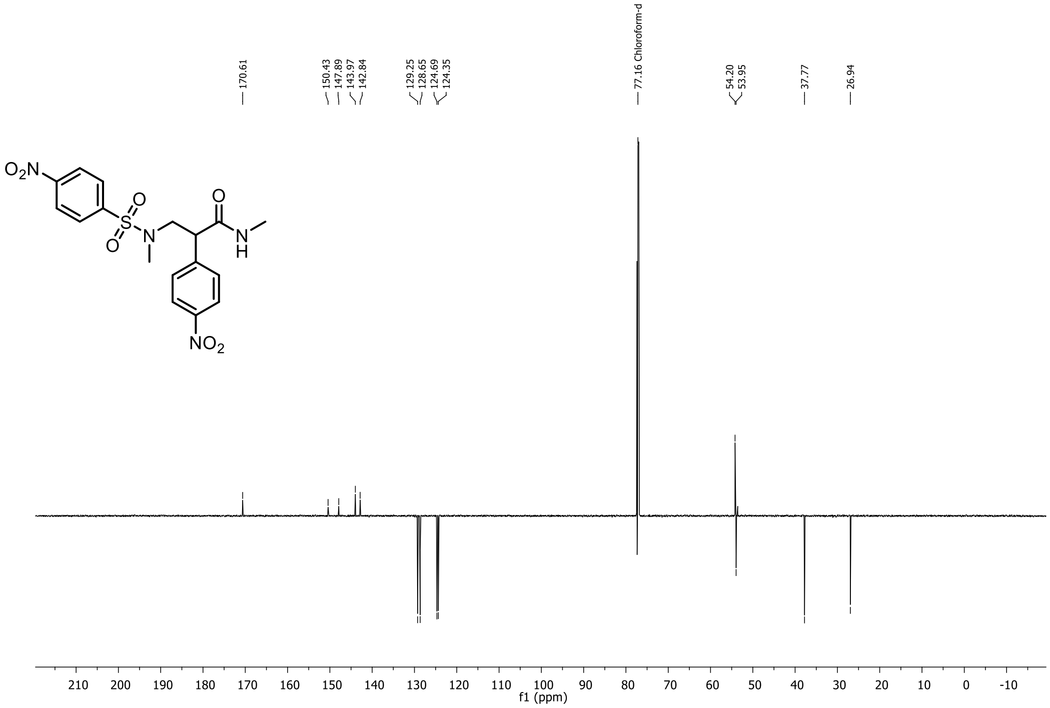

5g: 3-(diallylamino)-*N*-methyl-2-(4-nitrophenyl)propanamide

¹H NMR (600 MHz, $CDCl_3$):

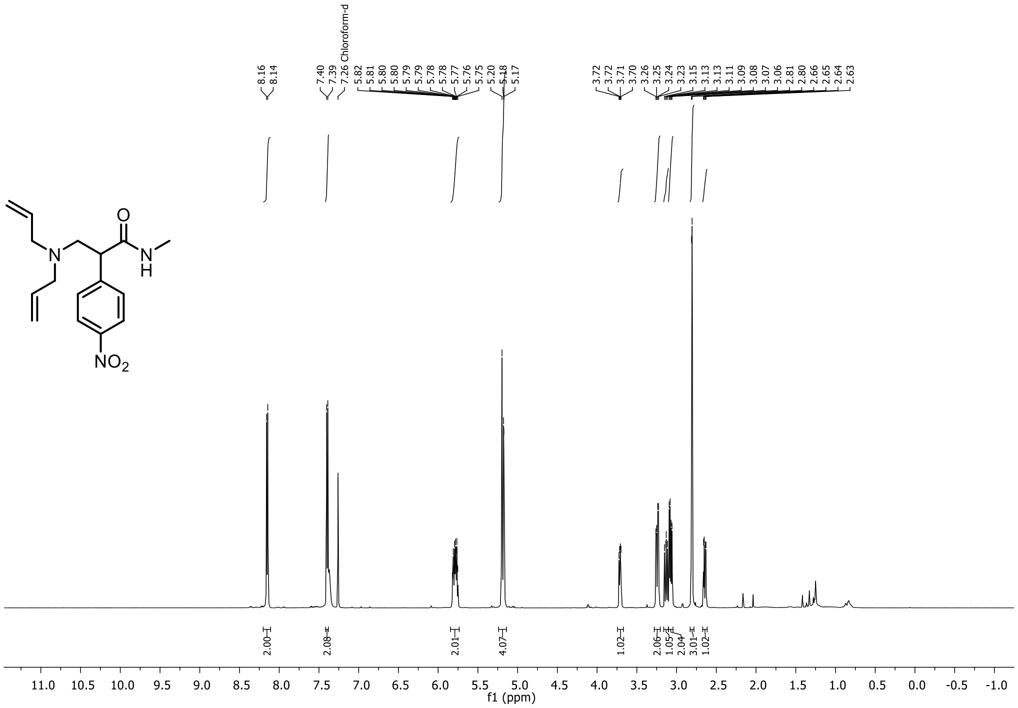

¹³C NMR (151 MHz, $CDCl_3$):

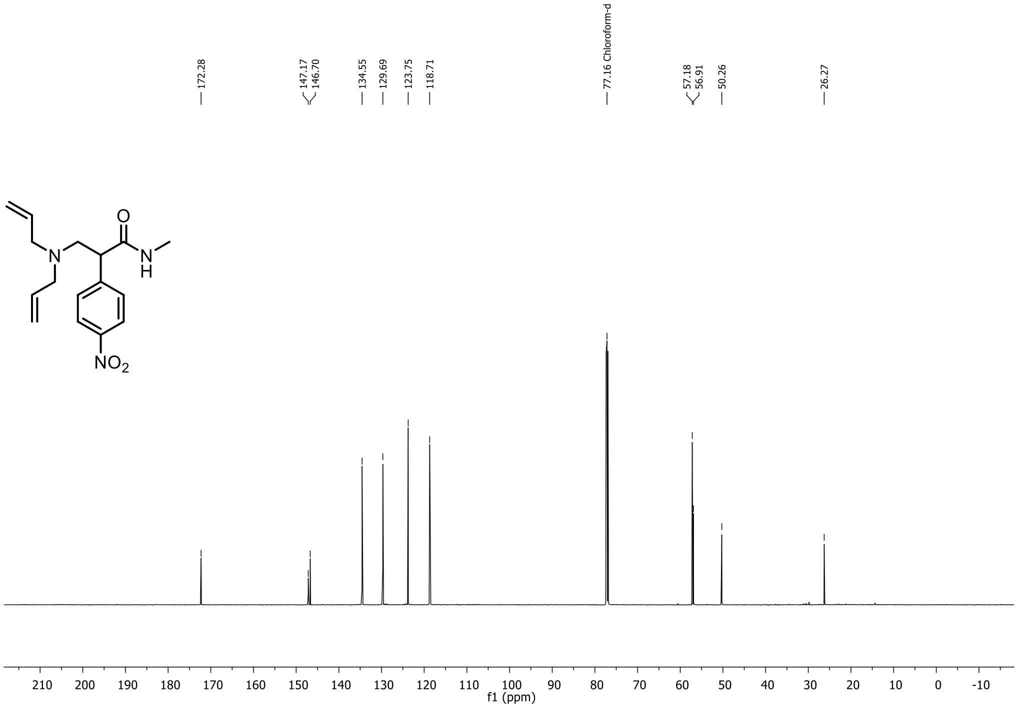

5h: 3-(benzyl(methyl)amino)-*N*-methyl-2-(4-nitrophenyl)propanamide

^{1}H NMR (600 MHz, $CDCl_3$):

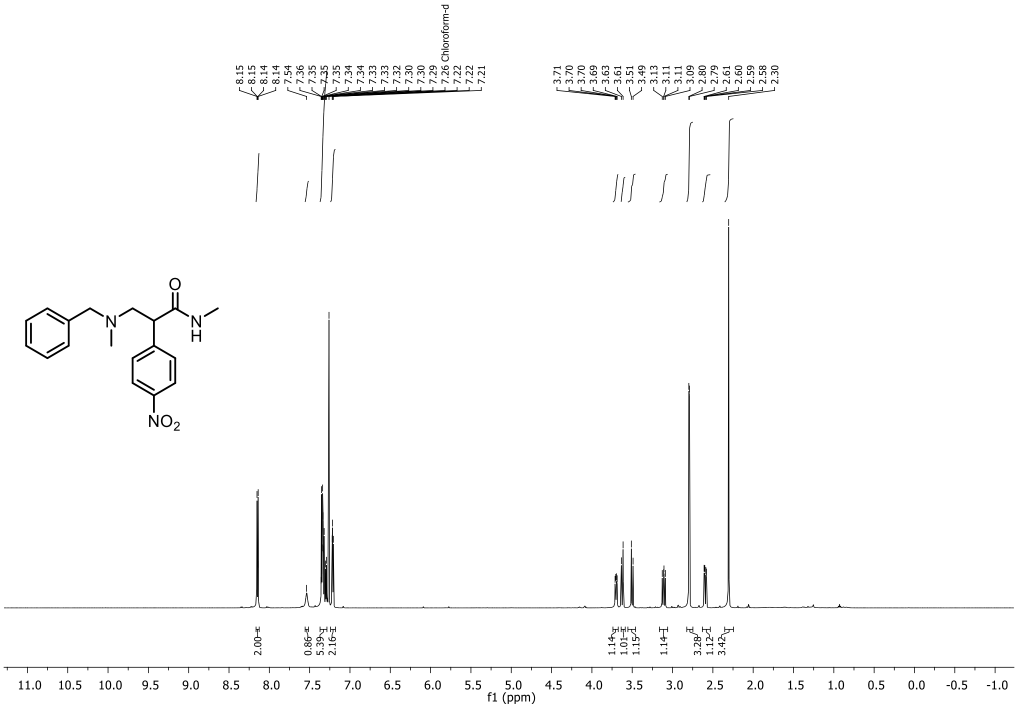

^{13}C NMR (151 MHz, $CDCl_3$):

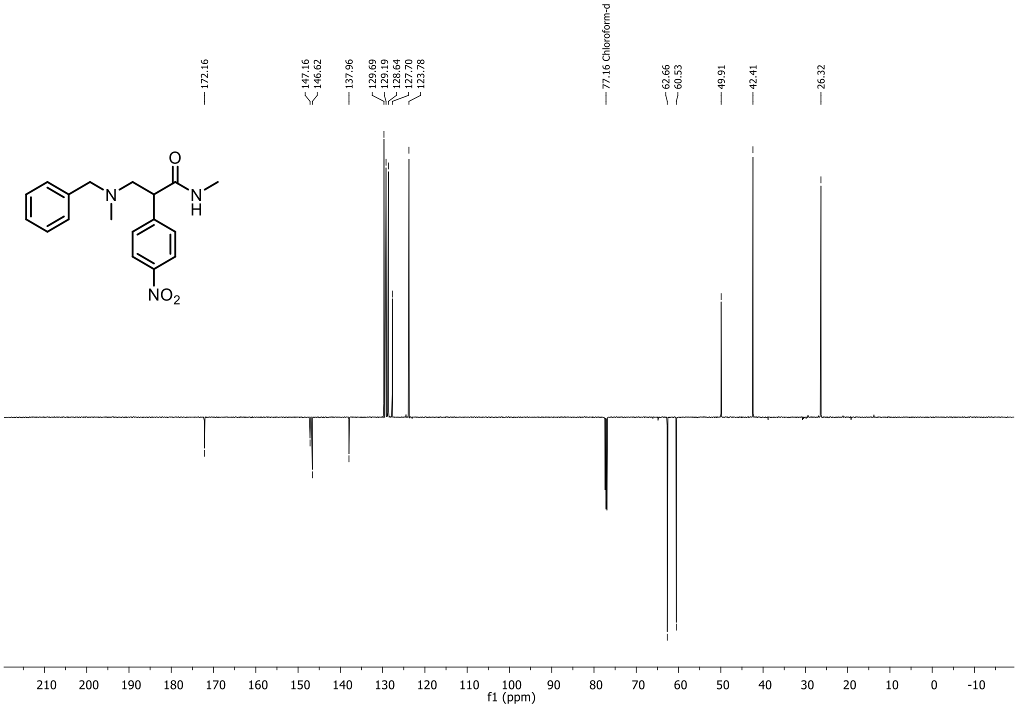

5i: *N*-methyl-3-(methyl(phenyl)amino)-2-(4-nitrophenyl)propanamide

^{1}H NMR (600 MHz, $CDCl_3$):

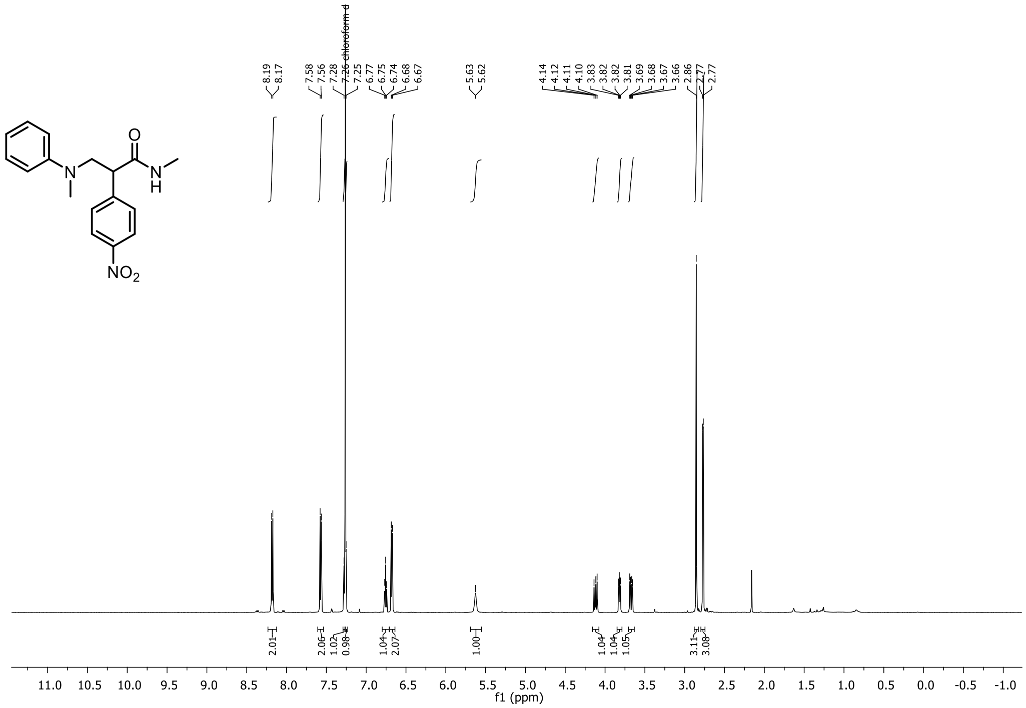

^{13}C NMR (151 MHz, $CDCl_3$):

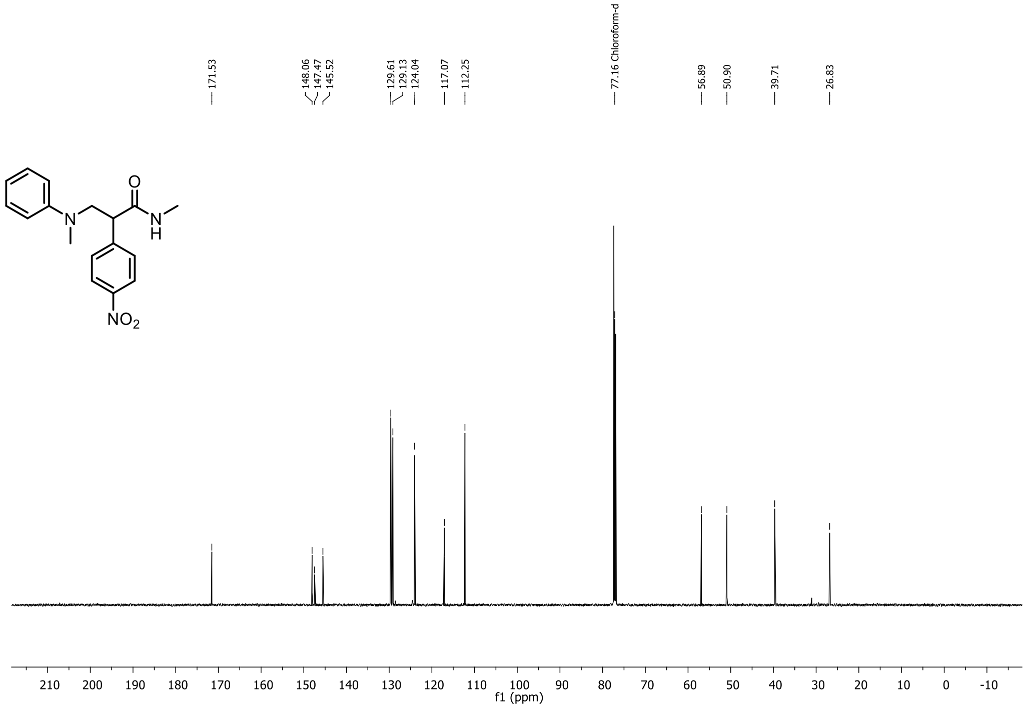

5j-A: 3-((3aR,6S,7aS)-8,8-dimethyl-2,2-dioxidotetrahydro-3H-3a,6-methanobenzo[c]isothiazol-1(4H)-yl)-*N*-methyl-2-(4-nitrophenyl)propanamide

^{1}H NMR (600 MHz, $CDCl_3$):

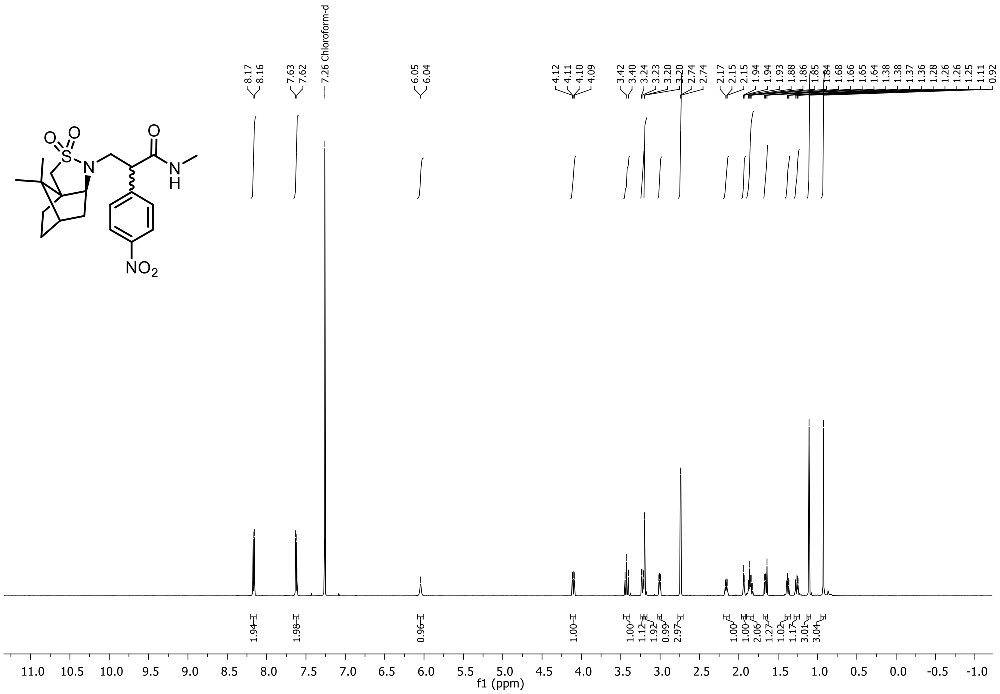

^{13}C NMR (151 MHz, $CDCl_3$):

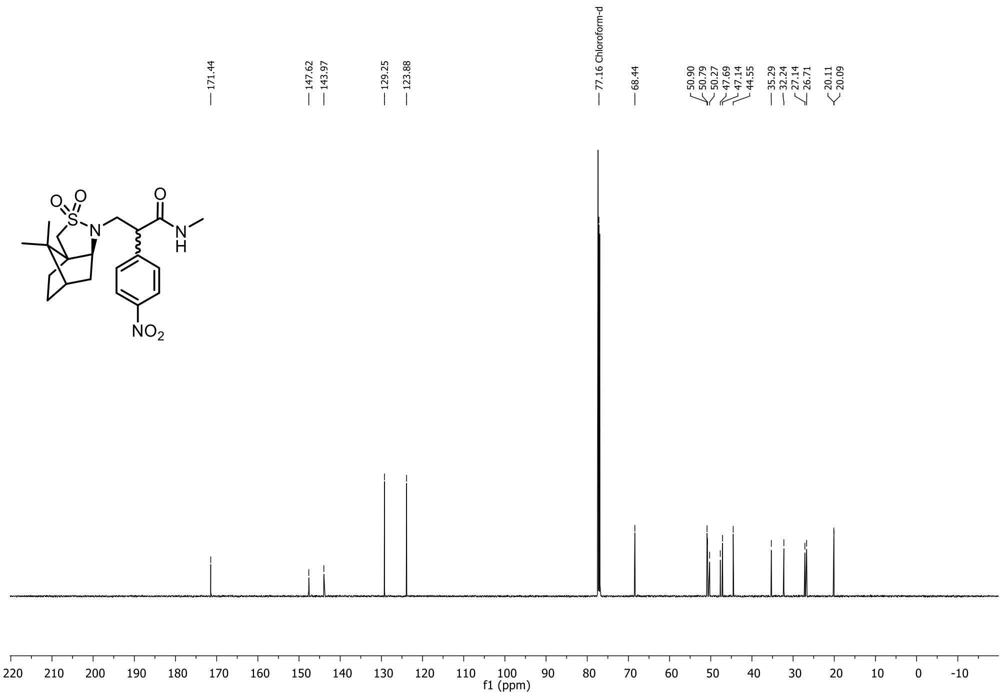

5j-B: 3-((3aR,6S,7aS)-8,8-dimethyl-2,2-dioxidotetrahydro-3H-3a,6-methanobenzo[c]isothiazol-1(4H)-yl)-*N*-methyl-2-(4-nitrophenyl)propanamide

^{1}H NMR (600 MHz, $CDCl_3$):

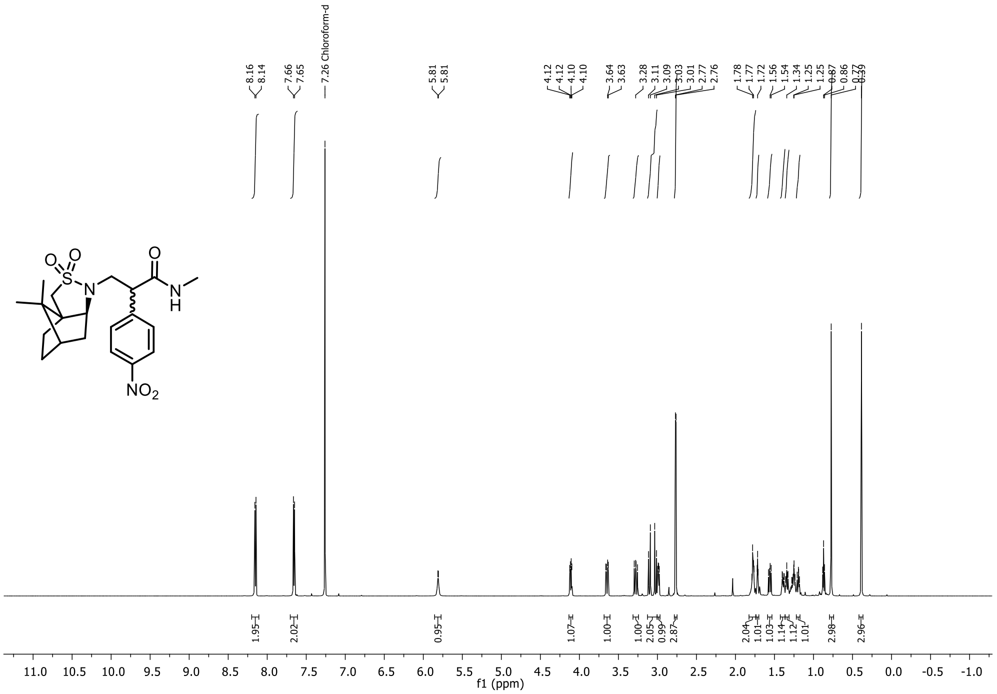

^{13}C NMR (151 MHz, $CDCl_3$):

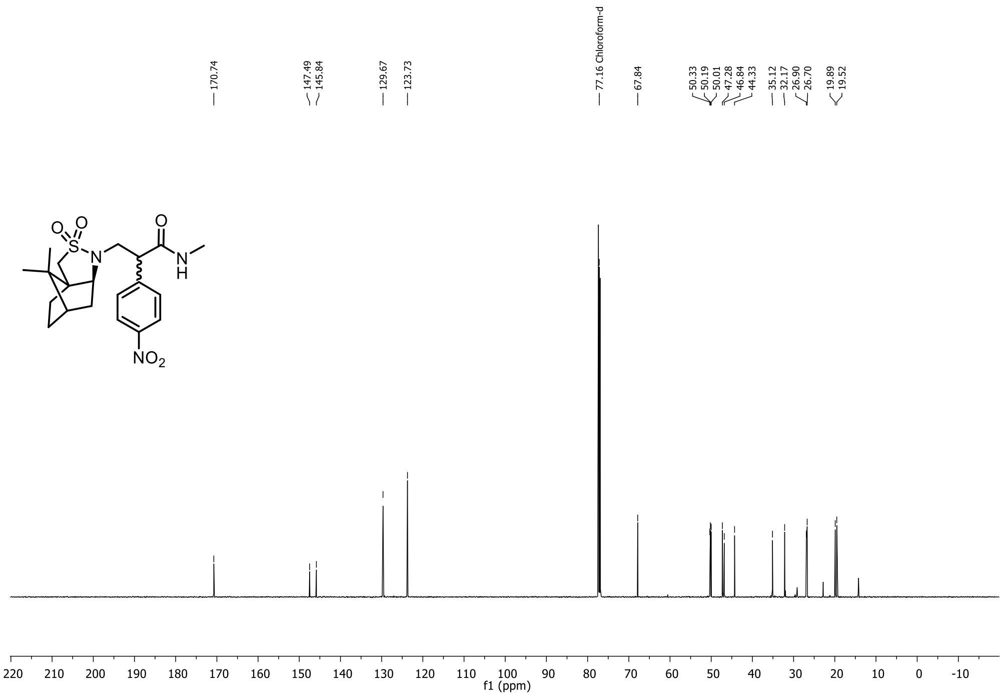

5k: *N*-methyl-3-morpholino-2-(4-nitrophenyl)propenamide

^{1}H NMR (700 MHz, $CDCl_3$):

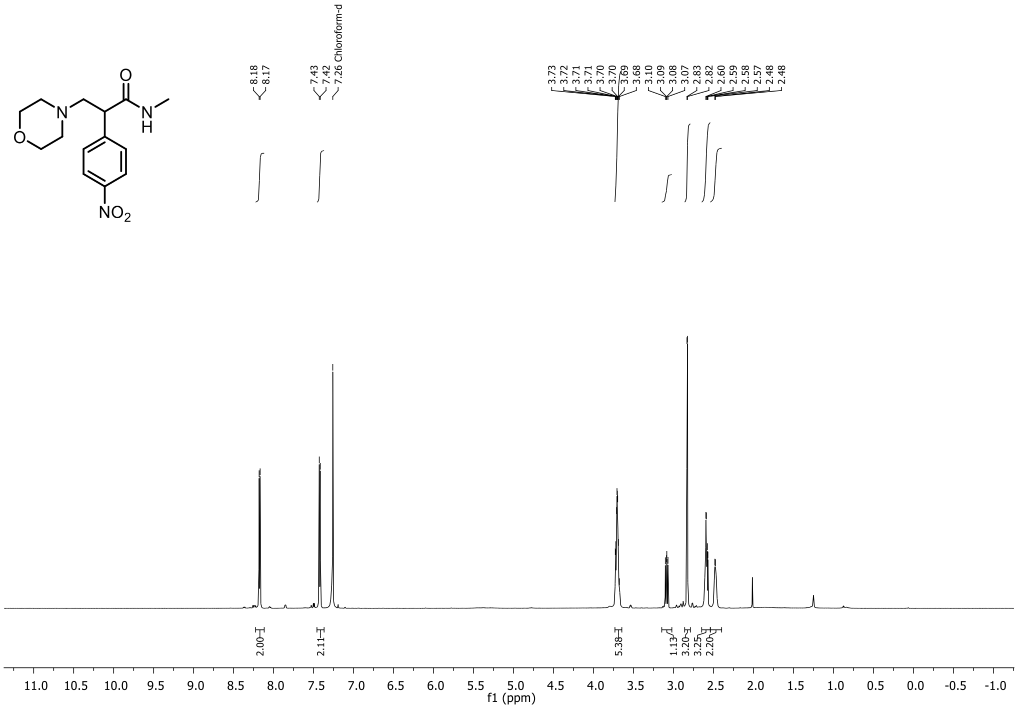

^{13}C NMR (176 MHz, $CDCl_3$):

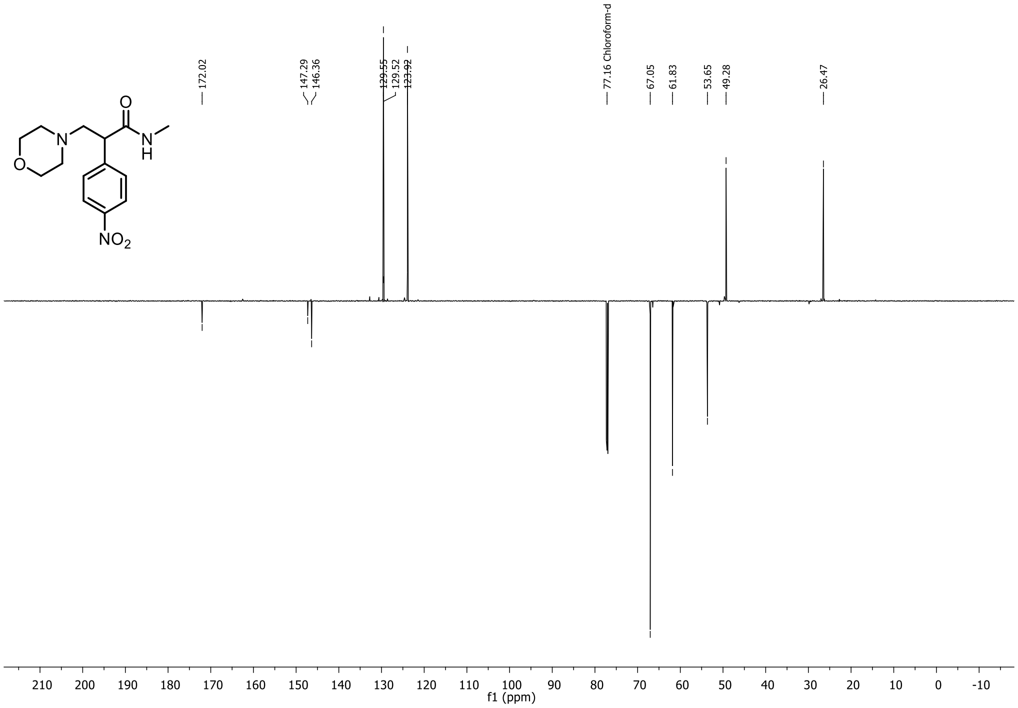

5l: 3-(1H-benzo[d][1,2,3]triazol-1-yl)-*N*-methyl-2-(4-nitrophenyl)propanamide

^{1}H NMR (400 MHz, $CDCl_3$):

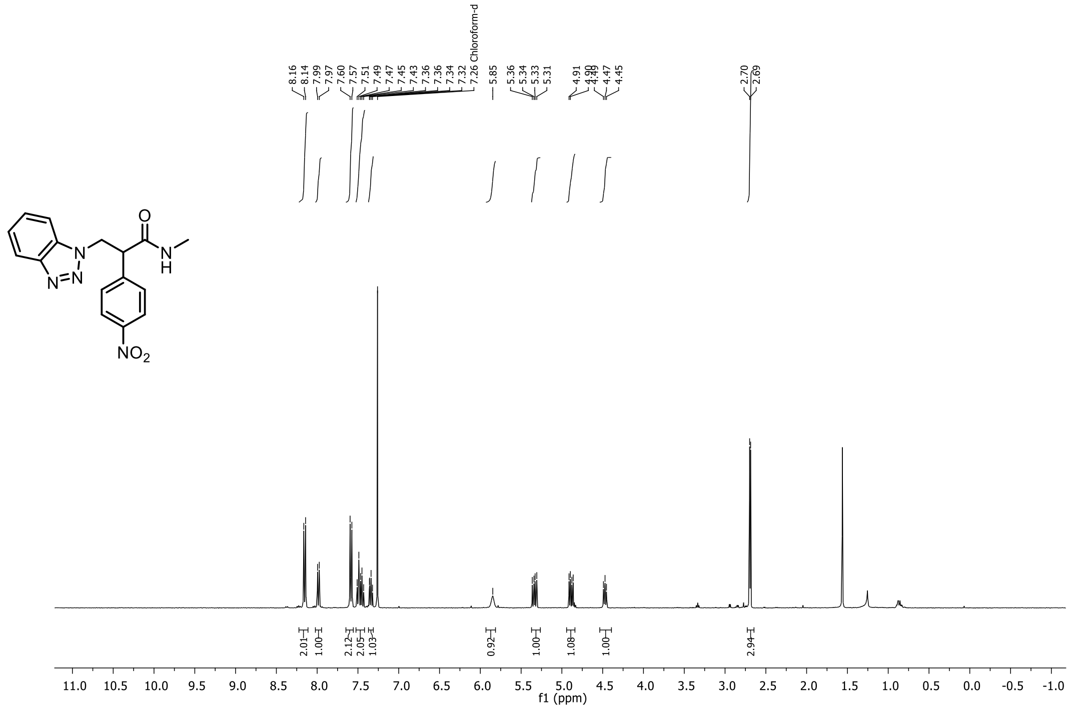

^{13}C NMR (176 MHz, $CDCl_3$):

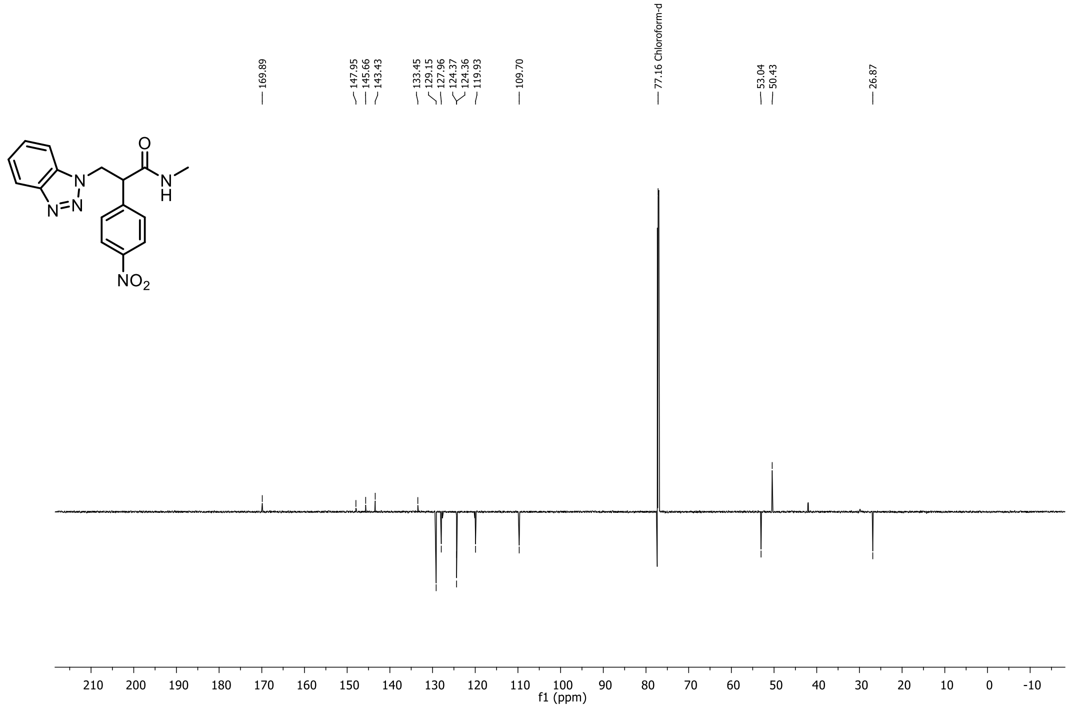

5m: 3-(benzylamino)-*N*-methyl-2-(4-nitrophenyl)propenamide

^{1}H NMR (700 MHz, $CDCl_3$):

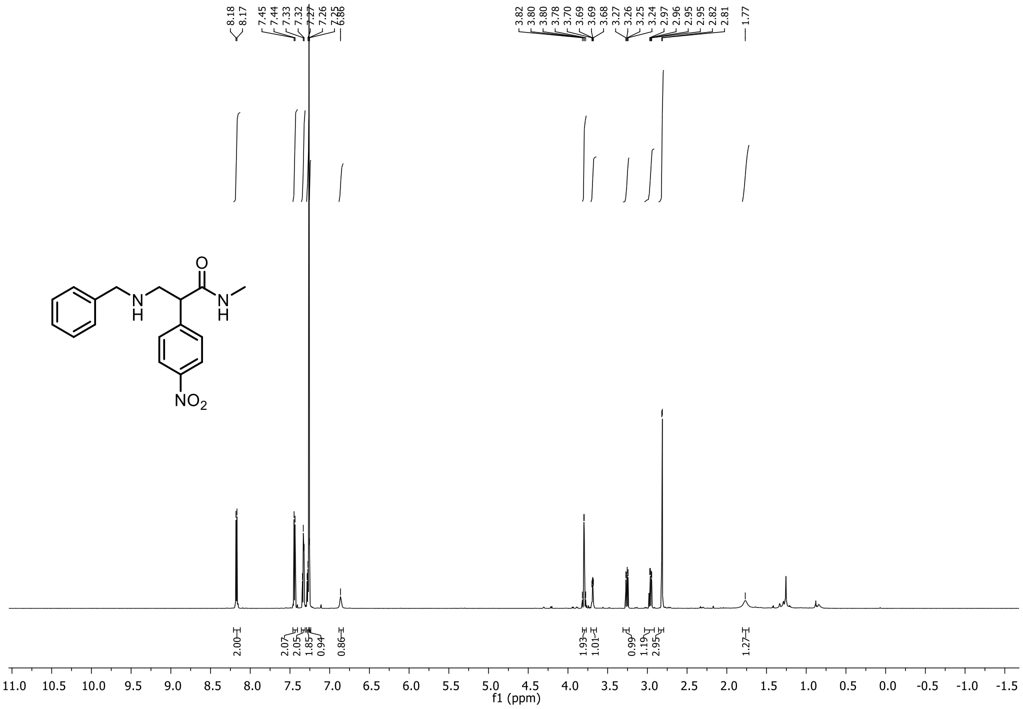

^{13}C NMR (176 MHz, $CDCl_3$):

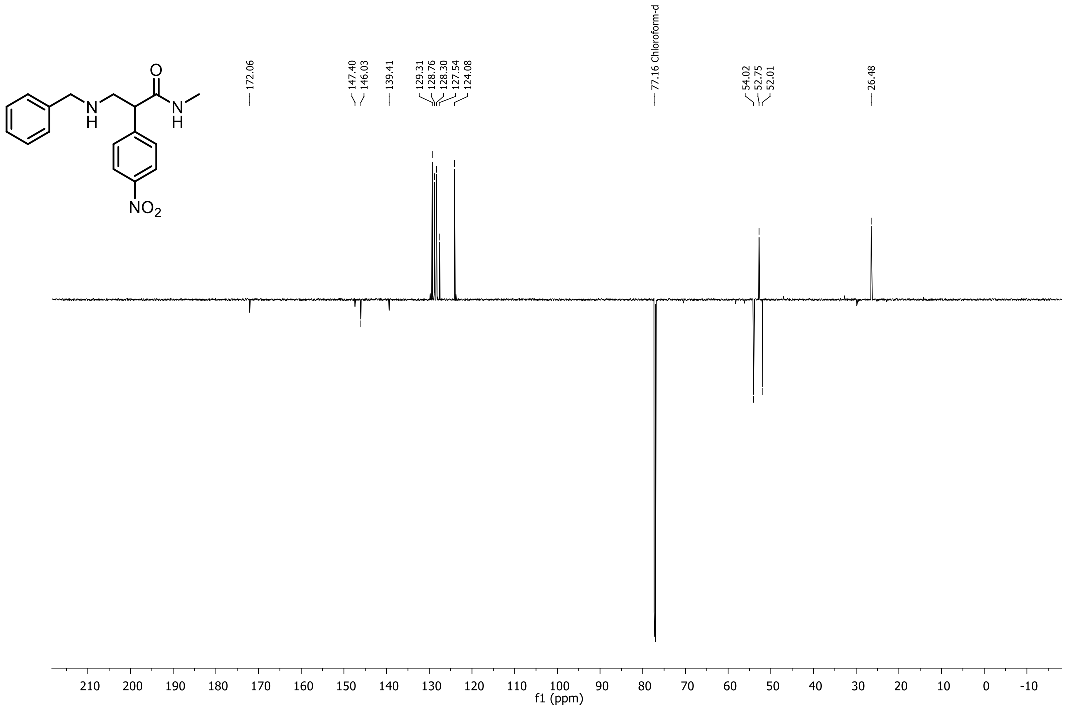

5n: 3-((2,3-dihydro-1H-inden-2-yl)amino)-*N*-methyl-2-(4-nitrophenyl)propenamide

^{1}H NMR (600 MHz, $CDCl_3$):

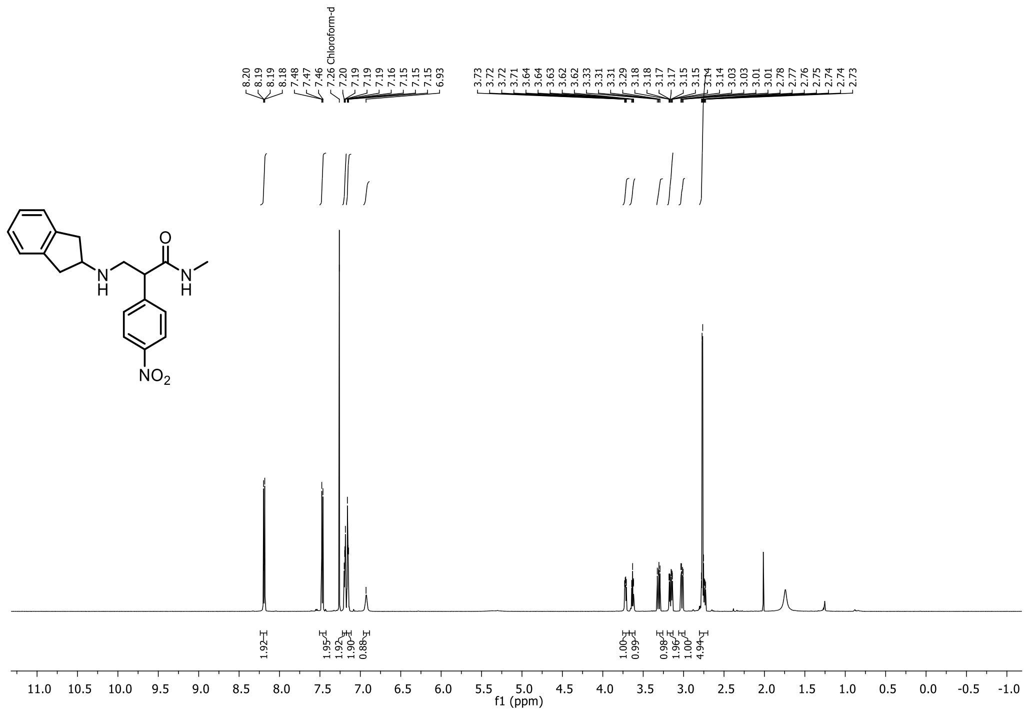

^{13}C NMR (151 MHz, $CDCl_3$):

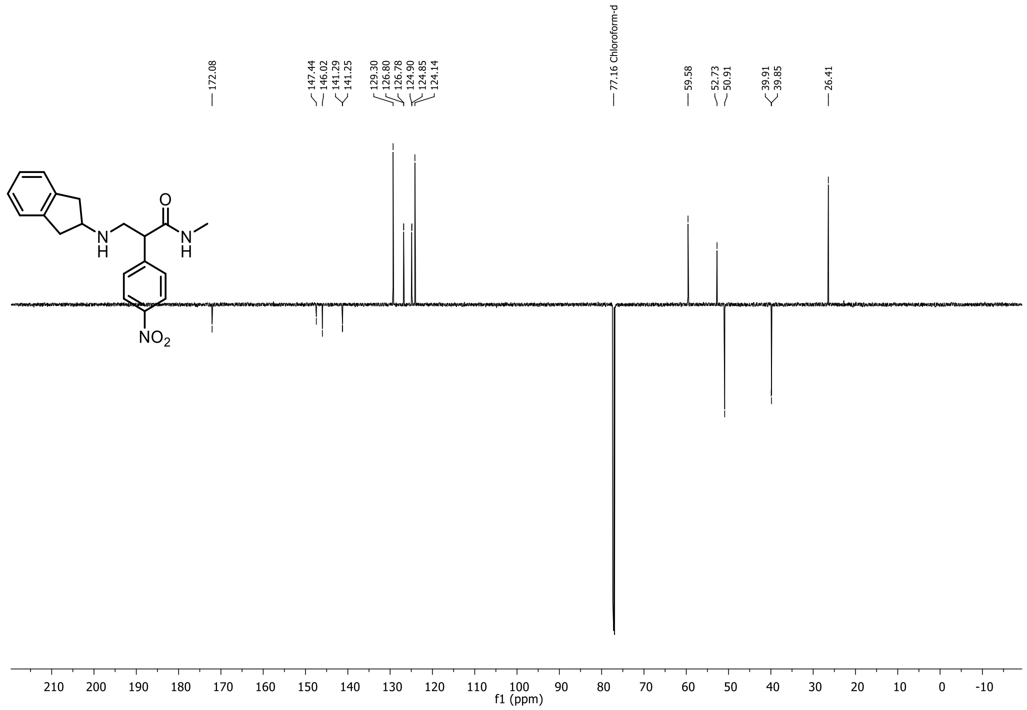

5o: *N*,2-dimethyl-3-((*N*-methyl-4-nitrophenyl)sulfonamido)-2-(4-nitrophenyl)propanamide

^{1}H NMR (400 MHz, $CDCl_3$):

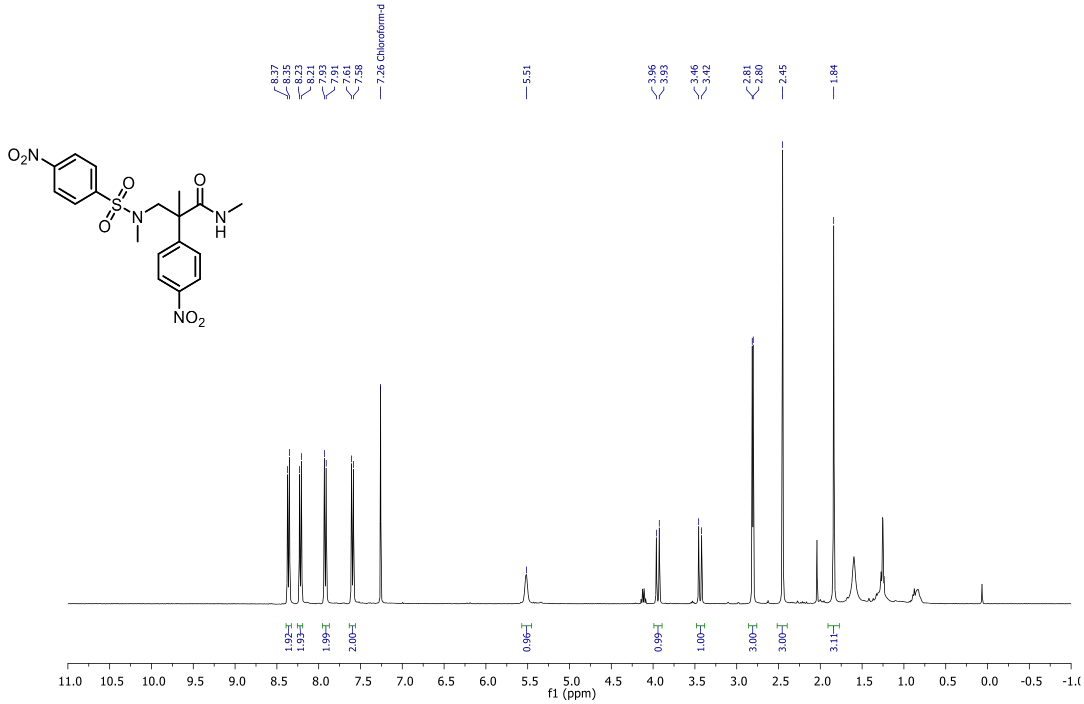

^{13}C NMR (101 MHz, $CDCl_3$):

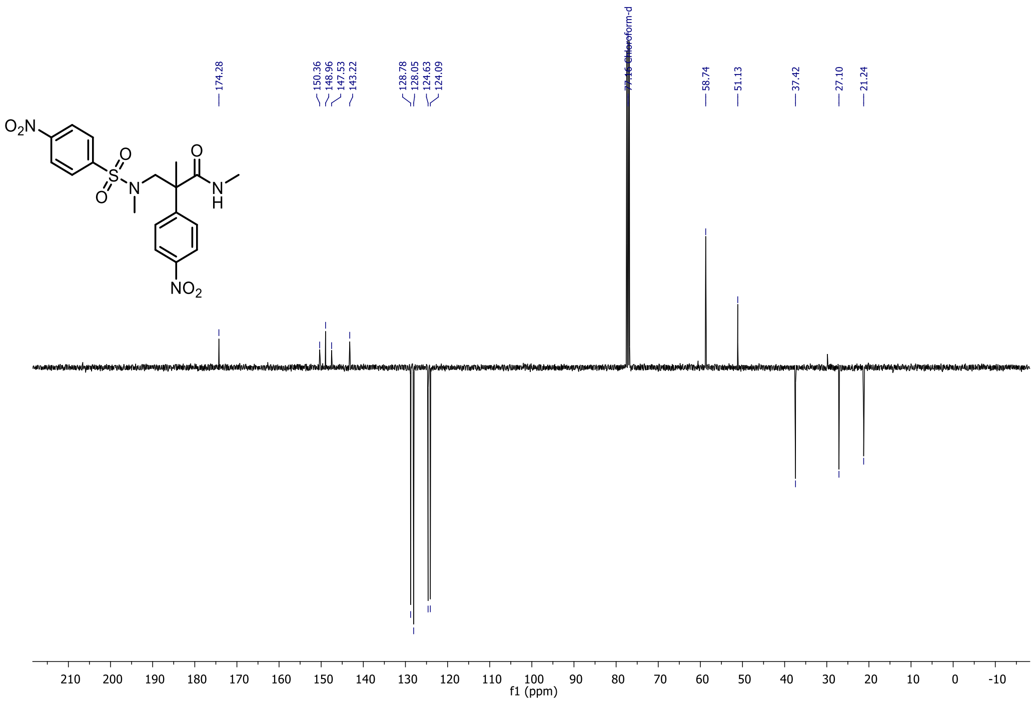

9b: *N*-methyl-3-((*N*-methyl-4-nitrophenyl)sulfonamido)-2-(4-nitrophenyl)-2-phenylpropanamide

^{1}H NMR (600 MHz, $CDCl_3$):

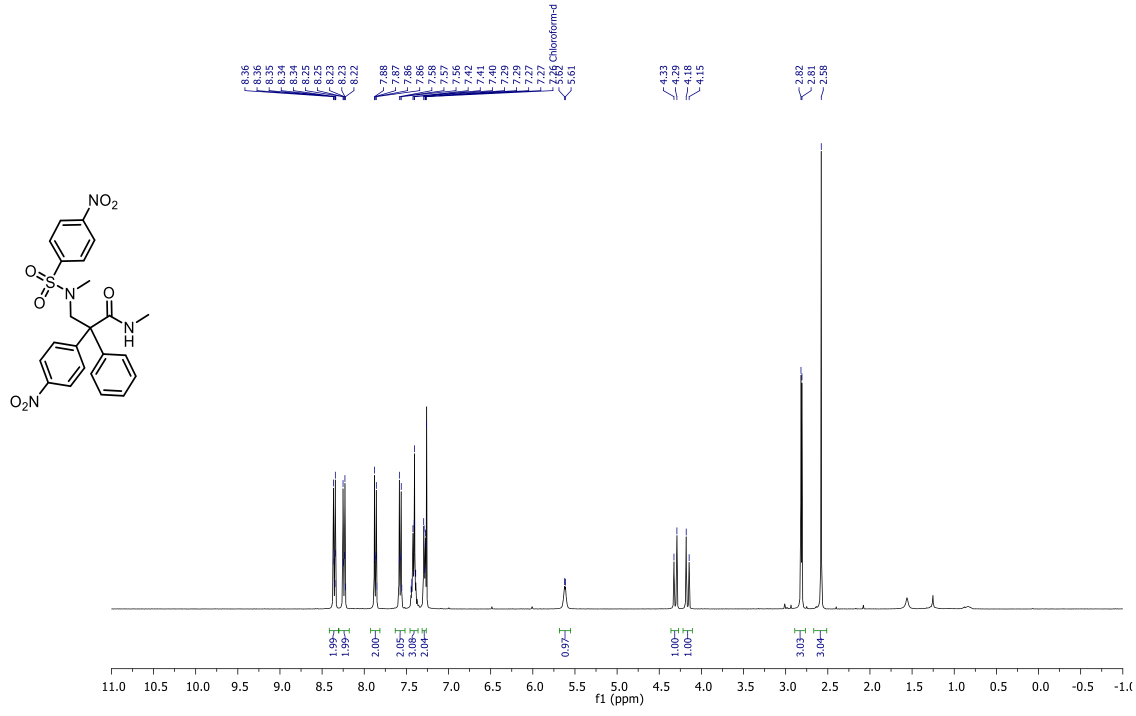

^{13}C NMR (151 MHz, $CDCl_3$):

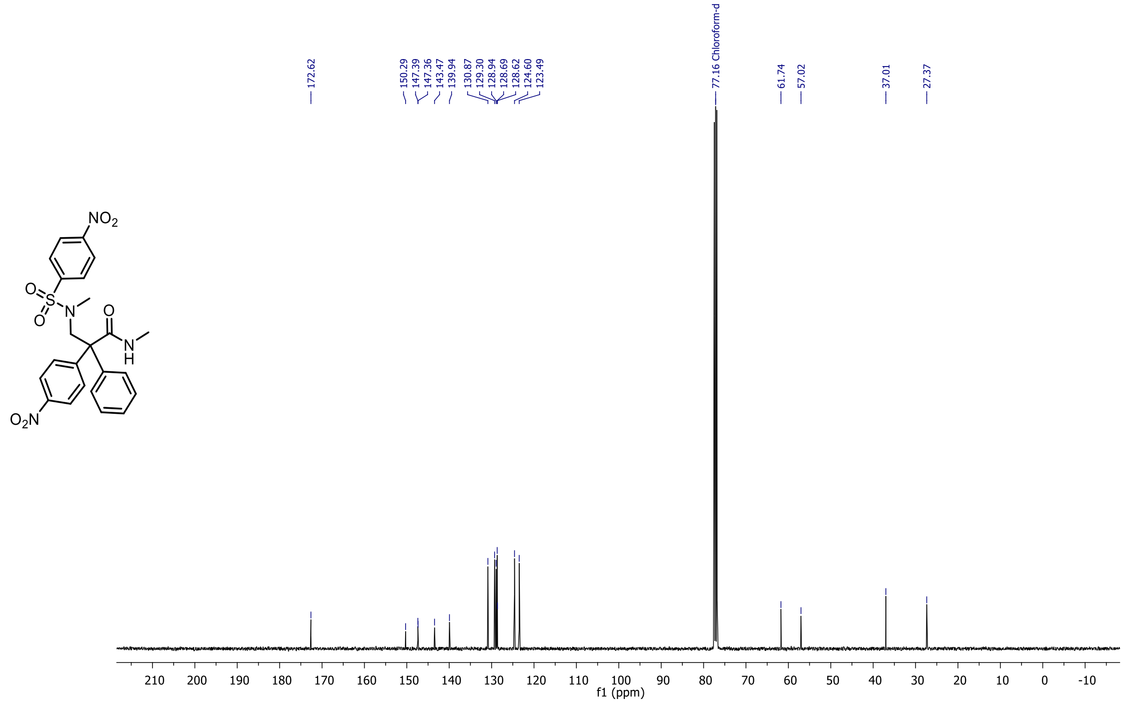

10b: *N*-methyl-N-((4-nitrophenyl)sulfonyl)-2-phenylacrylamide

^{1}H NMR (600 MHz, $CDCl_3$):

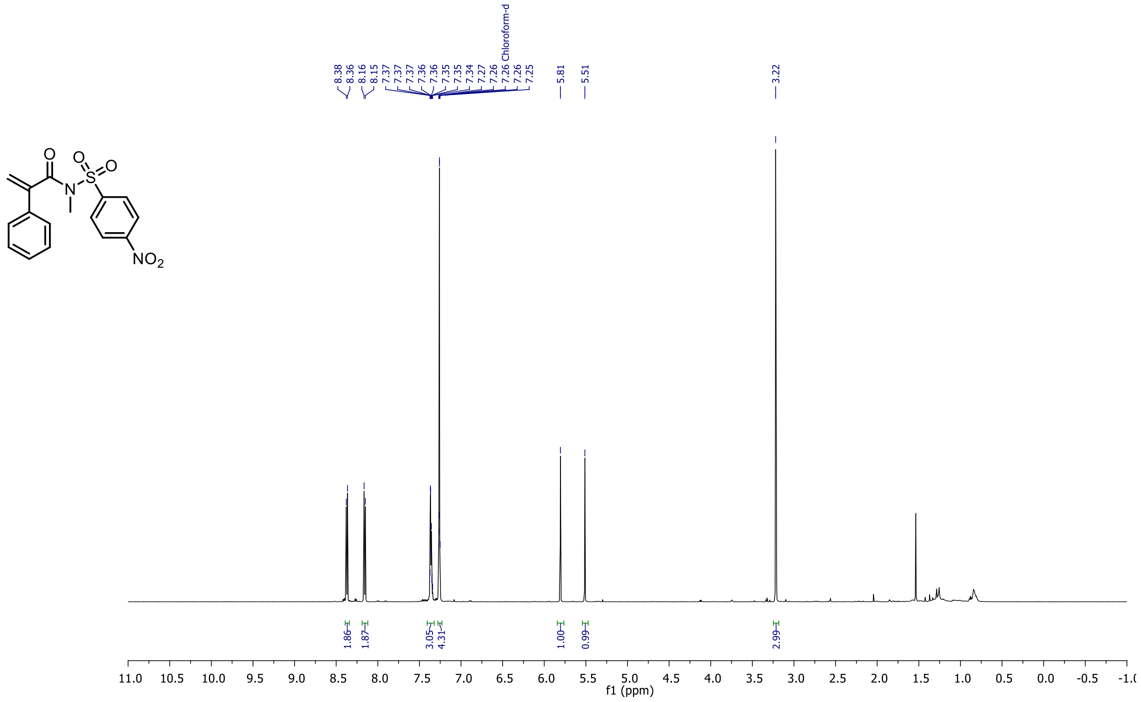

^{13}C NMR (151 MHz, $CDCl_3$):

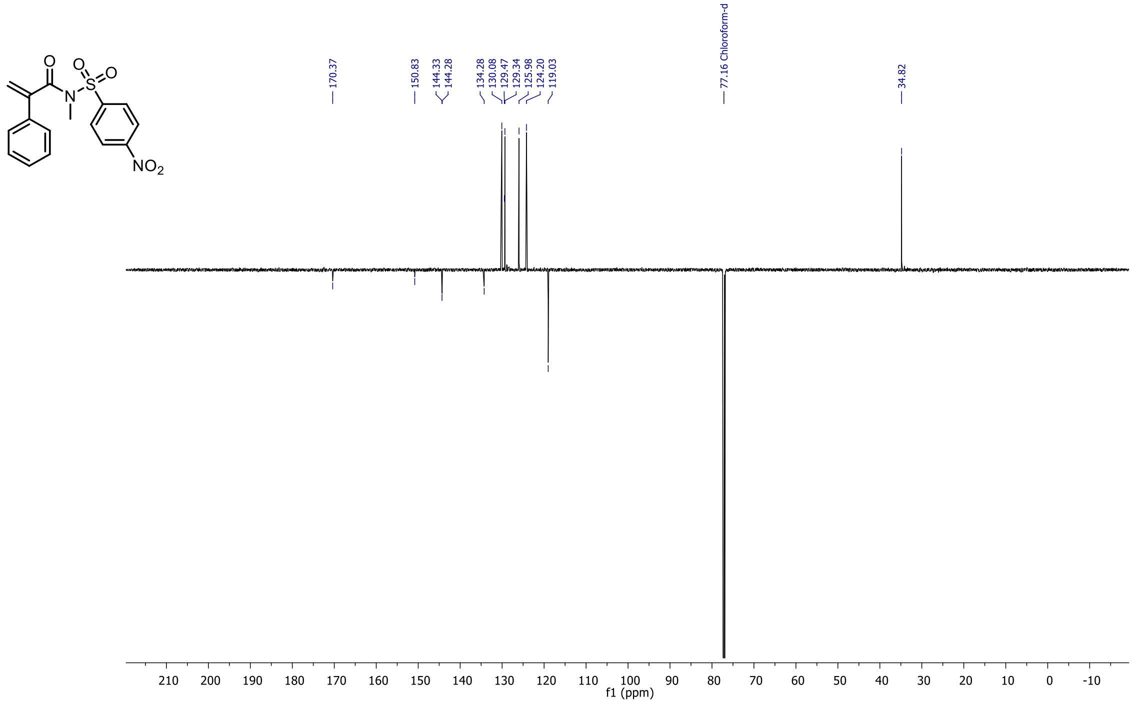

9a: 2-benzyl-*N*-methyl-3-((*N*-methyl-4-nitrophenyl)sulfonamido)-2-(4-nitrophenyl)propanamide

¹H NMR (700 MHz, $CDCl_3$):

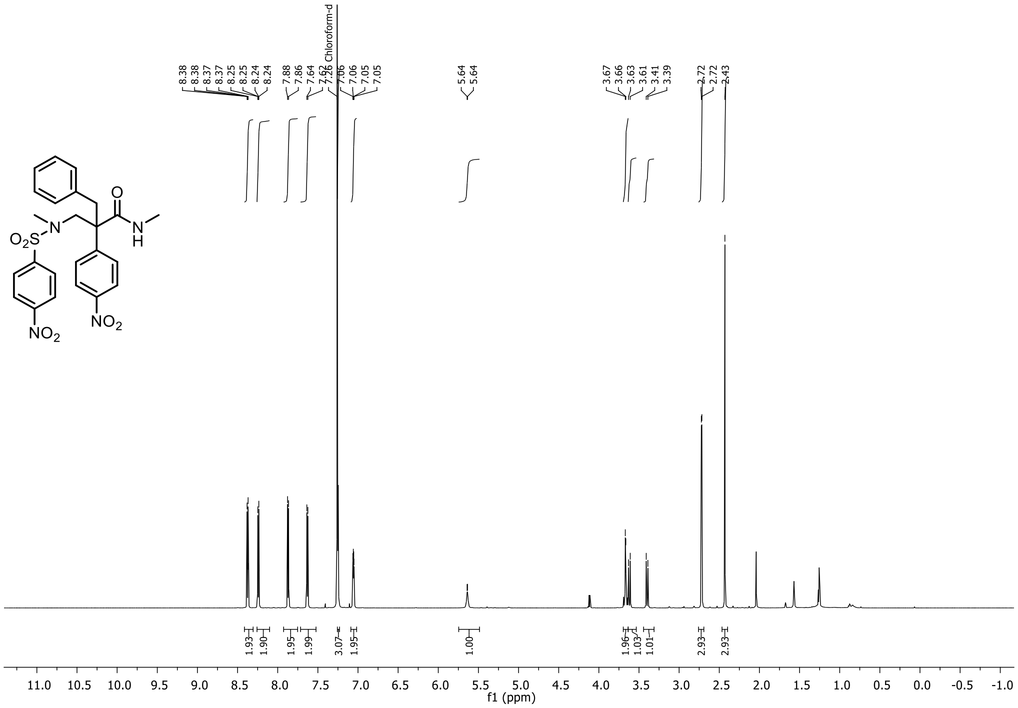

¹³C NMR (176 MHz, $CDCl_3$):

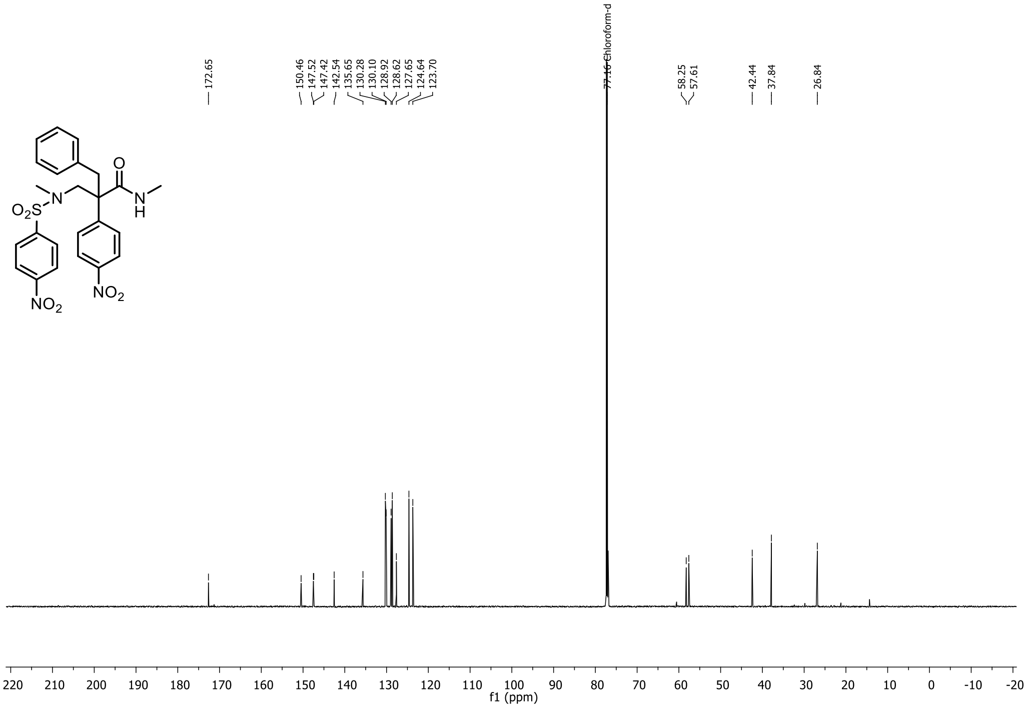

10a: 2-benzyl-*N*-methyl-*N*-((4-nitrophenyl)sulfonyl)acrylamide

^{1}H NMR (700 MHz, $CDCl_3$):

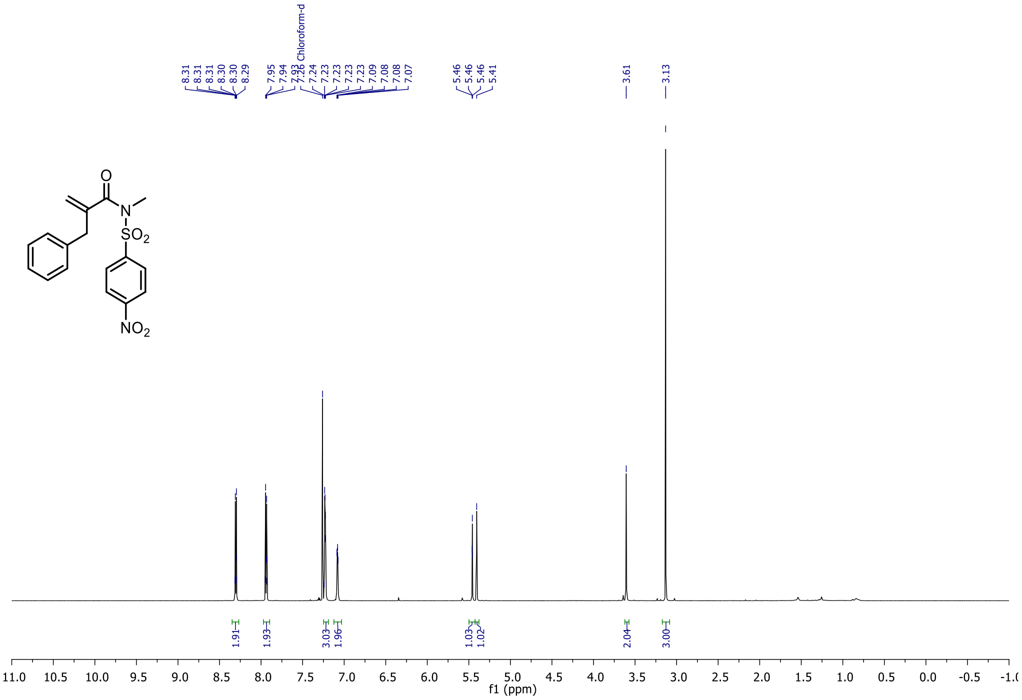

^{13}C NMR (176 MHz, $CDCl_3$):

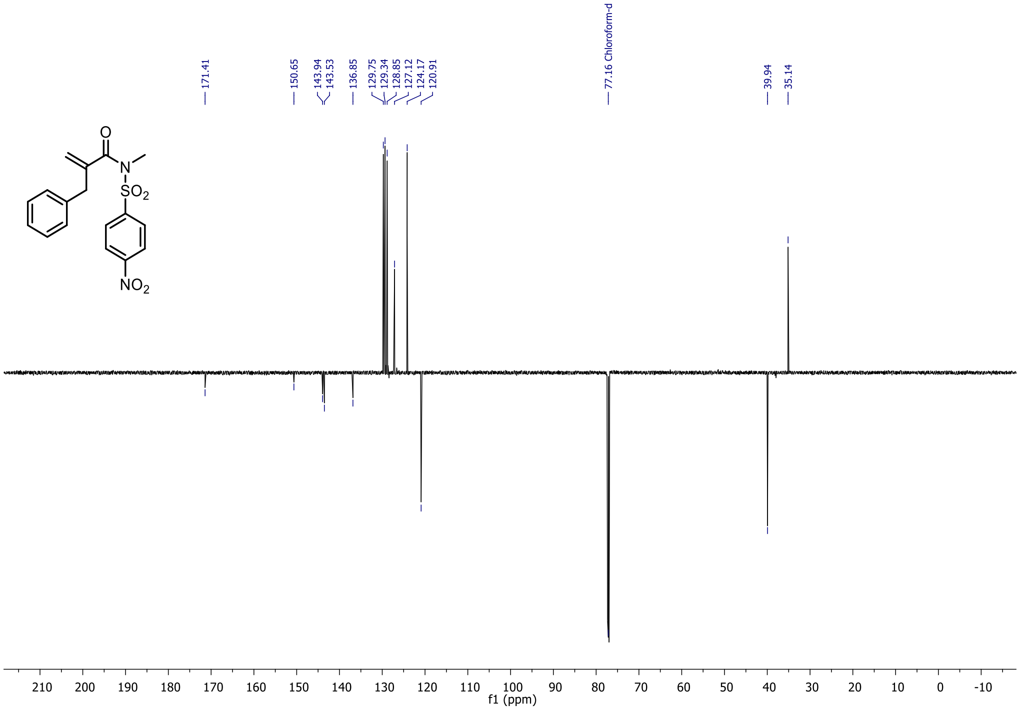

8. References

(1) Sheldrick, G. M. SHELXT - Integrated Space-Group and Crystal-Structure Determination. *Acta Crystallogr A Found Adv* **2015**, *71* (Pt 1), 3–8.

(2) Sheldrick, G. M. Crystal Structure Refinement with SHELXL. *Acta Crystallogr. B* **2015**, *71* (Pt 1), 3–8.

(3) Hübschle, C. B.; Sheldrick, G. M.; Dittrich, B. ShelXle: A Qt Graphical User Interface for SHELXL. *J. Appl. Crystallogr.* **2011**, *44* (Pt 6), 1281–1284.

(4) Lemmerer, M.; Zhang, H.; Fernandes, A. J.; Fischer, T.; Mießkes, M.; Xiao, Y.; Maulide, N. Synthesis of α-Aryl Acrylamides via Lewis-Base-Mediated Aryl/Hydrogen Exchange. *Angew. Chem. Int. Ed Engl.* **2022**, *61* (40), e202207475.

(5) Gonçalves, C. R.; Lemmerer, M.; Teskey, C. J.; Adler, P.; Kaiser, D.; Maryasin, B.; González, L.; Maulide, N. Unified Approach to the Chemoselective α-Functionalization of Amides with Heteroatom Nucleophiles. *J. Am. Chem. Soc.* **2019**, *141* (46), 18437–18443.

(6) Zhang, L.; Cheng, X.; Zhou, Q.-L. Electrochemical Synthesis of Sulfonyl Fluorides with Triethylamine Hydrofluoride. *Chin. J. Chem.* **2022**, *40* (14), 1687–1692.

(7) Izzo, J. A.; Myshchuk, Y.; Hirschi, J. S.; Vetticatt, M. J. Transition State Analysis of an Enantioselective Michael Addition by a Bifunctional Thiourea Organocatalyst. *Org. Biomol. Chem.* **2019**, *17* (16), 3934–3939.

(8) Day, D. M.; Farmer, T. J.; Sherwood, J.; Clark, J. H. An Experimental Investigation into the Kinetics and Mechanism of the Aza-Michael Additions of Dimethyl Itaconate. *Tetrahedron* **2022**, *121*, 132921.
